# Advancing foundational models in digital health technology adoption: A systematic literature review of multidisciplinary factors

**DOI:** 10.1371/journal.pdig.0001690

**Published:** 2026-09-02

**Authors:** Huey Yu Ng, Shamala Ramasamy, Saravanan Muthaiyah, Asnina Anandan, Seong Ting Chen, Thulasi Munohsamy, Gunasekar Thangarasu

**Affiliations:** 1 Centre for Postgraduate Studies for Research, Institute for Research, Development and Innovation, IMU University, Kuala Lumpur, Malaysia; 2 Department of Psychology and Counselling, School of Psychology and Social Sciences, IMU University, Kuala Lumpur, Malaysia; 3 School of Business and Technology, IMU University, Kuala Lumpur, Malaysia; 4 Division of Nutrition, Dietetics and Food Science, School of Health Sciences, IMU University, Kuala Lumpur, Malaysia; 5 Department of Social Sciences, School of Psychology and Social Sciences, IMU University, Kuala Lumpur, Malaysia; 6 Department of Digital Health and Health Informatics, School of Business and Technology, IMU University, Kuala Lumpur, Malaysia; University of Pittsburgh School of Medicine, UNITED STATES OF AMERICA

## Abstract

Digital health technologies (DHTs) such as patient portals, mobile applications, and electronic health records can improve access to healthcare, self-management and care coordination. However, their adoption remains inconsistent. This study systematically reviews the technological, psychological, social, cultural, health-related and environmental factors influencing DHT adoption. This protocol-registered review (PROSPERO: CRD420251056883) was conducted following the Preferred Reporting Items for Systematic reviews and Meta-Analyses (PRISMA) 2020 guidelines. A search of multidisciplinary databases was conducted through the EBSCO Discovery Service (EDS) to identify peer-reviewed English primary studies published between 2015 and 18 June 2025. Although studies published from 2015 onward were searched, only studies published from 2020 onward were retained for synthesis. Two researchers independently applied the Sample, Phenomenon of Interest, Design, Evaluation, Research type (SPIDER) framework to screen articles and extract data. As Covidence operationalises screening using the Population, Intervention, Comparison, Outcome, Study type (PICOS) framework, SPIDER elements were mapped to PICOS to ensure consistency across screening and extraction. Methodological quality was appraised using the Mixed Methods Appraisal Tool (MMAT) to inform interpretation. Data were synthesised using a structured thematic analysis workflow informed by the Thematic Analysis Matrix proposed by Zairul, which operationalises the thematic analysis principles described by Braun and Clarke. Coding, category development and theme generation were managed using ATLAS.ti (Version 24). Eighty-two studies published between 2020 and 2025 met the inclusion criteria. Five themes were identified: (1) access, equity and affordability; (2) usability, engagement and user empowerment; (3) trust, privacy and governance; (4) integration, workforce and sustainability; and (5) clinical effectiveness and quality of care. The findings showed that adoption of DHTs is a multi-layered process shaped by multidisciplinary factors. This review provides researchers, policymakers and healthcare providers with theoretical and practical insights to support sustainable, effective and equitable DHT adoption and to guide the development of future strategies.

## Author summary

Digital health technologies such as mobile health apps, patient portals and online health records are becoming part of everyday healthcare tools. These tools improve access to healthcare and support self-management of health. However, many individuals encounter difficulties when adopting these tools. To explore this issue, we reviewed 82 studies to identify the technological, psychological, social, cultural, health-related and environmental factors influencing the adoption of digital health technologies. We grouped these factors into five themes: access, equity and affordability; usability, engagement and user empowerment; trust, privacy and governance; integration, workforce and sustainability; and clinical effectiveness and quality of care. Our findings highlight the need for an integrated framework that combines multidisciplinary factors to strengthen existing technology adoption models. This framework can guide researchers, healthcare providers, policymakers and developers in developing digital health technologies that are equitable, scalable and effective.

## Introduction

DHTs such as digital health platforms, patient portals, mobile health (mHealth) applications, electronic health records (EHRs) and appointment scheduling applications are increasingly recognised for their potential to help address pressing challenges in health systems worldwide. These DHTs provide broader access to health information, support self-health management and enhance communication among healthcare providers, patients and caregivers [1,2]. With rapid global uptake, digital health has become an essential strategy for the future of healthcare delivery. Patients increasingly generate large volumes of health data outside clinical settings, raising the need for secure and user-friendly integration [1,3]. The COVID-19 pandemic has further accelerated the use of DHTs, telehealth platforms, mobile solutions and digital strategies to ensure the continuity of care [4–7]. The prospects of DHTs depend on their ability to transform healthcare services, improve outcomes and bridge the gaps in access and quality. They enable faster services, more efficient diagnosis and care coordination, as well as continuous patient monitoring through wearable and connected devices [8–11]. Beyond these clinical functions, they also promote digital and health literacy, empower patients in self-care and encourage active engagement with health management [8]. In doing so, DHTs can reduce pressure on overstretched health systems while supporting more scalable and patient-centred models of care. Within this context, DHT adoption is defined as the extent to which individuals, caregivers, or organisations accept and integrate DHTs into health management or health service delivery, and has become a central focus of digital health research [9].

Despite these benefits, DHT adoption continues to be hindered by persistent design and usability challenges. Poor interactive design is consistently identified as a major barrier to adoption. Users frequently encounter complex interfaces, unclear navigation and information overload, which increase cognitive burden and reduce engagement [1,2,12]. Recent empirical studies conducted between 2020 and 2025 across multiple countries consistently identify persistent usability and design-related barriers affecting DHT adoption. Studies conducted in the United Kingdom [13,14], Singapore [15,16], and Thailand [17,18] reported challenges related to complex user interfaces, poor navigation structures, and excessive information presentation, which negatively affected usability and user engagement. Similarly, studies conducted in Ethiopia [19], Saudi Arabia [20,21], and Malaysia [22–24] highlighted additional barriers, including limited localisation, language barriers, and insufficient accessibility features that constrained the adoption of DHTs. These usability-related issues were found to reduce perceived ease of use, user engagement, and adoption of DHTs across different healthcare settings [13,16,17,22]. Together, these studies demonstrate that usability-related barriers continue to affect DHT adoption across diverse healthcare and socio-cultural settings.

These usability-related failures increase cognitive burden, discourage adoption and contribute to digital fatigue among patients as well as frustration among clinicians [25–27], ultimately affecting engagement and continuous use of DHTs. Another challenge is the mismatch between developer assumptions and user needs. DHTs are often designed in a top-down manner with limited user involvement [28]. For instance, while developers may prioritise advanced analytics and visualisations, users often prefer simple, actionable feedback [28]. This gap reflects broader shortcomings in integrating user perspectives throughout the design and implementation process. Although user-centred design is widely used, researchers argue that technologies serving patients, clinicians, and caregivers simultaneously require multi-user-centred approaches [29]. Interoperability is also an important area for development, especially in supporting seamless data sharing across platforms [30]. Better integration and more standardised approaches may improve the ability of healthcare technologies to provide personalised and context-aware services. DHTs should also be developed not only as technological products but as healthcare services that fit human interactions and real-world healthcare contexts [12]. Principles of human-computer interaction (HCI) and intuitive design, which align system features with users’ expectations and mental models, are central to improving adoption and usability [1].

Existing empirical evidence further demonstrates that DHT adoption is influenced by a broad range of interconnected psychological, social, cultural, health-related, organisational and environmental factors beyond technological functionality alone. Psychological factors such as motivation, self-efficacy and emotional responses influence user engagement [31,32]. Social factors, including peer support, professional endorsement and caregiving relationships, shape users’ willingness to adopt DHTs across different contexts [33–36]. Cultural influences such as norms, stigma and value alignment affect perceptions of appropriateness and acceptance of digital health technologies [37–39]. For example, stigma surrounding mental health conditions and concerns regarding social judgment may discourage the use of mental health applications in certain populations [38,40]. Language mismatch, religious sensitivity and limited localisation may also reduce perceived relevance and appropriateness of DHTs across culturally diverse settings [37–39]. Health-related factors, including disease burden, perceived health needs and health literacy, determine the relevance and usability of DHTs for individuals [41,42]. Environmental and organisational factors such as infrastructure availability, affordability, organisational readiness and policy support act as enabling or constraining conditions for adoption [34,43,44]. Importantly, DHT adoption occurs across multiple stakeholder groups, including patients, caregivers, clinicians and healthcare administrators, each of whom may experience different contextual barriers, healthcare needs and adoption experiences [45]. Overall, these findings indicate that DHT adoption extends beyond a single behavioural or technological outcome and is shaped by interactions between technological systems, individual behaviours, healthcare conditions and contextual environments operating across multiple levels of healthcare ecosystems.

This is particularly important in chronic disease management, as non-communicable diseases such as cardiovascular diseases, diabetes and cancer remain the leading causes of death globally [46]. Although lifestyle changes can improve prevention and management, face-to-face intervention measures are difficult to scale up. Therefore, DHTs provide opportunities to deliver more scalable, personalised and accessible forms of support [47]. However, without equitable, inclusive and context-sensitive adoption strategies, the potential benefits of DHTs may not be fully realised across diverse healthcare settings and populations.

Accordingly, DHT adoption is increasingly understood as a dynamic and multi-level process rather than a one-time technological acceptance decision. Adoption may evolve over time through stages such as initial acceptance, use, discontinuance, and re-engagement [48,49]. Adoption outcomes are further shaped by interactions between individual, interpersonal, organisational, technological, and environmental conditions operating across different healthcare contexts [50–55].

However, existing technology adoption models continue to provide only partial explanations of these interconnected adoption processes. At the theoretical level, numerous models have been applied to explain DHT adoption. For example, the Technology Acceptance Model (TAM; Davis, 1989) [56], Unified Theory of Acceptance and Use of Technology (UTAUT; Venkatesh et al., 2003) [57], Health Belief Model (HBM; Rosenstock, 1974) [58], Theory of Planned Behavior (TPB; Ajzen, 1991) [59], Social Cognitive Theory (SCT; Bandura, 1986) [60] and Self-Determination Theory (SDT; Deci & Ryan, 1985) [61]. These frameworks largely focus on technological and behavioural constructs such as perceived usefulness, ease of use, social influence and performance expectancy. Although these constructs offer valuable insights, there remains an opportunity for existing models to comprehensively consider broader contextual influences such as cultural norms, emotional responses, health-related constraints, accessibility and environmental settings [56–61]. Most studies adopt a single theoretical perspective, while the multifaceted nature of DHT adoption across diverse healthcare settings and stakeholder groups may benefit from broader theoretical integration.

Building on the strengths of existing models, researchers have increasingly proposed integrated frameworks that combine constructs from multiple theoretical perspectives to broaden explanatory coverage in digital health contexts. Foundational models such as TAM, UTAUT, HBM, TPB, SCT and SDT are frequently integrated to incorporate broader behavioural, motivational and contextual considerations related to DHT adoption and health behaviour. However, several important gaps remain, including construct redundancy, insufficient emphasis on cultural and social impacts, limited validation across populations and inconsistent consideration of inclusivity-related dimensions. Existing integrated frameworks also vary considerably in their representation of accessibility, equity, language localisation, digital fatigue, data governance, organisational readiness, and environmental constraints [62–65]. Consequently, the theoretical understanding of DHT adoption may still benefit from further integration and contextual refinement. To provide a structured overview of commonly used integrated DHT adoption frameworks and the extent to which they represent inclusivity-related dimensions, Table 1 presents a comparative analysis of integrated models used in digital health adoption research.

**Table 1:** Conceptual comparison of integrated digital health technology adoption models.

Model	Key reference	Origin and theory base	Core constructs	Technological	Psychological	Social	Cultural	Health-related	Environmental
TAM-HBM	[66]	Combines TAM and HBM perspectives to predict health technology use	Perceived usefulness (PU), perceived ease of use (PEOU), perceived health risk, health consciousness	Explicitly represented (PU, PEOU)	Explicitly represented (perceived health risk, health consciousness)	Not explicitly represented	Not explicitly represented	Explicitly represented (perceived health risk, health consciousness)	Not explicitly represented
UTAUT-HBM	[67]	Integrates UTAUT and HBM perspectives to explain precision medicine adoption	Performance expectancy, price value, social influence, perceived threat	Explicitly represented (performance expectancy)	Explicitly represented (perceived threat)	Explicitly represented (social influence)	Not explicitly represented	Explicitly represented (perceived threat)	Partially represented (price value)
TAM-TPB	[68]	Combines TAM and TPB perspectives to interpret behavioural intention	PU, PEOU, attitude, subjective norm, perceived behavioural control (PBC)	Explicitly represented (PU, PEOU)	Explicitly represented (attitude, PBC)	Explicitly represented (subjective norm)	Not explicitly represented	Not explicitly represented	Not explicitly represented
UTAUT-TPB	[69]	Extends UTAUT and TPB to explain behavioural intention towards telemedicine use during COVID-19	Performance expectancy, effort expectancy, facilitating conditions, attitude, subjective norm, PBC	Explicitly represented (performance expectancy, effort expectancy)	Explicitly represented (attitude, PBC)	Explicitly represented (social influence, subjective norm)	Not explicitly represented	Not explicitly represented	Partially represented (facilitating conditions)
UTAUT-SDT	[70]	Combines UTAUT and SDT based-motivational constructs to describe the adoption of health-related sports applications	Performance expectancy, effort expectancy, facilitating conditions, autonomous, controlled motivation	Explicitly represented (performance expectancy, effort expectancy)	Explicitly represented (autonomous motivation, controlled motivation)	Not explicitly represented	Not explicitly represented	Not explicitly represented	Partially represented (facilitating conditions)
UTAUT-PMT	[71]	Integrates UTAUT, Protection Motivation Theory (PMT) perspectives to explain health technology adoption	Performance expectancy, social influence, facilitating conditions, perceived severity, perceived susceptibility, self-efficacy	Explicitly represented (performance expectancy)	Explicitly represented (self-efficacy)	Explicitly represented (social influence)	Not explicitly represented	Explicitly represented (perceived severity, perceived susceptibility)	Partially represented (facilitating conditions)
TAM-DTPB	[72]	Combines TAM and decomposed TPB to interpret telemedicine adoption among older adults	PU, PEOU, subjective norm, service environment and self-efficacy	Explicitly represented (PU, PEOU)	Explicitly represented (self-efficacy)	Explicitly represented (subjective norm)	Not explicitly represented	Not explicitly represented	Partially represented (service environment)
TAM-SCT	[73]	Combines TAM and SCT to explain health technology acceptance	PU, PEOU, self-efficacy objective and subjective usability	Explicitly represented (PU, PEOU, objective usability, subjective usability)	Explicitly represented (self-efficacy)	Not explicitly represented	Not explicitly represented	Not explicitly represented	Not explicitly represented

Therefore, a broader multidisciplinary perspective is needed to better understand DHT adoption across different healthcare and population settings. Although previous studies and integrated frameworks have examined some factors influencing DHT adoption, the evidence remains fragmented across technological, psychological, social, cultural, health-related, and environmental domains. In addition, the extent to which current frameworks represent broader contextual and inclusivity dimensions under real-world conditions remains unclear. This systematic literature review aims to identify and synthesise the factors influencing DHT adoption, examine existing theoretical and integrated frameworks, and provide an overview of the accessibility, inclusivity, and contextual factors represented within existing DHT adoption models.

## Materials and methods

This systematic literature review examines factors influencing DHT adoption from conceptual, theoretical and multidisciplinary perspectives, highlighting technological, psychological, social, cultural, health-related and environmental factors that complement existing technology adoption models. The review was conducted in accordance with the PRISMA 2020 guidelines across the identification, screening, eligibility and synthesis stages [74]. The protocol was registered in PROSPERO (CRD420251056883; S1 Text) on 26 May 2025 [75].

Prior to PROSPERO registration, a pilot search was conducted to refine the search strategy after preliminary searches retrieved a large number of records with limited relevance. No formal title/abstract screening, full-text screening, data extraction, methodological quality appraisal or evidence synthesis was conducted before registration.

Following registration, the protocol was amended twice, with both amendments documented in the PROSPERO record. The first amendment, published on 20 July 2025, refined the search strategy, clarified the eligibility criteria and updated methodological details while maintaining the original Malaysia-focused scope of the review. A second amendment, published on 5 February 2026, broadened the review into a global synthesis of multidisciplinary factors influencing digital health technology adoption, revised the review objectives and eligibility criteria, incorporated the Mixed Methods Appraisal Tool (MMAT) for methodological quality appraisal, and clarified the review timeline. These amendments were made to improve methodological alignment and transparency rather than in response to the review findings. Study selection had already been completed before the second amendment; however, the subsequent data analysis and evidence synthesis were conducted in accordance with the revised protocol. The amendments are documented in the PROSPERO record and reflected in the final methodology.

### Research framework

The SPIDER framework was adopted to structure the review because it is well-suited to integrating qualitative, quantitative and mixed-methods evidence, thereby capturing both empirical findings and conceptual explanations of DHT adoption influencing factors [76,77]. PICOS was not used as the primary framework, as it is primarily designed for intervention-focused effectiveness reviews and is less appropriate for synthesising multidisciplinary explanatory factors [76,77]. As Covidence operationalises screening using the PICOS framework, the SPIDER criteria were mapped to their closest PICOS equivalents to enable systematic screening without altering the conceptual orientation of the review. SPIDER therefore guided eligibility decisions and data extraction, while PICOS was used solely as a technical tool for screening within Covidence. The resulting SPIDER-PICOS mapping, eligibility criteria and their application during screening and data extraction are presented in Table 2.

**Table 2:** SPIDER-PICOS mapping, eligibility criteria and their application during study screening and data extraction.

Element	PICOS equivalent	Include	Exclude	Application during title/abstract and full-text screening	Application during data extraction
Sample (S)	Population (P)	Studies involving individuals or groups using DHTs such as apps, wearables, platforms and systems in healthcare contexts, in the real world or research settings.	Studies not involving human participants; studies where no DHT is used.	Used at the title/abstract screening stage to identify studies involving human users of DHTs within healthcare contexts.	Extracted participant type, user group, demographic characteristics, and population context.
Phenomenon of interest (PI)	Intervention/Exposure (I)	Studies that identify, examine, or analyse factors influencing the adoption of DHTs, including technological, psychological, social, cultural, health-related, or environmental aspects.	Studies not investigating adoption; studies focusing solely on general-purpose AI tools, such as ChatGPT, Gemini and/or Microsoft Copilot, without integration into DHTs.	Used to include studies explicitly examining DHT adoption.	Extracted adoption-related focus, behavioural context, and influencing factors.
Design (D)	Study characteristics (S)	Qualitative, quantitative, or mixed-methods primary empirical studies with clear data collection and analysis methods; studies reporting DHT adoption influencing factors as explicit outcomes; studies providing conceptual or theoretical interpretation of findings.	Studies without a structured design; secondary research (systematic reviews, scoping reviews, narrative reviews, meta-analyses); studies that exclusively conduct statistical testing, such as structural equation modeling (SEM), regression and prediction without conceptual or theoretical explanation of DHT adoption influencing factors.	Used at the full-text screening stage to exclude studies lacking empirical rigour or conceptual grounding.	Extracted study design, methodological approach, and analytical framework.
Evaluation (E)	Outcome (O)	Studies reporting multidisciplinary factors influencing adoption of DHTs; studies proposing, adapting, or analysing frameworks/models where DHT adoption influencing factors are conceptually, theoretically or interpretively discussed.	Studies that do not report any DHT adoption influencing factors; studies that only performed predictive or statistical testing of frameworks/models without providing conceptual, theoretical or interpretive discussion of DHT adoption influencing factors.	Used to identify and confirm conceptually or empirically discussed DHT adoption influencing factors.	Extracted technological, psychological, social, cultural, health-related, and environmental factors.
Research type (R)	Other	Peer-reviewed primary research, published in English between 2020 and June 2025, conducted globally.	Non-peer-reviewed sources; studies published before 2020; studies without full-text availability.	Used to confirm eligibility based on publication type, language, and timeframe; studies published before 2020 were excluded at the full-text screening stage.	Extracted publication year, research type, and analytical orientation.
–	Comparator/Context (C)	Not applicable			

During title/abstract screening, the Sample and Phenomenon of Interest criteria were applied to ensure inclusion of studies examining DHT adoption or non-adoption. At the full-text screening stage, the Design and Evaluation criteria ensured that included studies contributed empirical or conceptual evidence on influencing factors rather than merely testing existing technology adoption models. During data extraction, the Evaluation and Research Type criteria supported the systematic identification of multidisciplinary factors, which were organised into technological, psychological, social, cultural, health-related and environmental domains to facilitate structured synthesis.

### Search strategy

The search strategy was built around five domains: (1) digital health technology; (2) adoption behaviour; (3) theories and models; (4) obstacles and limitations; and (5) multidisciplinary influencing factors. These domains were selected to capture technical, behavioural and contextual dimensions of DHT adoption and to support theory-informed interpretation aligned with the review objective. Trial runs of search strings were conducted to optimise the sensitivity-precision balance, using Boolean operators, truncation and phrase searching to improve retrieval accuracy [78–82]. The final search strings are shown in Table 3.

**Table 3:** Final search strings.

Key domains	Description	Search terms
DHTs	Capturing various digital technologies used in healthcare and self-care settings.	• digital health technolog*
Adoption behaviour	Exploring the adoption of DHTs.	• technology adoption • technology acceptance
Theories and models	Investigating existing theories, models and frameworks related to the adoption of DHTs.	• model* • framework* • theor*
Obstacles and limitations	Targeting barriers, issues or limitations in the adoption of DHTs, while allowing facilitators or enabling factors to be identified during screening and synthesis when reported within included studies.	• limitation* • barrier* problem* • • issue*
Multidisciplinary influencing factors	Covering the technological, psychological, social, health, cultural and environmental factors related to the adoption of DHTs.	• psychological factor* • social factor* • cultural factor* • environmental factor* • health condition*
Final search string	(“digital health technolog*”) AND (“technology adoption” OR “technology acceptance”) AND (model* OR framework* OR theor*) AND (limitation* OR barrier* OR problem* OR issue*) AND (“psychological factor*” OR “social factor*” OR “cultural factor*” OR “environmental factor*” OR “health condition*”)	

During protocol development, broader exploratory pilot search strings were tested, but these retrieved a large volume of records with limited conceptual relevance to the review focus and eligibility criteria. Consequently, the final search strategy prioritised specificity by requiring the concurrent presence of all five domains, without applying country, region or health system filters to retain global coverage. This strategy may have overlooked some qualitative studies that focused on practical aspects of DHT adoption without explicit reference to theory, and this is acknowledged as a limitation.

Literature retrieval was conducted through the EDS, which provides access to Scopus, ACM Digital Library, MEDLINE and other multidisciplinary databases [83–85]. EDS was used to enable cross-disciplinary retrieval via a single platform and to reduce duplicates and screen burden. Additional EDS search features, including automatic “AND” insertion, synonym expansion and full-text searching, were utilised to support comprehensive retrieval of relevant studies [86,87]. The search covered peer-reviewed, English-language studies published between 2015 and 18 June 2025 to capture contemporary DHTs development and adoption trends, and the final synthesis retained only studies published from 2020 onwards, consistent with the predefined inclusion criteria.

### Inclusion and exclusion criteria

The eligibility criteria were defined using the SPIDER framework (Table 2) and aligned with the review objectives. Included studies were primary, peer-reviewed empirical studies employing qualitative, quantitative or mixed-methods designs that reported technological, psychological, social, cultural, health-related, or environmental factors influencing DHT adoption among stakeholders across diverse demographic groups such as patients, caregivers, children, young adults, older adults, healthcare professionals, healthcare administrators, academic researchers, technology developers, IT personnel, or government and regulatory stakeholders.

Secondary studies, including systematic reviews, scoping reviews and meta-analyses, were excluded as they do not generate original empirical evidence [88]. Studies lacking a clearly described methodological approach were also excluded to maintain interpretability and analytical rigour.

The publication timeframe was defined to ensure the evidence base reflects modern DHT development and adoption contexts. The initial search period (2015–18 June 2025) was selected because DHT increasingly emerged during this period as an umbrella concept encompassing earlier domains such as telemedicine, eHealth and mHealth, supported by widespread smartphone adoption and advancing digital infrastructures [89].

However, the final synthesis prioritised studies published from 2020 onward, as this period represents accelerated and system-level DHT adoption associated with the COVID-19 pandemic. During this phase, DHTs transitioned from optional innovations to essential components of healthcare delivery for prevention, monitoring, diagnosis, and continuity of care [90,91]. Studies published between 2015 and 2019 were screened according to the predefined eligibility criteria but were excluded at the full-text screening stage, as they predominantly reflected early-stage or exploratory DHT adoption contexts that differed conceptually from post-pandemic DHT use. Therefore, the final synthesis included studies published between 2020 and June 2025 to ensure conceptual and contextual comparability across included studies.

Studies focusing on general-purpose artificial intelligence (AI) tools such as ChatGPT, Gemini or Microsoft Copilot were excluded unless these technologies were embedded within or evaluated as part of a DHT system or health service context.

To ensure transparency, methodological consistency, and reproducibility, the review was restricted to peer-reviewed journal articles published in English. English-language studies were prioritised because English is the dominant language of scholarly communication in DHT adoption and health behaviour research. Also, resources for high-quality translation were limited, thereby increasing the risk of misinterpretation during data extraction and thematic analysis [92,93].

Grey literature, including conference proceedings, theses, dissertations, technical reports and other non-peer-reviewed materials, was excluded at the title/abstract screening stage. This decision was made to maintain a consistent quality threshold, given the absence of standardised peer-review processes and the challenges such sources pose for methodological appraisal, comparability and reproducibility [93,94].

### Data selection and extraction

All retrieved studies were imported into Covidence to facilitate duplicate removal, title/abstract screening, full-text screening, and structured study management [95]. To enhance reliability and minimise selection bias, two reviewers independently screened titles/abstracts and full texts, resolving disagreements through a third reviewer [95,96].

**Fig 1:**
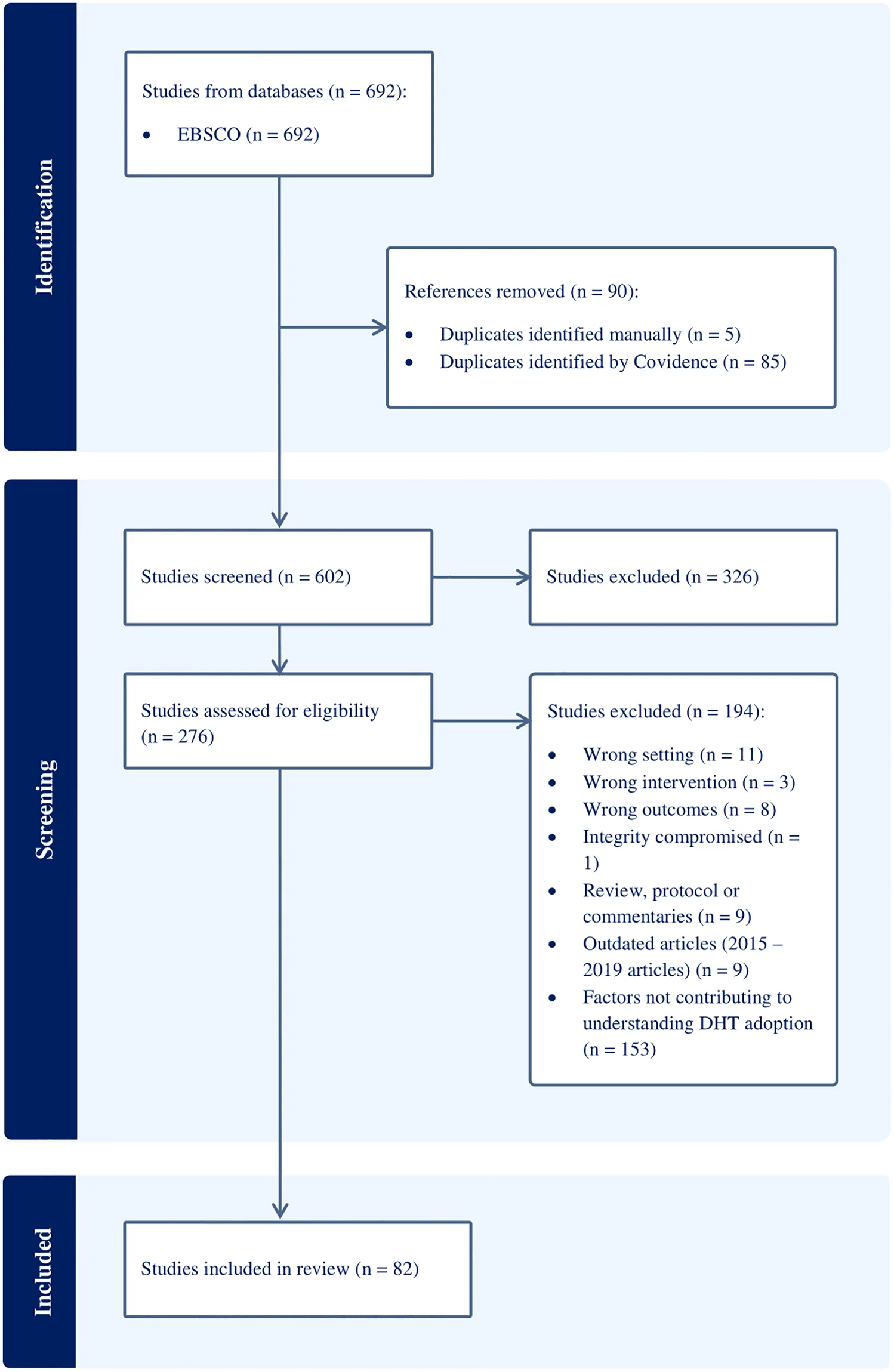
PRISMA flow chart.

The process followed PRISMA 2020 guidelines [74]; the S1 File guided reporting, and the study selection process is presented in Fig 1.

Data extraction was conducted using a predefined Microsoft Excel extraction form to capture study and sample characteristics, adoption phenomena, study design, influencing factors, research type, and reported country, setting and population characteristics. Extracted data were checked by a second reviewer for accuracy and consistency, with discrepancies resolved through discussion.

In line with Covidence’s system design, specific reasons for exclusion were not recorded at the title/abstract screening stage, as this phase was used to exclude records that clearly did not meet the inclusion criteria based on scope, focus, population or relevance to DHT adoption. For transparency, complete lists of records excluded at the title/abstract screening stage (S1 Table 1) and full-text screening stages, with reasons (S1 Table 2), are provided. When study characteristics or contextual details were not reported, these were recorded as “not reported”, and no imputation was performed.

The extracted data were imported into ATLAS.ti for coding and thematic synthesis to support systematic organisation and synthesis of qualitative evidence.

### Data analysis and synthesis

**Fig 2:**
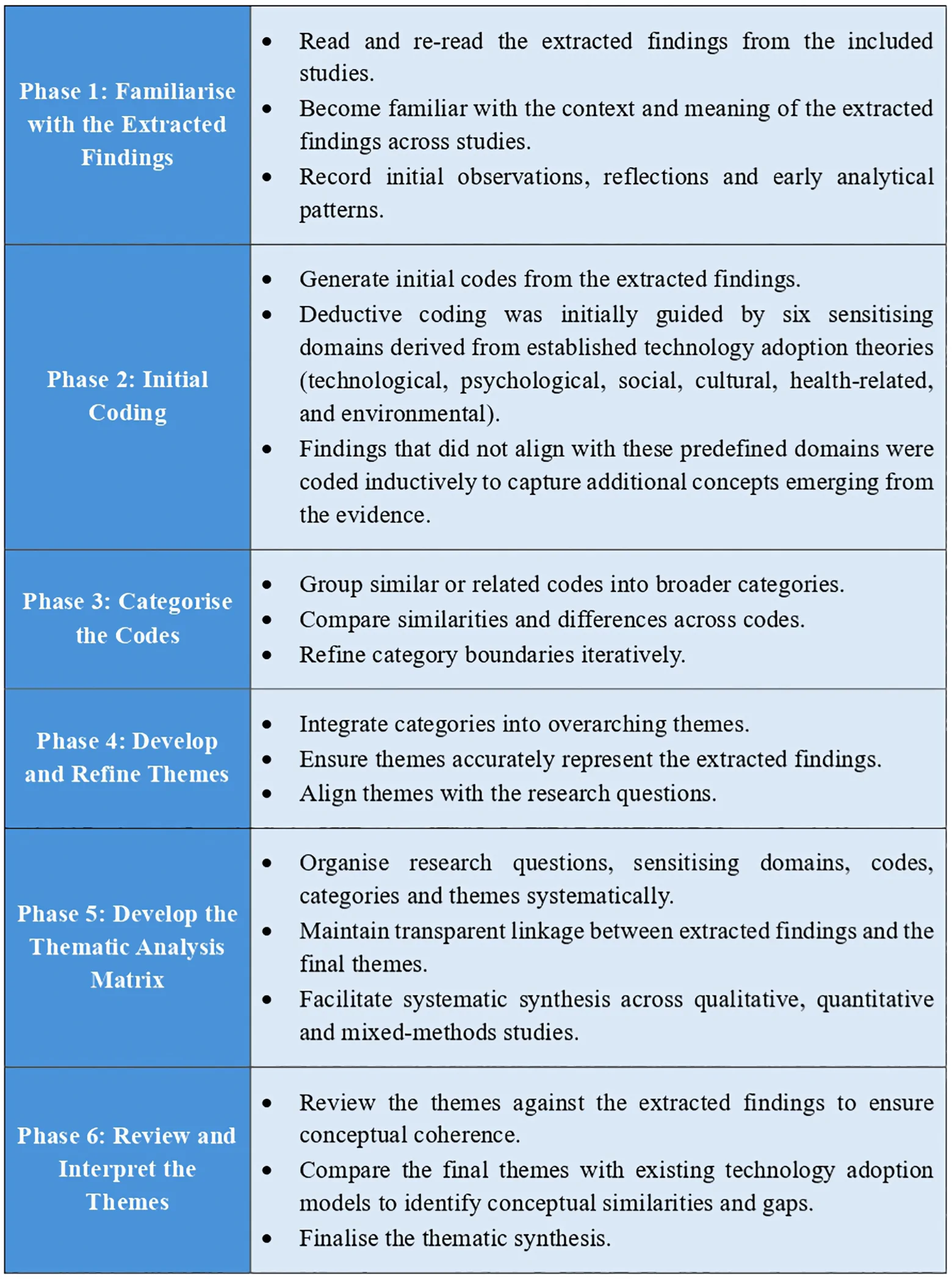
Structured thematic analysis workflow.

Data analysis was conducted using the Thematic Analysis Matrix proposed by Zairul [97], which provides a structured workflow for organising qualitative synthesis and operationalises the thematic analysis principles described by Braun and Clarke [98,99]. The matrix organised the progression from familiarisation, coding, category development and theme generation while maintaining clear relationships between the research questions, analytical domains, codes, categories and final themes. Coding, category development and theme generation were managed using ATLAS.ti (Version 24). The analysis followed an iterative process of familiarisation, coding, category development and theme refinement, as illustrated in Fig 2.

Initial coding involved systematic coding of the extracted findings to identify explicit and implicit factors influencing DHT adoption. Deductive coding was initially guided by six domains derived from established technology adoption theories (technological, psychological, social, cultural, health-related and environmental). Where extracted findings did not align with these predefined domains, new inductive codes were generated. Related codes were subsequently grouped into conceptually similar categories before being refined into higher-order themes representing common adoption patterns across the included studies. To enhance analytical rigour, emerging codes and preliminary themes were reviewed through supervisory discussions to clarify code definitions, examine alternative interpretations and refine theme boundaries. Differences in interpretation were resolved through discussion before the thematic structure was finalised. The six predefined domains remained the overarching analytical framework throughout coding. The final themes were subsequently developed by synthesising relationships across these domains. Thus, the themes represent higher-order synthesis themes rather than additional analytical domains. Themes and subthemes were then reviewed against the extracted data to ensure internal coherence and clear conceptual boundaries. Refinement involved merging overlapping categories, clarifying definitions and discarding weak themes, with peer debriefing informing revisions until conceptual stability was achieved.

Following theme development, a thematic interpretive comparison was conducted to compare the identified themes with constructs represented in existing technology adoption models. The relative prominence of thematic domains was interpreted descriptively based on thematic patterns rather than statistical effect sizes or causal inference. The analysis concluded after all extracted findings had been systematically coded, reviewed and organised into coherent themes representing the included evidence. The synthesis emphasised structured interpretation of multidisciplinary adoption factors consistent with the objectives of integrative qualitative evidence synthesis [100].

### Quality and bias considerations

Following inclusion, methodological quality appraisal was conducted using the MMAT (2018) to inform interpretation rather than eligibility decisions [101]. MMAT was selected to enable consistent appraisal across qualitative, quantitative, and mixed-methods studies. Studies were assessed using the two MMAT screening questions, followed by the design-specific criteria appropriate to each methodological category [101]. All included studies met the two MMAT screening criteria, indicating clearly stated research questions and data appropriate to the stated objectives, and were therefore eligible for quality appraisal and synthesis. Appraisal was conducted by the primary reviewer and checked by a second reviewer, with discrepancies resolved through discussion. No overall numerical quality score was applied, and appraisal findings were used to contextualise the evidence base [101]. For transparency, patterns in the extent to which MMAT criteria were met across studies were summarised descriptively to support interpretation, without applying categorical thresholds or composite scores [101].

Quality appraisal findings were used interpretively during synthesis. Studies meeting fewer MMAT criteria were retained but interpreted with caution. To assess the influence of study quality, themes were examined across studies demonstrating variation in methodological completeness to ensure that core findings were not based solely on studies meeting fewer criteria. This supported the robustness of the synthesis, while acknowledging variation in methodological strength across the evidence base.

### Rigour and data governance

Methodological rigour was enhanced through systematic and transparent analytical procedures. Consistent coding procedures were applied throughout the review, and the development of categories and themes was documented within the thematic analysis matrix. Codes, categories and themes were reviewed iteratively to refine conceptual boundaries and coding decisions. Key methodological decisions were documented throughout the review to enhance transparency and reproducibility. Data governance procedures included secure data storage and controlled access to analytical materials.

### Ethical considerations

The review was approved by the IMU University Joint Committee on Research and Ethics (IMU-JC), project code MMHS I-2025(07). The review was conducted in accordance with research integrity standards and with appropriate acknowledgement of all sources.

## Results

### Study selection and screening

A total of 692 records were identified through database retrieval. After duplicate removal and screening of titles, abstracts and full texts, 82 studies met the inclusion criteria. The selection process and exclusion reasons are illustrated in Fig 1.

### Study characteristics

A descriptive overview of the included studies is presented in Table 4, summarising their distribution by publication year, country or region, study population and methodological approach. The number of included studies increased over time, with a peak observed in 2024. Qualitative approaches were the most commonly employed methodology, followed by cross-sectional survey designs, while mixed methods, longitudinal studies, Delphi consensus techniques and policy analyses were less frequently reported. The included studies covered numerous countries and diverse research settings, with samples drawn from the United States, the United Kingdom and Saudi Arabia being among the most frequently represented. The research settings included institutional clinical environments, primary care systems, community-based contexts and digital or online platforms. The study population were heterogenous and included healthcare professionals, the general public, patients with chronic diseases, older adults and mixed stakeholder groups, while caregivers and students were less frequently represented. Most studies collected data through surveys and interviews, with some studies also employing usage logs, usability testing or policy reviews. The detailed characteristics of the included studies, including study design, country/setting, population characteristics, data collection methods, main findings and reported study limitations, are provided in the S2 Table.

**Table 4:** Characteristics of included studies.

Category	Subcategory	Frequency (n)
Publication Year	2020	4
	2021	7
	2022	11
	2023	14
	2024	29
	2025	17
Country	Australia	5
	Bulgaria	1
	Cambodia	1
	Canada and United States	1
	China	5
	Ethiopia	3
	Finland	1
	Finland & Ghana	1
	Germany	2
	Hong Kong	1
	India	4
	Indonesia	2
	Ireland	2
	Israel, Germany, and United Kingdom	1
	Italy	2
	Malawi	1
	Netherlands	1
	New Zealand	1
	Nigeria	1
	Norway	1
	Romania	1
	Saudi Arabia	8
	Singapore	5
	South Africa	1
	South Korea	1
	Spain	1
	Switzerland	1
	Taiwan	3
	Tanzania	1
	Uganda	2
	United Arab Emirates	2
	United Kingdom	8
	United Kingdom and United States	1
	United States	10
Population	Caregivers	3
	General public	28
	Healthcare professionals	17
	Mixed stakeholders	12
	Patients	22
Study Design	Case study	1
	Cross-sectional survey	25
	Mixed methods	13
	Others	1
	Qualitative	38
	Quantitative (Secondary data analysis)	4

### Thematic analysis

**Fig 3:**
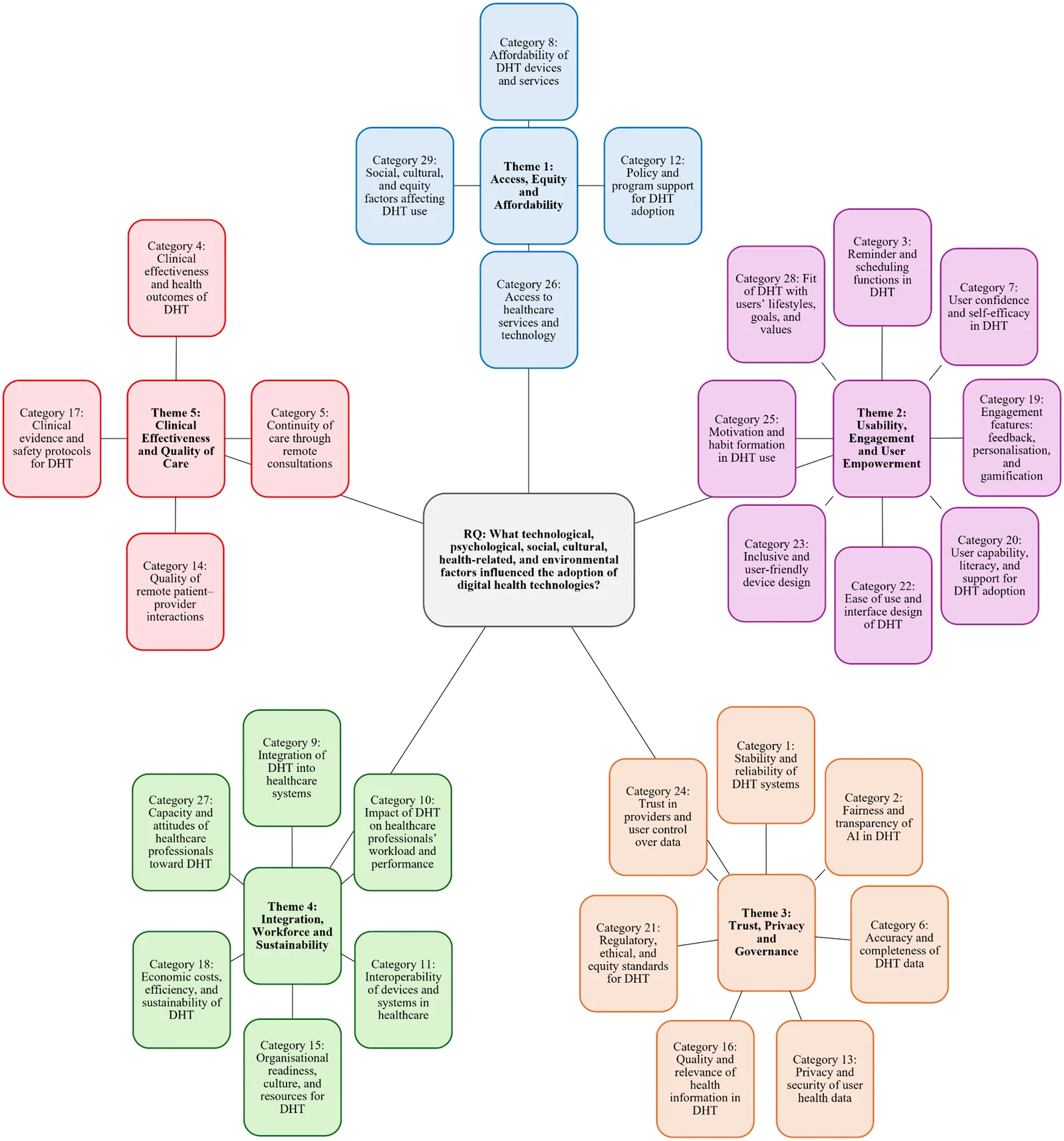
Conceptual map of DHT adoption factors.

Illustrative quotations with the corresponding codes are provided in S3 Table, and the progression from initial coding through category development to the final themes is documented in S1 Appendix. The thematic synthesis incorporated codes organised within the six analytical domains while allowing additional inductive concepts to emerge where appropriate. These codes were consolidated into conceptually similar categories and synthesised into final themes, enabling systematic comparison of multidisciplinary influences on DHT adoption across the included studies. The resulting thematic structure is presented in Fig 3.

#### Theme 1: Access, equity and affordability.

The availability of internet connectivity, information and communication technology (ICT) infrastructure and device ownership determines the adoption [102–105]. Barriers include unreliable power supply, poor network connectivity and the lack of smartphones, especially in rural areas [106–109]. The benefits, such as faster consultation and timely feedback, highlight the perceived value of DHT use [105,106,110]. Affordability significantly impacts users’ adoption and usage. The costs of devices, subscriptions and data plans were found to hinder DHT adoption [111–114]. Subsidies, insurance and cost savings promoted engagement, although hidden costs reduced confidence [106,108,115,116]. Community measures such as workshops, subsidies and pilot projects have expanded DHT adoption, but scalability and equitable distribution remain challenges [117–120]. Social and cultural backgrounds, including family encouragement, peer support, stigma and caregiving burdens, further influence uptake of DHTs [121–124]. Vulnerable populations, such as older adults, the disabled and rural residents, have been disproportionately affected [125–128]. The COVID-19 pandemic accelerated DHT adoption while simultaneously exposing structural inequities in access and use [111,129,130].

#### Theme 2: Usability, engagement and user empowerment.

Easy-to-use and intuitive design are the prerequisites for adoption, while complex systems reduce user interest [131–134]. Accessibility features such as multilingual options, ergonomic wearable devices and portability are valued [107,128,135–137]. Reminder and scheduling functions facilitate DHT adoption and support user engagement; notification overload can reduce effectiveness [105,109,111,128,129]. Adoption motivation is usually derived from health awareness, curiosity and external incentives and accountability [117,137–140]. Interactive elements, such as feedback, personalisation and gamification, enhance satisfaction, while poorly designed feedback and imbalanced gameplay difficulty reduce trust [109,119,126,139,141]. Digital and health literacy determine the ability to adopt DHT. Structured training and multimedia-assisted learning support engagement [110,113,122,133,142]. Confidence and self-efficacy enhance adoption, while fear and resistance limit it [117,125,143,144]. DHTs that align with users’ lifestyles and values are more likely to be adopted [110,127,145–147].

#### Theme 3: Trust, privacy and governance.

Trust in DHTs depends on the accuracy, clarity and relevance of the information. Concerns over data reliability, incomplete records and system instability have eroded confidence [109,113,131,148]. Users demand strong privacy protection, including encryption, anonymisation and clear policies [106,149–151]. A weak or fragmented legal and ethical framework has discouraged adoption, which has been further intensified by unclear ownership, insufficient regulation and bias [105,108,116,117]. As users value transparency and voluntary usage, trust in providers and autonomy over data are of vital importance [114,120,134,152]. The artificial intelligence (AI)-driven functions have raised users’ interest and doubt, demanding fairness, explainability and human oversight [103,120,153–155].

#### Theme 4: Integration, workforce and sustainability.

Interoperability among devices, applications and health records is a determining factor. Integration into the healthcare system is essential, but it is often limited by outdated hospital systems and fragmented practices [105,118,142,156]. Healthcare professionals have played the role of gatekeepers, with their digital literacy, adaptability and concerns about responsibility for influencing DHT adoption [104,105,151,156]. Some professionals have recognised the improvement in efficiency, while others have reported an increase in workload and disruptions in the workflow [102,117,135,156]. Organisational readiness, leadership and resources enabled adoption, while poor planning and personnel shortages reduced preparedness [102,105,156,157]. Economic barriers include funding gaps, maintenance costs and reliance on external suppliers, all of which raise issues related to system-level, rather than individual user adoption [105,106,117,120].

#### Theme 5: Clinical effectiveness and quality of care.

When DHTs demonstrate clinical validation, safety and oversight, their adoption is strengthened [134,143,156]. Certification, structured safety protocols and professional monitoring have reduced risks [143,144,155,158]. Visible health benefits such as improved monitoring and service quality have encouraged engagement [117,136,144,159]. Remote consultation supports the continuity of care, but is limited by users’ preference for face-to-face assurance [145,160,161]. Communication quality shapes satisfaction. Empathy, clarity and responsiveness are valued, while poor webside manner undermined user confidence and DHT adoption [116,123,133,153,162].

Overall, these five themes show that DHT adoption is influenced by multiple interrelated factors. These five themes represent higher-order synthesis themes developed from the coded data rather than additional analytical domains. Organisational readiness, workforce capacity, affordability and equity emerged as cross-cutting concepts across multiple analytical domains and were synthesised within the final themes rather than treated as independent analytical domains. Psychological factors were prominently presented in themes 2, 3 and 5, suggesting that they play a central role in shaping user motivation, confidence, trust and engagement during DHT adoption. Social and cultural factors also appeared particularly in themes 1, 2 and 5. Social influences reflected the role of family support, professional endorsement, peer norms and caregiving relationships. Cultural factors shaped attitudes toward technology, perceptions of appropriateness, stigmatisation, and value alignment. These factors influenced individuals’ willingness to adopt DHTs across different populations and contexts.

Health-related factors were observed in themes 1, 2 and 5. Users’ health conditions, perceived clinical relevance, disease management needs and expectations of care quality affected DHT adoption and engagement. These factors linked personal health status with perceived benefits of DHT use. Environmental, together with organisational influences, was primarily concentrated in Themes 1 and 4. Infrastructure availability, affordability, workforce readiness, organisational support and system-level integration influence DHT accessibility, scalability, and sustainability. Technological factors were mainly represented in themes 1, 3 and 4. These factors acted as facilitating or constraining conditions that interacted with psychological, social and health-related influences rather than functioning independently.

The findings also show that these domains are closely interconnected, as many included studies addressed multiple domains simultaneously rather than examining them as separate categories. Technological, psychological and social factors were commonly reflected across the identified themes, while environmental, organisational, cultural and health-related influences appeared in more context-specific ways. Environmental factors, together with organisational influences, were mainly related to infrastructure, affordability and system-level integration. These patterns suggest that existing technology adoption models provide important insights into individual-level cognitive and behavioural factors, while cultural, environmental, organisational, and equity-related influences may also be important to consider.

### Methodological quality assessment (MMAT)

An overview of methodological quality across all included studies is presented in S4 Table, which summarises MMAT design classifications and patterns in the extent to which core methodological criteria were met, grouped by study type. Full item-level MMAT assessments for each individual study, including criterion-by-criterion judgements and justifications, are provided in S2 Appendix.

#### Qualitative studies.

Most qualitative studies met the majority of applicable MMAT qualitative criteria, including the appropriateness of the qualitative approach, adequacy of data collection methods, coherence between data, analysis, and interpretation and substantiation of findings. Across studies, all or nearly all applicable qualitative criteria were satisfied, with only isolated limitations, most commonly related to sample scope or contextual specificity rather than analytic rigour.

#### Quantitative descriptive and quantitative non-randomised studies.

Quantitative studies showed greater variation in the extent to which MMAT criteria were fulfilled. In general, key criteria related to clear research questions and suitable measures were met. However, several studies did not fully meet criteria related to representativeness, response rates or handling of missing data. These limitations are mainly attributed to the cross-sectional research designs and the reliance on self-reported data. Overall, most quantitative studies met several, but not all, applicable MMAT criteria and findings from these studies were interpreted with appropriate caution.

#### Mixed-methods studies.

Mixed-methods studies generally met MMAT criteria for their qualitative and quantitative components. However, variability was observed in the extent to which the integration criterion was fulfilled. Some studies provide clear and concise explanations of how qualitative and quantitative findings were integrated, while others report the two components in parallel with limited methodological linkage. Where integration was clearly described, studies offered more comprehensive and multi-layered insights.

#### Influence of study quality on synthesis.

Themes were examined across studies demonstrating variation in methodological completeness to ensure that the synthesis was not driven solely by studies meeting fewer MMAT criteria. Core themes were consistently supported by studies that fulfilled a greater number of applicable MMAT criteria, while studies meeting fewer criteria contributed contextual or supplementary insights. The convergence of findings across studies with different methodological strengths enhances the robustness and credibility of the synthesised themes.

## Discussion

This review synthesised multidisciplinary factors influencing DHT adoption and identified five higher-order synthesis themes. Five higher-order synthesis themes were identified: (1) access, equity and affordability; (2) usability, engagement and user empowerment; (3) trust, privacy and governance; (4) integration, workforce and sustainability; and (5) clinical effectiveness and quality of care. Together, these findings demonstrate that DHT adoption is shaped by interacting individual-level, relational, and system-level determinants rather than isolated cognitive perceptions. The findings suggest that DHT adoption is conceptualised as a dynamic, context-sensitive and multi-level process shaped by interactions between technological systems, individual experiences and broader healthcare environments.

Existing models such as TAM, UTAUT, HBM, SCT and SDT have been instrumental in explaining perceptions, behavioural intentions and motivational processes related to technology adoption [56–61]. Emerging integrated DHT adoption models attempt to combine constructs from multiple theoretical perspectives to provide a more comprehensive understanding of digital health adoption behaviours. The findings of this review suggest that existing integrated adoption models collectively represent a broad range of multidisciplinary factors, while the emphasis placed on different constructs varies across models. Individual-level cognitive and motivational constructs, such as usability, perceived usefulness, motivation and self-efficacy, are consistently represented, whereas organisational readiness, affordability, governance, infrastructure accessibility and equity-related influences provide additional multidisciplinary perspectives that may complement existing models.

Table 1 provides a conceptual comparison of constructs represented across existing integrated digital health technology adoption models. Building on this comparison, Table 5 maps the higher-order synthesis themes identified in the present review against these model constructs, enabling a thematic comparison between the review findings and existing theoretical frameworks. This comparison highlights areas of conceptual alignment while illustrating how the multidisciplinary perspectives identified in this review complement existing adoption models. The comparison indicates that constructs related to usability, perceived usefulness, motivation and self-efficacy are consistently represented across existing models. Organisational readiness, affordability, governance, infrastructure accessibility and equity-related influences are also reflected in several frameworks and offer opportunities to further enrich multidisciplinary understanding of DHT adoption. For example, TAM-SCT incorporates usability and self-efficacy, while infrastructural access and affordability can further contribute within DHT adoption contexts [73]. Similarly, UTAUT-SDT emphasises motivational dimensions and can be complemented by considering organisational readiness and sustainability-related influences [70]. Overall, the findings demonstrate that existing integrated DHT adoption models provide valuable perspectives on digital health technology adoption, while the multidisciplinary themes identified in this review offer complementary contextual and system-level considerations that can support a more comprehensive understanding of equitable implementation across diverse real-world settings.

**Table 5:** Mapping synthesis themes to adoption models.

Integrated model	TAM-HBM	UTAUT-HBM	TAM-TPB	UTAUT-TPB	UTAUT-SDT	UTAUT-PMT	TAM-DTPB	TAM-SCT
**Access, Equity and Affordability** (ICT access, device ownership, affordability, subsidies, policy support, social/cultural inequities, vulnerable groups)	Not explicitly represented	Partially represented (price value)	Not explicitly represented	Partially represented (facilitating conditions)	Not explicitly represented	Not explicitly represented	Partially represented (service environment)	Not explicitly represented
**Usability, Engagement and User Empowerment** (Ease of use, accessibility, reminders, habit formation, feedback, gamification, literacy, self-efficacy, lifestyle fit)	Explicitly represented (PU, PEOU)	Explicitly represented	Explicitly represented	Explicitly represented	Explicitly represented (motivation)	Partially represented	Explicitly represented	Explicitly represented (usability, self-efficacy)
**Trust, Privacy and Governance** (Information accuracy, system reliability, privacy/security, regulation, data ownership, AI transparency)	Partially represented (risk-related concerns)	Partially represented (perceived threat)	Not explicitly represented	Not explicitly represented	Not explicitly represented	Partially represented (threat appraisal)	Not explicitly represented	Not explicitly represented
**Integration, Workforce and Sustainability** (Interoperability, system integration, HCP capacity, workload, organisational readiness, economic sustainability)	Not explicitly represented	Not explicitly represented	Not explicitly represented	Partially represented (facilitating conditions)	Not explicitly represented	Not explicitly represented	Not explicitly represented	Not explicitly represented
**Clinical Effectiveness and Quality of Care** (Clinical validation, safety, outcomes, continuity of care, communication quality)	Partially represented (health beliefs)	Partially represented	Not explicitly represented	Not explicitly represented	Not explicitly represented	Partially represented (severity, susceptibility)	Not explicitly represented	Not explicitly represented

Access, equity, and affordability emerged as fundamental prerequisites for adoption. Internet connectivity, device ownership and stable electricity were essential facilitators, while subscription costs and data charges created barriers, with financial and infrastructural gaps reinforcing divides [4,163,164]. Demographic factors such as age and education also influenced adoption. For example, older adults are particularly disadvantaged by fears, security concerns and physical limitations [42,165]. The COVID-19 pandemic accelerated DHT adoption but also widened inequalities [4,8,164]. National reimbursement initiatives such as Germany’s DiGA programme suggest that policy mechanisms may mitigate financial barriers [163]. These findings highlight infrastructural and financial determinants that are not consistently operationalised in existing technology adoption models, offering additional perspectives on equity and affordability in shaping adoption [56–61]. Here, environmental and policy-related factors function as foundational enabling conditions, while cultural and social positioning shape who can realistically benefit from DHTs.

Usability, engagement, and empowerment extended beyond the “ease of use” construct emphasised in TAM and UTAUT. Ease of use remains central, with additional elements such as personalised feedback, gamification and reminders that align with daily routines. Poorly designed notifications undermined adherence, with evidence showing app abandonment within 30–90 days [166]. Reinforcement mechanisms (gamification and feedback) and demographic moderators (age and gender) also affected engagement [70,167]. Therefore, adoption appears to be both intention-driven and habit-driven. This highlights the central psychological role of motivation, self-efficacy and habit formation in DHT adoption [32]. Constructs such as self-efficacy and motivation appear in HBM and SCT, while habit formation, reinforcement and user engagement are variably represented and may complement existing technology adoption frameworks.

Trust, privacy, and governance were multi-layered in nature. Confidence was built upon accurate healthcare data, reliable system performance, transparent data ownership and strong privacy protections. The introduction of AI-enabled tools added concerns over transparency, explainability, fairness and accountability [168]. Prior studies demonstrated that privacy risks reduce willingness to adopt [67], and trust mediates the relationship between usefulness and intention [72,169]. In AI-enabled DHTs, privacy concerns extend beyond data security to include secondary data use, algorithmic inference and opacity in automated decision-making [170,171]. Global governance frameworks such as the World Health Organisation (WHO) Global Strategy on Digital Health emphasise the role of regulatory maturity in enhancing legitimacy and public trust [7,163]. Implementation and enforcement of privacy protections vary substantially across regions [43,171]. As a result, mechanisms that enhance trust and data protection in one context may not be directly transferable to another. This review shows that trust operates at multiple levels: information, systems, institutions and algorithms. While trust is often treated in models as a single perceptual construct, the findings suggest that privacy and governance should be conceptualised as context-dependent, multi-layered determinants, highlighting the need for more granular conceptualisation within existing perspectives.

Integration, workforce, and sustainability were further decisive. Healthcare professionals acted as gatekeepers, with their digital literacy, workload, and liability concerns affecting the adoption of DHTs. Organisational readiness, leadership support and reimbursement influenced whether pilot programs were successfully implemented in routine practice. Interoperability challenges remain, particularly with electronic health records [4,65,172], while cost-effectiveness evidence is still limited, especially for elderly users [173]. These findings highlight that adoption is influenced not only by individual acceptance but also by organisational and systemic readiness. Although organisational and workforce factors are less frequently operationalised in existing models, their role is critical in determining successful adoption and implementation beyond pilot programmes [31,32]. This extends existing models, which primarily emphasise individual-level constructs, by highlighting workforce, organisational and system-level sustainability considerations.

Clinical effectiveness and quality of care also emerged as important determinants. Users and clinicians emphasised safety validation, certification and monitoring before trusting DHTs. Benefits such as improved monitoring and reduced travel promoted uptake, while risks of misdiagnosis and over-monitoring hindered it. Communication quality was shown to be as important as technical reliability, consistent with clinical validation [173] and emotional attitudes and fears [174]. These findings highlight that emotional and interpersonal dynamics influence acceptance and confidence, pointing to affective and relational qualities that are unevenly addressed in existing models but can serve as valuable extensions [56–61]. Health-related factors, including perceived clinical relevance, safety and quality of care, thus play a reinforcing role by legitimising DHT use within users’ health priorities and care expectations [31,32].

Taken together, the thematic findings demonstrate that DHT adoption is influenced by multiple factors that extend beyond individual perceptions and intentions. Psychological and social factors were consistently emphasised in the literature, which shape users’ motivation, engagement, trust and relational support. Health-related factors further reinforce DHT adoption by linking DHT use to perceived care quality, clinical relevance and health management needs. Environmental, organisational and cultural factors, which play a contextual and foundational role, influence access, equity, legitimacy and DHT adoption. However, these factors appear less consistently in existing technology adoption models, suggesting that such models emphasise individual perceptions and intentions over broader structural and contextual influences. Thus, although existing technology adoption models remain valuable, they can be further strengthened through more systematic integration of broader contextual and relational factors.

These findings have theoretical, practical, and policy implications for multiple stakeholder groups involved in DHT adoption. Theoretically, this review contributes to a broader multidisciplinary understanding of DHT adoption by integrating technological, psychological, social, cultural, health-related, and environmental perspectives. The findings also highlight the importance of considering contextual and system-level influences within existing frameworks.

Practically, the findings highlight several considerations for multidisciplinary stakeholders involved in DHT adoption. For patients and caregivers, factors such as usability, accessibility, trust, affordability, and fit with personal needs shape their DHT engagement. For healthcare providers and clinicians, workflow integration, trust, and communication quality are important in supporting adoption within clinical settings. For developers, the findings emphasise user-centred design, cultural relevance, accessibility, and real-world user needs and values. For healthcare administrators, organisational readiness, infrastructure capacity, and sustainability remain important considerations for large-scale implementation. For policymakers, broader system-level factors such as affordability, governance, infrastructure, and equity affect the successful implementation and equitable adoption of DHTs across different populations and healthcare settings.

Beyond stakeholder-specific implications, the findings also suggest several practical design considerations that emerged for the development and implementation of digital health technologies. DHTs need to incorporate multilingual and culturally appropriate interfaces, support low-bandwidth or offline functionality where appropriate, provide transparent and itemised cost information, deliver simple and actionable feedback, implement transparent privacy controls and explainable AI with appropriate human oversight, ensure interoperability with existing electronic health record systems, align with existing clinical workflows, and communicate evidence of clinical validation where available. Conversely, developers and implementers are encouraged to avoid notification overload, top-down design approaches that exclude end-user involvement, opaque automated decision-making, hidden costs, and one-size-fits-all solutions that fail to accommodate the needs of diverse user populations.

Table 6 summarises key DHT adoption factors and their practical implications across stakeholder groups.

**Table 6:** Key DHT adoption factors by stakeholder group.

Stakeholder group	Key influencing factors	Implications
Patients	Usability, health literacy, motivation, trust	Need simple, personalised, and accessible tools
Caregivers	Support burden, usability, communication	Require supportive and easy-to-use systems
Healthcare professionals	Workflow integration, workload, trust, training	Need efficient and integrated systems
Developers	Usability, interoperability, user-centred design	Develop user-centred DHTs that are accessible and compatible with healthcare workflows
Healthcare administrators	Cost, infrastructure, organisational readiness	Require sustainable and scalable solutions
Policymakers	Equity, access, regulation, governance	Need policies that support inclusive adoption
Researchers	Theory integration, contextual influences, longitudinal evaluation	Further refine and validate DHT engagement models across diverse settings

Collectively, these findings support the development of more equitable, user-centred and context-sensitive DHT implementation strategies across healthcare systems. The findings also provide valuable insights for national digital health strategies aimed at promoting inclusive, sustainable and patient-centred adoption. For example, policy frameworks such as Malaysia’s Ekonomi MADANI and national digital health roadmaps emphasise inclusivity, social wellbeing and digital transformation, which align with the multidimensional factors identified in this review. The findings may also inform broader digital health implementation efforts across different healthcare contexts. In addition, the findings highlight the importance of collaboration and education in supporting user-centred digital health development and implementation.

This review is strengthened by its multidisciplinary scope, protocol registration, and systematic review methodology, which supported a transparent and comprehensive synthesis of qualitative, quantitative and mixed-method evidence [74–77].

Nonetheless, several limitations must be acknowledged. Restricting the review to English, peer-reviewed studies from 2015 to 2025 may introduce language and publication bias. In addition, although the search strategy was designed to maximise conceptual relevance, it relied on a predefined set of adoption-related and theoretical keywords. As a result, some studies examining digital health use, engagement, implementation, or discontinuation without explicitly using adoption- or theory-oriented terminology may not have been captured.

The included studies varied in methodological clarity. Common limitations include small samples and cross-sectional designs using self-reported data, as identified through MMAT appraisal. As this review used thematic synthesis, pooled effect size estimation was not conducted, limiting quantitative comparison and causal inference across studies.

In addition, although this review has integrated evidence from various geographical regions, it did not conduct a jurisdiction-specific analysis of the data privacy regulations or artificial intelligence governance frameworks. Due to significant differences in legal protection, regulatory enforcement, cultural expectations regarding data usage and digital health infrastructure among various regions, the findings related to privacy and trust should be regarded as context-sensitive rather than universally generalisable [31,32]. Future research should address this gap by conducting comparative or jurisdiction-specific analyses to examine how regulatory and governance environments shape trust and adoption.

Although the review protocol was amended after registration, all amendments were documented in PROSPERO and are reflected in the final methodology. The review was subsequently conducted in accordance with the revised protocol. Furthermore, as most included studies employed cross-sectional designs, the findings primarily inform factors influencing digital health technology adoption rather than long-term use or sustained engagement. Future longitudinal studies are needed to examine how adoption factors evolve over time and across different stages of digital health technology implementation.

These limitations caution against generalising findings and highlight the need for methodologically robust future research, including longitudinal designs to examine adoption and disengagement over time, as well as intervention or quasi-experimental studies to evaluate how specific design features, policy interventions, or behaviour change strategies influence real-world adoption.

In conclusion, this review shows that adoption of DHTs is a multidimensional process driven not only by technological but also by psychological, social, cultural, health-related and environmental factors. Although existing adoption models provide valuable insights, broader contextual and system-level influences remain inconsistently represented. By integrating multidisciplinary evidence, this review presents a thematically grounded conceptual map that highlights the conceptual foundation and underrepresented determinants, providing a foundation for future model refinement and empirical validation. Future research should continue to empirically test, refine and validate these perspectives through longitudinal and intervention-based studies across diverse healthcare contexts to support sustainable, equitable and clinically meaningful digital health implementation.

## Supporting information

S1 Text PROSPERO registered protocol (CRD420251056883). (DOCX)

S1 File PRISMA Checklist. (DOCX)

S1 Table Records excluded at title/abstract and full text screening stages. (DOCX)

S2 Table Summary of extracted data from included studies. (DOCX)

S3 Table Details of quotations and codes. (DOCX)

S4 Table Overview of methodological appraisal of included studies by study design (MMAT). (DOCX)

S1 Appendix Thematic analysis matrix for digital health technology adoption factors. (DOCX)

S2 Appendix Item-level MMAT assessment of included studies. (DOCX)
