# Supplementary material for: Advancing foundational models in digital health technology adoption: A systematic literature review of multidisciplinary factors
Source: PLOS Digit Health. 2026 Sep 2;5(9):e0001690. doi: 10.1371/journal.pdig.0001690 (PMC13537594; doi:10.1371/journal.pdig.0001690)
Supplement: Supplementary file 2 [file pdig.0001690.s013.pdf]

Date: June 16, 2026

Review Report

Paper Title: Advancing Foundational Models in Digital Health Technology Adoption: A Systematic Literature Review of Multidisciplinary Factors

Reviewer: Dr. Nguyen Thi Phuong Lan

Reviewer's Comments：

The authors have adequately addressed all my earlier comments on a point-by-point basis. The manuscript has been substantially improved and strengthened through the revisions. In view of these improvements, I recommend the manuscript for publication in its present form.
