# Supplementary material for: Advancing foundational models in digital health technology adoption: A systematic literature review of multidisciplinary factors
Source: PLOS Digit Health. 2026 Sep 2;5(9):e0001690. doi: 10.1371/journal.pdig.0001690 (PMC13537594; doi:10.1371/journal.pdig.0001690)
Supplement: Supplementary file 3 [file pdig.0001690.s011.pdf]

Date: May 3, 2026

Review Report

Paper Tittle: Advancing foundational models in digital health technology adoption: A systematic literature review of multidisciplinary factors

Reviewer: Dr Nguyen Thi Phuong Lan

Reviewer's comments:

The authors aim to identify multidisciplinary factors influencing the adoption of digital health technologies (DHTs). Through a review of 82 studies examining technological, psychological, social, cultural, health-related, and environmental factors, five key themes were identified, highlighting adoption as multi-dimensional and supporting the need for an integrated framework to enhance equitable, scalable, and effective DHT implementation.

However, to enhance the rigor and overall quality of the paper, the following areas warrant further attention:

No.	Issue	Page Number
1.	**Background of the study:**<br><br>The claims advanced by the authors should be substantiated with empirical evidence, including relevant data, statistics, and credible sources.<br>The authors assert that the adoption of DHT is impeded by persistent design and usability challenges. However, the validity of this claim remains unclear unless it is supported with specific evidence. The authors should clearly identify the design and usability challenges, specifying the countries or regions concerned, as well as the relevant time periods. Furthermore, this assertion should be supported by empirical findings from multiple studies conducted across different countries, including Malaysia. Reliance on secondary sources such as systematic literature reviews (e.g., reference 14) or evidence limited to a single country (e.g., reference 16) is insufficient to substantiate such a broad claim.	90 - 08

2.	**Problem statement:**<br><br>The authors assert that there is no comprehensive framework for inclusive design in DHT; however, this claim is not supported by sufficient evidence. It would be beneficial for the authors to include a comparative table summarising prior studies and the specific frameworks they have adopted, thereby demonstrating the extent to which these frameworks have addressed inclusivity. Furthermore, the manuscript would benefit from a clearer justification for the inclusion of additional constructs—namely psychological, social, cultural, health-related, and environmental factors—within the proposed DHT model. Providing empirical or real-world evidence to support the relevance of these factors would significantly strengthen the study's problem statement and overall argument.	Page 131 – 139.
3.	**Methodology:**<br><br>The authors refer to "dynamic and multi-level DHT adoption," yet it is unclear how this concept is defined or how it is insufficiently addressed by existing technology adoption models. The manuscript would benefit from a more detailed elaboration of this concept, including a clear explanation of its dimensions and the specific limitations of current models in capturing these aspects.	140 – 142
4.	**Findings:**<br><br>The authors provide a detailed explanation of the study's methodology, including the study period and sampling selection criteria. However, a descriptive overview of the study sample is not presented. The manuscript states that the number of studies increased over time, reaching a peak in 2024, yet no supporting data, figures, or tables are provided to substantiate this claim. The authors are encouraged to include relevant descriptive statistics, such as the number of studies by year, country, and	379 – 393

	population of interest (e.g., patients, clinical workers, hospital management). Additionally, it would be beneficial to categorise the studies according to domain areas, including technological, psychological, social, cultural, health, and environmental dimensions.	
5.	**Research gap:**<br><br>The effectiveness of the presentation would be enhanced by incorporating tables and figures to summarise the study's findings. In its current form, the absence of sufficiently robust supporting evidence limits the clarity with which the research gaps in prior studies are articulated.	
6.	**Contribution:**<br><br>The contribution of the study is not clearly articulated. It is important to specify the intended beneficiaries of the research. Given that the technology acceptance model encompasses multiple user groups—such as patients, caregivers, and healthcare administrators/clinicians—the study would benefit from a more structured presentation of findings, categorised according to each user group's adoption context.	
