# Supplementary material for: Advancing foundational models in digital health technology adoption: A systematic literature review of multidisciplinary factors
Source: PLOS Digit Health. 2026 Sep 2;5(9):e0001690. doi: 10.1371/journal.pdig.0001690 (PMC13537594; doi:10.1371/journal.pdig.0001690)
Supplement: Supplementary file 3 [file pdig.0001690.s001.docx]

**Advancing Foundational Models in Digital Health Technology Adoption: A Systematic Review of Multidisciplinary Factors**

*Huey Yu Ng, Shamala Ramasamy, Thulasimani Munohsamy @ Munusamy, Seong Ting Chen, Asnina Anandan, Gunasekar Thangarasu*

**Citation** 1 change

Huey Yu Ng, Shamala Ramasamy, Thulasimani Munohsamy @ Munusamy, Seong Ting Chen, Asnina Anandan, Gunasekar Thangarasu. Advancing Foundational Models in Digital Health Technology Adoption: A Systematic Review of Multidisciplinary Factors. PROSPERO 2025 CRD420251056883. Available from

<https://www.crd.york.ac.uk/PROSPERO/view/CRD420251056883>.

REVIEW TITLE AND BASIC DETAILS

**Review title** 1 change

Advancing Foundational Models in Digital Health Technology Adoption: A Systematic Review of Multidisciplinary Factors

**Condition or domain being studied** 1 change

*Digital health technologies; Adoption*

Digital health technologies, including telemedicine, mHealth, eHealth, wearable devices and electronic health records, focus on adoption.

**Rationale for the review** 1 change

Digital health technologies (DHTs) such as telemedicine and mobile health apps have improved healthcare accessibility and efficiency. However, despite their technical maturity, real-world adoption remains uneven due to the complex interaction of technological, psychological, social, cultural, health-related and environmental factors. Existing technology adoption models, such as the technology acceptance model (TAM), unified theory of acceptance and use of technology (UTAUT) and health behaviour model (HBM), offer valuable theoretical foundations, but often address these influencers, for example, digital literacy, privacy concerns, cultural diversity, economic disparities and infrastructure limitations, in a fragmented manner. This review aims to synthesise evidence across disciplines to identify gaps and limitations in existing technology adoption models and inform future theoretical development.

**Review objectives** 1 change

To systematically synthesise and critically examine multidisciplinary factors influencing the adoption of digital health technology and identify conceptual gaps and limitations within existing technology adoption models.

**Keywords**

Digital health technology; Health technology adoption; Technology Acceptance Model; Healthcare equity; User-centered design

**Country**

Malaysia

ELIGIBILITY CRITERIA

**Population** 1 change

*Included*

Studies involving digital health technology (DHT) stakeholders, including users from diverse demographic groups, such as patients, caregivers, children, young adults and older adults; healthcare professionals, including clinicians, nurses and allied health staff; academic researchers; technology developers and IT personnel; healthcare administrators and managers; and government agency representatives, where digital health technologies are used or considered for use in healthcare contexts.

*Excluded*

Studies not involving human subjects or healthcare-related DHT.

**Intervention(s) or exposure(s)** 1 change

*Included*

*Digital health technologies*

- Studies focus on adopting of digital health technologies (DHTs) such as telemedicine, mobile health (mHealth), eHealth systems, wearable technologies and electronic health records.
- Studies examining determinants, constructs or measurement items influencing DHT adoption.
- Qualitative, quantitative and mixed-method studies contribute to understanding DHT adoption.

*Excluded*

- Studies not directly related to digital technologies in healthcare contexts.
- Studies that do not examine adoption-related factors of DHTs.

**Comparator(s) or control(s)**

This review does not have any comparators

**Study design** 1 change

Both randomized and nonrandomized study types will be included.

*Included*

- Peer-reviewed empirical studies.
- Published between 2015 and 2025 globally.
- Studies employing structured research designs with clearly reported data collection methods, analytical techniques and theoretical justification.
- Studies focusing on digital health technologies in healthcare contexts.

*Excluded*

- Non-peer-reviewed literature.
- Studies published before 2015.
- Studies lacking clear methodological or analytical descriptions.

**Context** 1 change

- Studies reporting technological, psychological, social, cultural, health-related and environmental factors influencing the adoption of digital health technologies (DHTs).
- Studies identifying patterns or determinants of DHT adoption.
- Studies proposing, applying or evaluating conceptual frameworks or models related to DHT adoption.

TIMELINE OF THE REVIEW

**Date of first submission to PROSPERO**

23 May 2025

**Review timeline**

Start date: 23 March 2025. End date: 31 July 2027.

**Date of registration in PROSPERO**

26 May 2025

AVAILABILITY OF FULL PROTOCOL

**Availability of full protocol** 1 change

A full protocol has been written and uploaded to PROSPERO. The protocol may be accessed through this link

<https://www.crd.york.ac.uk/PROSPEROFILES/ce3ccd5c2665a5d0ef563f2a247d5ffc.pdf>.

SEARCHING AND SCREENING

**Search for unpublished studies**

Only published studies will be sought.

**Main bibliographic databases that will be searched** 1 change

None of the major databases will be searched but other important databases will be, as described below.

*Other important or specialist databases that will be searched*

EBSCO Discovery Service, including Scopus, ACM Digital Library and MEDLINE

**Search language restrictions**

The review will only include studies published in English.

**Search date restrictions**

Databases will be searched for articles published from 1 January 2015 and before by 18 June 2025.

**Other methods of identifying studies**

No other methods will be used.

**Link to search strategy**

A full search strategy is available in the full protocol as described in the Availability of full protocol section

**Selection process**

Studies will be screened independently by at least two people (or person/machine combination) with a process to resolve differences.

**Other relevant information about searching and screening** 1 change

**Search Strategy:**

The search strategy focused on five domains: digital health technologies, adoption and usage behaviour, theoretical models, obstacles and barriers, and multidisciplinary influences, using Boolean operators, truncation, wildcards, and quotation marks in accordance with EBSCOhost guidelines.

**Additional Timing Concern (Clarification and Justification: 23 March 2025 to 26 May 2025):**

A pilot search was conducted during this period to test and refine search terms and improve specificity. Broader preliminary search strings yielded many records that were poorly aligned with the inclusion criteria; therefore, the strategy was refined and confirmed before the final search was conducted. Accordingly, a two-month interval occurred between the pilot search and protocol registration.

**Post-Registration Amendments (Clarification of Scope and Timing: 20 July 2025):**

Following PROSPERO registration, minor refinements were made to the search terms and search timeline, and clarifications were applied to the eligibility criteria. These amendments were implemented before full screening and synthesis.

**Temporal Inconsistencies (Clarification of Search Coverage vs Final Inclusion Period):**

Studies published prior to 2015 were excluded. Studies published between 2015 and 2019 were searched and screened at the full-text stage but excluded due to limited relevance to current digital health technologies and adoption contexts. The final synthesis, therefore, included studies published from 2020 onward. This reflects a distinction between the search coverage period and the final inclusion period.

DATA COLLECTION PROCESS

**Data extraction from published articles and reports**

Data will be extracted by one person (or a machine) and checked by at least one other person (or machine).

Authors will not be contacted for further information.

**Study risk of bias or quality assessment**

Risk of bias will be assessed using:

Covidence's built-in quality assessment and risk of bias forms are used and customized to accommodate qualitative, quantitative, and mixed-methods studies. While the default Cochrane RoB1 template is designed for randomized controlled trials, Covidence allows the creation of customized templates to assess the adequacy of study designs from other research types.

Data will be assessed by one person (or a machine) and checked by at least one other person (or machine).

Additional information will not be sought from study investigators if required information is unclear or unavailable in the study publications/reports.

**Reporting bias assessment** 1 change

Risk of bias due to missing results will be assessed during quality appraisal using the Mixed Methods Appraisal Tool (MMAT), with particular attention to reporting completeness, methodological transparency and the presence of clearly described outcomes across study components.

**Certainty assessment**

Certainty of findings will not be assessed

OUTCOMES TO BE ANALYSED

**Main outcomes** 1 change

Identification of technological, psychological, social, health-related, cultural and environmental factors influencing digital health technology (DHT) adoption.

**Additional outcomes** 1 change

Mapping conceptual gaps and limitations within existing technology adoption models.

PLANNED DATA SYNTHESIS

**Strategy for data synthesis** 1 change

A thematic analysis will be conducted to identify and categorise the multidisciplinary factors influencing the adoption of digital health technology. Coding will be iterative, with themes refined through discussion and peer debriefing. Findings will be synthesised to identify conceptual gaps and implications for model development.

CURRENT REVIEW STAGE

**Stage of the review at this submission** 1 change

| **Review stage** | **Started** | **Completed** |
| --- | --- | --- |
| Pilot work | ✓ | ✓ |
| Formal searching/study identification | ✓ | ✓ |
| Screening search results against inclusion criteria | ✓ | ✓ |
| Data extraction or receipt of IPD | ✓ |  |
| Risk of bias/quality assessment | ✓ |  |
| Data synthesis | ✓ |  |

**Review status**

The review is currently planned or ongoing.

**Publication of review results**

Results of the review will be published in English.

REVIEW AFFILIATION, FUNDING AND PEER REVIEW

**Review team members**

**Miss Huey Yu Ng** (review guarantor and contact) International Medical University. Malaysia.

No conflict of interest declared.

**Dr Shamala Ramasamy.** International Medical University. Malaysia.

No conflict of interest declared.

**Dr Thulasimani Munohsamy @ Munusamy.** International Medical University. Malaysia.

No conflict of interest declared.

**Dr Seong Ting Chen.** International Medical University. Malaysia.

No conflict of interest declared.

**Dr Asnina Anandan.** International Medical University. Malaysia.

No conflict of interest declared.

**Dr Gunasekar Thangarasu.** International Medical University. Malaysia.

No conflict of interest declared.

**Named contact**

**Miss Huey Yu Ng** (00000032308@student.imu.edu.my). International Medical University. Malaysia.

**Review affiliation**

International Medical University, Kuala Lumpur, Malaysia

**Funding source**

Review has no specific/external funding but is supported by guarantor/review team (non-commercial) institutions.

**Peer review** 1 change

The review protocol underwent external peer review as part of a manuscript submitted to PLOS Digital Health. Reviewer comments were received, and revisions are currently in progress.

ADDITIONAL INFORMATION

**Additional information** 1 change

This review adopts a global scope to synthesise multidisciplinary factors influencing digital health technology adoption across diverse healthcare contexts, with findings interpreted comparatively to identify theoretical gaps in existing technology adoption models. Preliminary pilot search activities were conducted before PROSPERO registration to refine the search strategy, while the final comprehensive search, screening, and data extraction were conducted following registration, with minor post-registration refinements applied before full synthesis.

**Review conflict of interest**

Declared individual interests are recorded under team member details.. No additional interests are recorded for this review.

**Medical Subject Headings**

Digital Health; Malaysia; Adoption; Systematic Review

**Revision note** 1 change

This revision clarifies the scope, search strategy, eligibility criteria, appraisal approach and timeline to improve alignment and methodological transparency. The protocol was updated to reflect refinements made prior to and following registration, and to clearly frame the review as a global synthesis of digital health technology adoption factors.

SIMILAR REVIEWS

**Check for similar records already in PROSPERO** 1 change

*PROSPERO identified a number of existing PROSPERO records that were similar to this one (last check made on 29 January 2026). These are shown below along with the reasons given by that the review team for the reviews being different and/or proceeding.*

- Digital Health Interventions for Strengthening Healthcare Delivery in Africa: A Systematic Review [published 14 March 2025] [CRD420251009629]. The review was judged not to be similar
- Technology Adoption Models and Theories in Health Information Systems Acceptance in Ethiopia: A Systematic Review [published 7 April 2024] [CRD42024529118]. The review was acknowledged as similar but the authors opted to continue because there are differences in intervention or comparator, the review looks at additional or different outcomes. The authors also noted: "Our review is more comprehensive and does not focus on a single country or digital health technology (DHT). It combines multidisciplinary elements like technological, psychological, social, cultural, health and environmental influences on the DHTs adoption and focuses on finding gaps in current models such as the technology acceptance model (TAM), unified theory of acceptance and use of technology (UTAUT), and health behaviour model (HBM)."
- Economic Evaluations of Digital Health Technologies: A Systematic Review of Methodological Approaches and Outcome Assessments [published 23 November 2024] [CRD42024613511]. The review was judged not to be similar

**PROSPERO version history** 1 change

- Version 3.0, published 05 Feb 2026
- Version 2.0, published 20 Jul 2025
- Version 1.0, published 26 May 2025

**Disclaimer**

The content of this record displays the information provided by the review team. PROSPERO does not peer review registration records or endorse their content.

PROSPERO accepts and posts the information provided in good faith; responsibility for record content rests with the review team. The guarantor for this record has affirmed that the information provided is truthful and that they understand that deliberate provision of inaccurate information may be construed as scientific misconduct.

PROSPERO does not accept any liability for the content provided in this record or for its use. Readers use the information provided in this record at their own risk.

Any enquiries about the record should be referred to the named review contact
