# Supplementary material for: Advancing foundational models in digital health technology adoption: A systematic literature review of multidisciplinary factors
Source: PLOS Digit Health. 2026 Sep 2;5(9):e0001690. doi: 10.1371/journal.pdig.0001690 (PMC13537594; doi:10.1371/journal.pdig.0001690)
Supplement: Supplementary file 5 [file pdig.0001690.s003.docx]

**S1 Table. Records excluded at title/abstract and full-text screening stages**

**Table 1. Records excluded at title and abstract screening stage**

| **Study ID** | **Citation** |
| --- | --- |
| 1 | Lee AT, Ramasamy RK, Subbarao A. Understanding psychosocial barriers to healthcare technology adoption: a review of TAM technology acceptance model and unified theory of acceptance and use of technology (UTAUT) frameworks. Healthcare. 2025;13(3):250. doi:10.3390/healthcare13030250. |
| 2 | Bai X, Yang L. Exploring the determinants of AIGC usage intention based on the extended AIDUA model: a multi-group structural equation modeling analysis. Front Psychol. 2025;16:1589318. doi:10.3389/fpsyg.2025.1589318. |
| 3 | Shang X, Liu Z, Gong C, Hu Z, Wu Y, Wang C. Knowledge mapping and evolution of research on older adults' technology acceptance: a bibliometric study from 2013 to 2023. Humanit Soc Sci Commun. 2024;11(1):21. doi:10.1057/s41599-024-03658-2. |
| 4 | Bao H, Lee EWJ. Examining theoretical frameworks and antecedents of health apps and wearables use: a scoping review. Health Commun. 2024;39(12):2671–2681. doi:10.1080/10410236.2023.2283655. |
| 5 | Livieri G, Mangina E, Protopapadakis ED, Panayiotou AG. The gaps and challenges in digital health technology use as perceived by patients: a scoping review and narrative meta-synthesis. Front Digit Health. 2025;7:1474956. doi:10.3389/fdgth.2025.1474956. |
| 6 | Hartono A, Roostika R, Muslichah I. Investigating factors influencing decision makers to adopt accreditation information system using multi-theory of technology acceptance model, resource dependence theory, and technology-organization-environment: evidence from Indonesian private universities. Int Rev Manag Mark. 2025;15(2):132–145. doi:10.32479/irmm.17931. |
| 7 | Liao YK, Liu YY, Wu WY, Chi HK, Huang KC, Vuong BH. Synthesizing comprehensive models for health behavior studies: connecting dots and revealing links on health enhancement through a meta-analysis approach. Front Psychol. 2025;16:1518834. doi:10.3389/fpsyg.2025.1518834. |
| 8 | Rathnayake AS, Nguyen TDHN, Ahn Y. Factors influencing AI chatbot adoption in government administration: a case study of Sri Lanka's digital government. Adm Sci. 2025;15(5):157. doi:10.3390/admsci15050157. |
| 9 | Agbeyangi AO, Lukose JM. Telemedicine adoption and prospects in Sub-Saharan Africa: a systematic review with a focus on South Africa, Kenya, and Nigeria. Healthcare. 2025;13(7):762. doi:10.3390/healthcare13070762. |
| 10 | He H, Raja Ghazilla RA, Abdul-Rashid SH. A systematic review of the usability of telemedicine interface design for older adults. Appl Sci. 2025;15(10):5458. doi:10.3390/app15105458. |
| 11 | Bertolazzi A, Quaglia V, Bongelli R. Barriers and facilitators to health technology adoption by older adults with chronic diseases: an integrative systematic review. BMC Public Health. 2024;24(1):17. doi:10.1186/s12889-024-18036-5. |
| 12 | Dangbut A, Watcharamaisakul F, Champahom T, Jomnonkwao S, Wisutwattanasak P, Phojaem T, et al. The impact of attitude on high-speed rail technology acceptance among elderly passengers in urban and rural areas: a multigroup SEM analysis. Infrastructures. 2024;9(10):174. doi:10.3390/infrastructures9100174. |
| 13 | Tohit NFM, Ya RM, Haque M. Bridging the gap: integrating food security into healthcare for healthier futures – a scoping review. Adv Hum Biol. 2025;15(2):177–199. doi:10.4103/aihb.aihb_22_25. |
| 14 | Qi Y, Mohamad E, Azlan AA, Zhang C. Utilization of artificial intelligence in clinical practice: a systematic review of China's experiences. Digit Health. 2025;11:20552076251343752. doi:10.1177/20552076251343752. |
| 15 | Mwogosi A, Mambile C. Applying PEST analysis to evaluate the adoption of electronic health records in Tanzanian healthcare: a scoping review. Digit Health. 2025;11:20552076251334029. doi:10.1177/20552076251334029. |
| 16 | Ahmed MM, Okesanya OJ, Olaleke NO, Adigun OA, Adebayo UO, Oso TA, et al. Integrating digital health innovations to achieve universal health coverage: promoting health outcomes and quality through global public health equity. Healthcare. 2025;13(9):1060. doi:10.3390/healthcare13091060. |
| 17 | Altman Ferreira PS. Managing operational resilience during the implementation of digital transformation in healthcare organisational practices. J Health Organ Manag. 2025;39(3):334–358. doi:10.1108/JHOM-04-2024-0155. |
| 18 | Binsar F, Mursitama TN, Hamsal M, Rahim RK. Does digital leadership impact the performance of lower-middle-class hospitals? Int J Innov Technol Manag. 2024;21(6):2450044. doi:10.1142/S0219877024500445. |
| 19 | Jarnda KV, Dai H, Ali A, Bestman PL, Trafialek J, Roberts-Jarnda GP, et al. A review on optical biosensors for monitoring of uric acid and blood glucose using portable POCT devices: status, challenges, and future horizons. Biosensors. 2025;15(4):222. doi:10.3390/bios15040222. |
| 20 | Cheng X, Qiao L, Yang B, Han R. To trust or not to trust: a qualitative study of older adults' online communication during the COVID-19 pandemic. Electron Commer Res. 2025;25(2):849–878. doi:10.1007/s10660-023-09679-4. |
| 21 | Bakker JP, Barge R, Centra J, Cobb B, Cota C, Guo CC, et al. V3+ extends the V3 framework to ensure user-centricity and scalability of sensor-based digital health technologies. NPJ Digit Med. 2025;8(1):13. doi:10.1038/s41746-024-01322-2. |
| 22 | Kapse S, Yenurkar G, Nyangaresi VO, Balpande G, Kale S, Jadhav M, et al. MediServe: an IoT-enhanced deep learning framework for personalized medication management for elderly care. Comput Mater Continua. 2025;83(1):935–976. doi:10.32604/cmc.2025.061981. |
| 23 | Chu LY, Zhang Y, Smith GD, Ho KHM. Interprofessional collaborations on interventions for people with loneliness: a scoping review. Nurs Open. 2025;12(5):e70239. doi:10.1002/nop2.70239. |
| 24 | Tang J, Jia J, Gao Y, Yu W, Meng ZN, Zhu H, et al. The influence of patient self-efficacy on value co-creation behavior and outcomes in chronic disease management: a cross-sectional study. BMC Public Health. 2025;25(1):18. doi:10.1186/s12889-025-22917-8. |
| 25 | Pouwer F, Perrin B, Lavender A, Najafi B, Ismail K, Vileikyte L. The quest for wellness: how to optimise self-care strategies for diabetic foot management? Diabetes Metab Res Rev. 2024;40(3):e3751. doi:10.1002/dmrr.3751. |
| 26 | Sun C, Meijer E, Chavannes NH, Dai H, Li X, Wang Y, et al. eHealth literacy in the general population: a cross-sectional study in China. BMC Public Health. 2025;25(1):14. doi:10.1186/s12889-025-21389-0. |
| 27 | Mengistu TS, Endalamaw A, Zewdie A, Wolka E, Assefa Y. Strengthening primary health care in Ethiopia: a scoping review of successes, challenges, and pathways towards universal health coverage using the WHO monitoring framework. PLoS Glob Public Health. 2025;5(4):e0004470. doi:10.1371/journal.pgph.0004470. |
| 28 | Zhang Z, Xuan Z, Fu Y, Zhao H, Zhan P, Yang C, et al. Knowledge, attitudes, and practices regarding hypertension diet among patients with hypertension. Sci Rep. 2025;15(1):13. doi:10.1038/s41598-025-97016-0. |
| 29 | Shao Y, Yang X, Chen Q, Guo H, Duan X, Xu X, et al. Determinants of digital health literacy among older adult patients with chronic diseases: a qualitative study. Front Public Health. 2025;13:1568043. doi:10.3389/fpubh.2025.1568043. |
| 30 | Zhang R, Wang H. Insights into the technological evolution and research trends of mobile health: bibliometric analysis. Healthcare. 2025;13(7):740. doi:10.3390/healthcare13070740. |
| 31 | Bao H, Wong YJ, Singh NB, Pai SGS, Pham BTP, Theng YL, et al. Data absenteeism in digital health technology research for older adults: a systematic review. BMC Digit Health. 2025;3(1):13. doi:10.1186/s44247-025-00152-w. |
| 32 | Yogarajah J, Prybutok V, Prybutok G. Transdisciplinary issues of the United States healthcare delivery system. Informing Sci. 2024;27:1–28. doi:10.28945/5376. |
| 33 | Alum EU. Optimizing patient education for sustainable self-management in type 2 diabetes. Discover Public Health. 2025;22(1):5. doi:10.1186/s12982-025-00445-5. |
| 34 | Ban S, Kim Y, Seomun G. Digital health literacy: a concept analysis. Digit Health. 2024;10:20552076241287894. doi:10.1177/20552076241287894. |
| 35 | Kushniruk A, Kaufman D. Human factors and organizational issues in health informatics: review of recent developments and advances. Yearb Med Inform. 2024;33(1):196–209. doi:10.1055/s-0044-1800744. |
| 36 | Amrutia P, Anjum S, Mehta J. Women entrepreneurship in developing countries: a bibliometric analysis. Lib Progress Lib Sci Inf Technol Comput. 2024;44(3):13260–13278. |
| 37 | Chakraborty I, Ilavarasan PV, Edirippulige S. What helps age-tech startups scale-up? Some insights. Int Entrep Manag J. 2025;21(1):32. doi:10.1007/s11365-025-01081-w. |
| 38 | Ho TQA, Le LKD, Engel L, Le N, Melvin G, Le HND, et al. Barriers to and facilitators of user engagement with web-based mental health interventions in young people: a systematic review. Eur Child Adolesc Psychiatry. 2025;34(1):83–100. doi:10.1007/s00787-024-02386-x. |
| 39 | Kunuratna S, Boonyagul S, Thongpance N, Mekkaew C, Chotikunnan P, Wongkamhang A. Innovative health mattress acceptance: using technology acceptance model. Pak J Life Soc Sci. 2024;22(1):6302–6325. doi:10.57239/PJLSS-2024-22.1.00465. |
| 40 | Lamovšek A, Uršič D. Generational perspective of digital technologies at work: a topic modelling approach. Društvena Istraživanja. 2025;34(1):21–42. doi:10.5559/di.34.1.02. |
| 41 | van Genugten CR, Thong MSY, van Ballegooijen W, Kleiboer AM, Spruijt-Metz D, Smit AC, et al. Beyond the current state of just-in-time adaptive interventions in mental health: a qualitative systematic review. Front Digit Health. 2025;7:1460167. doi:10.3389/fdgth.2025.1460167. |
| 42 | Mishiba T. Transforming occupational health and safety regulation: strategic pathways in the era of Industry 4.0. J Work Health Saf Regul. 2024;3(2):150–168. doi:10.57523/jaohlev.pp.24-016. |
| 43 | Gkintoni E, Vassilopoulos SP, Nikolaou G. Next-generation cognitive-behavioral therapy for depression: integrating digital tools, teletherapy, and personalization for enhanced mental health outcomes. Medicina. 2025;61(3):431. doi:10.3390/medicina61030431. |
| 44 | Wynn M, Garwood-Cross L, Vasilica C, Griffiths M, Heaslip V, Phillips N. Digitizing nursing: a theoretical and holistic exploration to understand the adoption and use of digital technologies by nurses. J Adv Nurs. 2023;79(10):3737–3747. doi:10.1111/jan.15810. |
| 45 | Andarwati M, Swalaganata G, Yuniarti S, Pamuji FY, Sitompul ER, Yudhistiro K, et al. Adoption of generative AI-enhanced profit sharing digital systems in MSMEs: a comprehensive model analysis. Int J Adv Comput Sci Appl. 2025;16(1):412–422. doi:10.14569/ijacsa.2025.0160141. |
| 46 | Zhang M. Older people's attitudes towards emerging technologies: a systematic literature review. Public Underst Sci. 2023;32(8):948–968. doi:10.1177/09636625231171677. |
| 47 | Zeng Y, Fu J, Cao W, Chen Y, Yang Z. Research hotspots and knowledge framework of digital healthcare service management from the perspective of the silver economy. Sustainability. 2024;16(22):9735. doi:10.3390/su16229735. |
| 48 | Zhang B. Research progress and intellectual structure of design for digital equity (DDE): a bibliometric analysis based on CiteSpace. Humanit Soc Sci Commun. 2024;11(1):18. doi:10.1057/s41599-024-03552-x. |
| 49 | Zheng K, Han F, Yang S, Li N. Building trust in long-term care settings using assistive technology: a systematic review. Front Rehabil Sci. 2024;5:1492104. doi:10.3389/fresc.2024.1492104. |
| 50 | Catapan SDC, Sazon H, Zheng S, Gallegos-Rejas V, Mendis R, Santiago PHR, et al. A systematic review of consumers' and healthcare professionals' trust in digital healthcare. NPJ Digit Med. 2025;8(1):9. doi:10.1038/s41746-025-01510-8. |
| 51 | Colombo B. Strategic leadership and innovation adoption: a cross-cultural analysis of SMEs in Italy. Eur J Bus Strateg Manag. 2024;9(1):46–57. doi:10.47604/ejbsm.2461. |
| 52 | Kyei-Arthur F. Prevalence and predictors of internet use among Ghanaian older adults: evidence from the Ghana 2021 population and housing census. Humanit Soc Sci Commun. 2024;11(1):9. doi:10.1057/s41599-024-03788-7. |
| 53 | Almeida F. Contributions of municipal initiatives to digital health equity. World. 2024;5(4):1165–1180. doi:10.3390/world5040059. |
| 54 | Caloudas AB, Frosio KE, Torous J, Goss CW, Novins DK, Lindsay JA, et al. Mobile mental health applications for American Indian and Alaska Native communities: review and recommendations. J Technol Behav Sci. 2024;9(3):474–485. doi:10.1007/s41347-023-00348-9. |
| 55 | Belhadi A, Kamble S, Subramanian N, Singh RK, Venkatesh M. Digital capabilities to manage agri-food supply chain uncertainties and build supply chain resilience during compounding geopolitical disruptions. Int J Oper Prod Manag. 2024;44(11):1946–1982. doi:10.1108/IJOPM-11-2022-0737. |
| 56 | Gjylbegaj V, Mousa SA. Netflix usage among youth in the UAE: habits, needs, perceptions and expectations. J Digit Media Policy. 2025;16(2):131–150. doi:10.1386/jdmp_00174_1. |
| 57 | International Journal of Integrated Care. 23rd International Conference on Integrated Care, Antwerp, Flanders, 22–24 May 2023. Int J Integr Care. 2023;23(Suppl):703–704. |
| 58 | Takano E, Maruyama H, Takahashi T, Mori K, Nishiyori K, Morita Y, et al. User experience of older people while using digital health technologies: a systematic review. Appl Sci. 2023;13(23):12815. doi:10.3390/app132312815. |
| 59 | Sharma P, Rao S, Krishna Kumar P, Nair AR, Agrawal D, Zadey S, et al. Barriers and facilitators for the use of telehealth by healthcare providers in India: a systematic review. PLoS Digit Health. 2024;3(12):e0000398. doi:10.1371/journal.pdig.0000398. |
| 60 | Allioui H, Allioui A, Mourdi Y. Navigating transformation: unveiling the synergy of IoT, multimedia trends, and AI for sustainable financial growth in African context. Multimedia Tools Appl. 2025;84(9):6473–6517. doi:10.1007/s11042-024-19038-6. |
| 61 | Janes G, Chesterton L, Heaslip V, Reid J, Lüdemann B, Gentil J, et al. Current nursing and midwifery contribution to leading digital health policy and practice: an integrative review. J Adv Nurs. 2025;81(1):116–139. doi:10.1111/jan.16265. |
| 62 | Yu S. A comparative study on the psychological audience acceptance of young people through local government public management combined with media: an exploration from a cross-cultural perspective. Lex Localis. 2025;23(1):186–209. doi:10.52152/23.1.186-209(2025). |
| 63 | Jutai JW, Hatoum F, Bhardwaj D, Hosseini M. Implementation of digital health technologies for older adults: a scoping review. Front Aging. 2024;5:1349520. doi:10.3389/fragi.2024.1349520. |
| 64 | Seakamela KP, Mashaba RG, Ntimana CB, Kabudula CW, Sodi T. Multimorbidity management: a scoping review of interventions and health outcomes. Int J Environ Res Public Health. 2025;22(5):770. doi:10.3390/ijerph22050770. |
| 65 | European Heart Journal. European Heart Journal 2023;44(Suppl 2). Eur Heart J. 2023;44(Suppl 2). |
| 66 | LoBuono DL, Milovich M Jr. A scoping review of nutrition health for older adults: does technology help? Nutrients. 2023;15(20):4402. doi:10.3390/nu15204402. |
| 67 | Mabil-Atem JM, Gumuskaya O, Wilson RL. Digital mental health interventions for the mental health care of refugees and asylum seekers: integrative literature review. Int J Ment Health Nurs. 2024;33(4):760–780. doi:10.1111/inm.13283. |
| 68 | Adel A, Alani NHS. Human-centric collaboration and Industry 5.0 framework in smart cities and communities: fostering sustainable development goals 3, 4, 9, and 11 in Society 5.0. Smart Cities. 2024;7(4):1723–1775. doi:10.3390/smartcities7040068. |
| 69 | He H, Abdul-Rashid SH, Raja Ghazilla RA. Research trends and hot spots in telemedicine for the elderly: a scientometric analysis. Healthcare. 2024;12(18):1853. doi:10.3390/healthcare12181853. |
| 70 | Ognjanović I, Zoulias E, Mantas J. Progress achieved, landmarks, and future concerns in biomedical and health informatics. Healthcare. 2024;12(20):2041. doi:10.3390/healthcare12202041. |
| 71 | Thuesen J, Nilsson C, Caserotti P. Abstracts from the 26th Nordic Congress of Gerontology. J Ageing Longev. 2023;3(3):203–432. doi:10.3390/jal3030016. |
| 72 | Journal of Psychosocial Oncology Research and Practice. 24th IPOS World Congress of Psycho-Oncology, Milan, Italy: 1–3 September 2023. J Psychosoc Oncol Res Pract. 2023;5(Suppl):i–202. |
| 73 | Kanwel S, Ma Z, Li M, Hussain A, Erum N, Ahmad S. The influence of hospital services on patient satisfaction in OPDs: evidence from the transition to a digital system in South Punjab, Pakistan. Health Res Policy Syst. 2024;22(1):18. doi:10.1186/s12961-024-01178-8. |
| 74 | Moretti F, Mazzi MA, Montresor S, Colpo S, Tocco Tussardi I, Facchinello D, et al. Proximity care pathways and digitalization: opportunities and concerns for medication safety management—insights from the ProSafe study on community perspectives. Front Public Health. 2025;13:1486814. doi:10.3389/fpubh.2025.1486814. |
| 75 | Maawati F, Iswanti DI, Saifudin IMM, Dedi B. Nurses' perception towards electronic medical records system: an integrative review of barriers and facilitators. Iran J Public Health. 2025;54(1):62–73. |
| 76 | Bekbolatova M, Mayer J, Ong CW, Toma M. Transformative potential of AI in healthcare: definitions, applications, and navigating the ethical landscape and public perspectives. Healthcare. 2024;12(2):125. doi:10.3390/healthcare12020125. |
| 77 | Krahe MA, Larkins SL, Adams N. Digital health implementation in Australia: a scientometric review of the research. Digit Health. 2024;10:20552076241297729. doi:10.1177/20552076241297729. |
| 78 | Rodrigues DA, Roque M, Mateos-Campos R, Figueiras A, Herdeiro MT, Roque F. Barriers and facilitators of health professionals in adopting digital health-related tools for medication appropriateness: a systematic review. Digit Health. 2024;10:20552076231225133. doi:10.1177/20552076231225133. |
| 79 | Battaglia E, Poletti V, Compalati E, Azzollini M, Volpato E. Unmasking the complex interplay of obesity hypoventilation syndrome, heart failure, and sleep dysfunction: a physiological and psychological perspective in a digital health world. Behav Sci. 2025;15(3):285. doi:10.3390/bs15030285. |
| 80 | Garg SK, Renner D, Rewers A. Virtual clinics for diabetes care. Diabetes Technol Ther. 2025;27(Suppl):S2–S13. doi:10.1089/dia.2025.8801.skg. |
| 81 | Bobrowicz-Campos E, Camilo C, Pinheiro GG. Protocol: attitudinal factors related to the use of digital technologies in health by older adults: an overview of reviews. Campbell Syst Rev. 2025;21(1):e70022. doi:10.1002/cl2.70022. |
| 82 | Reidy C, Papoutsi C, KC S, Gudgin B, Laverty AA, Greaves F, et al. Qualitative evaluation of the implementation and national roll-out of the NHS App in England. BMC Med. 2025;23(1):18. doi:10.1186/s12916-024-03842-w. |
| 83 | Gilboa Y, Pagis M, Lyons K. Self-determination theory as a lens to explore the implementation challenges of telehealth. Int J Telerehabil. 2024;16(2):1–5. doi:10.5195/ijt.2024.6648. |
| 84 | Borges do Nascimento IJ, Abdulazeem H, Vasanthan LT, Martinez EZ, Zucoloto ML, Østengaard L, et al. Barriers and facilitators to utilizing digital health technologies by healthcare professionals. NPJ Digit Med. 2023;6(1):28. doi:10.1038/s41746-023-00899-4. |
| 85 | Alruwaili MM, Shaban M, Elsayed Ramadan O. Digital health interventions for promoting healthy aging: a systematic review of adoption patterns, efficacy, and user experience. Sustainability. 2023;15(23):16503. doi:10.3390/su152316503. |
| 86 | Cecconi C, Adams R, Cardone A, Declaye J, Silva M, Vanlerberghe T, et al. Generational differences in healthcare: the role of technology in the path forward. Front Public Health. 2025;13:1546317. doi:10.3389/fpubh.2025.1546317. |
| 87 | Rahman TU, Jan Z, Alam A, Ali R. Smartphone use and personality: their effects on sleep quality across groups using mediation analysis. Digit Health. 2024;10:20552076241295797. doi:10.1177/20552076241295797. |
| 88 | Seo YW, La Marca V, Tandon A, Chiao JC, Drummond CK. Exploring the design for wearability of wearable devices: a scoping review. Computers. 2024;13(12):326. doi:10.3390/computers13120326. |
| 89 | Sackey E, Ofori-Atta A, Ohene S, Obeng K, Ben-Zeev D. mHealth and digital innovations as catalysts for transforming mental health care in Ghana. Glob Health Sci Pract. 2024;12(6):e00062. doi:10.9745/GHSP-D-24-00062. |
| 90 | Alam P, Bolio A, Lin L, Larson HJ. Stakeholders' perceptions of personal health data sharing: a scoping review. PLoS Digit Health. 2024;3(11):e0000652. doi:10.1371/journal.pdig.0000652. |
| 91 | Alanazi MA, Al-Zughaibi SBM, Al-Harbi FBS, Al Dhaheri MAB. Digital health integration in radiological screening practices: opportunities and challenges for technicians in Saudi Vision 2030. J Int Crisis Risk Commun Res. 2024;7(Suppl):2499–2512. |
| 92 | Ramalho A, Paulo R, Duarte-Mendes P, Serrano J, Petrica J. Age unplugged: a brief narrative review on the intersection of digital tools, sedentary and physical activity behaviors in community-dwelling older adults. Healthcare. 2024;12(9):935. doi:10.3390/healthcare12090935. |
| 93 | International Journal of Pharmacy Practice. Health services research and pharmacy practice conference abstracts. Int J Pharm Pract. 2024;32(Suppl):i1–i61. |
| 94 | Ramasawmy M, Poole L, Thorlu-Bangura Z, Chauhan A, Murali M, Jagpal P, et al. Frameworks for implementation, uptake, and use of cardiometabolic disease-related digital health interventions in ethnic minority populations: scoping review. JMIR Cardio. 2022;6(2):e37360. doi:10.2196/37360. |
| 95 | Piper L, de Cosmo LM, Benvenuto M, Viola C. How do the determinants of collaborative consumption influence its use in healthcare? A managerial perspective. Int J Health Policy Manag. 2024;13(1):16. doi:10.34172/ijhpm.8453. |
| 96 | Hui CY, Abdulla A, Ahmed Z, Goel H, Habib GMM, Toh TH, et al. Mapping national information and communication technology (ICT) infrastructure to the requirements of potential digital health interventions in low- and middle-income countries. J Glob Health. 2022;12:04094. doi:10.7189/jogh.12.04094. |
| 97 | Basulo-Ribeiro J, Teixeira L. The future of healthcare with Industry 5.0: preliminary interview-based qualitative analysis. Future Internet. 2024;16(3):68. doi:10.3390/fi16030068. |
| 98 | Yu X, Wang Y, Liu Z, Jung E. Technological functionality and system architecture of mobile health interventions for diabetes management: a systematic review and meta-analysis of randomized controlled trials. Front Public Health. 2025;13:1549568. doi:10.3389/fpubh.2025.1549568. |
| 99 | Stephens JH, Northcott C, Poirier BF, Lewis T. Consumer opinion on the use of machine learning in healthcare settings: a qualitative systematic review. Digit Health. 2025;11:20552076241288631. doi:10.1177/20552076241288631. |
| 100 | Annals of Behavioral Medicine. 2021 ABM annual meeting abstracts supplement. Ann Behav Med. 2021;55(Suppl):S1–S618. doi:10.1093/abm/kaab020. |
| 101 | Pavia G, Branda F, Ciccozzi A, Romano C, Locci C, Azzena I, et al. Integrating digital health solutions with immunization strategies: improving immunization coverage and monitoring in the post-COVID-19 era. Vaccines. 2024;12(8):847. doi:10.3390/vaccines12080847. |
| 102 | Abouzeid N, Lal S. The role of sociodemographic factors on the acceptability of digital mental health care: a scoping review protocol. PLoS One. 2024;19(4):e0301886. doi:10.1371/journal.pone.0301886. |
| 103 | Aslan A, Mold F, van Marwijk H, Armes J. What are the determinants of older people adopting communicative e-health services: a meta-ethnography. BMC Health Serv Res. 2024;24(1):16. doi:10.1186/s12913-023-10372-3. |
| 104 | Christopoulou SC. Machine learning models and technologies for evidence-based telehealth and smart care: a review. BioMedInformatics. 2024;4(1):754–779. doi:10.3390/biomedinformatics4010042. |
| 105 | Bressan T, Valdivia-Gago A, Silvera-Ccallo RM, Llanos-Cuentas A, Condor DF, Padilla-Huamantinco PG, et al. Challenges of design, implementation, acceptability, and potential for biomedical technologies in the Peruvian Amazon. Int J Equity Health. 2022;21(1):19. doi:10.1186/s12939-022-01773-7. |
| 106 | Diabetes Technology & Therapeutics. The official journal of ATTD advanced technologies & treatments for diabetes conference, 6–9 March 2024, Florence & online. Diabetes Technol Ther. 2024;26(Suppl):A1–A376. doi:10.1089/dia.2024.2525.abstracts. |
| 107 | Rony MKK, Parvin MR, Wahiduzzaman M, Akter K, Ullah M. Challenges and advancements in the health-related quality of life of older people. Adv Public Health. 2024;2024:8839631. doi:10.1155/2024/8839631. |
| 108 | Breazu A. Digitization and innovation in healthcare services. Bus Excell Manag. 2024;14(3):25–39. doi:10.24818/beman/2024.14.3-02. |
| 109 | Goumas G, Dardavesis TI, Syrigos K, Syrigos N, Simou E. Chatbots in cancer applications, advantages and disadvantages: all that glitters is not gold. J Pers Med. 2024;14(8):877. doi:10.3390/jpm14080877. |
| 110 | Implementation Science. Proceedings of the 15th Annual Conference on the Science of Dissemination and Implementation in Health. Implement Sci. 2023;18(3):72. doi:10.1186/s13012-023-01299-8. |
| 111 | Abel G, Atherton H, Sussex J, Akter N, Aminu AQ, Bak W, et al. Current experience and future potential of facilitating access to digital NHS primary care services in England: the Di-Facto mixed-methods study. Health Soc Care Deliv Res. 2024;12(32–34):1–197. doi:10.3310/JKYT5803. |
| 112 | Jayousi S, Barchielli C, Alaimo M, Guarducci S, Caputo S, Paffetti M, et al. Health community 4.0: an innovative multidisciplinary solution for tailored healthcare assistance management. Sensors. 2024;24(18):6059. doi:10.3390/s24186059. |
| 113 | Khorram-Manesh A, Burkle FM Jr, Goniewicz K. Pandemics: past, present, and future: multitasking challenges in need of cross-disciplinary, transdisciplinary, and multidisciplinary collaborative solutions. Osong Public Health Res Perspect. 2024;15(4):267–285. doi:10.24171/j.phrp.2023.0372. |
| 114 | Czaja SJ, Charness N, Rogers WA, Sharit J, Moxley JH, Boot WR. The benefits of technology for engaging aging adults: findings from the PRISM 2.0 trial. Innov Aging. 2024;8(6):igae042. doi:10.1093/geroni/igae042. |
| 115 | Sussex J, Smith J, Wu FM. Service innovations for people with multiple long-term conditions: reflections of a rapid evaluation team. Health Soc Care Deliv Res. 2024;12(15–16):1–76. doi:10.3310/PTRU7108. |
| 116 | de Siqueira Silva Í, de Araújo AJ, Lopes RH, Silva CRDV, Xavier PB, de Figueirêdo RC, et al. Digital home care interventions and quality of primary care for older adults: a scoping review. BMC Geriatr. 2024;24(1):19. doi:10.1186/s12877-024-05120-z. |
| 117 | Wang M, Huang K, Li X, Zhao X, Downey L, Hassounah S, et al. Health workers' adoption of digital health technology in low- and middle-income countries: a systematic review and meta-analysis. Bull World Health Organ. 2025;103(2):126. doi:10.2471/BLT.24.292157. |
| 118 | Lwin HNN, Punnakitikashem P, Thananusak T. E-health research in Southeast Asia: a bibliometric review. Sustainability. 2023;15(3):2559. doi:10.3390/su15032559. |
| 119 | Teo JYC, Ramachandran HJ, Jiang Y, Seah CWA, Lim ST, Nguyen HD, et al. The characteristics and acceptance of technology-enabled diabetes prevention programs amongst individuals with prediabetes: a scoping review. J Clin Nurs. 2023;32(17–18):5562–5578. doi:10.1111/jocn.16649. |
| 120 | Annals of Behavioral Medicine. 2022 ABM annual meeting abstracts supplement. Ann Behav Med. 2022;56(Suppl):S1–S687. doi:10.1093/abm/kaac014. |
| 121 | Kumar P, Dwivedi YK, Anand A. Responsible artificial intelligence (AI) for value formation and market performance in healthcare: the mediating role of patient’s cognitive engagement. Inf Syst Front. 2023;25(6):2197–2220. doi:10.1007/s10796-021-10136-6. |
| 122 | Spoladore D, Mondellini M, Mahroo A, Chicchi-Giglioli IA, De Gaspari S, Di Lernia D, et al. Smart waiting room: a systematic literature review and a proposal. Electronics. 2024;13(2):388. doi:10.3390/electronics13020388. |
| 123 | Phuyal S, Elvas LB, Ferreira JC, Bista R. Harnessing wearable devices for enhanced long-term care: opportunities, challenges, and future directions. Int J Comput Inf Syst Ind Manag Appl. 2024;16(3):502–514. |
| 124 | Ghorbanian Zolbin M, Huvila I, Nikou S. Health literacy, health literacy interventions and decision-making: a systematic literature review. J Doc. 2022;78(7):405–428. doi:10.1108/JD-01-2022-0004. |
| 125 | Cajochen C. Thirty-fifth annual meeting of the Society for Light Treatment and Biological Rhythms (SLTBR), 20–22 June, Prague, Czech Republic. Clocks Sleep. 2024;6(4):690–748. doi:10.3390/clockssleep6040047. |
| 126 | Manyazewal T, Davey G, Hanlon C, Newport MJ, Hopkins M, Wilburn J, et al. Innovative technologies to address neglected tropical diseases in African settings with persistent sociopolitical instability. Nat Commun. 2024;15(1):5496. doi:10.1038/s41467-024-54496-4. |
| 127 | Selvam KP, Kosalram K, Chinnaiyan S. Post-COVID pandemic: the new normal and aftermath. J Fam Med Prim Care. 2024;13(10):4308–4314. doi:10.4103/jfmpc.jfmpc_313_24. |
| 128 | Jabade M, Kaur J, Barde S. Exploring digital health for effective geriatric care: a comprehensive overview. Top Geriatr Rehabil. 2025;41(2):114–120. doi:10.1097/TGR.0000000000000469. |
| 129 | Hong Kong Association of Gerontology. 30th annual congress of gerontology. Asian J Gerontol Geriatr. 2024;19(1):28–40. |
| 130 | Addotey-Delove M, Scott RE, Mars M. Healthcare workers’ perspectives of mHealth adoption factors in the developing world: scoping review. Int J Environ Res Public Health. 2023;20(2):1244. doi:10.3390/ijerph20021244. |
| 131 | Dave P. How digital health is revolutionizing healthcare and contributing to positive health outcomes. J Drug Deliv Ther. 2024;14(9):287–293. doi:10.22270/jddt.v14i6.6640. |
| 132 | Spoladore D, Colombo V, Fumagalli A, Tosi M, Lorenzini EC, Sacco M. An ontology-based decision support system for tailored clinical nutrition recommendations for patients with chronic obstructive pulmonary disease: development and acceptability study. JMIR Med Inform. 2024;12:e50980. doi:10.2196/50980. |
| 133 | Calegari LP, Tortorella GL, Fettermann DC. Getting connected to m-health technologies through a meta-analysis. Int J Environ Res Public Health. 2023;20(5):4369. doi:10.3390/ijerph20054369. |
| 134 | Njoku C, Green Hofer S, Sathyamoorthy G, Patel N, Potts HWW. The role of accelerator programmes in supporting the adoption of digital health technologies: a qualitative study of the perspectives of small- and medium-sized enterprises. Digit Health. 2023;9:20552076231173303. doi:10.1177/20552076231173303. |
| 135 | Ghafurian M, Chandra S, Hutchinson R, Lim A, Baliyan I, Rhim J, et al. Systematic review of social robots for health and wellbeing: a personal healthcare journey lens. ACM Trans Hum Robot Interact. 2025;14(1):48. doi:10.1145/3700446. |
| 136 | Ploegmakers KJ, Linn AJ, Medlock S, Seppälä LJ, Bahat G, Caballero-Mora MA, et al. A European survey of older peoples’ preferences, and perceived barriers and facilitators to inform development of a medication-related fall-prevention patient portal. Eur Geriatr Med. 2024;15(3):817–829. doi:10.1007/s41999-024-00951-w. |
| 137 | Ciasullo MV, Lim WM, Manesh MF, Palumbo R. The patient as a prosumer of healthcare: insights from a bibliometric-interpretive review. J Health Organ Manag. 2022;36(9):133–157. doi:10.1108/JHOM-11-2021-0401. |
| 138 | Alves da Mata Ribeiro J, de Freitas Cavalcante P, da Silva Batista RL, de Oliveira Tito Borba AK, Ribeiro Vasconcelos EM. Factors associated with digital literacy in the health of elderly people: an integrative review. Saúde Coletiva. 2025;15(93):14726–14732. doi:10.36489/saudecoletiva.2025v15i93p14726-14732. |
| 139 | Theodore Armand TP, Kim HC, Kim JI. Digital anti-aging healthcare: an overview of the applications of digital technologies in diet management. J Pers Med. 2024;14(3):254. doi:10.3390/jpm14030254. |
| 140 | Niu T, Lei B, Guo L, Fang S, Li Q, Gao B, et al. A review of optimization studies for system appointment scheduling. Axioms. 2024;13(1):16. doi:10.3390/axioms13010016. |
| 141 | Stoumpos AI, Kitsios F, Talias MA. Digital transformation in healthcare: technology acceptance and its applications. Int J Environ Res Public Health. 2023;20(4):3407. doi:10.3390/ijerph20043407. |
| 142 | Barnett DJ, Sundermeir SM, Reznar MM, Lightner A, Poirier L, Rosenblum AJ, et al. Protocol for the Support Application for Food PAntrieS trial: design, implementation, and evaluation plan for a digital application to promote healthy food access and support food pantry operations. Front Public Health. 2024;12:1340707. doi:10.3389/fpubh.2024.1340707. |
| 143 | Kaarakainen M-T, Saikkonen L. Remark on digital accessibility: educational disparities define digital inclusion from adolescence onwards. Univers Access Inf Soc. 2023;22(4):1279–1292. doi:10.1007/s10209-022-00908-5. |
| 144 | Bulaj G, Coleman M, Johansen B, Kraft S, Lam W, Phillips K, et al. Redesigning pharmacy to improve public health outcomes: expanding retail spaces for digital therapeutics to replace consumer products that increase mortality and morbidity risks. Pharmacy (Basel). 2024;12(4):107. doi:10.3390/pharmacy12040107. |
| 145 | Lawrence K, Levine DL. The digital determinants of health: a guide for competency development in digital care delivery for health professions trainees. JMIR Med Educ. 2024;10:e54173. doi:10.2196/54173. |
| 146 | Tomičić A, Malešević A, Čartolovni A. Ethical, legal and social issues of digital phenotyping as a future solution for present-day challenges: a scoping review. Sci Eng Ethics. 2022;28(1):25. doi:10.1007/s11948-021-00354-1. |
| 147 | Abhari S, Morita P, Souza Miranda PADS, Garavand A, Hanjahanja-Phiri T, Chumachenko D. Non-fungible tokens in healthcare: a scoping review. Front Public Health. 2023;11:1266385. doi:10.3389/fpubh.2023.1266385. |
| 148 | Conway A, Ryan A, Harkin D, McCauley C, Goode D. A review of the factors influencing adoption of digital health applications for people living with dementia. Digit Health. 2023;9:20552076231162985. doi:10.1177/20552076231162985. |
| 149 | Ndayishimiye C, Lopes H, Middleton J. A systematic scoping review of digital health technologies during COVID-19: a new normal in primary health care delivery. Health Technol. 2023;13(2):273–284. doi:10.1007/s12553-023-00725-7. |
| 150 | Manning JB, Blandford A, Edbrooke-Childs J. High school teachers’ experiences of consumer technologies for stress management during the COVID-19 pandemic: qualitative study. JMIR Form Res. 2023;7:e50460. doi:10.2196/50460. |
| 151 | Chilunjika SRT, Uwizeyimana DE. Blockchain technology for health information management: a case of Zimbabwe. Insights Reg Dev. 2024;6(1):59–73. doi:10.9770/ird.2024.6.1(5). |
| 152 | Al Kuwaiti A, Nazer K, Al-Reedy A, Al-Shehri S, Al-Muhanna A, Subbarayalu AV, et al. A review of the role of artificial intelligence in healthcare. J Pers Med. 2023;13(6):951. doi:10.3390/jpm13060951. |
| 153 | Maetzler W, Mirelman A, Pilotto A, Bhidayasiri R. Identifying subtle motor deficits before Parkinson’s disease is diagnosed: what to look for? J Parkinsons Dis. 2024;14(Suppl):S287–S296. doi:10.3233/JPD-230350. |
| 154 | Diabetes Technology & Therapeutics. The official journal of ATTD advanced technologies & treatments for diabetes conference, 22–25 February 2023, Berlin & online. Diabetes Technol Ther. 2023;25(Suppl):A1–A269. doi:10.1089/dia.2023.2525.abstracts. |
| 155 | Alhussein G, Hadjileontiadis L. Digital health technologies for long-term self-management of osteoporosis: systematic review and meta-analysis. JMIR Mhealth Uhealth. 2022;10(4):e32557. doi:10.2196/32557. |
| 156 | Liu PJ, Inman JJ, Li B, Wong CA, Yang N. Consumer health in the digital age. J Assoc Consum Res. 2022;7(2):198–209. doi:10.1086/718457. |
| 157 | Wang Q, Liu J, Zhou L, Tian J, Chen X, Zhang W, et al. Usability evaluation of mHealth apps for elderly individuals: a scoping review. BMC Med Inform Decis Mak. 2022;22(1):17. doi:10.1186/s12911-022-02064-5. |
| 158 | Tsetoura A. Technological inequality and social exclusion of older people during the COVID-19 pandemic. Int J Soc Qual. 2022;12(2):72–93. doi:10.3167/IJSQ.2022.120205. |
| 159 | Shaw L, Jazayeri D, Kiegaldie D, Morris ME. Implementation of virtual communities of practice in healthcare to improve capability and capacity: a 10-year scoping review. Int J Environ Res Public Health. 2022;19(13):7994. doi:10.3390/ijerph19137994. |
| 160 | Kokorelias KM, Nelson MLA, Tang T, Gray CS, Ellen M, Plett D, et al. Inclusion of older adults in digital health technologies to support hospital-to-home transitions: secondary analysis of a rapid review and equity-informed recommendations. JMIR Aging. 2022;5(2):e35925. doi:10.2196/35925. |
| 161 | Klossner S, Ghanbari H, Rossi M, Sarv L. Personalization–privacy paradox in using mobile health services. In: Proceedings of the European Conference on Information Systems (ECIS); 2023. p. 1–16. |
| 162 | Abstracts. Ann Behav Med. 2016;50(Suppl):1–335. doi:10.1007/s12160-015-9766-4. |
| 163 | Pegoraro V, Bidoli C, Dal Mas F, Bert F, Cobianchi L, Zantedeschi M, et al. Cardiology in a digital age: opportunities and challenges for e-health: a literature review. J Clin Med. 2023;12(13):4278. doi:10.3390/jcm12134278. |
| 164 | Lo C, Yu J, Görges M, Matava C, Veyckemans F. Anesthesia in the modern world of apps and technology: implications and impact on wellness. Pediatr Anesth. 2021;31(1):31–38. doi:10.1111/pan.14051. |
| 165 | Abstract booklet 2022. Int J Stroke. 2023;18(Suppl):3–124. doi:10.1177/17474930221142512. |
| 166 | Katusiime J, Tumuhimbise W, Rwambuka Mugyenyi G, Kobutungi P, Mugaba A, Zender R, et al. The role of mobile health technologies in promoting COVID-19 prevention: a narrative review of intervention effectiveness and adoption. Digit Health. 2022;8:20552076221131146. doi:10.1177/20552076221131146. |
| 167 | Ali SG, Zhang C, Guan Z, Chen T, Wu Q, Li P, et al. AI-enhanced digital technologies for myopia management: advancements, challenges, and future prospects. Vis Comput. 2024;40(6):3871–3887. doi:10.1007/s00371-024-03391-6. |
| 168 | Hui E, Singh S, Lin PKF, Dillon D. Social media influence on emerging adults’ prosocial behavior: a systematic review. Basic Appl Soc Psychol. 2024;46(4):239–265. doi:10.1080/01973533.2024.2342396. |
| 169 | Zhai S, Chu F, Tan M, Chi N-C, Ward T, Yuwen W. Digital health interventions to support family caregivers: an updated systematic review. Digit Health. 2023;9:20552076231171967. doi:10.1177/20552076231171967. |
| 170 | Mazzù MF, Baccelloni A, Romani S. Counteracting noncommunicable diseases with front-of-pack nutritional labels’ informativeness: an inquiry into the effects on food acceptance and portion selection. Br Food J. 2023;125(13):562–578. doi:10.1108/BFJ-04-2023-0285. |
| 171 | Vis C, Bührmann L, Riper H, Ossebaard HC. Health technology assessment frameworks for eHealth: a systematic review. Int J Technol Assess Health Care. 2020;36(3):204–216. doi:10.1017/S026646232000015X. |
| 172 | Ben-Zeev D, Larsen A, Beaulieu A, Guler J, Bawaneh A. Mental health priorities in the Arab world: insights from Jordan. Psychiatr Serv. 2024;75(6):604–607. doi:10.1176/appi.ps.20230365. |
| 173 | Wang T, Giunti G, Melles M, Goossens R. Design-relevant factors affecting the patient experience in digital health: preliminary results of an umbrella systematic review. Stud Health Technol Inform. 2021;290:862–866. doi:10.3233/SHTI220202. |
| 174 | Savage MO, Fernandez-Luque L, Graham S, van Dommelen P, Araujo M, de Arriba A, et al. Adherence to r-hGH therapy in pediatric growth hormone deficiency: current perspectives on how patient-generated data will transform r-hGH treatment towards integrated care. Patient Prefer Adherence. 2022;16:1663–1671. doi:10.2147/PPA.S271453. |
| 175 | Hurvitz N, Ilan Y. The constrained-disorder principle assists in overcoming significant challenges in digital health: moving from “nice to have” to mandatory systems. Clin Pract. 2023;13(4):994–1014. doi:10.3390/clinpract13040089. |
| 176 | Zhang W, Xiong K, Zhu C, Evans R, Zhou L, Podrini C. Promoting child and adolescent health through wearable technology: a systematic review. Digit Health. 2024;10:20552076241260507. doi:10.1177/20552076241260507. |
| 177 | Ramachandran M, Brinton C, Wiljer D, Upshur R, Gray CS. The impact of eHealth on relationships and trust in primary care: a review of reviews. BMC Prim Care. 2023;24(1):33. doi:10.1186/s12875-023-02176-5. |
| 178 | Wright PJ, Raynor PA, Bowers D, Combs EM, Corbett CF, Hardy H, et al. Leveraging digital technology for social connectedness among adults with chronic conditions: a systematic review. Digit Health. 2023;9:20552076231204746. doi:10.1177/20552076231204746. |
| 179 | Ramasawmy M, Khan N, Sunkersing D, Sajid M, Modha SH, Mistry M, et al. Recommendations for improving accessibility of digital health interventions for cardiometabolic disease for ethnically diverse populations. Digit Health. 2025;11:20552076241272600. doi:10.1177/20552076241272600. |
| 180 | Samir M. Record growth: over 294,000 Saudis join private sector in 2024. Leaders Magazine. 2025 Apr;100:46–57. |
| 181 | Hoe SL. Digital health in Southeast Asia: startups and digital technology applications. Asian J Innov Policy. 2022;11(2):183–201. doi:10.7545/ajip.2022.11.2.183. |
| 182 | Sagoo GS, Robinson T, Coughlan D, Meader N, Rice S, Vale L. Evaluating high-cost technologies – no need to throw the baby out with the bathwater. Expert Rev Pharmacoecon Outcomes Res. 2023;23(10):1177–1183. doi:10.1080/14737167.2023.2263647. |
| 183 | Moreno A, Nap HH, Helal S, Gutman GM. Editorial: Gerontechnologies for home support. Front Psychol. 2024;1–Apr:1477507. doi:10.3389/fpsyg.2024.1477507. |
| 184 | Sundermeir S, Martin N, Poirier L, Reznar M, Barnett D, Uriarte A, et al. Current use and demand for digital tools to enhance food pantry management: findings from a nationwide survey. J Hunger Environ Nutr. 2024;19(6):938–951. doi:10.1080/19320248.2023.2228728. |
| 185 | Abimbola S, Patel B, Peiris D, Patel A, Harris M, Usherwood T, et al. The NASSS framework for ex post theorisation of technology-supported change in healthcare: worked example of the TORPEDO programme. BMC Med. 2019;17(1):17. doi:10.1186/s12916-019-1463-x. |
| 186 | Zainal H, Xin X, Thumboo J, Yong FK. Exploring the views of Singapore junior doctors on medical curricula for the digital age: a case study. PLoS ONE. 2023;18(3):e0281108. doi:10.1371/journal.pone.0281108. |
| 187 | Daniore P, Nittas V, Gille F, von Wyl V. Promoting participation in remote digital health studies: an expert interview study. Digit Health. 2023;9:20552076231212063. doi:10.1177/20552076231212063. |
| 188 | Chung-Lee L, Catallo C. A new approach to digital health? Virtual COVID-19 care: a scoping review. Digit Health. 2023;9:20552076231152171. doi:10.1177/20552076231152171. |
| 189 | Wilson J, Heinsch M, Betts D, Booth D, Kay-Lambkin F. Barriers and facilitators to the use of e-health by older adults: a scoping review. BMC Public Health. 2021;21(1):11623. doi:10.1186/s12889-021-11623-w. |
| 190 | Bhatt P, Liu J, Gong Y, Wang J, Guo Y. Emerging artificial intelligence–empowered mHealth: scoping review. JMIR mHealth uHealth. 2022;10(6):e35053. doi:10.2196/35053. |
| 191 | Benge JF, Scullin MK. A meta-analysis of technology use and cognitive aging. Nat Hum Behav. 2025. doi:10.1038/s41562-025-02159-9. |
| 192 | Hussein Ofayr IS, Alyami AA, Al salem HA, Algamily SM, Aljameeli SM, Obed Alanazi FH, et al. The role of nursing and healthcare assistants in providing patient care using digital techniques. J Int Crisis Risk Commun Res. 2024;7(Suppl):861–868. |
| 193 | Wegener EK, Bergschöld JM, Whitmore C, Winters M, Kayser L. Involving older people with frailty or impairment in the design process of digital health technologies to enable aging in place: scoping review. JMIR Hum Factors. 2023;10:131–144. doi:10.2196/37785. |
| 194 | Gamberini C, Angeli F, Ambrosino E. Exploring solutions to improve antenatal care in resource-limited settings: an expert consultation. BMC Pregnancy Childbirth. 2022;22(1):4778. doi:10.1186/s12884-022-04778-w. |
| 195 | Singh H, Tang T, Gray CS, Kokorelias K, Thombs R, Plett D, et al. Recommendations for the design and delivery of transitions-focused digital health interventions: rapid review. JMIR Aging. 2022;5(2):e35929. doi:10.2196/35929. |
| 196 | Ruyobeza B, Grobbelaar SS, Botha A. Hurdles to developing and scaling remote patients' health management tools and systems: a scoping review. Syst Rev. 2022;11(1):23. doi:10.1186/s13643-022-02033-z. |
| 197 | Zawada SJ, Ganjizadeh A, Hagen CE, Demaerschalk BM, Erickson BJ. Feasibility of observing cerebrovascular disease phenotypes with smartphone monitoring: study design considerations for real-world studies. Sensors (Basel). 2024;24(11):3595. doi:10.3390/s24113595. |
| 198 | Tan S, Mills G. Designing Chinese hospital emergency departments to leverage artificial intelligence – a systematic literature review on the challenges and opportunities. Front Med Technol. 2024;Jan-15:1307625. doi:10.3389/fmedt.2024.1307625. |
| 199 | Baumann H, Heuel L, Bischoff LL, Wollesen B. mHealth interventions to reduce stress in healthcare workers (Fitcor): study protocol for a randomized controlled trial. Trials. 2023;24(1):7182. doi:10.1186/s13063-023-07182-7. |
| 200 | Bakker CJ, Wyatt TH, Breth MCS, Gao G, Janeway LM, Lee MA, et al. Nurses' roles in mHealth app development: scoping review. JMIR Nurs. 2023;6(1):e46058. doi:10.2196/46058. |
| 201 | Hochmuth A, Wrona KJ, Exner A-K, Dockweiler C. Digitization and health inequality and equity in nursing: a scoping review. Pflege. 2021;34(3):151–158. doi:10.1024/1012-5302/a000803. |
| 202 | Zhang Z, Xia E, Huang J. Impact of the moderating effect of national culture on adoption intention in wearable health care devices: meta-analysis. JMIR mHealth uHealth. 2022;10(6):e30960. doi:10.2196/30960. |
| 203 | SYMPOSIA ABSTRACTS. Can J Pain. 2018;2(1):A4–A45. doi:10.1080/24740527.2018.1476310. |
| 204 | Ilangakoon TS, Weerabahu SK, Samaranayake P, Wickramarachchi R. Adoption of Industry 4.0 and lean concepts in hospitals for healthcare operational performance improvement. Int J Prod Perform Manag. 2022;71(6):2188–2213. doi:10.1108/IJPPM-12-2020-0654. |
| 205 | Devi R, Kanitkar K, Narendhar R, Sehmi K, Subramaniam K. A narrative review of the patient journey through the lens of non-communicable diseases in low- and middle-income countries. Adv Ther. 2020;37(12):4808–4830. doi:10.1007/s12325-020-01519-3. |
| 206 | Fischer RP, Volpert A, Antonino P, Ahrens TD. Digital patient twins for personalized therapeutics and pharmaceutical manufacturing. Front Digit Health. 2024;1:1302338. doi:10.3389/fdgth.2023.1302338. |
| 207 | Vailati Riboni F, Comazzi B, Bercovitz K, Castelnuovo G, Molinari E, Pagnini F. Technologically-enhanced psychological interventions for older adults: a scoping review. BMC Geriatr. 2020;20(1):1594. doi:10.1186/s12877-020-01594-9. |
| 208 | Xu X, Yasmeen R, Shah WUH. Efficiency evaluation, regional technological heterogeneity and determinant of total factor productivity change in China's healthcare system. Sci Rep. 2024;14(1):70736. doi:10.1038/s41598-024-70736-5. |
| 209 | Alanazi SA, Alanazi AO, Alanazi HS, Alanazi TG, Almutiri MH, Alharbi AA, et al. Integrating digital technologies and artificial intelligence into electronic health records to enhance healthcare outcomes. J Int Crisis Risk Commun Res. 2024;7(Suppl):975–983. |
| 210 | Azizi Z, Adedinsewo D, Rodriguez F, Lewey J, Merchant RM, Brewer LC. Leveraging digital health to improve the cardiovascular health of women. Curr Cardiovasc Risk Rep. 2023;17(11):205–214. doi:10.1007/s12170-023-00728-z. |
| 211 | Torous J, Nicholas J, Larsen ME, Firth J, Christensen H. Clinical review of user engagement with mental health smartphone apps: evidence, theory and improvements. Evid Based Ment Health. 2018;21(3):116–119. |
| 212 | Abstracts of the MDS Virtual Congress 2021. Mov Disord. 2021;36(Suppl):S1–S599. doi:10.1002/mds.28794. |
| 213 | Cajamarca G, Proust V, Herskovic V, Cádiz RF, Verdezoto N, Fernández FJ. Technologies for managing the health of older adults with multiple chronic conditions: a systematic literature review. Healthcare (Basel). 2023;11(21):2897. doi:10.3390/healthcare11212897. |
| 214 | von Huben A, Howell M, Howard K, Carrello J, Norris S. Health technology assessment for digital technologies that manage chronic disease: a systematic review. Int J Technol Assess Health Care. 2021;37(1):e14. doi:10.1017/S0266462321000362. |
| 215 | Kraaij W, Verberne S, Koldijk S, de Korte E, van Dantzig S, Sappelli M, et al. Personalized support for well-being at work: an overview of the SWELL project. User Model User-Adap Interact. 2020;30(3):413–446. doi:10.1007/s11257-019-09238-3. |
| 216 | Masenya TM. Digital transformation of medical libraries: positioning and pioneering electronic health record systems in South Africa. Int J E-Health Med Commun (IJEHMC). 2024;15(1):1–13. doi:10.4018/IJEHMC.345402. |
| 217 | Choukou MA, Sanchez-Ramirez DC, Pol M, Uddin M, Monnin C, Syed-Abdul S. COVID-19 infodemic and digital health literacy in vulnerable populations: a scoping review. Digit Health. 2022;8:20552076221076927. doi:10.1177/20552076221076927. |
| 218 | Shah AM, Shah SV. Digital therapeutics—a new era in health care. Natl J Physiol Pharm Pharmacol. 2023;13(11):2190–2196. doi:10.5455/njppp.2023.13.09464202302102023. |
| 219 | Samal L, Fu HN, Camara DS, Wang J, Bierman AS, Dorr DA. Health information technology to improve care for people with multiple chronic conditions. Health Serv Res. 2021;56:1006–1036. doi:10.1111/1475-6773.13860. |
| 220 | Aryankhesal A, Behzadifar M, Bakhtiari A, Azari S, Behzadifar M. Unleashing the potential: the imperative of political support for health technology assessment in Iran. Health Econ Rev. 2024;14(1):1–5. doi:10.1186/s13561-024-00563-x. |
| 221 | Serpa RC, Rolfe S, Gibson G, Lawrence J, McCall V. Agency and the limits of responsibility: co-management of technology-enabled care in supported housing. Social Sciences. 2023;12(4):248. doi:10.3390/socsci12040248. |
| 222 | Marengo L-L, Barberato-Filho S. Involvement of human volunteers in the development and evaluation of wearable devices designed to improve medication adherence: a scoping review. Sensors (Basel). 2023;23(7):3597. doi:10.3390/s23073597. |
| 223 | Manyazewal T, Woldeamanuel Y, Blumberg HM, Fekadu A, Marconi VC. The potential use of digital health technologies in the African context: a systematic review of evidence from Ethiopia. NPJ Digit Med. 2021;4:13. doi:10.1038/s41746-021-00487-4. |
| 224 | Pirzada P, Wilde A, Doherty GH, Harris-Birtill D. Ethics and acceptance of smart homes for older adults. Informatics Health Soc Care. 2022;47(1):37. doi:10.1080/17538157.2021.1923500. |
| 225 | Barbosa CE, de Lima YO, Costa LF, dos Santos HS, Lyra A, Argôlo M, et al. Future of work in 2050: thinking beyond the COVID-19 pandemic. Eur J Futures Res. 2022;10:19. doi:10.1186/s40309-022-00210-w. |
| 226 | Hung J. Smart elderly care services in China: challenges, progress, and policy development. Sustainability. 2023;15(1):178. doi:10.3390/su15010178. |
| 227 | Lowe B, Fraser I, Souza-Monteiro DM. A change for the better? Digital health technologies and changing food consumption behaviors. Psychol Mark. 2015;32(5):585–600. doi:10.1002/mar.20802. |
| 228 | Sarkar IN. Transforming health data to actionable information: recent progress and future opportunities in health information exchange. Yearb Med Inform. 2022;31(1):203–214. doi:10.1055/s-0042-1742519. |
| 229 | Okada K, Samreth S. Do political regimes matter for technology diffusion? J Knowl Econ. 2024;15(1):3409–3422. doi:10.1007/s13132-023-01266-0. |
| 230 | Mattison G, Canfell OJ, Forrester D, Dobbins C, Smith D, Reid D, et al. A step in the right direction: the potential role of smartwatches in supporting chronic disease prevention in health care. Med J Aust. 2023;218(9):384–388. doi:10.5694/mja2.51920. |
| 231 | Karki S, Weiss A, Dcruz J, Hunt D, Haigood B, Ouakou PT, et al. Assessment of the Chad guinea worm surveillance information system: a pivotal foundation for eradication. PLoS Negl Trop Dis. 2021;15(8):e0009675. doi:10.1371/journal.pntd.0009675. |
| 232 | Dodds S, Russell-Bennett R, Chen T, Oertzen AS, Salvador-Carulla L, Hung YC. Blended human-technology service realities in healthcare. J Serv Theory Pract. 2022;32(1):75–99. doi:10.1108/JSTP-12-2020-0285. |
| 233 | Rasool N, Hassan M. The role of digital mental healthcare in reducing mental health stigma: insights from the Covid-19 pandemic. Curr Psychol. 2025;Jan 13:edssjs.AA10EE72. doi:10.1007/s12144-025-07803-1. |
| 234 | Budhwani S, Fujioka J, Thomas-Jacques T, Vera KD, Challa P, Silva RD, et al. Challenges and strategies for promoting health equity in virtual care: findings and policy directions from a scoping review of reviews. J Am Med Inform Assoc. 2022;29(5):990–999. doi:10.1093/jamia/ocac022. |
| 235 | Baig MM, GholamHosseini H, Moqeem AA, Mirza F, Lindén M. Clinical decision support systems in hospital care using ubiquitous devices: current issues and challenges. Health Informatics J. 2019;25(3):1091–1104. doi:10.1177/1460458217740722. |
| 236 | Piculell E, Skär L, Sanmartin Berglund J, Anderberg P, Bohman D. A concept analysis of health communication in a home environment: perspectives of older persons and their informal caregivers. Scand J Caring Sci. 2021;35(3):1006–1024. doi:10.1111/scs.12928. |
| 237 | Vansimaeys C, Benamar L, Balagué C. Digital health and management of chronic disease: a multimodal technologies typology. Int J Health Plann Manage. 2021;36(4):1107–1125. doi:10.1002/hpm.3161. |
| 238 | Rodrigues SM, Kanduri A, Nyamathi A, Dutt N, Khargonekar P, Rahmani AM. Digital health-enabled community-centered care: scalable model to empower future community health workers using human-in-the-loop artificial intelligence. JMIR Form Res. 2022;6(4):e29535. doi:10.2196/29535. |
| 239 | Wienroth M, Lund Holm Thomsen L, Høstgaard AM. Health technology identities and self: patients' appropriation of an assistive device for self-management of chronic illness. Sociol Health Illn. 2020;42(5):1077–1094. doi:10.1111/1467-9566.13079. |
| 240 | Phuong J, Ordóñez P, Cao J, Moukheiber M, Moukheiber L, Caspi A, et al. Telehealth and digital health innovations: a mixed landscape of access. PLoS Digit Health. 2023;2(12):e0000401. doi:10.1371/journal.pdig.0000401. |
| 241 | Kaiser FK, Wiens M, Schultmann F. Use of digital healthcare solutions for care delivery during a pandemic—chances and (cyber) risks referring to the example of the COVID-19 pandemic. Health Technol. 2021;11(5):1125–1137. doi:10.1007/s12553-021-00541-x. |
| 242 | Western MJ, Armstrong MEG, Islam I, Morgan K, Jones UF, Kelson MJ. The effectiveness of digital interventions for increasing physical activity in individuals of low socioeconomic status: a systematic review and meta-analysis. Int J Behav Nutr Phys Act. 2021;18:21. doi:10.1186/s12966-021-01218-4. |
| 243 | Drazich BF, Li Q, Perrin NA, Szanton SL, Lee JW, Huang CM, et al. The relationship between older adults' technology use, in-person engagement, and pandemic-related mental health. Aging Ment Health. 2023;27(1):156–165. doi:10.1080/13607863.2022.2046695. |
| 244 | Partheniadis K, Stavrakis M. Design and evaluation of a digital wearable ring and a smartphone application to help monitor and manage the effects of Raynaud's phenomenon. Multimedia Tools Appl. 2019;78:3365–3394. doi:10.1007/s11042-018-6514-3. |
| 245 | Asthana S, Jones R, Sheaff R. Why does the NHS struggle to adopt eHealth innovations? A review of macro, meso and micro factors. BMC Health Serv Res. 2019;19:1. doi:10.1186/s12913-019-4790-x. |
| 246 | Wildenbos GA, Peute L, Jaspers M. Facilitators and barriers of electronic health record patient portal adoption by older adults: a literature study. Stud Health Technol Inform. 2017;235:308–312. doi:10.3233/978-1-61499-753-5-308. |
| 247 | Lowe B, Johnson D. Diagnostic and prescriptive benefits of consumer participation in virtual communities of personal challenge. Eur J Mark. 2017;51(11–12):1817–1835. doi:10.1108/EJM-05-2016-0271. |
| 248 | Bol N, Strycharz J, Helberger N, van de Velde B, de Vreese CH. Vulnerability in a tracked society: combining tracking and survey data to understand who gets targeted with what content. New Media Soc. 2020;22(11):1996–2017. doi:10.1177/1461444820924631. |
| 249 | Seghers LE, Anderson M, Schutte NM, Monti G, Haig M, Schmidt J, et al. A scoping review and expert consensus on digital determinants of health/Revue exploratoire et consensus d'experts sur les determinants numeriques de la sante/Revision del alcance y consenso de expertos sobre los determinantes digitales de la salud. Bull World Health Organ. 2025;103(2):110. doi:10.2471/BLT.24.292057. |
| 250 | Pap IA, Oniga S. A review of converging technologies in eHealth pertaining to artificial intelligence. Int J Environ Res Public Health. 2022;19(18):N.PAG. doi:10.3390/ijerph191811413. |
| 251 | Cameron P, Munyan K. Systematic review of telehospice telemedicine and e-health. Telemed e-Health. 2021;27(11):1203–1214. doi:10.1089/tmj.2020.0451. |
| 252 | McMahon C, Aiken M. Introducing digital wellness: bringing cyberpsychological balance to healthcare and information technology. In: 2015 IEEE International Conference on Computer & Information Technology; Ubiquitous Computing & Communications; Dependable, Autonomic & Secure Computing; Pervasive Intelligence & Computing. 2015. p. 1417–1422. doi:10.1109/CIT/IUCC/DASC/PICOM.2015.212. |
| 253 | Qirtas MM, Zafeiridi E, Pesch D, White EB. Loneliness and social isolation detection using passive sensing techniques: scoping review. JMIR mHealth uHealth. 2022;10(4):e34638. doi:10.2196/34638. |
| 254 | Xiong M, Pfau J, Young AT, Wei ML. Artificial intelligence in teledermatology. Curr Dermatol Rep. 2019;8(3):85–90. doi:10.1007/s13671-019-0259-8. |
| 255 | Miller E, Polson D. Apps, avatars, and robots: the future of mental healthcare. Issues Ment Health Nurs. 2019;40(3):208–214. doi:10.1080/01612840.2018.1524535. |
| 256 | Li Y, Alencar A. A tale of two cities: digital place-making and elderly Houniao migration in China. J Ethn Migr Stud. 2023;49(4):1032–1049. doi:10.1080/1369183X.2022.2115630. |
| 257 | Kataria S, Ravindran V. Digital health and rheumatology: the Indian context. Indian J Rheumatol. 2022;17(Suppl):S377–S383. doi:10.4103/injr.injr_127_21. |
| 258 | Bhebhe M, Grobbelaar SS. The innovator’s cookbook: engaging lead users in healthcare product development. Manag Rev Q. 2025;Jan 61. doi:10.1007/s11301-025-00505-9. |
| 259 | Erkılıç CE, Yalçın A. Evaluation of the wearable technology market within the scope of digital health technologies. Gazi J Econ Bus. 2020;6(3):310–323. doi:10.30855/gjeb.2020.6.3.006. |
| 260 | Dundon A, Cipolla D, Mitchell J, Lyapustina S. Reflections on digital health tools for respiratory applications. J Aerosol Med Pulm Drug Deliv. 2020;33(3):127–132. doi:10.1089/jamp.2020.1597. |
| 261 | Jackson DN, Trivedi N, Baur C. Re-prioritizing digital health and health literacy in Healthy People 2030 to affect health equity. Health Commun. 2021;36(10):1155–1162. doi:10.1080/10410236.2020.1748828. |
| 262 | Rosenbaum DL, Piers AD, Schumacher LM, Kase CA, Butryn ML. Racial and ethnic minority enrollment in randomized clinical trials of behavioural weight loss utilizing technology: a systematic review. Obes Rev. 2017;18(7):808–817. doi:10.1111/obr.12545. |
| 263 | Moses JC, Adibi S, Shariful Islam SM, Wickramasinghe N, Nguyen L. Application of smartphone technologies in disease monitoring: a systematic review. Healthcare (Basel). 2021;9(7):889. doi:10.3390/healthcare9070889. |
| 264 | Fosu A, Giba-Fosu N, Mokoka M. Harnessing the digital revolutions within the digital age: opportunities for developing nations in e-commerce, renewable technologies, e-health, and e-education. Afr J Dev Stud. 2025;15(1):95. doi:10.31920/2634-3649/2025/v15n1a5. |
| 265 | Turja T, Kork AA, Ilomäki S, Hellstrand I, Koistinen AK. Care robot literacy: integrating AI ethics and technological literacy in contemporary healthcare. AI Ethics. 2025;5(3):2623–2640. doi:10.1007/s43681-024-00576-6. |
| 266 | Arslantaş S. Artificial intelligence and big data from digital health applications: publication trends and analysis. J Health Organ Manag. [Year unknown];[Volume unknown]:[Pages unknown]. doi:10.1108/JHOM-06-2024-0241. |
| 267 | Adil A, Zaheer L. Socio-cultural, technical & institutional challenges to digital health literacy in Pakistan and rest of the world: a systematic literature review. Pakistan Vision. 2016;17(1):119–145. |
| 268 | Morgan H. ‘Pushed’ self-tracking using digital technologies for chronic health condition management: a critical interpretive synthesis. Digit Health. 2016;2:Jan–41. doi:10.1177/2055207616678498. |
| 269 | Holtzclaw-Stone N, Choo-Kang G, Robles Granda M, Huq A, King T, Kumaran A, et al. Health Technology @ Illinois: innovating for health. In: Proc Int Symp Hum Factors Ergon Healthc. 2021;10(1):148–151. doi:10.1177/2327857921101082. |
| 270 | Schorr EN, Gepner AD, Dolansky MA, Forman DE, Park LG, Petersen KS, et al. Harnessing mobile health technology for secondary cardiovascular disease prevention in older adults: a scientific statement from the American Heart Association. Circ Cardiovasc Qual Outcomes. 2021;14(5):e000103. doi:10.1161/HCQ.0000000000000103. |
| 271 | Segur-Ferrer J, Moltó-Puigmartí C, Pastells-Peiró R, Vivanco-Hidalgo RM. Methodological frameworks and dimensions to be considered in digital health technology assessment: scoping review and thematic analysis. J Med Internet Res. 2024;26:e48694. doi:10.2196/48694. |
| 272 | Prasad VK, Verma A, Bhattacharya P, Shah S, Chowdhury S, Bhavsar M, et al. Revolutionizing healthcare: a comparative insight into deep learning’s role in medical imaging. Sci Rep. 2024;14:edssjs.7676AA2A. doi:10.1038/s41598-024-71358-7. |
| 273 | Jassil FC, Flint SW, Brown A. Lessons learned from the lived experiences of people living with obesity during the first COVID-19 lockdown in the United Kingdom: epidemiology and population health. Int J Obes. 2025;49(6):1173–1180. doi:10.1038/s41366-025-01763-z. |
| 274 | Ozkaynak M, Sircar CM, Frye O, Valdez RS. A systematic review of design workshops for health information technologies. Informatics. 2021;8(2):34. doi:10.3390/informatics8020034. |
| 275 | Daly B, Brawley OW, Gospodarowicz MK, Olopade OI, Fashoyin-Aje L, Smart VW, et al. Remote monitoring and data collection for decentralized clinical trials. JAMA Netw Open. 2024;7(4):e246228. doi:10.1001/jamanetworkopen.2024.6228. |
| 276 | Curtis K, Price K. Factors that influence older people's engagement with digital health technology. Nurs Older People. 2017;29(10):27–30. doi:10.7748/nop.2017.e986. |
| 277 | Endalamaw A, Zewdie A, Wolka E, Assefa Y. A scoping review of digital health technologies in multimorbidity management: mechanisms, outcomes, challenges, and strategies. BMC Health Serv Res. 2025;25:382. doi:10.1186/s12913-025-12548-5. |
| 278 | Shanab BM, Gaffey AE, Schwamm L, Zawalich M, Sarpong DF, Pérez-Escamilla R, et al. Closing the gap: digital innovations to address hypertension disparities. Curr Cardiol Rep. [Year unknown];27(1):23. doi:10.1007/s11886-024-02171-x. |
| 279 | Orlando G, Prior Y, Reeves ND, Vileikyte L. Patient and provider perspective of smart wearable technology in diabetic foot ulcer prevention: a systematic review. Medicina (Kaunas). 2021;57(12):1359. doi:10.3390/medicina57121359. |
| 280 | Dolezel M, Smutny Z. Usage of eHealth/mHealth services among young Czech adults and the impact of COVID-19: an explorative survey. Int J Environ Res Public Health. 2021;18(13):7147. doi:10.3390/ijerph18137147. |
| 281 | Goodridge D, Marciniuk D. Rural and remote care. Chronic Respir Respir Dis. 2016;13(2):192–203. doi:10.1177/1479972316633414. |
| 282 | Yao R, Zhang W, Evans R, Cao G, Rui T, Shen L. Inequities in health care services caused by the adoption of digital health technologies: scoping review. J Med Internet Res. 2022;24(3):e34144. doi:10.2196/34144. |
| 283 | Qiu Y, Huang H, Gai J, De Leo G. The effects of the COVID-19 pandemic on age-based disparities in digital health technology use: secondary analysis of the 2017–2022 Health Information National Trends Survey. J Med Internet Res. 2024;26:e65541. doi:10.2196/65541. |
| 284 | Sieck CJ, Sheon A, Ancker JS, Castek J, Callahan B, Siefer A. Digital inclusion as a social determinant of health. NPJ Digit Med. 2021;4(1):1. doi:10.1038/s41746-021-00413-8. |
| 285 | Gitonga I, Desmond D, Mullen L, Thomas D, Osborne C, O’Loughlin B, et al. Connected health in cancer survivorship: evaluating the usability and utility of the cancer thriving and surviving programme in Ireland. Ir J Med Sci. 2025;[Epub ahead of print]. doi:10.1007/s11845-025-03931-6. |
| 286 | Chan S, Li L, Torous J, Gratzer D, Yellowlees PM. Review of use of asynchronous technologies incorporated in mental health care. Curr Psychiatry Rep. 2018;20(10):1–12. doi:10.1007/s11920-018-0954-3. |
| 287 | Seçkin G, Hughes S, Yeatts D, Degreve T. Digital pathways to positive health perceptions: does age moderate the relationship between medical satisfaction and positive health perceptions among middle-aged and older Internet users? Innov Aging. 2019;3(1):igy039. doi:10.1093/geroni/igy039. |
| 288 | Pong C, Tseng RMWW, Tham YC, Lum E. Current implementation of digital health in chronic disease management: scoping review. J Med Internet Res. 2024;26:e53576. doi:10.2196/53576. |
| 289 | Jeong EB, Lee DH. Metaverse applications in healthcare: opportunities and challenges. Service Business. 2025;19(1):[Epub ahead of print]. doi:10.1007/s11628-024-00577-9. |
| 290 | Sun F, Wang Y, Li W, Li M, Hui T, Shi W. Exploring the influence of living arrangements on mental health among adolescents during the COVID-19 pandemic: a cross-sectional study in China. BMC Public Health. 2025;25:162. doi:10.1186/s12889-025-21368-5. |
| 291 | Zhao Y, Rokhani FZ, Sazlina SG, Devaraj NK, Su J, Chew BH. Defining the concepts of a smart nursing home and its potential technology utilities that integrate medical services and are acceptable to stakeholders: a scoping review. BMC Geriatr. 2022;22(1):[Epub ahead of print]. doi:10.1186/s12877-022-03424-6. |
| 292 | Etingen B, Amante DJ, Martinez RN, Smith BM, Shimada SL, Richardson L, et al. Supporting the implementation of connected care technologies in the Veterans Health Administration: cross-sectional survey findings from the Veterans Engagement with Technology Collaborative (VET-C) cohort. J Particip Med. 2020;12(3):[Epub ahead of print]. doi:10.2196/21214. |
| 293 | Zhou Z, Jin D, He J, Zhou S, Wu J, Wang S, et al. Digital health platform for improving the effect of the active health management of chronic diseases in the community: mixed methods exploratory study. J Med Internet Res. 2024;26:e50959. doi:10.2196/50959. |
| 294 | Wani C, McCann L, Lennon M, Radu C. Digital mental health interventions for adolescents in low- and middle-income countries: scoping review. J Med Internet Res. 2024;26:e51376. doi:10.2196/51376. |
| 295 | Zhu S, Dong Y, Li Y, Wang H, Jiang X, Guo M, et al. Experiences of patients with cancer using electronic symptom management systems: qualitative systematic review and meta-synthesis. J Med Internet Res. 2024;26:e59061. doi:10.2196/59061. |
| 296 | Almeman A. The digital transformation in pharmacy: embracing online platforms and the cosmeceutical paradigm shift. J Health Popul Nutr. 2024;43:60. doi:10.1186/s41043-024-00550-2. |
| 297 | [Author unknown]. Reallocating resources for digitalisation in response to COVID-19: health, education and Industry 4.0. Econ Outlook Southeast Asia, China India. 2021;2021(1):133. |
| 298 | Woolley KE, Bright D, Ayres T, Morgan F, Little K, Davies AR. Mapping inequities in digital health technology within the World Health Organization's European Region using PROGRESS PLUS: scoping review. J Med Internet Res. 2023;25:e44181. doi:10.2196/44181. |
| 299 | Kepper MM, Fowler LA, Kusters IS, Davis JW, Baqer M, Sagui-Henson S, et al. Expanding a behavioral view on digital health access: drivers and strategies to promote equity. J Med Internet Res. 2024;26:e51355. doi:10.2196/51355. |
| 300 | Maaß L, Angoumis K, Freye M, Pan CC. Mapping digital public health interventions among existing digital technologies and internet-based interventions to maintain and improve population health in practice: scoping review. J Med Internet Res. 2024;26:e53927. doi:10.2196/53927. |
| 301 | Anisha SA, Sen A, Bain C. Evaluating the potential and pitfalls of AI-powered conversational agents as humanlike virtual health carers in the remote management of noncommunicable diseases: scoping review. J Med Internet Res. 2024;26:e56114. doi:10.2196/56114. |
| 302 | Sabharwal A, Barua S, Kerr D. A systems approach to achieve equity in healthcare research. GetMobile: Mobile Comput Commun. 2022;25(3):5–Nov. doi:10.1145/3511285.3511287. |
| 303 | Daniore P, Nittas V, von Wyl V. Enrollment and retention of participants in remote digital health studies: scoping review and framework proposal. J Med Internet Res. 2022;24(9):e39910. doi:10.2196/39910. |
| 304 | Kuziemsky CE, Chrimes D, Minshall S, Mannerow M, Lau F. AI quality standards in health care: rapid umbrella review. J Med Internet Res. 2024;26:e54705. doi:10.2196/54705. |
| 305 | Vujkovic B, Brkovic V, Pajičić A, Pavlovic V, Stanisavljevic D, Krajnović D, Jovic Vranes A. Serbian version of the eHealth Literacy Questionnaire (eHLQ): translation, cultural adaptation, and validation study among primary health care users. J Med Internet Res. 2024;26:e57963. doi:10.2196/57963. |
| 306 | Liu KH, Kuo TL, Ko NY, Li YH, Ko WC, Martini S, Li CY. Socioeconomic disparity in the effect of SARS-CoV-2 on outpatient visits among people with type 2 diabetes in Taiwan. Tohoku J Exp Med. 2023;262(3):211–220. doi:10.1620/tjem.2023.J104. |
| 307 | Canfell OJ, Woods L, Meshkat Y, Krivit J, Gunashanhar B, Slade C, et al. The impact of digital hospitals on patient and clinician experience: systematic review and qualitative evidence synthesis. J Med Internet Res. 2024;26:e47715. doi:10.2196/47715. |
| 308 | Baines R, Stevens S, Austin D, Anil K, Bradwell H, Cooper L, et al. Patient and public willingness to share personal health data for third-party or secondary uses: systematic review. J Med Internet Res. 2024;26:e50421. doi:10.2196/50421. |
| 309 | Wang T, Giunti G, Goossens R, Melles M. Timing, indicators, and approaches to digital patient experience evaluation: umbrella systematic review. J Med Internet Res. 2024;26:e46308. doi:10.2196/46308. |
| 310 | Kaihlanen AM, Virtanen L, Kainiemi E, Heponiemi T. Professionals evaluating clients' suitability for digital health and social care: scoping review of assessment instruments. J Med Internet Res. 2023;25:e51450. doi:10.2196/51450. |
| 311 | Borghouts J, Eikey E, Mark G, De Leon C, Schueller SM, Schneider M, et al. Barriers to and facilitators of user engagement with digital mental health interventions: systematic review. J Med Internet Res. 2021;23(3):e24387. doi:10.2196/24387. |
| 312 | Benis A, Tamburis O, Chronaki C, Moen A. One digital health: a unified framework for future health ecosystems. J Med Internet Res. 2021;23(2):e22189. doi:10.2196/22189. |
| 313 | Meudec L, Richebé P, Pascaud J, Mariette X, Nocturne G. Janus kinase inhibitors alter NK cell phenotypes and inhibit their antitumour capacity. Rheumatology (Oxford). 2023;62(8):2855–2863. doi:10.1093/rheumatology/keac710. |
| 314 | Krumholz HM. 12th Korea Healthcare Congress 2021; The time for digital health is almost here. Yonsei Med J. 2022;63(5):493–498. doi:10.3349/ymj.2022.63.5.493. |
| 315 | Nißen M, Rüegger D, Stieger M, Flückiger C, Allemand M, Wangenheim FV, Kowatsch T. The effects of health care chatbot personas with different social roles on the client-chatbot bond and usage intentions: development of a design codebook and web-based study. J Med Internet Res. 2022;24(4):e32630. doi:10.2196/32630. |
| 316 | Goswami A, Poole L, Thorlu-Bangura Z, Khan N, Hanif W, Khunti K, et al. The use of digital health interventions for cardiometabolic diseases among South Asian and Black minority ethnic groups: realist review. J Med Internet Res. 2023;25:e40630. doi:10.2196/40630. |
| 317 | Airola E. Learning and use of eHealth among older adults living at home in rural and nonrural settings: systematic review. J Med Internet Res. 2021;23(12):e23804. doi:10.2196/23804. |
| 318 | Harst L, Lantzsch H, Scheibe M. Theories predicting end-user acceptance of telemedicine use: systematic review. J Med Internet Res. 2019;21(5):e13117. doi:10.2196/13117. |
| 319 | Wang T, Giunti G, Melles M, Goossens R. Digital patient experience: umbrella systematic review. J Med Internet Res. 2022;24(8):e37952. doi:10.2196/37952. |
| 320 | Leach B, Parkinson S, Gkousis E, Abel G, Atherton H, Campbell J, et al. Digital facilitation to support patient access to web-based primary care services: scoping literature review. J Med Internet Res. 2022;24(7):e33911. doi:10.2196/33911. |
| 321 | Abstracts from the 2022 Annual Meeting of the Society of General Internal Medicine. J Gen Intern Med. 2022;Jun 17:1–536. doi:10.1007/s11606-022-07653-8. |
| 322 | Spadaro B, Martin-Key NA, Bahn S. Building the digital mental health ecosystem: opportunities and challenges for mobile health innovators. J Med Internet Res. 2021;23(10):e27507. doi:10.2196/27507. |
| 323 | 1,2; 3,4; 5,6; 1,5; 1. Neurocognitive assessment tools for military personnel with mild traumatic brain injury: scoping literature review. J Med Internet Res. 2021;23(2):N.PAG–N.PAG. doi:10.2196/26360. |
| 324 | Abstracts from the 2020 Annual Meeting of the Society of General Internal Medicine. J Gen Intern Med. 2020;35(Suppl 1):1–779. doi:10.1007/s11606-020-05890-3. |
| 325 | Lyles CR, Nelson EC, Frampton S, Dykes PC, Cemballi AG, Sarkar U. Using electronic health record portals to improve patient engagement: research priorities and best practices. Ann Intern Med. 2020;172(11 Suppl):S123–S129. doi:10.7326/M19-0876. |
| 326 | Abstracts from the 2016 Society of General Internal Medicine Annual Meeting. J Gen Intern Med. 2016;31(Suppl 2):85–922. doi:10.1007/s11606-016-3657-7. |

**Table 2. Full-text articles excluded, with reasons**

| **Study ID** | **Citation** | **Exclusion Reason** |
| --- | --- | --- |
| 1 | Wang Z, Wang Y, Zeng Y, Su J, Li Z. An investigation into the acceptance of intelligent care systems: an extended technology acceptance model (TAM). Sci Rep. 2025;15:Jan-20. doi:10.1038/s41598-025-02746-w. | Factors not influencing digital health technology adoption and sustained use |
| 2 | Kim SD. Application and challenges of the technology acceptance model in elderly healthcare: insights from ChatGPT. Technologies (Basel). 2024;12(5):68. doi:10.3390/technologies12050068. | Factors not influencing digital health technology adoption and sustained use |
| 3 | Ni L, Liu W, Huang Y, Pan M. Technology adoption and promotion in the age of skepticism: examining COVID-19 mitigation through technological and human factors. J Promot Manag. 2023;29(7):905–926. doi:10.1080/10496491.2023.2165207. | Factors not influencing digital health technology adoption and sustained use |
| 4 | Chuang LM, Huang SH. AI-supported healthcare technology resistance and behavioral intention: a serial mediation empirical study on the JD-R model and employee engagement. Systems. 2025;13(4):268. doi:10.3390/systems13040268. | Factors not influencing digital health technology adoption and sustained use |
| 5 | Torkman R, Ghapanchi AH, Ghanbarzadeh R. Investigating antecedents to older adults' uptake of health information systems: a quantitative case study of electronic personal health records. Informatics. 2025;12(1):3. doi:10.3390/informatics12010003. | Factors not influencing digital health technology adoption and sustained use |
| 6 | Health status: the pivot point in trusting smart health-care systems and providers for use. Inf Comput Secur. 2025;33(3):390–407. #26 Alshare 2025. [Exclusion: Factors not influencing digital health technology adoption] | Factors not influencing digital health technology adoption and sustained use |
| 7 | Mwogosi A. AI-driven optimisation of EHR systems implementation in Tanzania's primary health care. Transform Gov People Process Policy. 2025;19(2):288–315. doi:10.1108/TG-08-2024-0195. | Wrong setting |
| 8 | Lin XY, Moxley J, Sharit J, Czaja SJ. Beyond the digital divide: factors associated with adoption of technologies related to aging in place. J Appl Gerontol. 2025;44(6):959–969. doi:10.1177/07334648251318789. | Factors not influencing digital health technology adoption and sustained use |
| 9 | de Andrés-Sánchez J, Arias-Oliva M, Souto-Romero M, Shoukat MH. Antecedents of the intention to use implantable technologies for nonmedical purposes: a mixed-method evaluation. Hum Behav Emerg Technol. 2024;Jan-20. doi:10.1155/hbe2/1064335. | Wrong intervention |
| 10 | Sallam M, Al-Mahzoum K, Alaraji H, et al. Apprehension toward generative artificial intelligence in healthcare: a multinational study among health sciences students. Front Educ. 2025;Jan-15. doi:10.3389/feduc.2025.1542769. | Factors not influencing digital health technology adoption and sustained use |
| 11 | Alruwaili AN, Alshammari AM, Alhaiti A, et al. Neonatal nurses' experiences with generative AI in clinical decision-making: a qualitative exploration in high-risk NICUs. BMC Nurs. 2025;24:Jan-20. doi:10.1186/s12912-025-03044-6. | Wrong setting |
| 12 | Song LL, Li BX, Wu HB, Wu CC, Zhang XQ. Understanding the factors of wearable devices among the patients with thyroid cancer: a modified UTAUT2 model. PLoS ONE. 2024;19(7):Jan-21. doi:10.1371/journal.pone.0305944. | Factors not influencing digital health technology adoption and sustained use |
| 13 | Understanding older adults' acceptance of Chatbots in healthcare delivery: an extended UTAUT model. Front Public Health. 2024;Jan-17. #50 Yu 2024. | Factors not influencing digital health technology adoption and sustained use |
| 14 | Ngusie HS, Kassie SY, Zemariam AB, et al. Understanding the predictors of health professionals' intention to use electronic health record system: extend and apply UTAUT3 model. BMC Health Serv Res. 2024;24:Jan-16. doi:10.1186/s12913-024-11378-1. | Factors not influencing digital health technology adoption and sustained use |
| 15 | Hanqing Z, Chen X, Zhang H, Wong CUI. Enhancing digital health engagement among Asian seniors: investigating the acceptance and use of fitness apps in promoting healthy aging. Appl Sci. 2025;15(5):2294. doi:10.3390/app15052294. | Factors not influencing digital health technology adoption and sustained use |
| 16 | Adoption challenges to artificial intelligence literacy in public healthcare: an evidence based study in Saudi Arabia. Front Public Health. 2025;Jan-20. #56 Kumar 2025. [Exclusion: Wrong outcomes] | Wrong outcomes |
| 17 | Bao H, Lee EWJ. Examining the antecedents and health outcomes of health apps and wearables use: an integration of the technology acceptance model and communication inequality. Behav Inf Technol. 2024;43(4):695–716. doi:10.1080/0144929X.2023.2183062. | Factors not influencing digital health technology adoption and sustained use |
| 18 | Advancing user-centric design and technology adoption for aging populations: a multifaceted approach. Front Public Health. 2024;Sep-1. #63 Stamate 2024. [Exclusion: Review] | Review, protocol or commentary |
| 19 | Mbombi MO, Bopape MA, Ravele T, et al. Applying an E-Learning framework to explore learner nurses' and nurse educators' perceptions about technology platforms in nursing. PLoS ONE. 2025;20(3):Jan-23. doi:10.1371/journal.pone.0312681. | Factors not influencing digital health technology adoption and sustained use |
| 20 | Bertolazzi A, Marzęda-Młynarska K, Kięczkowska J, Zanier ML. Datafication of care: security and privacy issues with health technology for people with diabetes. Societies. 2024;14(9):163. doi:10.3390/soc14090163. | Factors not influencing digital health technology adoption and sustained use |
| 21 | Chen Y, Yuan J, Shi L, Zhou J, Wang H, Li C, et al. Understanding the role of technology anxiety in the adoption of digital health technologies (DHTs) by older adults with chronic diseases in Shanghai: an extension of the unified theory of acceptance and use of technology (UTAUT) model. Healthcare (Basel). 2024;12(14):1421. doi:10.3390/healthcare12141421. | Factors not influencing digital health technology adoption and sustained use |
| 22 | Bridging the digital divide for diabetes care: affordability and adoptability of health technology in Pakistan. Discover Health Syst. 2025;4(1):Jan-14. #80 Fayez 2025. | Factors not influencing digital health technology adoption and sustained use |
| 23 | Ruzi R, Pan Y, Ng ML, Su R, Wang L, Dang J, et al. A speech-based mobile screening tool for mild cognitive impairment: technical performance and user engagement evaluation. Bioengineering (Basel). 2025;12(2):108. doi:10.3390/bioengineering12020108. | Factors not influencing digital health technology adoption and sustained use |
| 24 | Factors influencing the continuance intention of the women's health WeChat public account: an integrated model of UTAUT2 and HBM. Front Public Health. 2024;Dec-1. #82 HeweiMin 2024. | Factors not influencing digital health technology adoption and sustained use |
| 25 | Feasibility of a computerized clinical decision support system delivered via a socially assistive robot during grand rounds: a pilot study. Digit Health. 2025;Jan-21. #85 Å afran 2025. | Factors not influencing digital health technology adoption and sustained use |
| 26 | Mechta M, Mouloudj K, Bouarar AC, Bovsh L, Rasulov A. Customers' intentions to adopt digital health services: an extended TAM. Marketing Sci & Inspir. 2024;19(4):Feb-16. doi:10.46286/msi.2024.19.4.1. | Factors not influencing digital health technology adoption and sustained use |
| 27 | Vlad AL, Popazu C, Lescai AM, Voinescu DC, Baltă AAS. The role of artificial intelligence in the diagnosis and management of rheumatoid arthritis. Medicina (Kaunas). 2025;61(4):689. doi:10.3390/medicina61040689. | Wrong outcomes |
| 28 | Sommer D, Lermer E, Wahl F, Lopera G LI. Assistive technologies in healthcare: utilization and healthcare workers perceptions in Germany. BMC Health Serv Res. 2025;25:Jan-16. doi:10.1186/s12913-024-12162-x. | Factors not influencing digital health technology adoption and sustained use |
| 29 | Fan M, Ukaegbu OC. Information literacy and intention to adopt e-pharmacy: a study based on trust and the theory of reasoned action. BMC Health Serv Res. 2024;24:Jan-18. doi:10.1186/s12913-024-11301-8. | Factors not influencing digital health technology adoption and sustained use |
| 30 | Sestino A, Bernardo A, Rizzo C, Bresciani S. An explorative analysis of the antecedents and consequents of gamification in the digital therapeutic context. Eur J Innov Manag. 2025;28(4):1315–1341. doi:10.1108/EJIM-08-2023-0633. | Factors not influencing digital health technology adoption and sustained use |
| 31 | Hossain MK, Sutanto J, Handayani PW, Haryanto AA, Bhowmik J, Frings-Hessami V. An exploratory study of electronic medical record implementation and recordkeeping culture: the case of hospitals in Indonesia. BMC Health Serv Res. 2025;25:Jan-20. doi:10.1186/s12913-025-12399-0. | Wrong setting |
| 32 | Roy I, Salles J, Neveu E, Larivière-Bastien D, Blondin A, Levac D, et al. Exploring the perspectives of health care professionals on digital health technologies in pediatric care and rehabilitation. J Neuroeng Rehabil. 2024;21:Sep-1. doi:10.1186/s12984-024-01431-9. | Factors not influencing digital health technology adoption and sustained use |
| 33 | Ezeudoka BC, Fan M. Exploring the impact of digital distrust on user resistance to e-health services among older adults: the moderating effect of anticipated regret. Humanities & Soc Sci Commun. 2024;11:Jan-19. doi:10.1057/s41599-024-03457-9. | Factors not influencing digital health technology adoption and sustained use |
| 34 | Necaise A, Mejia C, Kider JT, Shoss M, Amon MJ. Wearables for well-being: a longitudinal in situ study of smartwatches to understand and enhance line-level housekeeping work. Int J Hum-Comput Interact. 2025;41(2):1204–1220. doi:10.1080/10447318.2024.2313887. | Factors not influencing digital health technology adoption and sustained use |
| 35 | Keshavarz H, Saeidnia HR, Wang T. Navigating technostress: a deep dive into health practitioners' technological challenges in hospital settings. BMC Health Serv Res. 2025;25:Sep-1. doi:10.1186/s12913-024-12196-1. | Factors not influencing digital health technology adoption and sustained use |
| 36 | Holtz BE, Kanthawala S, Martin K, Nelson V, Parrott S. Young adults' adoption and use of mental health apps: efficient, effective, but no replacement for in-person care. J Am Coll Health. 2025;73(2):602–610. doi:10.1080/07448481.2023.2227727. | Factors not influencing digital health technology adoption and sustained use |
| 37 | Jang M. Why do people use telemedicine apps in the post-COVID-19 era? Expanded TAM with e-health literacy and social influence. Informatics. 2023;10(4):85. doi:10.3390/informatics10040085. | Factors not influencing digital health technology adoption and sustained use |
| 38 | Technology engagement in enhancing memory functionality in elderly care centers. J Educ Health Promot. 2025;14(1):Aug-1. #128 Al-Habies 2025. [Exclusion: Wrong setting] | Wrong setting |
| 39 | Dom NC, Dapari R, Kamal HA, Harun QN, Salleh SA, Aljaafre AF. Exploring community willingness and barriers to digital solutions and training for dengue prevention: a cross-sectional study in major urban areas of Malaysia. BMC Public Health. 2025;25:Jan-14. doi:10.1186/s12889-025-22677-5. | Factors not influencing digital health technology adoption and sustained use |
| 40 | Yeh TM, Lu HY, Pai FY. Applying multi-criteria decision analysis methods to explore the key factors in using interactive intelligent health promotion equipment. SAGE Open. 2025;15(1):Jan-18. doi:10.1177/21582440251327474. | Factors not influencing digital health technology adoption and sustained use |
| 41 | Willing or reluctant to share health data? A moderated mediation analysis of wearable device usage and data-sharing intentions among older adults. Digit Health. 2025;Jan-13. #137 Zhang 2025. | Factors not influencing digital health technology adoption and sustained use |
| 42 | Tian YJ, Duong V, Buhr E, Felber NA, Schwab DR, Wangmo T. Monitored and cared for at home? Privacy concerns when using smart home health technologies to care for older persons. AJOB Empir Bioeth. 2025;16(2):61–76. doi:10.1080/23294515.2024.2416121. | Factors not influencing digital health technology adoption and sustained use |
| 43 | Cavacece Y, Maggiore G, Resciniti R, Moretta Tartaglione A. Evaluating digital health attributes for users' satisfaction: an application of the Kano model. TQM J. 2025;37(3):831–852. doi:10.1108/TQM-09-2023-0301. | Factors not influencing digital health technology adoption and sustained use |
| 44 | Shah UM, Chiew TK, Mehmood Y. A usability evaluation instrument for pain management mobile applications: an elderly's perspective. Int J Hum-Comput Interact. 2024;40(9):2265–2281. doi:10.1080/10447318.2022.2158517. | Factors not influencing digital health technology adoption and sustained use |
| 45 | Digital health and quality of care in primary health care: an evaluation model. Front Public Health. 2024;Jan-13. #144 Silva 2024. | Factors not influencing digital health technology adoption and sustained use |
| 46 | Influence of doctor-patient trust on the adoption of mobile medical applications during the epidemic: a UTAUT-based analysis. Front Public Health. 2024;Jan-13. #147 DongMeng 2024. | Factors not influencing digital health technology adoption and sustained use |
| 47 | Realdon O, Adorni R, Ginelli D, Micucci D, Blasi V, Bellavia D, et al. Embedding the patient-citizen perspective into an operational framework for the development and the introduction of new technologies in rehabilitation care: the Smart&Touch-ID model. Healthcare (Basel). 2023;11(11):1604. doi:10.3390/healthcare11111604. | Factors not influencing digital health technology adoption and sustained use |
| 48 | Xin Y, Hu W, Deng Y. Digital literacy impacts quality of life among older adults through hierarchical mediating mechanisms. Sci Rep. 2025;15(1):Aug-1. doi:10.1038/s41598-025-04472-9. | Factors not influencing digital health technology adoption and sustained use |
| 49 | Demand, utilization, and supply of community smart senior care services for older people in China. Digit Health. 2025;Jan-17. #153 Fa 2025. | Factors not influencing digital health technology adoption and sustained use |
| 50 | Siette J, Adam PJ, Harris CB. Acceptability of virtual reality to screen for dementia in older adults. BMC Geriatr. 2024;24:Sep-1. doi:10.1186/s12877-024-05115-w. | Factors not influencing digital health technology adoption and sustained use |
| 51 | Shih HJ, Min H, Chang J. Assessing health disparities in digital services and technologies during and after the COVID-19 pandemic: a pooled cross-sectional analysis using HINTS data. Patient Prefer Adherence. 2025;19:87–96. doi:10.2147/PPA.S495318. | Factors not influencing digital health technology adoption and sustained use |
| 52 | Improving consumer trust in digital health: a mixed methods study involving people living with chronic kidney disease. Digit Health. 2025;Jan-16. #172 Catapan 2025. | Factors not influencing digital health technology adoption and sustained use |
| 53 | Amorim P, Paiva J, Silva de Lima J, Portugal da Fonseca L, Martins H, Silva PA. Lessons learned from investigating patients' and physiotherapists' perspectives on the design of a telerehabilitation platform. Disabil Rehabil Assist Technol. 2024;19(6):2377–2388. doi:10.1080/17483107.2023.2287160. | Factors not influencing digital health technology adoption and sustained use |
| 54 | Applying a social determinants of health framework to guide digital innovations that reduce disparities in chronic disease. Psychosom Med. 2023;85(7):659–669. #177 Goldstein 2023. [Exclusion: commentary] | Review, protocol or commentary |
| 55 | Valuable insights into general practice staff's experiences and perspectives on AI-assisted diabetic retinopathy screening—an interview study. Front Med. 2025;Nov-1. #188 Krogh 2025. [Exclusion: wrong outcomes] | Wrong outcomes |
| 56 | Gee B, Clarke T, Garner J, Teague B, Coote G, Dunne A, et al. Understanding stakeholder views of the use of digital therapeutic interventions within children and young people's mental health services. Front Psychiatry. 2025;Jan-14. doi:10.3389/fpsyt.2025.1505345. | Factors not influencing digital health technology adoption and sustained use |
| 57 | The impact of a digital cancer survivorship patient engagement toolkit on older cancer survivors' health outcomes. Comput Inform Nurs. 2025;43(1):Jan-13. #191 Nahm 2025. | Factors not influencing digital health technology adoption and sustained use |
| 58 | Grates M, Brandt M. (How) did self-rated health status shape Internet use among older adults during the COVID-19 pandemic in Germany? New Media Soc. 2025;27(2):1073–1092. doi:10.1177/14614448231188971. | Factors not influencing digital health technology adoption and sustained use |
| 59 | Lee S, Ory MG, Vollmer Dahlke D, Smith ML. Technology use among older adults and their caregivers: cross-sectional survey study. JMIR Aging. 2024;7:Nov-1. doi:10.2196/50759. | Factors not influencing digital health technology adoption and sustained use |
| 60 | Uncovska M, Freitag B, Meister S, Fehring L. Patient acceptance of prescribed and fully reimbursed mHealth apps in Germany: an UTAUT2-based online survey study. J Med Syst. 2023;47(1):Jan-14. doi:10.1007/s10916-023-01910-x. | Factors not influencing digital health technology adoption and sustained use |
| 61 | Luu AP, Nguyen TT, Cao VTC, Ha THD, Chung LTT, Truong TN, et al. Acceptance and user experiences of a wearable device for the management of hospitalized patients in COVID-19–designated wards in Ho Chi Minh City, Vietnam: action learning project. JMIR Hum Factors. 2024;11:Oct-1. doi:10.2196/44619. | Factors not influencing digital health technology adoption and sustained use |
| 62 | Zin KSLT, Kim S, Kim H-S, Feyissa IF. A study on technology acceptance of digital healthcare among older Korean adults using extended TAM (extended technology acceptance model). Admin Sci. 2023;13(2):42. doi:10.3390/admsci13020042. | Factors not influencing digital health technology adoption and sustained use |
| 63 | Factors influencing user's health information discernment abilities in online health communities: based on SEM and fsQCA. Front Public Health. 2024;Jan-21. #213 CaiPingWei 2024. | Factors not influencing digital health technology adoption and sustained use |
| 64 | Kobayashi MA, St. George SM, Leite RO, Noriega Esquives B, Wetstone R, Pulgaron ER, et al. Formative development of a technology-based physical activity intervention for grandparents and grandchildren. J Aging Phys Act. 2022;30(3):482–494. doi:10.1123/japa.2020-0481. | Factors not influencing digital health technology adoption and sustained use |
| 65 | Park EH, Im GP, Zhang J, Lee YH, Chun KH, Park YS. The impact of role overload and role conflict on physicians' technology adoption. Australas J Inf Syst. 2023;27:Jan-37. doi:10.3127/ajis.v27i0.3769. | Factors not influencing digital health technology adoption and sustained use |
| 66 | Yang K, Li Y, Qi H. Determinants of and willingness to use and pay for digital health technologies among the urban elderly in Hangzhou, China. Risk Manag Healthc Policy. 2023;16:463–478. doi:10.2147/RMHP.S393767. | Factors not influencing digital health technology adoption and sustained use |
| 67 | Pejić Bach M, Mihajlović I, Stanković M, Khawaja S, Qureshi FH. Determinants of intention to use of hospital information systems among healthcare professionals. Systems. 2024;12(7):235. doi:10.3390/systems12070235. | Factors not influencing digital health technology adoption and sustained use |
| 68 | Van Rhoon L, McSharry J, Byrne M. Development and testing of a digital health acceptability model to explain the intention to use a digital diabetes prevention programme. Br J Health Psychol. 2022;27(3):716–740. doi:10.1111/bjhp.12569. | Factors not influencing digital health technology adoption and sustained use |
| 69 | Pekmezaris R, Martinez S, Correa Gomez V, Marino J, Goris N, Williams MS, et al. Culturally congruent Latino-adapted telemonitoring of underrepresented adults with type 2 diabetes: the CULTURA-DM2 trial. Clin Diabetes. 2025;43(1):79–91. doi:10.2337/cd24-0002. | Factors not influencing digital health technology adoption and sustained use |
| 70 | Application of SEM and fsQCA to predict m-health adoption in the BoP market. Digit Policy Regul Gov. 2023;25(6):545–567. #260 Hussain 2023. | Factors not influencing digital health technology adoption and sustained use |
| 71 | Perceived technology usefulness for caregiving among unpaid caregivers: a national cross-sectional study. Front Public Health. 2025;Aug-1. #263 Smith 2025. | Factors not influencing digital health technology adoption and sustained use |
| 72 | A service-user digital intervention to collect real-time safety information on acute, adult mental health wards: the WardSonar mixed-methods study. Health Soc Care Deliv Res (HSDR). 2024;12(15/16):1–182. #264 Baker 2024. | Factors not influencing digital health technology adoption and sustained use |
| 73 | Fernandes T, Costa M. Privacy concerns with COVID-19 tracking apps: a privacy calculus approach. J Consum Mark. 2023;40(2):181–192. doi:10.1108/JCM-03-2021-4510. | Factors not influencing digital health technology adoption and sustained use |
| 74 | Liu S, Zheng D. Impacts of tourists' trust, perception and acceptance of health quick response technology on responsible pandemic travel behaviours. J Hosp Tour Technol. 2023;14(2):278–294. doi:10.1108/JHTT-11-2021-0330. | Factors not influencing digital health technology adoption and sustained use |
| 75 | Digitisation and health: second nationwide survey of internet users in Germany. Digit Health. 2024;Jan-16. #271 DeSantis 2024. | Factors not influencing digital health technology adoption and sustained use |
| 76 | Choi SL, Hites L, Bolland AC, Lee J, Payne-Foster P, Bissell K. Telehealth uptake among middle-aged and older Americans during COVID-19: chronic conditions, social media communication, and race/ethnicity. Aging Ment Health. 2024;28(1):160–168. doi:10.1080/13607863.2022.2149696. | Factors not influencing digital health technology adoption and sustained use |
| 77 | Teo SH, Chew EAL, Ng DWL, Tang WE, Koh GCH, Teo VHY. Implementation and use of technology-enabled blood pressure monitoring and teleconsultation in Singapore's primary care: a qualitative evaluation using the socio-technical systems approach. BMC Prim Care. 2023;24(1):Jan-15. doi:10.1186/s12875-023-02014-8. | Factors not influencing digital health technology adoption and sustained use |
| 78 | The impact of digital literacy on personal health: evidence from the China family panel study. Int J Health Plann Manage. 2024;39(5):1411–1433. #289 Li 2024. | Factors not influencing digital health technology adoption and sustained use |
| 79 | Ma Y, Achiche S, Pomey M-P, Paquette J, Adjtoutah N, Vicente S, et al. Adapting and evaluating an AI-based chatbot through patient and stakeholder engagement to provide information for different health conditions: master protocol for an adaptive platform trial (the MARVIN Chatbots Study). JMIR Res Protoc. 2024;13:Jan-22. doi:10.2196/54668. | Factors not influencing digital health technology adoption and sustained use |
| 80 | Lee EWJ, Bao H, Wu YS, Wang MP, Wong YJ, Viswanath K. Examining health apps and wearable use in improving physical and mental well-being across U.S., China, and Singapore. Sci Rep. 2024;14(1):Dec-1. doi:10.1038/s41598-024-61268-z. | Factors not influencing digital health technology adoption and sustained use |
| 81 | Zheng D, Liu S, Lu W. Do you trust digital health pass? Understanding tourists' responses toward using health QR codes in pandemic travel. J China Tour Res. 2023;19(1):31–49. doi:10.1080/19388160.2022.2087817. | Factors not influencing digital health technology adoption and sustained use |
| 82 | Panggabean B, Suharjo B, Sumarwan U, Yuliati LN. Strategy development to increase consumer intention of purchasing prescription drugs through e-pharmacy in Indonesia. Int J Risk Saf Med. 2024;35(1):49–73. doi:10.3233/JRS-220067. | Wrong outcomes |
| 83 | Digital health interventions for individuals with disabilities and their impacts on health, quality of life, and social participation. Digit Health. 2024;Jan-14. #321 Kandeel 2024. Excluded: Review. | Review, protocol or commentary |
| 84 | Yang H, Peng Z, Guo X, Lai K-H. Balancing online pharmacy services for patient adherence: a stimulus-organism-response perspective. Internet Res. 2021;31(6):2000–2032. doi:10.1108/INTR-10-2020-0603. | Wrong intervention |
| 85 | Alanezi F. Factors influencing patients' engagement with ChatGPT for accessing health-related information. Crit Public Health. 2024;34(1):Jan-20. doi:10.1080/09581596.2024.2348164. | Factors not influencing digital health technology adoption and sustained use |
| 86 | A mobile app to predict and manage behavioral and psychological symptoms of dementia: development, usability, and users' acceptability. Digit Health. 2024;Dec-1. #348 Cho 2024. | Factors not influencing digital health technology adoption and sustained use |
| 87 | Das J, Barry G, Walker R, Vitorio R, Morris R, Stuart S. The integration of technology into a home-based visuo-cognitive training intervention for people with Parkinson's: is the future digital? PLoS ONE. 2023;18(6):Jan-20. doi:10.1371/journal.pone.0285100. | Factors not influencing digital health technology adoption and sustained use |
| 88 | Lee SM, Lee D. Healthcare wearable devices: an analysis of key factors for continuous use intention. Serv Bus. 2020;14(4):503–531. doi:10.1007/s11628-020-00428-3. | Factors not influencing digital health technology adoption and sustained use |
| 89 | de Looff PC, Noordzij ML, Nijman HLI, Goedhard L, Bogaerts S, Didden R. Putting the usability of wearable technology in forensic psychiatry to the test: a randomized crossover trial. Front Psychiatry. 2024;Jan-13. doi:10.3389/fpsyt.2024.1330993. | Factors not influencing digital health technology adoption and sustained use |
| 90 | Liang Z, Melcer E, Khotchasing K, Hoang NH. Co-design personal sleep health technology for and with university students. Front Digit Health. 2024;Oct-1. doi:10.3389/fdgth.2024.1371808. | Factors not influencing digital health technology adoption and sustained use |
| 91 | Kostka G, Habich-Sobiegalla S. In times of crisis: public perceptions toward COVID-19 contact tracing apps in China, Germany, and the United States. New Media Soc. 2024;26(4):2256–2294. doi:10.1177/14614448221083285. | Factors not influencing digital health technology adoption and sustained use |
| 92 | Use of telemedicine technology among general practitioners during COVID-19: a modified technology acceptance model study in Poland. Int J Environ Res Public Health. 2022;19(17):10937. #366 Walczak 2022. | Factors not influencing digital health technology adoption and sustained use |
| 93 | Lai Y, Panagiotopoulos P, Lioliou E. Empowering users with medical artificial intelligence technologies. Proc Eur Conf Inf Syst (ECIS). 2023;Jan-17. | Factors not influencing digital health technology adoption and sustained use |
| 94 | Wong AKC, Tso WC, Su JJ, Hui VC, Chow KKS, Wong SM, et al. Effectiveness of support from community health workers on the sustained use of a wearable monitoring device among community-dwelling older adults: a randomized trial protocol. PLoS ONE. 2023;18(12):Nov-1. doi:10.1371/journal.pone.0294517. | Review, protocol or commentary |
| 95 | De Regge M, Van Baelen F, Beirão G, Den Ambtman A, De Pourcq K, Dias JC, et al. Personal and interpersonal drivers that contribute to the intention to use gerontechnologies. Gerontology. 2020;66(2):176–186. doi:10.1159/000502113. | Factors not influencing digital health technology adoption and sustained use |
| 96 | Disease characteristics influence the privacy calculus to adopt electronic health records: a survey study in Germany. Digit Health. 2024;Jan-17. #384 vonKalckreuth 2024. | Factors not influencing digital health technology adoption and sustained use |
| 97 | Determinants of American adults' use of digital health and willingness to share health data to providers, family, and social media: a cross-sectional study. Comput Inform Nurs. 2023;41(11):892–902. #385 Tolentino 2023. | Factors not influencing digital health technology adoption and sustained use |
| 98 | Analysis of demand and influencing factors for smart senior care among older adults in underdeveloped regions of western China: a case study of Lanzhou. Front Public Health. 2024;Jan-13. #386 YunhuaWang 2024. Excluded: Wrong setting. | Wrong setting |
| 99 | Automation in contemporary clinical information systems: a survey of AI in healthcare settings. Yearb Med Inform. 2023;32(1):115–126. #388 Magrabi 2023. Excluded: Review. | Review, protocol or commentary |
| 100 | Walle AD, Butta FW, Kassie SY, Chereka AA, Kanfe SG, Dubale AT, Enyew EB, Dube GN, Shibabaw AA, Hunde MK, Kitil GW, Ferede TA, Wubante SM, Baykemagn ND, Demsash AW, Ardila CM. Healthcare professionals' attitude to using mobile health technology and its associated factors in a resource-limited country—an implication for digital health implementers: a cross-sectional study. Biomed Res Int. 2024;2024:Sep-1. doi:10.1155/2024/1631376. | Factors not influencing digital health technology adoption and sustained use |
| 101 | Obeid S, Mashiach-Eizenberg M, Gur A, Lavy I. Examining ethnic disparities in digital healthcare services utilization: insights from Israel. J Multidiscip Healthc. 2023;16:3533–3544. doi:10.2147/JMDH.S429121. | Factors not influencing digital health technology adoption and sustained use |
| 102 | Barbaric A, Munteanu C, Ross H, Cafazzo JA. A voice app design for heart failure self-management: proof-of-concept implementation study. JMIR Form Res. 2022;6(12):Dec-1. doi:10.2196/40021. | Factors not influencing digital health technology adoption and sustained use |
| 103 | Maximizing the value of patient and public involvement in the digital health co-design process: a qualitative descriptive study with design leaders and patient-public partners. PLoS Digit Health. 2023;2(10):Jan-18. #406 Voorheis 2023. | Factors not influencing digital health technology adoption and sustained use |
| 104 | Behavioral patterns of supply and demand sides of health services for the elderly in sustainable digital transformation: a mixed methods study. Int J Environ Res Public Health. 2022;19(13):8221-N.PAG. #407 Zhou 2022. | Factors not influencing digital health technology adoption and sustained use |
| 105 | Sivakumar CLV, Mone V, Abdumukhtor R. Addressing privacy concerns with wearable health monitoring technology. WIREs Data Min Knowl Discov. 2024;14(3):Jan-13. doi:10.1002/widm.1535. | Factors not influencing digital health technology adoption and sustained use |
| 106 | Yap KYL, Ho J, Toh PST. Development of a Metaverse Art Gallery of Image Chronicles (MAGIC) for healthcare education: a digital health humanities approach to patients' medication experiences. Information. 2024;15(8):431. doi:10.3390/info15080431. | Wrong setting |
| 107 | Allen MR, Webb S, Mandvi A, Frieden M, Tai-Seale M, Kallenberg G. Navigating the doctor-patient-AI relationship – a mixed-methods study of physician attitudes toward artificial intelligence in primary care. BMC Prim Care. 2024;25(1):Dec-1. doi:10.1186/s12875-024-02282-y. | Factors not influencing digital health technology adoption and sustained use |
| 108 | Kouvonen A, Kemppainen T, Taipale S, Olakivi A, Wrede S, Kemppainen L. Health and self-perceived barriers to internet use among older migrants: a population-based study. BMC Public Health. 2022;22(1):Dec-1. doi:10.1186/s12889-022-12874-x. | Factors not influencing digital health technology adoption and sustained use |
| 109 | Ilozumba O, Kabukye J, de Keizer N, Cornet R, Broerse JEW. Cancer as a death sentence: developing an initial program theory for an IVR intervention. Health Promot Int. 2022;37(3):Dec-1. doi:10.1093/heapro/daac070. | Wrong setting |
| 110 | Papadopoulos I, Koulouglioti C, Lazzarino R, Ali S, Wright S, Martín-García Á, et al. Views about perceived training needs of health care professionals in relation to socially assistive robots: an international online survey. Contemp Nurse. 2023;59(4–5):344–361. doi:10.1080/10376178.2023.2238095. | Factors not influencing digital health technology adoption and sustained use |
| 111 | Patterns of acceptance and use of digital health services among the persistent frequent attenders of outpatient care: a qualitatively driven multimethod analysis. Digit Health. 2023;Jan-14. #431 Virtanen 2023. | Factors not influencing digital health technology adoption and sustained use |
| 112 | Morris ME, Brusco NK, Jones J, Taylor NF, East C, Semciw AI, et al. The widening gap between the digital capability of the care workforce and technology-enabled healthcare delivery: a nursing and allied health analysis. Healthcare. 2023;11(7):994. doi:10.3390/healthcare11070994. | Factors not influencing digital health technology adoption and sustained use |
| 113 | Applying an extended UTAUT2 model to explain user acceptance of lifestyle and therapy mobile health apps: survey study. JMIR mHealth uHealth. 2022;10(1):160–175. #437 Schomakers 2022. | Factors not influencing digital health technology adoption and sustained use |
| 114 | Xu Z, Zhang D, Zheng X, Lee RCM, Wong SYS, Wong CKM. Use, satisfaction, and preference of online health services among older adults with multimorbidity in Hong Kong primary care during COVID-19. BMC Geriatr. 2023;23(1):Aug-1. doi:10.1186/s12877-023-04061-3. | Factors not influencing digital health technology adoption and sustained use |
| 115 | Wearable technologies for healthy ageing: prospects, challenges, and ethical considerations. J Frailty Aging. 2024;13(2):149–156. #441 Canali 2024. Excluded: Review. | Review, protocol or commentary |
| 116 | Rothgangel A, Kanera I, van den Heuvel R, Wientgen M, Jamin G, Lenssen T, Braun S. Physiotherapists' clinical use and acceptance of a telemonitoring platform during anterior cruciate ligament rehabilitation: a prospective clinical study. Disabil Rehabil Assist Technol. 2022;17(2):184–191. doi:10.1080/17483107.2020.1774810. | Wrong setting |
| 117 | Digital health literacy and use of patient portals among Spanish-preferred patients in the United States: a cross-sectional assessment. Front Public Health. 2024;Aug-1. #447 Philpot 2024. | Factors not influencing digital health technology adoption and sustained use |
| 118 | Lee J, Kim J, Ory MG. The impact of immersive virtual reality meditation for depression and anxiety among inpatients with major depressive and generalized anxiety disorders. Front Psychol. 2024;Oct-1. doi:10.3389/fpsyg.2024.1471269. | Wrong outcomes |
| 119 | Serrano KM, Mendes GHS, Lizarelli FL, Ganga GMD. Assessing the telemedicine acceptance for adults in Brazil. Int J Health Care Qual Assur. 2021;34(1):35–51. doi:10.1108/IJHCQA-06-2020-0098. | Factors not influencing digital health technology adoption and sustained use |
| 120 | Exploring telehealth readiness in a resource limited setting: digital and health literacy among older people in rural India (DAHLIA). Geriatrics. 2022;7(2):28. #463 Rasekaba 2022. | Factors not influencing digital health technology adoption and sustained use |
| 121 | Sezgin E, Kocaballi AB, Dolce M, Skeens M, Militello L, Huang Y, et al. Chatbot for social need screening and resource sharing with vulnerable families: iterative design and evaluation study. JMIR Hum Factors. 2024;11:Jan-20. doi:10.2196/57114. | Factors not influencing digital health technology adoption and sustained use |
| 122 | Tsarfati B, Cojocaru D. Introducing computerized technology to nurses: a model based on cognitive instrumental and social influence processes. Healthcare. 2023;11(12):1788. doi:10.3390/healthcare11121788. | Factors not influencing digital health technology adoption and sustained use |
| 123 | Designing mobile health applications to support walking for older adults. Int J Environ Res Public Health. 2023;20(4):3611. #471 Felberbaum 2023. | Factors not influencing digital health technology adoption and sustained use |
| 124 | Dr. Google: physicians–the web–patients triangle: digital skills and attitudes towards e-health solutions among physicians in South Eastern Poland–a cross-sectional study in a pre-COVID-19 era. Int J Environ Res Public Health. 2023;20(2):978. #473 Burzyńska 2023. | Factors not influencing digital health technology adoption and sustained use |
| 125 | Paccoud I, Valero MM, Marín LC, Bontridder N, Ibrahim A, Winkler J, et al. Patient perspectives on the use of digital medical devices and health data for AI-driven personalised medicine in Parkinson's disease. Front Neurol. 2024;Sep-1. doi:10.3389/fneur.2024.1453243. | Factors not influencing digital health technology adoption and sustained use |
| 126 | O’Connor S, Treanor C, Ward E, Wickens R, O’Connell A, Culliford L, et al. Acceptability of home monitoring for neovascular age-related macular degeneration reactivation: a qualitative study. Stud Health Technol Inform. 2024;315:425–429. doi:10.3233/SHTI240182. | Factors not influencing digital health technology adoption and sustained use |
| 127 | Relationship between citizens' perspective on digital health and underlying health risks. Digit Health. 2023;Jan-14. #479 Pedersen 2023. | Factors not influencing digital health technology adoption and sustained use |
| 128 | Cervera-Torres S, Núñez-Benjumea FJ, de Arriba Muñoz A, Chicchi Giglioli IA, Fernández-Luque L. Digital health for emotional and self-management support of caregivers of children receiving growth hormone treatment: a feasibility study protocol. BMC Med Inform Decis Mak. 2022;22(1):Jan-13. doi:10.1186/s12911-022-01935-1. | Factors not influencing digital health technology adoption and sustained use |
| 129 | Lin Z, Zhang Y, Matteson M, Li X, Tu X, Zhou Y, Wang J. Older adults’ eHealth literacy and the role libraries can play. J Librariansh Inf Sci. 2021;53(3):488–498. doi:10.1177/0961000620962847. | Factors not influencing digital health technology adoption and sustained use |
| 130 | Implementing automated text messaging for patient self-management in the Veterans Health Administration: qualitative study applying the Nonadoption, Abandonment, Scale-up, Spread, and Sustainability framework. JMIR mHealth uHealth. 2021;9(11):Dec-1. #485 Yakovchenko 2021. | Factors not influencing digital health technology adoption and sustained use |
| 131 | Arcury TA, Sandberg JC, Melius KP, Quandt SA, Leng X, Latulipe C, et al. Older adult internet use and eHealth literacy. J Appl Gerontol. 2020;39(2):141–150. doi:10.1177/0733464818807468. | Factors not influencing digital health technology adoption and sustained use |
| 132 | Dinsmore J, Hannigan C, Smith S, Murphy E, Kuiper JML, O’Byrne E, et al. A digital health platform for integrated and proactive patient-centered multimorbidity self-management and care (ProACT): protocol for an action research proof-of-concept trial. JMIR Res Protoc. 2021;Jan-16. doi:10.2196/22125. | Review, protocol or commentary |
| 133 | Harim J, Yoo JH, Goh M, Song H. Deep breathing in your hands: designing and assessing a DTx mobile app. Front Digit Health. 2024;Dec-1. doi:10.3389/fdgth.2024.1287340. | Factors not influencing digital health technology adoption and sustained use |
| 134 | Victoria-Castro AM, Martin M, Yamamoto Y, Ahmad T, Arora T, Calderon F, et al. Pragmatic randomized trial assessing the impact of digital health technology on quality of life in patients with heart failure: design, rationale and implementation. Clin Cardiol. 2022;45(8):839–849. doi:10.1002/clc.23848. | Factors not influencing digital health technology adoption and sustained use |
| 135 | Tan TC, Roslan NEB, Li JW, Zou X, Chen X, Ratnasari, Santosa A. Patient acceptability of symptom screening and patient education using a chatbot for autoimmune inflammatory diseases: survey study. JMIR Form Res. 2023;7(1):Dec-1. doi:10.2196/49239. | Factors not influencing digital health technology adoption and sustained use |
| 136 | Mobile consulting as an option for delivering healthcare services in low-resource settings in low- and middle-income countries: a mixed-methods study. Digit Health. 2021;7:Jan-24. #500 Harris 2021. | Factors not influencing digital health technology adoption and sustained use |
| 137 | Reliable contactless monitoring of heart rate, breathing rate, and breathing disturbance during sleep in aging: digital health technology evaluation study. JMIR mHealth uHealth. 2024;12:Jan-20. #502 GRavindran 2024. Excluded: Wrong setting. | Wrong setting |
| 138 | Miller E, Zelenko O. The caregiving journey: arts-based methods as tools for participatory co-design of health technologies. Soc Sci. 2022;11(9):396-N.PAG. doi:10.3390/socsci11090396. | Factors not influencing digital health technology adoption and sustained use |
| 139 | Ruetsch C, Davis T, Liberman JN, Velligan DI, Robinson D, Jaeger C, et al. Prescriber attitudes, experiences, and proclivities toward digital medicine and how they influence adoption of digital medicine platforms. Neuropsychiatr Dis Treat. 2021;17:3715–3726. doi:10.2147/NDT.S318344. | Factors not influencing digital health technology adoption and sustained use |
| 140 | Kassaw M, Amare G, Shitu K, Tilahun B, Assaye BT. Willingness to use remote patient monitoring among cardiovascular patients in a resource-limited setting: a cross-sectional study. Front Digit Health. 2024;Sep-1. doi:10.3389/fdgth.2024.1437134. | Factors not influencing digital health technology adoption and sustained use |
| 141 | Strubbia C, Levack WMM, Grainger R, Takahashi K, Tomori K. Use of an iPad app (Aid for Decision-making in Occupational Choice) for collaborative goal setting in interprofessional rehabilitation: qualitative descriptive study. JMIR Rehabil Assist Technol. 2021;8(4):Jan-13. doi:10.2196/33027. | Factors not influencing digital health technology adoption and sustained use |
| 142 | Keeling DI, de Ruyter K, Mousavi S, Laing A. Technology push without a patient pull: examining digital unengagement (DU) with online health services. Eur J Mark. 2019;53(9):1701–1732. doi:10.1108/EJM-10-2017-0692. | Outdated article |
| 143 | Paccoud I, Baumann M, Le Bihan E, Pétré B, Breinbauer M, Böhme P, et al. Socioeconomic and behavioural factors associated with access to and use of personal health records. BMC Med Inform Decis Mak. 2021;21(1):Nov-1. doi:10.1186/s12911-020-01383-9. | Factors not influencing digital health technology adoption and sustained use |
| 144 | Wang Y, Li M, Chen N, Hou W, Zhang Y, Zhu M, Tang X. Older adults' perspectives on adopting smart home technology for 'proactive health': a qualitative study. J Adv Nurs. 40252233. doi:10.1111/jan.16964. #524. | Factors not influencing digital health technology adoption and sustained use |
| 145 | Ravi RK, Baby P, Pareek B, George TJ, Mohamed MG, Ahmed SK. Preferences, acceptability and usage of mobile health applications among undergraduate nursing students: a multisite, cross-sectional survey. SAGE Open Nurs. 2024;Oct-1. doi:10.1177/23779608241247431. | Factors not influencing digital health technology adoption and sustained use |
| 146 | Kim J, Jeon SW, Byun H, Yi E. Exploring e-health literacy and technology-use anxiety among older adults in Korea. Healthcare. 2023;11(11):1556. doi:10.3390/healthcare11111556. | Factors not influencing digital health technology adoption and sustained use |
| 147 | Kumar A, Tandon P, Meratwal G. Electronic health literacy and demand for digital health technology among the elderly in Rajasthan. J Surg Specialties Rural Pract. 2023;4(2):57–62. doi:10.4103/jssrp.jssrp_5_23. | Factors not influencing digital health technology adoption and sustained use |
| 148 | Pietrantonio F, Vinci A, Maurici M, Ciarambino T, Galli B, Signorini A, et al. Intra- and extra-hospitalization monitoring of vital signs—two sides of the same coin: perspectives from LIMS and Greenline-HT study operators. Sensors. 2023;23(12):5408. doi:10.3390/s23125408. | Factors not influencing digital health technology adoption and sustained use |
| 149 | Steffens D, Denehy L, Solomon M, Koh C, Ansari N, McBride K, et al. Consumer perspectives on the adoption of a prehabilitation multimodal online program for patients undergoing cancer surgery. Cancers. 2023;15(20):5039. doi:10.3390/cancers15205039. | Wrong outcomes |
| 150 | Atkinson 2024. Emerging health technologies: opportunities and practical applications for the exercise professional. ACSM Health Fitness J. 2024;28(5):44–52. #540. Excluded: Commentary. | Review, protocol or commentary |
| 151 | Chen 2023. Effectiveness, safety and patients' perceptions of an immersive virtual reality–based exercise system for poststroke upper limb motor rehabilitation: proof-of-concept and feasibility RCT. Digit Health. 2023;Jan-33. #541. Excluded: Wrong intervention. | Wrong intervention |
| 152 | Yoo 2023. Digital technology use, technological self-efficacy, and subjective well-being among North Korean migrants during the COVID-19 pandemic: moderated moderation. Digit Health. 2023;Jan-13. #543. | Factors not influencing digital health technology adoption and sustained use |
| 153 | Smuck M, Odonkor CA, Wilt JK, Schmidt N, Swiernik MA. The emerging clinical role of wearables: factors for successful implementation in healthcare. NPJ Digit Med. 2021;4(1):Aug-1. doi:10.1038/s41746-021-00418-3. | Factors not influencing digital health technology adoption and sustained use |
| 154 | KC S, Tewolde S, Laverty AA, Costelloe C, Papoutsi C, Reidy C, et al. Uptake and adoption of the NHS App in England: an observational study. Br J Gen Pract. 2023;73(737):e932–e940. doi:10.3399/bjgp.2022.0150. | Factors not influencing digital health technology adoption and sustained use |
| 155 | Pathiravasan CH, Zhang Y, Wang X, Trinquart L, Benjamin EJ, Borrelli B, et al. Factors associated with long-term use of digital devices in the electronic Framingham Heart Study. NPJ Digit Med. 2022;5(1):Nov-1. doi:10.1038/s41746-022-00735-1. | Factors not influencing digital health technology adoption and sustained use |
| 156 | Gordon NP, Yin C, Lo JC. Examining whether patient portal and video visit use differs by race and ethnicity among older adults in a US integrated health care delivery system: cross-sectional electronic health record and survey-based study. JMIR Aging. 2024;7:Jan-16. doi:10.2196/63814. | Factors not influencing digital health technology adoption and sustained use |
| 157 | He Q, Keith D, Eckhoff DO, Park C, Ng BP. Accessibility and utilization of telehealth services during the COVID-19 pandemic among Medicare beneficiaries by diabetes status. Res Gerontol Nurs. 2023;16(3):134–146. doi:10.3928/19404921-20230301-01. | Factors not influencing digital health technology adoption and sustained use |
| 158 | Ben-Sasson A, Ben-Sasson E, Jacobs K, Malinovitch R. The relationship between users' technology approaches and experiences in a child development mobile application. Health Technol. 2020;10(5):1079–1094. doi:10.1007/s12553-020-00457-y. | Factors not influencing digital health technology adoption and sustained use |
| 159 | Jeng MY, Pai FY, Yeh TM. Antecedents for older adults' intention to use smart health wearable devices—technology anxiety as a moderator. Behav Sci (Basel). 2022;12(4):114. doi:10.3390/bs12040114. | Factors not influencing digital health technology adoption and sustained use |
| 160 | Assaye 2023. Association of digital health literacy and information-seeking behaviors among physicians during COVID-19 in Ethiopia: a cross-sectional study. Digit Health. 2023;9:Nov-1. #563. | Factors not influencing digital health technology adoption and sustained use |
| 161 | Campos BA, Cummins E, Sonnay Y, Brindle ME, Cauley CE. A digital communication intervention to support older adults and their care partners transitioning home after major surgery: protocol for a qualitative research study. JMIR Res Protoc. 2024;13:Aug-1. doi:10.2196/59067. | Factors not influencing digital health technology adoption and sustained use |
| 162 | Lim D, Norman R, Robinson S. Consumer preference to utilise a mobile health app: a stated preference experiment. PLoS ONE. 2020;15(2):Dec-1. doi:10.1371/journal.pone.0229546. | Factors not influencing digital health technology adoption and sustained use |
| 163 | Mohlman J, Basch CH. Health-related correlates of demonstrated smartphone expertise in community-dwelling older adults. J Appl Gerontol. 2021;40(5):510–518. doi:10.1177/0733464820902304. | Factors not influencing digital health technology adoption and sustained use |
| 164 | Alazzam MB, Al-Radaideh AT, Alhamarnah RA, Alassery F, Hajjej F, Halasa A. A survey research on the willingness of gynecologists to employ mobile health applications. Comput Intell Neurosci. 2021;2021:Jul-1. doi:10.1155/2021/1220374. | Integrity compromised |
| 165 | Alvarez-Perez 2021. Cocreation of massive open online courses to improve digital health literacy in diabetes: pilot mixed methods study. JMIR Diabetes. 2021;6(4):Jan-13. #581. Excluded: Wrong outcomes. | Wrong outcomes |
| 166 | Yermukhanova L, Bagiyarova F, Zholdassova N, Seidakhmetova A, Otarov Y. Opportunities and challenges in the adoption of digital health technologies in Kazakhstan. Bratisl Med J. 2025;Jan-17. doi:10.1007/s44411-025-00218-1. | Review, protocol or commentary |
| 167 | Göttgens I, Darweesh SKL, Bloem BR, Oertelt-Prigione S. A multidimensional gender analysis of health technology self-efficacy among people with Parkinson’s disease. J Neurol. 2024;271(10):6750–6760. doi:10.1007/s00415-024-12635-3. | Factors not influencing digital health technology adoption and sustained use |
| 168 | Slevin 2019. A qualitative study of chronic obstructive pulmonary disease patient perceptions of the barriers and facilitators to adopting digital health technology. Digit Health. 2019;5:N.PAG-N.PAG. #598. Excluded: Outdated article. | Outdated article |
| 169 | Li L, Peng W, Kononova A, Bowen M, Cotten SR. Factors associated with older adults' long-term use of wearable activity trackers. Telemed e-Health. 2020;26(6):769–775. doi:10.1089/tmj.2019.0052. | Factors not influencing digital health technology adoption and sustained use |
| 170 | Su D, Michaud TL, Estabrooks P, Schwab RJ, Eiland LA, Hansen G, et al. Diabetes management through remote patient monitoring: the importance of patient activation and engagement with the technology. Telemed e-Health. 2019;25(10):952–959. doi:10.1089/tmj.2018.0205. | Outdated article |
| 171 | Zhou W, Cho Y, Shang S, Jiang Y. Use of digital health technology among older adults with cancer in the United States: findings from a national longitudinal cohort study (2015–2021). J Med Internet Res. 2023;25:edsgcl.763679714. doi:10.2196/46721. | Factors not influencing digital health technology adoption and sustained use |
| 172 | Naiseh M, Babiker A, Al-Shakhsi S, Cemiloglu D, Al-Thani D, Montag C, Ali R. Attitudes towards AI: the interplay of self-efficacy, well-being, and competency. J Technol Behav Sci. 2025;Jan-14. doi:10.1007/s41347-025-00486-2. | Wrong setting |
| 173 | Polak S, Sillevis Smitt M, Jacobs A, van Leeuwen C, Doyle J, Cullen-Smith S, Dinsmore J. A user typology for digital health self-management technologies: a qualitative study of the long-term user experiences of older adults with a digital self-management platform for multimorbidity (ProACT). J Healthc Inform Res. 2025;Jan-20. doi:10.1007/s41666-024-00183-4. | Factors not influencing digital health technology adoption and sustained use |
| 174 | Ranieri J, Guerra F, Angione AL, Di Giacomo D, Passafiume D. Cognitive reserve and digital confidence among older adults as new paradigm for resilient aging. Gerontol Geriatr Med. 2021;1:Aug-1. doi:10.1177/2333721421993747. | Factors not influencing digital health technology adoption and sustained use |
| 175 | Bentley CL, Otesile O, Bacigalupo R, Elliott J, Noble H, Hawley MS, et al. Feasibility study of portable technology for weight loss and HbA1c control in type 2 diabetes. BMC Med Inform Decis Mak. 2016;16:Jan-15. doi:10.1186/s12911-016-0331-2. | Outdated article |
| 176 | Bienvenida JCM, Aschbrenner KA, Bartels SJ, Brunette M, Naslund JA, Grinley T. A survey of online and mobile technology use at peer support agencies. Psychiatric Q. 2018;89(3):539–548. doi:10.1007/s11126-017-9561-4. | Outdated article |
| 177 | Gitonga I, Desmond D, Maguire R. Who uses connected health technologies after a cancer diagnosis? Evidence from the US Health Information National Trends Survey. 38744797. doi:10.1007/s11764-024-01615-1. #622. | Factors not influencing digital health technology adoption and sustained use |
| 178 | Manyazewal T, Ali MK, Kebede T, Solomon S, Hailemariam D, Patel SA, Escoffery C, Woldeamanuel Y, Marinucci F, Joseph M, Getinet T, Amogne W, Fekadu A, Marconi VC. Digital continuous glucose monitoring systems for patients with HIV-diabetes comorbidity in Ethiopia: a situational analysis. Sci Rep. 2024;14(1):edssjs.5818B4A2. doi:10.1038/s41598-024-79967-y. | Factors not influencing digital health technology adoption and sustained use |
| 179 | Pan CC, De Santis KK, Muellmann S, Hoffmann S, Spallek J, Barnils NP, Ahrens W, Zeeb H, Schüz B. Sociodemographics and digital health literacy in using wearables for health promotion and disease prevention: cross-sectional nationwide survey in Germany. J Prev. 2024;Jan-21. doi:10.1007/s10935-024-00821-y. | Factors not influencing digital health technology adoption and sustained use |
| 180 | Silvera-Tawil D, Cameron J, Li J, Varnfield M, Allan LP, Harris M, Lannin NA, Redd C, Cadilhac DA. Multicomponent support program for secondary prevention of stroke using digital health technology: co-design study with people living with stroke or transient ischemic attack. 26:e54604. 39172512. doi:10.2196/54604. #628. | Factors not influencing digital health technology adoption and sustained use |
| 181 | Wang N, Zhou S, Liu Z, Han Y. Perceptions and satisfaction with the use of digital medical services in urban older adults of China: mixed methods study. JMIR. 26:e48654. 39303282. doi:10.2196/48654. #633. | Factors not influencing digital health technology adoption and sustained use |
| 182 | Fanta GB, Pretorius L. Sociotechnical factors of sustainable digital health systems: a system dynamics model. Health Policy Technol. 2023;12(1):N.PAG-N.PAG. doi:10.1016/j.hlpt.2023.100729. | Factors not influencing digital health technology adoption and sustained use |
| 183 | Pearce LMN, Pryor J, Redhead J, Sherrington C, Hassett L. Advanced technology in a real-world rehabilitation setting: longitudinal observational study on clinician adoption and implementation. JMIR. 26:e60374. 39753210. doi:10.2196/60374. #640. | Factors not influencing digital health technology adoption and sustained use |
| 184 | Lai WX, Visaria A, Østbye T, Malhotra R. Prevalence and correlates of use of digital technology for managing hypertension among older adults. J Hum Hypertens. 2023;37(1):80–87. doi:10.1038/s41371-022-00654-4. | Factors not influencing digital health technology adoption and sustained use |
| 185 | Greenhalgh T, Wherton J, Papoutsi C, Lynch J, Hughes G, A'Court C, et al. Beyond adoption: a new framework for theorizing and evaluating nonadoption, abandonment, and challenges to the scale-up, spread, and sustainability of health and care technologies. JMIR J Med Internet Res. 2017;19(11):e367. 29092808. doi:10.2196/jmir.8775. #656. | Outdated article |
| 186 | Haddad TC, Maita KC, Inselman JW, Avila FR, Torres-Guzman RA, Coffey JD, et al. Patient satisfaction with a multisite, multiregional remote patient monitoring program for acute and chronic condition management: survey-based analysis. JMIR. 25:e44528. 37343182. doi:10.2196/44528. #666. | Factors not influencing digital health technology adoption and sustained use |
| 187 | Yang SH. The implications of using digital technologies in the management of COVID-19: comparative study of Japan and South Korea. JMIR. 25:e45705. 37279042. doi:10.2196/45705. #667. | Factors not influencing digital health technology adoption and sustained use |
| 188 | Journal of Medical Internet Research. Investigator experiences using mobile technologies in clinical research: qualitative descriptive study. 2021;23(2):N.PAG-N.PAG. EPTOC149133834. #673. Excluded: Wrong setting. | Wrong setting |
| 189 | Chiu CJ, Liu CW. Understanding older adult's technology adoption and withdrawal for elderly care and education: mixed method analysis from national survey. JMIR J Med Internet Res. 2017;19(11):e374. 29101093. doi:10.2196/jmir.7401. #678. | Outdated article |
| 190 | Dharanikota S, LeRouge CM, Lyon V, Durneva P, Thompson M. Identifying enablers of participant engagement in clinical trials of consumer health technologies: qualitative study of influenza home testing. JMIR. 23(9):e26869. 34519664. doi:10.2196/26869. #680. | Factors not influencing digital health technology adoption and sustained use |
| 191 | Srinivasan M, Asch S, Vilendrer S, Thomas SC, Bajra R, Barman L, et al. Qualitative assessment of rapid system transformation to primary care video visits at an academic medical center. Ann Intern Med. 2020;173(7):527–535. 32628536. doi:10.7326/M20-1814. #685. | Wrong outcomes |
| 192 | Choi NG, DiNitto DM, Lee OE, Choi BY. Internet and health information technology use and psychological distress among older adults with self-reported vision impairment: case-control study. JMIR. 22(6):e17294. 32490851. doi:10.2196/17294. #688. | Factors not influencing digital health technology adoption and sustained use |
| 193 | Paré G, Leaver C, Bourget C. Diffusion of the digital health self-tracking movement in Canada: results of a national survey. JMIR J Med Internet Res. 20(5):e177. 29720359. doi:10.2196/jmir.9388. #690. | Outdated article |
| 194 | Featherall J, Lapin B, Chaitoff A, Havele SA, Thompson N, Katzan I. Characterization of patient interest in provider-based consumer health information technology: survey study. JMIR J Med Internet Res. 20(4):e128. 29674312. doi:10.2196/jmir.7766. #691. | Outdated article |
