# Supplementary material for: Advancing foundational models in digital health technology adoption: A systematic literature review of multidisciplinary factors
Source: PLOS Digit Health. 2026 Sep 2;5(9):e0001690. doi: 10.1371/journal.pdig.0001690 (PMC13537594; doi:10.1371/journal.pdig.0001690)
Supplement: Supplementary file 6 [file pdig.0001690.s004.docx]

**S2 Table. Summary of extracted data from included studies**

| **No** | **Full Reference** | **Country/Setting** | **Population Characteristics** | **Study design** | **Sample size** | **Data collection method** | **Main findings** | **Study limitations** |
| --- | --- | --- | --- | --- | --- | --- | --- | --- |
| [[1]](https://formative.jmir.org/2021/2/e19074/) | Freise L, Neves AL, Flott K, Harrison P, Kelly J, Darzi A, et al. Assessment of patients’ ability to review electronic health record information to identify potential errors: cross-sectional web-based survey. JMIR Form Res. 2021;5(2):e19074. doi:10.2196/19074 | United Kingdom – Care Information Exchange (CIE), a patient-controlled electronic health record portal in North-West London, initiated at Imperial College Healthcare NHS Trust. | Registered users of the Care Information Exchange who accessed their records during the data collection period and were invited via an on-screen message while logging in. Respondents represented a variety of clinical departments (e.g. oncology, colposcopy, HIV service, renal, rheumatology). Information collected on participant demographics, characteristics, and context was limited. | Cross-sectional web-based survey using the Care Information Exchange (CIE) portal to assess patients’ perceptions of understanding their electronic health records (EHRs) and identifying errors | 210 responses from patients (160 unique respondents, some completed the survey multiple times) | Online web-based survey (Qualtrics), including structured multiple-choice and unstructured free-text questions | Understanding of EHR information: Majority of patients understood their EHR information; 58.6% had no difficulty, while 26.7% experienced some.  Medical terminology barrier: Patients struggled with jargon and acronyms, which limited understanding.  Interpretation of test results barrier: Patients were unsure about the meaning of the results and what actions to take.  Portal layout and accessibility barrier: Patients found it difficult to locate or access updated information in the portal.  Presentation of results barrier: Graphical displays hindered interpretation; some preferred raw numbers.  Incorrect information in records: Patients noticed wrong details such as addresses, appointment times and misclassified or false test results.  Incomplete information in records: Missing details, such as appointments and test results, limited the usefulness of the portal. | Limited demographic data collected  Possible repeat responses by same patients  Lack of objective error verification  Need for more robust future studies |
| [[2]](https://mhealth.jmir.org/2021/1/e23155/) | Yang S, Chen Y, Zhou L, Huang Y, Dai J. Willingness to adopt mHealth among Chinese parents during the COVID-19 outbreak: cross-sectional questionnaire study. JMIR Mhealth Uhealth. 2021;9(1):e23155. doi:10.2196/23155 | China – Online recruitment via social media groups and webpages during the COVID-19 outbreak. | Chinese parents who completed an online survey during the COVID-19 outbreak. Parents were excluded if survey completion time was <120 seconds; child was older than 14 years; parent did not live with the child during the pandemic; parent(s) had confirmed COVID-19; no internet access; duplicate IP submissions; or missing items. | Online cross-sectional questionnaire survey conducted during the COVID-19 outbreak (January 25-February 15, 2020) | 254 parents (172 female, 82 male) | Structured online questionnaire (23 items) distributed via social media groups (e.g. breastfeeding, kindergarten/primary school groups, paediatric researchers’ pages) | Age factor: Parents aged 26-35 years showed higher willingness to adopt mobile health (mHealth) compared to younger parents.  Pandemic interest factor: Higher attention to the COVID-19 pandemic increased willingness.  Medical provider recommendation: Advice from medical professionals strongly influenced adoption.  Child’s chronic disease: Parents with children suffering from chronic illness were most likely to adopt.  Internet use duration: Longer daily internet use increased the likelihood of adoption.  Prior experience: Previous mHealth users were more willing to adopt during COVID-19.  Hygiene behaviour correlation: Frequent mHealth use was linked to better hygiene practices.  Health monitoring correlation: Frequent mHealth use related to child temperature checks and mental health care. | Small sample size limits generalisability  Potential self-reporting biases  Confounding factors not fully accounted for  Cross-sectional design limits causal inference |
| [[3]](https://www.jmir.org/2021/6/e28944) | Saigí-Rubió F, Vidal-Alaball J, Torrent-Sellens J, Jiménez-Zarco A, López Segui F, Carrasco Hernandez M, et al. Determinants of Catalan public primary care professionals’ intention to use digital clinical consultations (eConsulta) in the post–COVID-19 context: mixed methods study. J Med Internet Res. 2021;23(6):e28944. doi:10.2196/28944 | Spain – Public primary care system in Catalonia. | Public primary care professionals in Catalonia, including physicians, nurses, reproductive and sexual health service staff, social workers, and collective health workers. | Mixed qualitative and quantitative methodology; study based on the Technology Acceptance Model (TAM) with exploratory and confirmatory factor analysis plus logistic regression | 1,189 professionals (79.2% women, mean age 48 years; 62.2% physicians, 35.2% nurses/other health staff, 2.2% client care staff) | Online questionnaire (validated via pre-test) distributed to Catalan Health Institute staff across 285 primary care teams | Perceived benefits: The strongest predictor of intention was the perceived usefulness of eConsulta in improving efficiency and patient relationships.  Type of use: Habitual or broader use of eConsulta increased the likelihood of continued adoption.  Environmental pressure: Influence from patients, colleagues and institutions shaped adoption decisions.  Experience with technology: Prior familiarity with ICTs and telemedicine boosted adoption.  Gender influence: Gender differences were significant, especially among physicians.  Degree of implementation: Higher levels of institutional implementation encouraged adoption.  Professional profile: Type of professional role influenced adoption differently (physicians vs nurses).  Nurse group factors: For nurses, perceived benefits and level of use were the strongest predictors; environmental pressure also mattered.  Physician group factors: For physicians, perceived benefits, gender, type of use, environmental pressure and ease of use were significant. | Response bias inherent in surveys  Use of a new, unvalidated survey instrument  Timing during pandemic may affect responses |
| [[4]](https://www.frontiersin.org/journals/medicine/articles/10.3389/fmed.2024.1421559/full) | Abebe SA, Endehabtu BF, Walle AD, Hailu DT, Yeshaneh AT, Dres EA, et al. Intention to use personal health record system and its predictors among chronic patients enrolled at public hospitals in Bahir Dar city, northwest Ethiopia: using modified UTAUT2 model. Front Med (Lausanne). 2024;11:1421559. doi:10.3389/fmed.2024.1421559 | Ethiopia – Public hospitals in Bahir Dar City, northwest Ethiopia (Amhara regional state). | Adult chronic patients (≥18 years) with follow-up for diabetes mellitus, hypertension, epilepsy, and/or cardiac heart failure in Bahir Dar City public hospitals. | Institutional-based cross-sectional study using a modified Unified Theory of Acceptance and Use of Technology 2 (UTAUT2) model among chronic patients at Bahir Dar city public hospitals | 908 participants (response rate 98.27%), majority >44 years old, 50.7% male, 70% urban residents, 34.1% higher education | Questionnaire developed in English, translated into Amharic, pre-tested, and collected via Kobo Collect with interviewer administration | Intention level: Less than half of chronic patients intended to use personal health records (PHRs).  Performance expectancy: Strongest predictor of intention; patients perceived PHRs as useful.  Effort expectancy: Ease of use increased intention to adopt.  Social influence: External encouragement (family, friends or co-workers) significantly impacted adoption.  Facilitating conditions: Availability of resources and support encouraged adoption.  Hedonic motivation: No significant effect on intention.  Price value: No significant effect on intention.  Habit: No significant effect on intention.  Age moderation: Younger patients (≤40) had a stronger link between effort expectancy and intention.  Gender moderation: No significant gender differences found. | Cross-sectional design limits causal inference  Restricted to one city and public hospitals  Limited generalisability |
| [[5]](https://www.frontiersin.org/journals/health-services/articles/10.3389/frhs.2025.1460077/full) | Alameraw TA, Asemahagn MA, Gashu KD, Walle AD, Kelkay JM, Mitiku AB, et al. Intention to use telemonitoring for chronic illness management and its associated factors among nurses and physicians at public hospitals in Bahir Dar, northwest Ethiopia: using a modified UTAUT2 model. Front Health Serv. 2025;5:1460077. doi:10.3389/frhs.2025.1460077 | Ethiopia – Public hospitals in Bahir Dar City, northwest Ethiopia (Addis Alem Primary Hospital; Felege Hiwot Comprehensive Specialized Hospital; Tibebe Ghion Specialized Teaching Hospital). | Nurses and physicians working at the listed public hospitals. The study included permanently employed nurses and physicians and excluded those not permanently employed for <6 months. | Facility-based cross-sectional study using a modified UTAUT2 model among nurses and physicians at Bahir Dar public hospitals | 732 respondents (93.7% response rate) - 55.7% male, 67.6% nurses, median age 35 years | Questionnaire developed from UTAUT2 constructs, translated into Amharic, back-translated, pre-tested, and distributed with case scenarios to support understanding | Intention level: More than half of nurses and physicians intended to use telemonitoring.  Performance expectancy: Strongest predictor; belief in improved work performance and service quality increased adoption.  Effort expectancy: Ease of learning and using telemonitoring enhanced intention.  Facilitating conditions: Availability of resources and infrastructure positively influenced adoption.  Habit: Established use patterns increased intention to use telemonitoring.  Social influence: No significant effect on intention among participants.  Hedonic motivation: No significant effect on intention.  Gender moderation: Gender moderated effort expectancy; stronger for men.  Age moderation: Younger professionals had a stronger effort expectancy effect; adults had a stronger facilitating conditions effect. | Cross-sectional design limits causality  Limited generalisability to other settings |
| [[6]](https://www.mdpi.com/2078-2489/13/11/509) | Alanazi F, Gay V, Alturki R. Poor compliance of diabetic patients with AI-enabled e-health self-care management in Saudi Arabia. Information. 2022;13(11):509. doi:10.3390/info13110509 | Saudi Arabia – Hospitals in the Jeddah area, including King Abdulaziz Medical City Hospital. | Adult Saudi patients with diabetes aged ≥18 years with more than one year of diabetes treatment. Excluded were individuals aged <18 years, non-Saudi citizens, and those with less than one year of treatment. | Quantitative cross-sectional survey conducted with diabetic patients in Jeddah, Saudi Arabia, to assess compliance with artificial intelligence (AI)-enabled e-health self-care management | 210 valid responses (58.5% female; 54.8% between 25-50 years old; 57.4% had a bachelor’s/diploma; 50.5% unemployed; 59.9% low income) | Questionnaire (Arabic, pilot tested) following CHERRIES guidelines; distributed to patients registered on CDSS via smartphone applications; used 4-point Likert scale; SPSS v27 Spearman correlation used for analysis | Medication adherence: High compliance with prescribed medical treatment.  Diet control: Low compliance with dietary recommendations.  Physical activity: Very low compliance with exercise regimens.  E-health registration: Less than half are registered in hospital e-health systems.  System availability: Limited hospital e-health system presence reduced access.  Blood glucose monitoring: Higher than prescribed compliance, but poor e-reporting.  Insulin injections: High compliance with injections, but low e-reporting to hospitals.  Self-care programme: Less than half followed a structured self-care programme.  Reporting of abnormal conditions: Very low reporting to hospitals.  Education and income influence: Higher education and income are linked with better experience and attitude toward e-health.  Patient experience: Positive experience with e-health linked to better attitude. | Restricted to Jeddah adult diabetics  Small sample size may affect precision  Focused more on self-care than e-health specifically |
| [[7]](https://www.frontiersin.org/journals/public-health/articles/10.3389/fpubh.2024.1385713/full) | Aldekhyyel RN, Alshuaibi F, Alsaaid O, Bin Moammar F, Alanazy T, Namshah A, et al. Exploring behavioral intention to use telemedicine services post COVID-19: a cross-sectional study in Saudi Arabia. Front Public Health. 2024;12:1385713. doi:10.3389/fpubh.2024.1385713 | Saudi Arabia – Public venues in Riyadh (Riyadh International Book Fair and Riyadh Front); data collection in June 2022. | Saudi citizens aged ≥18 years (“health consumers”) recruited at the venues and completing a web-based survey. | Online survey to assess Saudi citizens’ behavioural intention to use telemedicine services post-COVID-19, examining factors such as electronic health (eHealth) literacy and attitudes | 385 participants (51% female; 57% aged 18-20; 71% reported internet proficiency; 56% uninsured) | Structured web-based survey (Google Forms), validated and translated into Arabic, administered via QR code at two large public events in Riyadh (Book Fair and Riyadh Front) | High intention to use: Majority of participants reported strong willingness to use telemedicine.  Positive attitudes: Strong agreement that telemedicine saves time, money and increases access.  Perceived complexity: Half of the participants found telemedicine video consultations complex.  eHealth literacy association: Higher literacy scores linked to higher intention.  Attitudes as mediator: Attitudes significantly mediated the effect of eHealth literacy on intention.  No demographic effect: Age, gender, income, employment and insurance showed no significant effect.  Younger population bias: The majority of respondents were under 28, potentially influencing high adoption. | Reliance on self-reported data  Over-representation of young, urban population  Exclusion of other potential factors |
| [[8]](https://journals.sagepub.com/doi/full/10.1177/00380385231218695) | Alevizou P, Michaelidou N, Daskalopoulou A, Appiah-Campbell R. Self-tracking among young people: lived experiences, tensions and bodily outcomes. Sociology. 2024;58(4):947-64. doi:10.1177/00380385231218695 | United Kingdom. | Young people aged 18–24 years who use a wearable device and/or a self-tracking mobile application; participants were British. | Interpretivist qualitative design using a life story approach to explore the lived experiences of self-tracking among young people | 23 participants (16 female, 7 male; aged 18-24; majority White, some Black and Asian; predominantly middle class; all British) | Semi-structured life story interviews (50-70 minutes each), online, audio-recorded, transcribed, pseudonymised; researcher also used wearable devices for empathetic insight | Learning process: Self-tracking adoption began through gifting, imitation and informal learning.  Social learning risks: Negative parental influence shaped problematic relationships with self-tracking.  Informal digital learning: Young people relied on Google/social media for self-tracking guidance.  Motivational use: Devices provided satisfaction, nudges and motivation to continue body work.  Private project: Self-tracking became controlling, obsessive and linked to negative mental health.  Social media pressure: Exposure to influencers created unrealistic body ideals.  Shared project: Sharing self-tracking data fostered competition, validation and motivation. | Homogenous sample (predominantly White, middle-class, British)  Need for education about self-tracking’s risks  One-size-fits-all design of self-tracking technologies problematic |
| [[9]](https://etasr.com/index.php/ETASR/article/view/8747) | Aljohani N. Digital health transformation in Saudi Arabia: examining the impact of health information seeking on m-health adoption during the COVID-19 pandemic. Eng Technol Appl Sci Res. 2025;15(1):19933-40. doi: 10.48084/etasr.8747 | Saudi Arabia – National context during the COVID-19 pandemic. | Users in Saudi Arabia who met the inclusion criteria of being at least 18 years old and having prior experience using m-health applications. | Online survey based on the Unified Theory of Acceptance and Use of Technology (UTAUT) framework with added construct of health information seeking; focused on Saudi m-health app users during COVID-19 | 343 valid respondents (56% male, 44% female; majority aged 25-34, most had bachelor’s degree) | Online questionnaire (5-point Likert scale), bilingual (English/Arabic), pilot-tested, distributed via social media (Twitter, WhatsApp health groups) | Health information seeking: Strongest predictor of adoption; users prioritised information access over technical performance.  Social influence: Positive effect on adoption intention through peer and community encouragement.  Effort expectancy: Ease of use significantly improved intention to adopt m-health apps.  Performance expectancy: Not a significant driver of adoption in this context.  Age limitation: Findings mainly reflect younger users; older adults are under-represented. | Quantitative-only design  Exclusion of other potential variables  Findings mainly reflect younger users |
| [[10]](https://journals.plos.org/digitalhealth/article?id=10.1371/journal.pdig.0000586) | Alkaabi A, Elsori D. Navigating digital frontiers in UAE healthcare: a qualitative exploration of healthcare professionals’ and patients’ experiences with AI and telemedicine. PLOS Digit Health. 2025;4(4):e0000586. doi:10.1371/journal.pdig.0000586 | United Arab Emirates – Hospitals in Dubai and Abu Dhabi. | Healthcare professionals (doctors, nurses, telemedicine specialists), patients who had interacted with AI and telemedicine services, administrators from adopting healthcare institutions, and IT staff involved in implementation. | Descriptive qualitative design using thematic analysis to explore healthcare professionals’ and patients’ experiences with AI and telemedicine in the UAE | 15 participants (8 healthcare professionals, 7 patients; mix of doctors, nurses, administrators, IT staff and patients) | Face-to-face and virtual semi-structured interviews (about 60 minutes), pre-tested interview guide; transcribed verbatim, thematically analysed with independent coding and member checking | Patient-centric care: Telemedicine empowered patients and strengthened doctor-patient relationships.  Cost and time efficiency: AI and telemedicine reduced costs, duplication and time burdens.  Infectious disease control: Remote care reduced exposure and enabled outbreak detection.  Chronic disease management: Telemedicine supported continuity of care; AI-enabled predictive monitoring.  Misdiagnosis and misinformation: Lack of face-to-face contact raised diagnostic risks.  Safety and ethical concerns: Biases, regulation and trust issues limited adoption.  Financial barriers: High costs and lack of insurance coverage restricted access.  Infrastructural challenges: Data storage, workflow disruption and small-institution gaps hindered adoption.  Resistance to change: Professionals feared loss of autonomy and resisted disruption.  Skills gap: Lack of technical expertise limited effective use.  Awareness and training facilitators: Awareness campaigns and flexible training boosted adoption.  Stakeholder involvement: Engaging providers in design fostered ownership and alignment.  Incentives: Financial and non-financial incentives encouraged adoption.  Patient experience: Convenience, patient-doctor communication and health monitoring improved satisfaction.  Privacy and human touch concerns: Patients feared data misuse and loss of empathy. | Small sample size, qualitative design limits generalisability  Restricted to Dubai and Abu Dhabi  Purposive sampling narrowed perspectives |
| [[11]](https://www.iieta.org/journals/ijsdp/paper/10.18280/ijsdp.200309) | Al-Shami SA, Kusmantini T, Purwandari S, Sharif MS, Doheir M, Rashid N, et al. System service quality factors and their impact on user satisfaction and continuance intention to use m-health: the moderating influence of monetary cost. Int J Sustain Dev Plan. 2025;20(3):1015-27. doi:10.18280/ijsdp.200309 | United Arab Emirates – Abu Dhabi, including the Imperial College London Diabetes Center. | Young adults aged 20–30 years with type 2 diabetes who used m-health services in Abu Dhabi, UAE. | Survey of type 2 diabetes (T2D) patients in Abu Dhabi, UAE, based on an extended information systems (IS) success model including service quality, interaction quality, information quality, user satisfaction and monetary cost as moderators | 292 valid respondents (169 female, 113 male, aged 20-30, young adults with T2D) | Structured online questionnaire distributed via WhatsApp, Facebook and Instagram; analysed using SmartPLS 3.0 with bootstrapping (5000 resamples) | Interaction quality strongly drives continuance intention: ITQ had the highest positive effect on users’ satisfaction and their continuous intention to use m-health.  System quality supports satisfaction and continuance intention: SQ significantly influenced both user satisfaction and continuous intention to use m-health.  Information quality affects satisfaction but not continuance: IFQ was significant for user satisfaction but had no effect on continuance intention.  User satisfaction mediates service quality and continuance intention: Satisfaction strengthened the link between SQ, ITQ, IFQ and continuance intention.  Monetary cost moderates the satisfaction-continuance relationship: Higher perceived cost increased the strength of the relationship between satisfaction and continuance intention.  Accessibility and availability of the system are critical: SQ emphasised availability and accessibility, enabling anytime/anywhere health services. | Limited to one institution and disease type  UAE-specific context limits generalisability  Self-report bias possible  Online survey excluded digitally disadvantaged patients  Cross-sectional design prevents longitudinal insights  Likert scale limitations |
| [[12]](https://www.mdpi.com/1660-4601/19/7/4289) | Alsharif AH. Attitudes of patients with chronic diseases toward management eHealth applications systems in post-COVID-19 times. Int J Environ Res Public Health. 2022;19(7):4289. doi:10.3390/ijerph19074289 | Saudi Arabia – National context, framed within post-/ongoing COVID-19 period and Saudi Vision 2030. | Adults aged >18 years living in Saudi Arabia who were accessing or using eHealth services; described as patients with chronic diseases/chronic illnesses. | Online survey of adult e-health users in Saudi Arabia to assess attitudes of patients with chronic diseases toward e-health applications post-COVID-19 | 234 valid responses (53.4% male, 46.6% female; majority aged 18-24; 55.9% bachelor’s degree holders) | Structured online questionnaire (18 items); bilingual (English/Arabic), pilot tested; distributed via social media and WhatsApp groups | Necessity-driven adoption: Majority of patients adopted e-health mainly out of necessity, not interest.  Low willingness for future use: More than half indicated they would not continue using e-health after the pandemic.  Privacy and security concerns: A significant portion worried about the confidentiality of health data.  Limited interest in e-health: Few participants were highly interested, with most only somewhat or not interested.  Preferred usage for primary healthcare: Most patients used e-health for diagnosis, advice and treatment.  Barriers to adoption: Many disagreed that e-health was secure or easy to share information.  Socio-demographic influence: Age and gender differences influenced future adoption likelihood. | Small and skewed sample size  Many incomplete responses reduced validity  Mostly descriptive analysis, limited statistical power  Need for more focus on older populations |
| [[13]](https://peerj.com/articles/cs-2773/) | Al-Somali SA. Integrating artificial intelligence (AI) in healthcare: advancing older adults’ health management in Saudi Arabia through AI-powered chatbots. PeerJ Comput Sci. 2025;11:e2773. doi:10.7717/peerj-cs.2773 | Saudi Arabia – Study conducted under King Abdulaziz University, Jeddah. | Individuals aged 60 years and above who used healthcare chatbot solutions. | Survey based on UTAUT2 and the Expectancy Confirmation Model (ECM), focusing on older adults’ sustained use of AI-powered healthcare chatbots in Saudi Arabia | 158 respondents, all aged ≥60 (46.2% male, 53.8% female; 67.7% bachelor’s degree holders; income distribution skewed toward lower income) | Structured questionnaire via Google Forms; snowball sampling through WhatsApp; 5-point Likert scale items adapted from prior validated studies; analysis via PLS-SEM (SmartPLS 4.0) | Hedonic motivation increases satisfaction: Enjoyment from using chatbots significantly improves satisfaction among older adults.  Facilitating conditions strengthen satisfaction: Adequate infrastructure and support encourage higher satisfaction.  Social influence not significant: Peer and family influence did not significantly affect satisfaction.  Performance expectancy strongly drives satisfaction: Belief in chatbots’ usefulness significantly increases satisfaction.  Effort expectancy improves satisfaction: Simplicity and ease of use positively affect satisfaction.  Confirmation enhances satisfaction: Meeting expectations increases satisfaction with chatbots.  Perceived security influences both satisfaction and continuance: Data protection strongly affects adoption.  Satisfaction predicts continuous intention: Higher satisfaction leads to stronger continuance intention.  Perceived humanness mediates satisfaction-continuance link: Human-like chatbot design strengthens the relationship. | Limited sample size and geographic focus  Selection bias from digital-only sampling  Self-report and recall bias  Cross-sectional design prevents causal inference  Narrow scope of variables studied |
| [[14]](https://journals.sagepub.com/doi/10.1177/20552076251336271) | Alsyouf A, Al-Momani AM, Alsubahi N, et al. Predicting digital contact tracing tool adoption during COVID-19 from the perspective of TAM: the role of trust, fear, privacy, anxiety, and social media. Digit Health. 2025;11:20552076251336271. doi:10.1177/20552076251336271 | Saudi Arabia – National setting. | Saudi participants who were smartphone users in the Kingdom; participants were aged ≥18 years. | Quantitative, cross-sectional design using an online survey distributed during Saudi Arabia’s COVID-19 lockdown | 400 participants (52.5% male, 49% aged 18-34 years, 54% with a bachelor’s degree and 59% from the Western Province) | Online self-administered questionnaire (Google Form), translated into Arabic and validated by experts, shared on social media and community groups | Usefulness affects intention: Perceived usefulness had a significant positive effect on intention to use digital contact tracing (DCT) tools.  Effort expectancy affects usefulness: Perceived ease of use strongly increased perceived usefulness.  Effort expectancy affects intention: Perceived ease of use had a significant positive impact on adoption intention.  Intention drives behaviour: Intention to use significantly led to actual DCT tool usage.  Trust facilitates intention: Trust in government significantly increased intention to use DCT tools.  Social media awareness shows no effect: Social media awareness did not significantly influence DCT usage.  Social influence affects adoption: Social influence (peer behaviour and recommendations) significantly increased actual DCT usage.  Anxiety influences acceptance: COVID-19-induced anxiety significantly shaped technology acceptance, mediated by social influence.  Privacy and fear affect adoption: Privacy concerns and event-related fear influenced adoption through social influence mediation. | Context limited to Saudi Arabia (mandatory adoption)  Cross-sectional data, not longitudinal  Limited cultural and global comparison  Lack of qualitative perspectives |
| [[15]](https://e-journal.unair.ac.id/JISEBI/article/view/41371) | Alviani R, Purwandari B, Eitiveni I, Purwaningsih M. Factors affecting adoption of telemedicine for virtual healthcare services in Indonesia. J Inf Syst Eng Bus Intell. 2023;9(1):47-69. doi:10.20473/jisebi.9.1.47-69 | Indonesia. | Adult individuals in Indonesia who were users or potential users of telemedicine services; reported age range 17–55 years. | Quantitative, cross-sectional design using UTAUT2 framework with added factors (e-health literacy, privacy concern, trust) | 144 respondents (69.2% female, mostly aged 17-25) | Online questionnaire (Google Form), distributed randomly among Indonesian adults, adapted from validated instruments, using 5-point Likert scale | Performance expectancy significantly predicts adoption: Users adopt telemedicine if they perceive it as useful and beneficial.  Effort expectancy influences intention: Ease of use drives intention to use telemedicine.  Social influence is significant: Opinions of relatives, friends and providers shape adoption.  E-health literacy strongly affects adoption: Higher literacy improves adoption, trust and performance expectancy.  Trust enhances behavioural intention: Confidence in platforms increases adoption.  Facilitating conditions not significant: Availability of infrastructure and resources does not strongly influence intention.  Price value not significant: Perceived cost-benefit does not significantly affect intention.  Privacy concern not significant: Worries about data confidentiality do not strongly influence intention.  Gender moderates adoption factors: Women are more influenced by effort expectancy, social influence, literacy and trust.  Age moderates adoption factors: Younger groups are more influenced by performance expectancy and trust. | Small sample size limits generalisation  Only users’ perspective considered  Limited factors studied |
| [[16]](https://bmchealthservres.biomedcentral.com/articles/10.1186/s12913-023-10368-z) | Anttila M, Koivisto J, Luoma ML, et al. How to adopt technologies in home care: a mixed methods study on user experiences and change of home care in Finland. BMC Health Serv Res. 2023;23:1342. doi:10.1186/s12913-023-10368-z | Finland – National governmental programme “Smart Ageing and Care at Home” (KATI), involving regional projects across Finland. | Home care professionals in Finland; the study also described perspectives of older people and informal caregivers. Adult professionals (≥18 years), Finnish-speaking/reading, able to provide informed consent. | Threefold mixed-methods design: (1) documented technologies piloted in the KATI programme; (2) focus-group interviews to explore requirements; (3) repeated surveys to assess changes in work and processes | 25 home care professionals in focus groups (female, aged 21-58, mean 39.7); 90 professionals in baseline survey; 70 in follow-up survey | Semi-structured focus groups (8 groups, grouped by technology type) via Microsoft Teams; baseline and follow-up Webropol team surveys; closed platform documentation of pilot technologies | Familiarity, commitment and benefit understanding required: Adoption depended on professionals, older people and caregivers being familiar with and committed to technology use, and perceiving clear benefits.  Perceived usefulness motivates adoption: Technology-enabled rehabilitation, independence, resource allocation, safety and social connection.  Ease of use improves adoption despite initial difficulties: Both professionals and older people faced early challenges but adapted over time.  Positive attitudes strengthen adoption: Acceptance and trust developed when technology was safe and automatic.  Negative attitudes hinder adoption: Suspicion, resistance and perceived lack of benefit reduced willingness to adopt.  Continuance intention uncertain: Professionals, older people and caregivers were unsure about future continuation due to cost and availability.  Technology provides new skills and insights: Professionals, older people and caregivers gained technical, guiding and coping skills.  Technology enhances information provision: Provided real-time data on wellbeing, care practices and new possibilities.  Technology changes procedures: Created new work processes, improved coping and allowed procedure modification.  Technology reduces unnecessary procedures: Reduced need for physical visits and repetitive checks.  Technology motivates through aid and independence: Professionals, older people and caregivers were motivated by improved independence, safety and resource efficiency. | Wide variation in technologies and pilots complicates consistency  Interview and survey questions not pilot tested  Focus mainly on professionals’ perspectives; limited direct input from older people and caregivers  Difficulties reaching participant  Data analysed as a whole, not by individual technologies |
| [[17]](https://diabetes.jmir.org/2024/1/e56756) | Arueyingho O, Aprioku JS, Marshall P, O’Kane AA. Insights into sociodemographic influences on type 2 diabetes care and opportunities for digital health promotion in Port Harcourt, Nigeria: quantitative study. JMIR Diabetes. 2024;9:e56756. doi:10.2196/56756 | Nigeria – Port Harcourt. | People diagnosed with type 2 diabetes residing in Port Harcourt, Nigeria; eligibility included diagnosis for >6 months, age >18 years, and ability to provide informed consent. | Cross-sectional quantitative study conducted from February-April 2023 in Port Harcourt, Nigeria, exploring sociodemographic influences, self-care, collaborative care and digital health opportunities | 110 participants with T2D (55 men, 55 women, aged ≥18) | Structured questionnaire, informed by prior WhatsApp interviews with patients, pharmacists and caregivers; analysed with SPSS and JASP | High literacy among participants: Most respondents had tertiary education, creating opportunities for digital health education and co-design.  Hybrid management practices: Patients combined prescribed medications with traditional medicines, diet, exercise and spirituality.  Low technology adoption beyond glucometers: Familiarity was limited, with most only using glucometers.  Preference for physical contact: In-person care and information were strongly preferred over digital channels.  Cost, ignorance and religious beliefs as barriers: These hindered the adoption of digital tools like glucometers and apps.  High mobile phone ownership: Nearly all participants owned phones, mostly Android with internet access, offering an opportunity for m-health.  WhatsApp and Facebook as dominant platforms: Widely used, though not fully leveraged for health communication.  Word of mouth as main health information source: Community and interpersonal networks remain trusted channels.  Opportunities for context-specific interventions: Local design and integration into routine practices could enhance adoption. | Did not measure household size or purchasing power  Questionnaire lacked questions on alternative communication channels or digital health needs  Sample skewed toward educated, higher-income respondents  Recruitment bias from in-person and online sampling  WhatsApp automation recruitment was costly and limited |
| [[18]](https://sigmapubs.onlinelibrary.wiley.com/doi/10.1111/jnu.12619) | Aure CF, Kluge A, Moen A. Older adults’ engagement in technology-mediated self-monitoring of diet: a mixed-method study. J Nurs Scholarsh. 2021;53(1):25-34. doi:10.1111/jnu.12619 | Norway – Participants recruited from four municipalities. | Older adults living at home, including individuals receiving home care services and individuals connected to a senior center; reported age range 68–95 years. | Combined descriptive analysis of log data with qualitative interviews to explore older adults’ engagement in self-monitoring diet using Appetitus | 25 older adults (68-95 years), recruited from home care services and senior centres | Automatic log data from Appetitus app; baseline and follow-up interviews on user experience; structured demographic interviews; support provided by healthcare professionals and senior volunteers | High regular use among participants: Most older adults used the nutrition app consistently over the 8-week trial.  Personal interest and commitment facilitated adoption: Motivation was driven by nutrition interest and commitment to the project.  Poor health and lack of relevance hindered use: Frailty and low perceived relevance limited adoption.  App was easy to use for inexperienced users: Even those without prior technology experience managed to use the app.  Daily monitoring increased diet awareness: Regular users reported greater awareness of food intake.  Recording perceived as burdensome by some: Continuous logging felt restrictive or tiring for certain participants.  Altruism-motivated engagement: Desire to contribute to research encouraged participation.  Technology boosted confidence and skills: Older adults gained digital confidence and expanded tech use.  Support from professionals and relatives encouraged use: Guidance from healthcare professionals and family engagement facilitated sustained adoption. | Small, selective sample with good cognitive functioning  Cognitive health may influence uptake  Generalisability limited |
| [[19]](https://onlinelibrary.wiley.com/doi/10.1111/jep.13923) | Bagot KL, Bladin CF, Vu M, Bernard S, Smith K, Hocking G, et al. Factors influencing the successful implementation of a novel digital health application to streamline multidisciplinary communication across multiple organisations for emergency care. J Eval Clin Pract. 2024;30(2):184-98. doi:10.1111/jep.13923 | Australia – Rural hospitals and ambulance branches in Victoria. | Ambulance paramedics and hospital clinicians involved in the care of people with suspected stroke and cardiac conditions. | Real-world feasibility pilot and implementation study combining quantitative surveys, app usage logs and qualitative open-text analysis | 172 clinicians (82 paramedics, 90 hospital-based staff). Median age of paramedics: 44; hospital staff: 37 | Surveys: Organisational Readiness for Implementing Change (ORIC), UTAUT (acceptance and intention), Self-Determination Theory (SDT). Usage logs from the app dashboard. Qualitative directed content analysis of perceived benefits and barriers | Habit is the strongest predictor: Regular use habits most strongly influenced intentions to adopt Pulsara™.  Effort expectancy predicts adoption: Ease of use significantly increased clinicians’ intentions to adopt.  Performance expectancy influences adoption: Belief in usefulness for patient care and workflow efficiency drove adoption.  Organisational readiness supports implementation: Change-ready organisations were more likely to succeed with adoption.  Facilitating conditions affect usability: Infrastructure and familiar equipment eased adoption, but connectivity issues created barriers.  Password and login issues hindered use: Users faced access problems that reduced ease of adoption.  Photo/image quality issues limited reliability: Poor quality in moving vehicles affected adoption confidence.  Training and familiarity needed: Lack of digital literacy and insufficient training reduced adoption.  Benefits included improved communication and faster care: Users reported timelier, more standardised and accessible information.  Concerns about duplication of work: Retaining usual communication systems during trial created inefficiencies. | Measured intentions, not actual individual-level use  Generic log-ins limited tracking of individual adoption  Generalisability limited to rural settings  Ongoing sustainability and costs not evaluated  Need for integration with electronic health records |
| [[20]](https://pmc.ncbi.nlm.nih.gov/articles/PMC10990475/) | Baik D, Reeder B, Coats H, Baker C, Jankowski C. Perceptions and attitudes toward a proposed digital health physical activity program among older family caregivers of persons with heart failure: a qualitative study. Inform Health Soc Care. 2023;48(3):239-51. doi:10.1080/17538157.2023.2227704 | United States – Outpatient Advanced Heart Failure Clinic, University of Colorado Hospital, Colorado. | Older family caregivers of persons with heart failure; inclusion criteria included age ≥65 years, co-residing with a family member with heart failure, providing unpaid care, English-speaking, and able to provide informed consent. | Qualitative descriptive study guided by an adapted UTAUT explored perceptions of older family caregivers (FCGs) on video-conferencing, fitness tracker and text messaging for a digital health physical activity programme | 13 caregivers (≥65 years; mean age 70; 85% female, mostly spouses) | Conducted by phone (50-60 minutes), audio-recorded and transcribed; analysed with directed content analysis in Dedoose | Video-conferencing perceived as easy and familiar: Caregivers found Zoom/Skype easy due to COVID-19 use for family, church and medical visits.  Setup and handling difficulties created barriers: Some struggled with setup, functionality and camera positioning.  Fitness trackers viewed as easy and useful: Most participants reported trackers were user-friendly and motivating.  Tracker burden reduced adoption: Wearing devices constantly and frequent notifications felt annoying.  Text messages divisive: Some saw them as useful reminders, others as annoying or unnecessary.  Usefulness of video-coaching reinforced adoption: Coaching added accountability, motivation and professional validation.  Facilitating conditions support use: Fitting with daily physical activity lifestyle, internal motivation, and available technical support encouraged adoption.  Age and digital skills moderated adoption: Some felt too old for new tech, while others with prior computer experience were confident.  Positive patient experience encouraged adoption: When HF patients had good tech experiences, caregivers were more willing to try.  Internet connectivity as a barrier: Poor internet connections made video sessions frustrating and unreliable. | Conducted at a single medical centre, limited generalisability  Sample lacked diversity (mostly White)  Phone interviews limited richness of data  Social influence excluded from adapted UTAUT but emerged indirectly  Participants may differ from those who declined participation |
| [[21]](https://www.jmir.org/2020/9/e15307/) | Burns K, Nicholas R, Beatson A, Chamorro-Koc M, Blackler A, Gottlieb U. Identifying mobile health engagement stages: interviews and observations for developing brief message content. J Med Internet Res. 2020;22(9):e15307. doi:10.2196/15307 | Australia – Queensland University of Technology (Brisbane, QLD); participants recruited in a large metropolitan city in Australia and via the Global Corporate Challenge workplace pedometer competition. | Adults aged >18 years who had used an mHealth technology for at least two weeks prior to participation (pregnancy apps excluded); participants were able to communicate effectively in English and provided informed consent. | Qualitative study (interviews and observations) used O’Brien and Toms’ engagement framework to explore m-health engagement stages and generate message content | 19 participants (individual use, age 18-49) and 25 participants (group use, age 18-69, ≥70 years) | In-depth confidential interviews and structured face-to-face observations over 3 sessions; thematic analysis in Atlas.ti; triangulated with observation data | Technology usability supports adoption: Ease of use, wearability, portability, convenience and seamless syncing motivate engagement, while usability failures lead to disengagement.  Technology features drive motivation: Health data collection, monitoring, dashboard tracking, gamification and statistics encourage sustained engagement and re-engagement.  Technology aesthetics influence re-engagement: Attractive interface, ergonomic design and unobtrusiveness matter when users consider returning to or upgrading devices.  Use motivations to sustain engagement: Intrinsic motivators (fitness, wellness, stress reduction) and extrinsic motivators (team, competition, obligations) encourage use.  Health awareness promotes accountability: M-health devices help users recognise health behaviours, evaluate performance and increase accountability.  Goal setting enhances engagement: Goals for exercise, nutrition, sleep, weight and heart rate motivate sustained use, self-management and re-engagement.  Social support fosters sustained engagement: Customer support, peer support, team interaction and community enhance motivation and uptake.  Interruptions cause disengagement: Family, work, illness, short battery life and device failures disrupt engagement, leading to limited use or self-management. | Small, exploratory sample in a single country  Limited generalisability across cultures and contexts  More quantitative and experimental work needed |
| [[22]](https://journals.plos.org/plosone/article?id=10.1371/journal.pone.0303302) | Carrera A, Lettieri E, Lietti G, Martignoni S, Sgarbossa C, Cafazzo J. Therapies go digital. What drives physicians’ acceptance? PLoS One. 2024;19(5):e0303302. doi:10.1371/journal.pone.0303302 | Italy – Survey conducted in collaboration with Italy’s leading diabetes scientific association and distributed to members of the Italian scientific society in diabetology. | Physicians and clinicians in diabetology targeted via the leading Italian scientific society in diabetology. | Cross-sectional quantitative survey combined the Technology Acceptance Model (TAM) and Institutional Theory to examine rational and institutional determinants of physicians’ acceptance of digital therapeutics (DTx) | 107 physicians (members of the Italian scientific society in diabetology) | Web-based self-administered questionnaire (61 items, 5-point Likert scale) distributed via Italian diabetes scientific society; analysed with structural equation modelling (STATA 17) | Perceived usefulness strongly predicts intention to use: Physicians are more willing to adopt DTx when they perceive them as beneficial for patient care and work effectiveness.  Ease of use enhances perceived usefulness: Simplicity and intuitiveness of DTx increase perceptions of their effectiveness.  Hospital culture supports ease of use: Organisational openness to innovation makes physicians view DTx as less complex.  Regulations from scientific societies foster trust and usefulness: Structured regulatory frameworks from associations enhance perceived benefits of DTx.  Colleagues’ opinions enhance perceived usefulness: Physicians’ views are influenced by peers and respected colleagues in scientific associations.  Control variables affect intention to use: Age, professional role, sense of belonging to scientific societies and risk propensity significantly shape acceptance. | Limited to one specialty and country  Quantitative only; qualitative could add depth  Generalisability limited by sample size  Focused only on physician-driven distribution model |
| [[23]](https://www.frontiersin.org/journals/public-health/articles/10.3389/fpubh.2024.1449594/full) | Wu C, Lim GG. Investigating older adults users’ willingness to adopt wearable devices by integrating the technology acceptance model (UTAUT2) and the Technology Readiness Index theory. Front Public Health. 2024;12:1449594. doi:10.3389/fpubh.2024.1449594 | China – Including rural China. | Older adult users and consumers aged over 60 years. | Cross-sectional quantitative study (SEM-based) integrated UTAUT2 with the Technology Readiness Index (TRI) and digital health literacy (DHL) to assess the adoption of smart wearables by older adults in China | 389 older adults (aged ≥60, diverse education and income levels) | Online snowball distribution and paper questionnaires in public areas; oral assistance for those with impairments; SPSS and AMOS used for SEM and mediation/moderation analysis | Performance expectancy supports willingness: Older adults are more likely to adopt wearable devices when they believe these devices improve daily life and health management.  Effort expectancy encourages adoption: Ease of learning and operating devices increases willingness to adopt.  Social influence fosters willingness: Family, peers and social networks encourage device use.  Facilitating conditions increase adoption: Availability of resources, knowledge and technical support enhances acceptance.  Hedonic motivation drives intention: Enjoyment and pleasure in using wearables boost willingness.  Price value influences willingness: Cost-effectiveness is a critical determinant for older adults.  DHL enhances adoption: Higher DHL improves understanding and willingness to use devices.  Optimism motivates adoption via expectancy: Positive attitudes boost perceived usefulness and ease of use.  Innovativeness boosts acceptance: Willingness to try new tech enhances both usefulness and ease of use perceptions.  Discomfort hinders adoption: Anxiety and unease reduce both usefulness and ease of use perceptions.  Insecurity lowers adoption: Privacy and safety concerns reduce ease of use perceptions.  DHL moderates expectancy effects: High DHL reduces dependence on performance and effort expectancy. | Sample limited to China; cultural differences not captured  Omitted some UTAUT2 moderators (age, gender, habit)  Other relevant variables not tested  Cross-sectional quantitative only; need mixed methods |
| [[24]](https://www.mdpi.com/2076-3425/15/5/464) | Chen ML, Hsieh KL, Hsieh SL, Hsieh MC, Lo DCT, Hsu JL. Barriers to using mobile app-based cognitive testing in older adults with probable Alzheimer’s disease: a qualitative study. Brain Sci. 2025;15(5):464. doi:10.3390/brainsci15050464 | Taiwan – Outpatient clinic at a hospital neurological department. | Older adults with probable Alzheimer’s disease and their family caregivers; eligibility and exclusion characteristics were specified for patients and caregivers, including cognitive status, education level, language fluency, and prior smartphone use. | Qualitative study explored barriers to using mobile app-based cognitive assessment (SymptomTrace) among older adults with probable Alzheimer’s disease and their caregivers in Taiwan | 32 older adults with probable Alzheimer’s disease; 19 family caregivers | Participants used SymptomTrace every 2 weeks for 12 months (24 sessions). Afterwards, an acceptability questionnaire (usefulness, ease, satisfaction, willingness) and semi-structured phone interviews were conducted; thematic analysis was applied | Family support barrier: Older adults with probable Alzheimer’s disease require reminders and assistance from family members to use mobile app-based cognitive assessments.  Health-related barriers: Memory decline, emotional distress and physical health issues hinder engagement with digital cognitive testing.  App-related barriers: Small fonts, unclear images, login issues, crashes and a non-intuitive interface make app usage difficult for older adults with Alzheimer’s disease. | Age range limited (64-75 years)  Did not assess digital literacy  Sample mostly living with family  Limited transferability beyond Taiwan outpatient setting |
| [[25]](https://journals.sagepub.com/doi/10.1177/20552076231173560) | Cheraghi-Sohi S, Davies K, Gordon L, Jones H, Sanders C, Ong BN. A study to explore the usefulness of a mobile health application to support people with mild cognitive and/or communication impairment due to dementia and their carers. Digit Health. 2023;9:20552076231173560. doi:10.1177/20552076231173560 | United Kingdom – Study team based in Manchester with a partner app provider in London; participants recruited via care organisations, community organisations, and the Join Dementia Research website. | People living with dementia with mild cognitive and/or communication impairment and their carers, including formal and informal carers. | Longitudinal qualitative study evaluated the “Hear Me Now” (HMN) app, originally co-designed with people with learning disabilities, adapted for people with dementia and carers; used the domestication of technology model (DTM) | 13 people with mild cognitive/communication impairment due to dementia and 12 carers (formal and informal) | Three phases: recruitment and initial interviews; training carers and people with mild cognitive/communication impairment; 3-month home use with follow-up interviews and focus groups. Remote methods adopted due to COVID-19 | Altruistic motivation drives participation: Some participants joined the study to help future dementia research, showing research-related motivations beyond personal benefit.  Memory support motivation: Carers expected the app to help with memory problems and communication in daily life.  Future-oriented motivation: Some saw potential for long-term communication support and independence as dementia progresses.  Organisational aid motivation: Participants hoped the app could bring order to daily tasks and caregiving routines.  Technology anxiety barrier: Some participants expressed fear and anxiety about learning new technology, leading to dropout.  Preference for familiar low-tech alternatives: Some users preferred calendars, post-its or existing devices over the app, seeing duplication of effort.  Carer burden as barrier: Carers felt the time and effort needed to use the app conflicted with daily caregiving responsibilities.  Progression of dementia as a barrier: Declining memory and health reduced the ability to engage with the app over time.  Design and functionality issues: Lack of cross-linking features, speed and diary limitations reduced usefulness and hindered adoption.  Value for independence and personhood: For a few, the app helped maintain identity, daily activities and future-proofing information. | COVID-19 disrupted participatory co-design process  Remote data collection limited observational insights  Some dropouts due to frustration or limited perceived usefulness  Small sample, heterogeneous needs, limited generalisability |
| [[26]](https://journals.sagepub.com/doi/10.1177/21582440241228639) | Lu C-C, Tsai-Lin T-F. Are older adults special in adopting public eHealth service initiatives? The modified model of UTAUT. SAGE Open. 2024;14(1). doi:10.1177/21582440241228639 | Taiwan – Hsinchu City, including community care centres and the public eHealth service context of the Hsinchu City Government. | Older adults in Hsinchu City with experience using the “Babybot” self-monitoring telecare device at community care stations. | Cross-sectional quantitative study applied modified UTAUT to older adults’ adoption of a public e-health service (Babybot self-monitoring telecare device) | 510 older adults (Hsinchu City, Taiwan; 80.6% female, mean age mostly 65-84) | Self-administered questionnaire at 28 community care stations; items measured social influence (relationship and reference), facilitating conditions (government support and health benefits), performance expectancy, effort expectancy and behavioural intention; pretested and supported by local volunteers | Social influence supports adoption: Family, peers and socially important people significantly shape older adults’ intention to use public e-health services.  Facilitating conditions are critical: Government support and perceived health benefits strongly predict intention to adopt public e-health services.  Performance expectancy moderates influence of support: Performance expectancy strengthens the effect of government support on intention to adopt, though not consistently across all factors.  Effort expectancy moderates adoption: Effort expectancy strengthens the effect of government support and relationship-based social influence on intention to adopt.  Government-provided trust and design boost adoption: Trustworthiness, user-friendly interfaces and policy initiatives make older adults more secure and confident in e-health use.  Cultural and social acceptance enhance uptake: Adoption grows when e-health is perceived as beneficial, popular and socially or culturally endorsed.  Volunteers and social encouragement reduce barriers: Volunteer programmes and encouragement from close acquaintances increase adoption confidence. | Findings limited to one city and public e-health context  Only local government policy examined  Did not consider intrinsic motivation factors  Volunteer role not fully assessed |
| [[27]](https://journals.sagepub.com/doi/10.1177/11795972241277292) | Das Gupta A. Conceptualizing patient as an organization with the adoption of digital health. Biomed Eng Comput Biol. 2024;15:11795972241277292. doi:10.1177/11795972241277292 | India – Management Development Institute Gurgaon, Gurgaon, Haryana. | Respondents described as mobile device users; respondents included males and females across age groups, with varying education and income levels, and reported digital health use and health conditions. | Cross-sectional quantitative survey (PLS-SEM) extended TAM with the Health Belief Model (HBM) and UTAUT constructs to examine the adoption intention of digital health, conceptualising the patient as an “organisation” | 164 respondents (49% male, 51% female, balanced across age groups; around 30% had diabetes, 20% hypertension, 10% heart disease) | Randomised online survey among mobile device users; SmartPLS used for structural equation modelling and hypothesis testing | Perceived usefulness drives adoption: Perceived usefulness significantly impacts intention to adopt digital health.  Customer innovativeness motivates adoption: Willingness to try new technologies increases adoption.  Health beliefs enhance perceived usefulness: Healthy lifestyle beliefs increase perception of digital health tools’ value.  Perceived ease of use improves usefulness and adoption: Ease of use strongly enhances perceived usefulness and indirectly adoption.  Reference group influence boosts adoption: Peer and social group opinions strongly predict adoption.  System capability enhances ease of use and adoption: Strong frameworks and adaptability increase ease and adoption.  Privacy and data security are not decisive: Privacy protection has no significant effect on adoption.  Online healthcare resources reduce usability: Online resources negatively impact ease of use.  User guidance is ineffective: User guidance has no significant influence on adoption.  Patient empowerment conceptualisation: Patients as organisations produce and manage health data, increasing engagement and autonomy. | Sample size relatively small  Limited digital health experience among respondents  Did not include broader intrinsic or social factors  Findings may not generalise across contexts |
| [[28]](https://www.researchgate.net/publication/338187344_Consumer_adoption_of_smartphone_fitness_apps_an_extended_UTAUT2_perspective) | Dhiman N, Arora N, Dogra N, Gupta A. Consumer adoption of smartphone fitness apps: an extended UTAUT2 perspective. J Indian Bus Res. 2020;12(3):363-88. doi:10.1108/JIBR-05-2018-0158 | India – Participants recruited across three states in North India via social media platforms and email invitations. | Individuals who had acquired membership of a fitness club or gym and had used smartphone fitness applications. | Cross-sectional quantitative survey (PLS-SEM) extended UTAUT2 by adding self-efficacy and personal innovativeness to examine determinants of adoption of fitness apps in India | 324 valid respondents (54% male, 46% female; 80% aged 20-40; all were gym or fitness club members) | Purposive sampling via gyms or fitness clubs across 3 Indian states; questionnaire link shared on social media and email; analysed with PLS-SEM (R and plspm package) | Effort expectancy supports adoption: Ease of using smartphone fitness apps significantly increases intention to adopt.  Social influence shapes intention: Opinions of family, friends and colleagues strongly affect adoption decisions.  Price value motivates adoption: Perceived financial benefits versus costs positively influence adoption.  Habit drives continued use: Past use habits strongly influence intention to continue adoption.  Personal innovativeness is the strongest predictor: Willingness to experiment with new technologies most strongly predicts adoption.  Self-efficacy indirectly supports adoption: Confidence enhances effort expectancy, which influences adoption.  Performance expectancy not significant: Contrary to TAM or UTAUT assumptions, perceived usefulness did not influence intention.  Facilitating conditions not significant: Availability of resources and support did not impact adoption.  Hedonic motivation not significant: Fun and enjoyment did not affect initial adoption intentions. | Conducted only in India; limited generalisability  Focused only on adoption, not post-adoption use  Excluded other potential variables (e.g. anxiety, credibility, UI)  Did not test moderating effects of age, gender or experience |
| [[29]](https://pharmacia.pensoft.net/article/108957/) | Dimitrova M, Dimitrov R, Ahchiyska K, Nikolaeva M, Ganova M, Petrova G. Digital health in Bulgaria: imagination or possible reality? Pharmacia. 2023;70(3):649-55. doi:10.3897/pharmacia.70.e108957 | Bulgaria. | Stakeholders from the health sector in Bulgaria, including patients, healthcare professionals, regulatory authorities, industry representatives, and medical and pharmacy students. | Two-step approach: (1) policy and regulatory review of Bulgaria’s digital health ecosystem; (2) stakeholder questionnaire on barriers, opportunities and benefits | 380 stakeholders (280 patients, 22 pharma industry, 12 tech industry, 9 regulators, 41 healthcare professionals, 49 students) | Reviewed national health strategies, regulations and NGO initiatives; online survey with 21 questions (16 closed, 5 open) distributed to stakeholders (June-December 2021); descriptive statistics in Excel | Regulatory barriers slow adoption: Lack of clear framework, telemedicine rules and data usage guidelines delay digital transformation.  Infrastructure limitations hinder access: Poor internet connectivity, outdated systems and rural inequalities reduce effectiveness.  Funding constraints restrict implementation: Budget limitations slow investment in robust digital health systems.  Data privacy and security concerns: Strict regulations slow adoption despite the importance of trust and innovation.  Cultural resistance limits digital literacy: Low digital skills and resistance to change block effective adoption.  Organisational fragmentation prevents interoperability: Lack of coordination and standardised practices across healthcare providers slows digitalisation.  Need for digital literacy in the healthcare workforce: Training future professionals and policymakers is essential.  Public awareness and information campaigns required: Citizens need awareness initiatives before adoption.  Perceived benefits encourage adoption: Stakeholders believe digitalisation improves access, prevention, transparency and efficiency. | National-level focus may not reflect local variations  Stakeholder survey limited by voluntary participation  Early stage of policy implementation limits conclusions |
| [[30]](https://www.frontiersin.org/journals/public-health/articles/10.3389/fpubh.2023.1325031/full) | Liu D, Son S, Cao J. The determinants of public acceptance of telemedicine apps: an innovation diffusion perspective. Front Public Health. 2023;11:1325031. doi:10.3389/fpubh.2023.1325031 | China – Beijing; survey conducted at four transportation hubs (Wangjing West, Dongzhimen, Beijing South Station, and Beijing Zoo). | Consumers and residents in Beijing who had previously used telemedicine applications. | Cross-sectional quantitative survey (VB-SEM, PLS-SEM) integrated diffusion of innovation (DOI) theory and perceived value theory (PVT) to assess public acceptance of telemedicine apps in Beijing | 387 residents (68.5% female, 78% aged 18-39, 86% higher education, mostly income 5,000-15,000 RMB/month) | Conducted at 4 Beijing transportation hubs (July 2023); paper and QR code online surveys; data analysed using VB-SEM or PLS-SEM | Relative advantage supports adoption: Time-saving, efficiency and usefulness of telemedicine apps increase perceived value and acceptance.  Compatibility fosters positive perception: Alignment with users’ healthcare practices, beliefs and values enhances perceived value and acceptance.  Complexity hinders adoption: Difficulty of use reduces perceived value and public acceptance.  Trialability increases perceived value: Opportunities to try out apps reduce uncertainty and enhance acceptance.  Observability encourages adoption: Visibility of positive outcomes builds trust and motivates use.  Perceived value predicts acceptance: Perceived value mediates the relationship between innovation attributes and public acceptance.  Demographic factors not significant: Gender, age, education and income did not significantly influence public acceptance.  Mediating role of perceived value: Relative advantage, compatibility, complexity, trialability and observability affect acceptance via perceived value. | Sample limited to Beijing, lacks wider representation  Cross-sectional design limits understanding of long-term adoption  Younger, educated bias in sample  Narrow theoretical scope (only DOI and PVT)  Excluded key variables like privacy or security concerns |
| [[31]](https://www.jmir.org/2021/2/e24767/) | Elkefi S, Yu Z, Asan O. Online medical record nonuse among patients: data analysis study of the 2019 Health Information National Trends Survey. J Med Internet Res. 2021;23(2):e24767. doi:10.2196/24767 | United States – Data from a nationally representative survey of the US noninstitutionalized adult population. | Adults included in the analysis who had experienced an outpatient visit within the previous 12 months and answered the online behavior question regarding use of online medical records. | Cross-sectional quantitative survey secondary analysis of the 2019 Health Information National Trends Survey (HINTS 5, Cycle 3) focusing on reasons for non-use of online medical records | 4,328 US adults (57.7% female, 67.6% aged >50, 16% with cancer) | Self-administered survey with online medical record use questions and demographic variables; descriptive and chi-square analysis in R | High non-use rates: More than half of patients surveyed did not use online medical records.  Age barrier: Older adults (≥65) were less likely to use online medical records.  Race/ethnicity barrier: Black and Hispanic patients had significantly higher non-use rates.  Cancer diagnosis facilitates use: Cancer patients were more likely to use online medical records.  Preference for in-person interaction: Most non-users preferred speaking directly to their healthcare providers.  Perceived no need: Many respondents felt they did not need online records.  Privacy concerns: Minorities and older adults expressed greater concern about privacy.  Low digital comfort: Older adults felt uncomfortable with technology use.  Login and access problems: Patients struggled with logging in and managing multiple records.  Demographic predictors: Female patients were more likely to use online records; older, Black and Hispanic patients were less likely. | Cross-sectional, no causality  Low survey response rate (20-30%) may bias  Limited variables; education not examined  Pre-pandemic data; context shifted since COVID-19 |
| [[32]](https://journals.sagepub.com/doi/10.1177/20552076251334043) | Goldberg DG, Soylu TG, Hoffman CF, Kishton RE, Cronholm PF. Clinicians’ perspectives on the adoption and implementation of EMR-integrated clinical decision support tools in primary care. Digit Health. 2025;11:20552076251334043. doi:10.1177/20552076251334043 | United States – Primary care practice settings across the United States. | Primary care clinicians, including physicians, nurse practitioners, and physician assistants with prescribing authority, working in primary care settings; participants reported using clinical decision support tools for patient care. | In-depth interviews explored facilitators/barriers to adoption of clinical decision support (CDS) tools in primary care, using UTAUT and diffusion of innovation (DOI) as theoretical frameworks | 32 primary care clinicians (24 physicians, 3 nurse practitioners, 5 physician assistants) | Interviews (2021-2023) using UTAUT/DOI constructs; thematic coding with NVivo; multidisciplinary team reviewed codes; saturation reached | EMR inefficiencies hinder adoption: Clinicians cited difficulties with usability, interoperability and “click burden.”  Resistance to change: Long-practising clinicians resisted shifting from traditional decision-making to CDS tools.  Time and effort barriers: High workload and short patient visits left little time for CDS tool use.  System incompatibility: Lack of seamless EMR integration discouraged use.  Organisational approval requirement: Adoption depended on hospital or committee approval, limiting autonomy.  Need for champions and leadership support: Organisational champions encouraged adoption and peer uptake.  Technical support and training facilitators: Hands-on support, guides and training sessions reduced adoption barriers.  Evidence-based validation required: Clinicians demanded strong clinical evidence of effectiveness before adoption.  User-friendliness critical: Tools needed simple, efficient and minimal-click designs to be practical.  Ethical and legal concerns: Clinicians feared bias, malpractice liability and over-reliance on CDS tools.  Preferred gradual implementation: Pilot programmes and staged rollouts were seen as more successful. | Qualitative, exploratory; limited generalisability  Recruitment bias possible; time constraints excluded some  Geographic and setting imbalance |
| [[33]](https://journals.healio.com/doi/10.3928/00220124-20241107-03?url_ver=Z39.88-2003&rfr_id=ori:rid:crossref.org&rfr_dat=cr_pub%20%200pubmed) | Han J, Kim M, Ryu KH, Shin K. Acceptance of digital health care technology and the role of nursing education. J Contin Educ Nurs. 2024;55(12):575-87. doi:10.3928/00220124-20241107-03 | South Korea – Specialized cancer medical institution. | Nurses working in a specialized cancer medical institution in South Korea. | Cross-sectional quantitative study (SEM-based) integrated UTAUT, DOI and individual factors (self-efficacy, innovativeness) to examine nurses’ acceptance of digital healthcare technology in a cancer specialty hospital | 237 nurses (92.8% female, majority in 20s-30s, with 1-5 years of tenure) | Printed surveys distributed in March 2024; analysed using SEM (Mplus 8.4, SPSS 23); mediation analysis with bootstrapping | Performance expectancy drives intention: Nurses’ belief that digital healthcare enhances job performance strongly predicts acceptance.  Facilitating conditions support acceptance: Organisational and technical support significantly increase intention to adopt.  Innovativeness predicts adoption: Willingness to try new technologies has a direct positive effect on intention.  Self-efficacy strengthens mediators: While not directly influencing intention, self-efficacy enhances effort expectancy, social influence and facilitating conditions.  Relative advantage improves mediators: Perceived superiority of digital healthcare increases performance expectancy, social influence and facilitating conditions.  Trialability fosters positive expectations: Opportunities to try digital healthcare tools enhance performance expectancy and facilitating conditions.  Complexity hinders adoption: Perceived difficulty negatively affects performance expectancy, effort expectancy and facilitating conditions, reducing acceptance.  Mediating role of expectancy and conditions: Performance expectancy and facilitating conditions mediate effects of relative advantage, trialability, complexity and self-efficacy on acceptance intention. | Conducted in one cancer specialty hospital, limiting generalisability  Limited variable scope  Did not evaluate technical aspects of specific tools |
| [[34]](https://bmcnurs.biomedcentral.com/articles/10.1186/s12912-025-03108-7) | He Y, Fan G, Fan G, et al. Exploring nurse and patient perspectives on WeChat-based prenatal education in Chinese public hospitals: a qualitative inquiry. BMC Nurs. 2025;24:459. doi:10.1186/s12912-025-03108-7 | China – Four public hospitals in Shanxi Province. | Obstetric nurses in public hospitals involved in WeChat-based prenatal education and pregnant women aged ≥18 years who used WeChat to receive prenatal education or communicate with obstetric nurses. | Qualitative exploratory study examined how obstetric nurses and pregnant women perceive and engage with WeChat-based prenatal education in public hospitals in Shanxi, China | 17 obstetric nurses; 8 pregnant women (nurses aged 25-34; pregnant women aged 23-35) | Conducted face to face, 30-45 minutes each; explored usefulness, ease of use, digital literacy, workload and family influence; thematic analysis applied | WeChat extends nurses’ professional reach: Nurses viewed WeChat as useful for frequent micro-interactions and continuous support beyond clinic hours.  Blurred boundaries and emotional strain: Nurses felt constant availability pressures, creating role ambiguity and emotional burden.  Balancing high-tech with high-touch: Nurses feared that digital text-based exchanges diluted empathy compared to face-to-face care.  Organisational gaps hinder adoption: Lack of policies, guidelines and formal training forced nurses to improvise digital communication practices.  Concerns over content quality and privacy: Nurses stressed verifying information and managing patient privacy on WeChat.  Cultural and family influences: Extended family members often participated in chats, sometimes conflicting with medical advice.  Digital literacy gaps among pregnant women: Tech-savvy mothers embraced WeChat, while less skilled women struggled with basic features.  Visual and tailored content helps engagement: Infographics, colour-coding and voice messages improved comprehension.  Expanded access and reduced costs: Pregnant women valued WeChat for saving travel time and wages.  Generational resistance and digital divide: Older or less educated women felt hesitant, embarrassed or marginalised.  Shared trust in nurse-vetted content: Both nurses and patients agreed that hospital-approved content enhanced trust.  Response-time expectations diverge: Patients expected fast replies, while nurses struggled with workload.  Need for structured institutional support: Both groups recommended digital office hours, content libraries and training to improve sustainability. | Conducted in one province (Shanxi), limited generalisability  Under-representation of older or less digitally literate women  Possible authority bias in recruitment  Cross-sectional only; lacks longitudinal perspective |
| [[35]](https://dl.acm.org/doi/10.1145/3635472) | Jardine J, Nadal C, Robinson S, Enrique A, Hanratty M, Doherty G. Between rhetoric and reality: real-world barriers to uptake and early engagement in digital mental health interventions. ACM Trans Comput Hum Interact. 2024;31(2):27. doi:10.1145/3635472 | United Kingdom and United States – Study sites included an NHS health service site and a UK university, and a US university. | Adults aged >18 years who accessed SilverCloud via a self-referral website and provided informed consent. | Cross-sectional observational study using mixed methods combining quantitative survey and qualitative semi-structured interviews to examine barriers to uptake and early engagement with a digital mental health intervention. | 205 adults for questionnaire (75.6% female; majority aged 18–24 years; mostly White; 64.4% college/university educated); 20 adults for interviews (70% female; age range 18–64 years; mostly White; 55% college/university educated) | Quantitative: Online questionnaire administered to adults accessing the SilverCloud self-referral website. Qualitative: Semi-structured interviews conducted with a subset of survey respondents to explore experiences, perceptions, expectations, and barriers. | Uncertainty about usefulness and usability: Many participants hesitated to sign up because they were unsure if the digital mental health intervention would be useful or easy to use.  Forgetting and lack of time: Early engagement was hindered when participants forgot about the programme or could not find time to use it.  Need for human connection: Users expressed that human contact was still essential alongside digital solutions.  Impact of self-stigma on help-seeking: Stigma prevented individuals from engaging with digital interventions.  Lack of knowledge: Limited understanding of digital mental health interventions and therapy reduced adoption.  Desire for personally relevant care: Participants wanted interventions tailored to their personal context.  Fluctuating nature of mental health: Ongoing, changing mental health needs shaped adoption decisions. | Sample skewed toward white, educated women from specific services in the UK and the United States  Only early-stage engagement explored  Possible self-selection bias  Two specific interventions only |
| [[36]](https://www.mdpi.com/2227-9032/8/4/494) | Jeng MY, Yeh TM, Pai FY. Analyzing older adults’ perceived values of using smart bracelets by means–end chain. Healthcare (Basel). 2020;8(4):494. doi:10.3390/healthcare8040494 | Taiwan – Senior citizens’ learning center in Taichung. | Older adults aged over 60 years participating at a senior citizens’ learning center. | Used means-end chain (MEC) and laddering interviews to identify attributes, consequences and values of smart bracelet use among older adults; constructed hierarchical value map (HVM) | 40 older adults wore Xiaomi smart bracelets for 3 months before interviews (60% male, 40% female; mostly 66-70 years old) | Participants used Xiaomi bracelets (steps, heart rate, sleep, sedentary reminder) for 3 months. One-to-one interviews (40-60 minutes) were conducted, guiding respondents from attributes → consequences → values; content analysis and coding into attributes/consequences/values | Essential device attributes: Older adults valued smart bracelets with safe use, real-time feedback, correct data, comfortable wear and clear screen.  Outcome benefits: Smart bracelets helped increase health awareness, satisfy curiosity, support relaxation and enhance technology understanding.  Value goals: Users aimed for healthier bodies, improved quality of life and a sense of social belonging.  Comfort and vision needs: Comfort and readability were critical due to physical and sensory changes in older age.  Motivation to change health behaviours: Real-time feedback and activity monitoring encouraged healthy behaviour.  Social and emotional benefits: Devices supported social connections, reduced loneliness and enhanced well-being.  Barriers to adoption: Few devices were tailored to older users’ needs; fast tech updates made adoption harder.  Privacy and security concerns: Older adults worried about sensitive health and social data being used or shared. | Devices not designed specifically for older adults  Market changes and rapid product replacement hinder adoption  Effectiveness of health monitoring apps not well proven  Small, exploratory sample; limited generalisability  Privacy and security concerns remain |
| [[37]](https://journals.sagepub.com/doi/10.1177/20552076221126034?url_ver=Z39.88-2003&rfr_id=ori:rid:crossref.org&rfr_dat=cr_pub%20%200pubmed) | Yang J, Luo B, Zhao C, Zhang H. Artificial intelligence healthcare service resources adoption by medical institutions based on TOE framework. Digit Health. 2022;8:20552076221126034. doi:10.1177/20552076221126034 | China – People’s Republic of China; study focused on integrated medical and healthcare organizations. | Expert participants from medical and healthcare institutions, including senior hospital leadership, clinical and administrative leaders, and managers of integrated medical and healthcare organizations. | Examined the adoption of AI healthcare service resources by hospitals in China using the technology-organisation-environment (TOE) framework | 290 hospital administrators (mid to senior level, across multiple provinces) | Online and paper-based distribution to hospital staff; PLS-SEM used for analysis; variables included technology (relative advantage, complexity), organisation (top management support, resources), environment (policy, competition) | Root factors for adoption: Lack of awareness, data risks, weak leadership and unclear policies form the foundation of adoption challenges.  Direct barriers at the decision level: Competitive pressures, patient trust and weak partnerships directly influenced adoption choices.  Technological challenges: The complexity of AI systems, scepticism of AI capabilities and medically derived risks hindered acceptance.  Organisational constraints: Lack of talent, financial costs and inability to share information were key internal barriers.  Environmental pressures: Geographical disparities and unclear hospital ownership complicated AI implementation.  Most constraining factor: Information sharing limitations were identified as the strongest bottleneck in adoption.  Social and patient needs: Meeting elderly patients’ complex needs and gaining trust were crucial for successful adoption. | Limited to Chinese hospitals  Self-reported survey bias  Cross-sectional design  Excluded patient perspectives |
| [[38]](https://journals.lww.com/cinjournal/abstract/2025/05000/feasibility_and_acceptability_of_smartwatches_for.11.aspx) | Johnson AH, Lee K, Reeder B, Popejoy L, Vogelsmeier A. Feasibility and acceptability of smartwatches for use by nursing home residents. CIN Comput Inform Nurs. 2025;43(5):e01245. doi:10.1097/CIN.0000000000001245 | United States – Nursing home within a hospital system in a rural Midwestern community. | Nursing home stakeholders, including nursing home leaders, direct care staff, residents, and residents’ family members. | Three-phase feasibility and acceptability evaluation of Fitbit Versa 3 smartwatch in a US nursing home | 4 residents (aged, varied mobility: 1 wheelchair, 2 walker, 1 independent); 2 family caregivers; 10 staff (nursing home leaders, nurses, therapists, certified nursing assistants) | Phase 1: pre-trial interviews with leaders, staff, residents and families; Phase 2: 7-day smartwatch wear trial; Phase 3: post-trial interviews or focus groups. Thematic analysis applied | Integration into workflow: Staff emphasised that smartwatch data must be embedded into daily routines, not add extra burden.  Technical usability issues: Residents faced difficulties with step tracking, reading displays and syncing devices.  Social normalisation: Residents accepted smartwatches as part of modern life, comparing them to regular watches.  Privacy concerns: Some residents worried about who could access their health data.  Health monitoring value: Residents and staff valued real-time tracking of heart rate, oxygen, sleep and activity.  Family involvement: Families wanted access to smartwatch data to monitor loved ones and reduce uncertainty.  Clinical utility: Staff highlighted smartwatch alerts for illness detection and rehab tracking as highly useful.  Positive attitudes toward adoption: Residents were excited and open, despite age-related digital divide concerns.  Barriers to feasibility: Mobility limitations, Bluetooth connectivity issues and small displays threatened adoption. | Small sample size, limited representativeness  Short duration (7 days)  Single site, US rural nursing home  Only one smartwatch model tested |
| [[39]](https://journals.lww.com/cinjournal/fulltext/2024/08000/user_needs_and_factors_associated_with_the.6.aspx) | Kahsay DT, Salanterä S, Tommila M, Liukas T, Rosio R, Diji AK, et al. User needs and factors associated with the acceptability of audiovisual feedback devices for chest compression monitoring in cardiopulmonary resuscitation. CIN Comput Inform Nurs. 2024;42(8):583-92. doi:10.1097/CIN.0000000000001126 | Finland – Southwest Finland Rescue Department, Turku University Hospital, and Southwest Finland Emergency Services; Ghana – Ghana Red Cross Society (Ashanti Branch) and Komfo Anokye Teaching Hospital, Kumasi. | Healthcare professionals and Red Cross–trained first aid personnel involved in emergency and lifesaving activities, including paramedics, emergency nurses, intensive care nurses, anesthesiologists, and first aiders. | Qualitative descriptive study used UTAUT to explore the acceptability of audiovisual feedback (AVF) devices for chest compression monitoring in CPR | 22 participants (18 Finland, 4 Ghana). Professions: paramedics (50%), intensive care nurses (13.7%), emergency nurses (13.7%), anaesthesiologists (9%), Red Cross first aiders (13.7%) | Interviews guided by UTAUT (performance expectancy, effort expectancy, social influence, facilitating conditions). Conducted via Zoom, audio-recorded, transcribed, and thematically analysed | Perceived usefulness drives acceptance: Users emphasised the need for clear, reliable and efficient feedback from devices.  Improved outcomes expected: Devices were seen to enhance CPR quality and patient survival.  Applicability across contexts: Users wanted devices usable in different environments, weather conditions and for all patient groups.  Ease of use matters: Simplicity, lightweight design and user-friendly features encouraged adoption.  Complexity as a barrier: Overly complex feedback discouraged users, especially laypersons.  Influence of colleagues and supervisors: Social reinforcement from respected peers encouraged adoption.  Organisational decisions shape use: Adoption often followed institutional directives, not personal choice.  Training and staff competence: Regular training and technical support were essential for acceptance.  Cost as barrier or enabler: High cost discouraged adoption, particularly in resource-limited settings.  Compatibility encourages use: Interoperability across settings and devices supports adoption. | Uneven exposure to AVF devices among participant  Small groups, some dyadic interviews  Limited generalisability despite cross-country sample |
| [[40]](https://dl.acm.org/doi/10.1145/3609327) | Karnatak N, Loughrin B, Kuo TA, Mateu-Silvernail O, Medhi Thies I, Thies W, et al. Is it even giving the correct reading or not? How trust and relationships mediate blood pressure management in India. ACM Trans Comput Hum Interact. 2023;30(6):90. doi:10.1145/3609327 | India – Low-resourced rural and semi-urban communities in Haryana, Uttarakhand, and Uttar Pradesh. | Adults aged ≥18 years clinically diagnosed with abnormal blood pressure from low-income, low-resource communities, comfortable communicating in English or Hindi. | Explored experiences of managing abnormal blood pressure among low-income communities in rural or semi-urban India, focusing on trust and relationships in blood pressure management through semi-structured interviews | 21 patients from Haryana, Uttarakhand and Uttar Pradesh (8 female, 13 male; mean age ≈ 47; hypertensive = 15, hypotensive = 6) | Conducted in Hindi over phone calls (45-60 minutes); recorded, translated, transcribed; inductive coding and thematic analysis used | Diagnosis often accidental: Many participants discovered abnormal blood pressure by chance, not through intentional monitoring.  Lifestyle adjustments as feasible actions: Participants adopted only manageable changes, despite awareness of healthier practices.  Reliance on home remedies: Low-cost, culturally familiar remedies were preferred, especially for hypotension.  Medication adherence gap: Forgetfulness, workload and subsiding symptoms led to irregular medication use.  Distrust in digital devices: Participants questioned the accuracy of digital blood pressure devices compared to manual ones.  Low uptake of teleconsultation: Lack of awareness, trust and comfort hindered adoption of teleconsultation.  Trust in quacks and local providers: Informal providers were trusted more than hospital staff for accessibility and attentiveness.  Social circles as key influencers: Friends, neighbours and family influenced doctor choice, medicines and lifestyle.  Device sharing practices: Blood pressure devices were often shared within communities, reinforcing social ties but with boundaries.  Hidden costs of care: Transport, lost wages, and companion costs added burdens even when services were free.  Gendered barriers for women: Household responsibilities and dependence on men limited women’s access to care.  Family as care and tech intermediaries: Relatives supported with remedies, physical help and technology use. | Limited to Hindi-speaking, North Indian low-income communities  Small sample size  Did not account for caste or religion influences |
| [[41]](https://journals.sagepub.com/doi/10.1177/20552076221150745) | Keogh A, Alcock L, Brown P, Buckley E, Brozgol M, Gazit E, et al. Acceptability of wearable devices for measuring mobility remotely: observations from the Mobilise-D technical validation study. Digit Health. 2023;9:20552076221150745. doi:10.1177/20552076221150745 | Israel, Germany, and United Kingdom – Five European study sites including hospitals and research institutions. | Healthy older adults and participants from clinical cohorts with Parkinson’s disease, chronic obstructive pulmonary disease, chronic heart failure, multiple sclerosis, and recovery from proximal femoral fracture. | Prospective, mixed-methods study within the Mobilise-D technical validation study; combined quantitative questionnaires with qualitative semi-structured interviews | 106 participants completed questionnaires (10 congestive heart failure, 17 chronic obstructive pulmonary disease, 20 healthy adults, 20 multiple sclerosis, 20 Parkinson’s disease, 19 proximal femoral fracture). A subset of 36 participants completed interviews | Comfort Rating Scale (CRS) and Rabinovich questionnaire assessed device comfort and acceptability; interviews were conducted with around 40% of participants, audio-recorded, transcribed and content-analysed | High comfort and acceptability: Participants rated the device as both comfortable and acceptable across cohorts.  Ease of use: Minimal interaction and simple positioning made the device easy to use.  Comfort preferences: Comfort varied by personal preference, but strap irritation and size were noted.  Perceived lack of direct usefulness: Patients trusted that it was collecting data, but wanted some feedback.  Limited willingness for long-term wear: Wearing for a week was acceptable, but longer caused reluctance  Trust in institutions vs companies: Users trusted clinicians and universities but distrusted commercial companies.  Communication needs with healthcare: Technology is seen as a bridge to improve patient-clinician communication.  Altered expectations and lifestyle adjustments: Illness progression forced lifestyle changes, influencing openness to tech.  Uncertainty of technology’s role: Participants did not fully understand how tech could help their condition. | Single researcher conducted qualitative analysis  Interview sample skewed towards UK and multiple sclerosis participant  COVID-19 pandemic affected recruitment, especially congestive heart failure and chronic obstructive pulmonary disease  Some interviewers had limited qualitative research experience  Uncertainty about saturation in exploratory component  No measurement of health or digital literacy |
| [[42]](https://www.jmir.org/2023/1/e44131) | Kerr JI, Naegelin M, Benk M, von Wangenheim F, Meins E, Viganò E, et al. Investigating employees’ concerns and wishes regarding digital stress management interventions with value sensitive design: mixed methods study. J Med Internet Res. 2023;25:e44131. doi:10.2196/44131 | Switzerland – Employees recruited via an intranet news post at a large Swiss insurance company. | Employees of the Swiss insurance company; inclusion criteria included being employed by the company, aged 18–65 years, and working on a computer for most working hours. | Mixed-methods study using the Value Sensitive Design (VSD) framework: literature review to identify values, followed by a web-based quantitative survey and qualitative free-text analysis of employees’ concerns and wishes regarding digital stress management interventions (dSMIs) | 241 recruited, 170 participants included in final quantitative analysis (after exclusions); 71 female (41.8%), 99 male (58.2%); varied positions (management vs employees), age groups, stress levels and prior tech experience | Quantitative: web-based closed questions on identified values, user acceptance and trust. Qualitative: framework method analysis (FMA) of free-text responses from 70 participants (85 responses) | Health and well-being concerns: Employees worried dSMIs may not reduce stress or might worsen it.  Privacy concerns: Data security and fear of surveillance were significant issues, especially with machine learning (ML) monitoring.  Autonomy concerns: Employees stressed the importance of voluntary use and control over interaction with dSMIs.  Accountability concerns: Calls for certified oversight, transparency, and employer responsibility.  Identity concerns: Fears of stigmatisation at work due to visible use of dSMIs.  Digital independence: Some employees preferred non-digital methods, resisting more digital engagement.  Integrability: Desire for seamless integration with existing devices and apps.  User-friendliness: Preference for simple, intuitive design and adaptability.  Trust and usefulness: Intention to use and perceived usefulness were moderate to high but varied with prior experience and trust levels. | Different preconceived ideas of dSMIs among participants may have influenced responses  Possible imbalance in experimental conditions due to video design  Values list and scales not exhaustive; self-developed survey  Self-selection bias; recruited from one Swiss insurance company only  Limited power for small effect sizes; larger samples needed for validation |
| [[43]](https://www.frontiersin.org/journals/public-health/articles/10.3389/fpubh.2025.1531173/full) | Kim B, Jeong CH, Blood E, Arthanat S, Corvini M, Golomski C, et al. Multilevel factors influencing eHealth adoption among older adults during the pandemic. Front Public Health. 2025;13:1531173. doi:10.3389/fpubh.2025.1531173 | United States – Recruitment through community partners serving older adults in Northern New England; participants lived in a range of community housing settings. | Older adults aged 60 years and above living independently, with broadband access, and with at least one chronic condition and/or ongoing health issue; participants with cognitive impairment or progressive disorders were excluded. | Semi-structured virtual interviews guided by the Push-Pull-Mooring (PPM) and Unified Theory of Acceptance and Use of Technology (UTAUT) frameworks; analysed with constant comparative method and thematic coding | 31 older adults (aged 60-88, mean 74.27 years); 81% female; 45% lived alone; 74% had more than a high school education | Conducted remotely via Zoom or phone between June-September 2021; iPads/Wi-Fi provided for access; interviews recorded, transcribed verbatim, coded by two independent coders with negotiated agreement approach | Pandemic as push factor: COVID-19 forced older adults to adopt e-health due to shutdowns and distancing.  Pandemic-induced fear and anxiety: Fear of infection motivated e-health use.  Perceived usefulness: Convenience and better communication made e-health attractive.  Enhanced patient-provider communication: E-health enabled more direct and documented communication.  Ease of use with familiarity: Repetition and prior ICT experience improved confidence.  Social influence: Family and broader social movements encouraged adoption.  Security concerns: Fear of data misuse, especially Medicare and Social Security number (SSN).  Information overload: Fear of unnecessary messages discouraged adoption.  Unfamiliarity and lack of confidence: Limited skills caused anxiety in use.  Physical limitations: Typing and device use were effortful.  Lack of social influence: Absence of e-health use among peers reduced motivation.  Limited infrastructure: Costly equipment and poor internet restricted access. | Small, convenience sample may bias results  Online-only interviews may exclude less tech-comfortable participants  Possible novelty effect inflated adoption attitudes |
| [[44]](https://www.liebertpub.com/doi/10.1089/tmj.2021.0418?url_ver=Z39.88-2003&rfr_id=ori%3Arid%3Acrossref.org&rfr_dat=cr_pub++0pubmed) | Kim HM, Xu Y, Wang Y. Overcoming the mental health stigma through m-health apps: results from the Healthy Minds Study. Telemed J E Health. 2022;28(10):1534-40. doi:10.1089/tmj.2021.0418 | United States – Data from the Healthy Minds Study conducted across multiple colleges and universities. | University and college students aged ≥18 years who owned a smartphone and reported no prior use of a mental health application. | Extended Technology Acceptance Model (TAM) was tested with sociocultural factors using structural equation modelling (SEM) | 429 US college students (mean age 23.53; 61.3% female; 78.3% White, 11.9% Asian, 7.2% Hispanic, 5.4% Middle Eastern, 4.7% African American) | Measures: mobile health app (MHA) acceptance (behavioural intent), perceived ease of use, perceived usefulness, stigma and mental health service utilisation | Perceived usefulness predicts acceptance: Students who found MHAs helpful were more likely to accept them.  Ease of use indirectly increases acceptance via usefulness: Ease of use raised perceived usefulness, which then increased acceptance.  Direct effect of ease of use not significant: Ease of use alone did not directly predict acceptance.  Stigma reduced in-person service use: Higher stigma was linked to lower utilisation of traditional services.  Indirect effect of stigma on MHA acceptance: Stigma did not directly lower acceptance, but reduced in-person use, which increased MHA openness.  Privacy and anonymity as strengths: MHAs were valued for confidentiality compared to face-to-face care.  Equal importance of tech and sociocultural determinants: Both usefulness/ease and stigma/service use pathways equally influenced acceptance. | Non-representative sample (mostly White students from US schools)  Secondary dataset not designed by researcher  Service utilisation measure limited to past 12 months  Behavioural intent measured, not actual use  Need for replication in diverse populations and real users |
| [[45]](https://www.jmir.org/2023/1/e41915) | Kim S, Chow BC, Park S, Liu H. The usage of digital health technology among older adults in Hong Kong and the role of technology readiness and eHealth literacy: path analysis. J Med Internet Res. 2023;25:e41915. doi:10.2196/41915 | Hong Kong, China. | Older adults in Hong Kong aged over 60 years. | Web-based survey using the Technology Readiness and Acceptance Model (TRAM) with e-health literacy as moderator; structural equation modelling used for hypothesis testing | 306 older adults (≥60 years) in Hong Kong (mean e-health literacy 3.417 ± 0.805). Demographics: gender balanced, 230 married, SES varied, around 91% accessed the internet via mobile | Measures: e-health literacy (eHEALS, Cronbach α = 0.939), technology readiness (optimism, innovativeness, discomfort, insecurity), TAM constructs (perceived usefulness, perceived ease of use, attitude, usage intention). Surveys translated into Cantonese, back-translated and pre-tested with 10 older adults | Optimism increases perceived usefulness and ease of use: Optimistic older adults were more confident in using digital health technology and saw it as beneficial.  Innovativeness improves ease of use: Older adults with higher innovativeness found digital health technology easier to use.  Discomfort reduces ease of use: Feeling overwhelmed by technology discouraged adoption.  Ease of use enhances usefulness: Older adults who found digital health technology easy to use also found it more useful.  Positive attitudes drive intention: Attitudes shaped by usefulness and ease of use predicted intention.  E-health literacy strengthens positive effects: High literacy enhanced optimism and innovativeness while weakening discomfort effects.  Socioeconomic and marital status influence literacy: Married and higher-income older adults had higher e-health literacy.  Training needed to overcome discomfort and insecurity: Education and guidance could help reduce fear and enhance skills. | Sample excluded tech-unwilling older adults  Limited generalisability beyond Hong Kong  Other influencing factors beyond TRAM not included  Measured intention, not actual behaviour |
| [[46]](https://pmc.ncbi.nlm.nih.gov/articles/PMC9790857/) | Kim YK, Ang S. Older adults with functional limitations and their use of telehealth during COVID-19. Res Aging. 2023;45(9-10):609-19. doi:10.1177/01640275221147642 | United States. | Medicare beneficiaries aged 70 years and older in 2020, with varying levels of functional limitations. | Analysis of the National Health and Aging Trends Study (NHATS) COVID-19 supplementary survey linked with the main NHATS data (2018-2020) and the Family and Friends Survey; logistic regression with average marginal effects | 3,151 Medicare beneficiaries aged ≥70 (mean age 78.3; 56% female; 54% married; 81% non-Hispanic White). Subsample of 1,458 had family or friend tech-support survey | Measures: functional limitations (6 tasks), internet use, telehealth use (video calls, emails/texts/portal), access to computer, informal tech support. Covariates included demographics, SES, health conditions and dementia status | Functional limitations reduce computer and internet access: Older adults with more limitations had lower access to digital tools.  Functional limitations increase video-based telehealth use: Those with more limitations were more likely to adopt video calls with providers.  No effect on text/portal use: Functional limitations did not affect use of emails, texts or portals.  Recent internet use as moderator: Prior consistent internet use supported telehealth adoption.  Family and friend tech support: Social support increased the likelihood of telehealth use among those with limitations.  Digital exclusion risk: Without supportive design and access, older adults with limitations risk being excluded. | Cross-sectional data limits causal inference  Sample limited to older Medicare beneficiaries aged ≥70  Overestimation of computer access possible  Under-reporting of informal support possible  Lack of detail on type/frequency of telehealth use  Cohort effect not addressed |
| [[47]](https://journals.plos.org/plosone/article?id=10.1371/journal.pone.0303416) | Kiruthu-Kamamia C, Berner-Rodoreda A, O’Bryan G, Sande O, Huwa J, Thawani A, et al. Acceptability and feasibility of a mobile electronic medical record system for community-based antiretroviral therapy in Lilongwe, Malawi: a rapid qualitative analysis. PLoS One. 2025;20(5):e0303416. doi:10.1371/journal.pone.0303416 | Malawi – Lilongwe; Lighthouse Trust clinics and community-based ART services. | Healthcare workers involved in the nurse-led community-based ART program and the CARES app, including clinical, managerial, monitoring and evaluation, and IT/data staff. | In-depth interviews with healthcare workers (HCWs) analysed using rapid qualitative analysis guided by Technology Acceptance Model (TAM1, TAM2 and extended model) | 15 HCWs (10 nurses, 5 monitoring and evaluation/IT staff; median age 39; median employment 4 years) | IDIs conducted September-October 2023; interviews guided by TAM2 and CARES optimisation processes; audio-recorded, transcribed, analysed using rapid matrix-based RQA approach | High perceived usefulness: CARES app valued for improving antiretroviral therapy (ART) service delivery and data management.  Ease of use through familiarity with electronic medical record systems (EMRS): Interface design similar to EMRS improved usability.  Output quality problems: App slowness, resets and data errors undermined efficiency.  Social influence and organisational pressure: Continued use is partly driven by expectations from supervisors and developers.  Optimism and cognitive processes: Users stayed hopeful about future improvements and relevance for their work.  Continuous engagement as a success factor: Iterative involvement of HCWs and feedback loops improved design.  Sustainability needs: Infrastructure, training and stakeholder alignment are essential for long-term adoption.  Integration with other services: Expansion to other health areas and interoperability with EMRS are critical for success. | Small sample size, though included all relevant HCWs  Conducted during prototype testing, not post-optimisation  Potential social desirability bias |
| [[48]](https://journals.sagepub.com/doi/10.1177/11786329241284401?url_ver=Z39.88-2003&rfr_id=ori:rid:crossref.org&rfr_dat=cr_pub%20%200pubmed) | Kitole FA, Shukla S. Cloud horizons: strengthening rural healthcare through telemedicine’s digital canopy. Health Serv Insights. 2024;17:11786329241284401. doi:10.1177/11786329241284401 | Tanzania – Morogoro Region (Mvomero District), including selected wards and primary healthcare facilities. | Healthcare workers from public, private, and faith-based primary healthcare facilities in the study area. | Multi-stage sampling of healthcare facilities in rural Tanzania; descriptive statistics for quantitative data; thematic analysis for qualitative insights | 44 healthcare workers (50% private, 37.5% public, 12.5% faith-based); 57.5% male; 52.5% vocational training, 30% university education | Structured questionnaires with 44 practitioners; 4 key informants from private, public and faith-based facilities. Qualitative analysis in NVivo, quantitative analysis in SPSS | Perceived benefits drive adoption: Improved access, cost efficiency, timely consultations, monitoring and prescription management encouraged use.  Technology cost as a barrier: High costs limited adoption across facilities.  Legal practices as constraints: Registration requirements and data transfer standards created adoption challenges.  Resource availability and affordability: Lack of infrastructure and inputs limited adoption in rural areas.  Technological innovation and finance: Supply-side adoption depended on innovation and access to financial resources.  Political willingness and regulatory support: Environmental factors such as political and institutional backing strongly shaped adoption.  Data privacy and security needs: Trust requires robust data protection in telemedicine use.  Organisational differences in adoption: Faith-based and private facilities showed stronger adoption than public facilities due to resources and autonomy.  Policy and training needs: Targeted strategies, training and clear policies were required to enable adoption. | Small sample size  Lack of pilot testing  Potential interviewer bias  Reliance on self-reported data  Need for larger and more diverse studies |
| [[49]](https://www.frontiersin.org/journals/public-health/articles/10.3389/fpubh.2025.1547189/full) | Larnyo E, Tettegah S, Nutakor JA, Addai-Dansoh S, Arboh F. Technology access, use, socioeconomic status, and healthcare disparities among African Americans in the US. Front Public Health. 2025;13:1547189. doi:10.3389/fpubh.2025.1547189 | United States – National survey of non-institutionalized civilian adults. | African American adults aged 18 years and older. | Analysis of Health Information National Trends Survey (HINTS) Wave 6 dataset using partial least squares structural equation modelling (PLS-SEM) | 815 African Americans (majority female, most aged 50-64; education mostly “some college”; most income $20,000-34,999) | Measures: healthcare disparity (discrimination, delays in care), socioeconomic status (education, income), technology access (device ownership, internet use), health technology use (telehealth, online health video/forums, sharing). Validated with reliability, convergent and discriminant validity tests | Technology access improves health technology use: Better access increases adoption of health technologies.  Technology access reduces healthcare disparities: Access helps mitigate inequities in care.  Health technology use reduces disparities: Use of apps, telehealth and electronic health records improves equity.  Socioeconomic status enhances technology access: Higher socioeconomic status boosts access to devices and the internet.  Socioeconomic status moderates the impact of technology on disparities: Benefits of access vary by status levels.  Systemic racism shapes disparities: Structural racism, discrimination and digital redlining worsen inequities.  Low socioeconomic status creates multiple barriers: Cost, literacy and infrastructure gaps reduce adoption.  Need for policy and community programmes: Broadband, subsidies and training can reduce disparities. | US-only sample limits generalisability  Reliance on self-reported data  Cross-sectional design limits causal claims  Limited measure of technology access |
| [[50]](https://games.jmir.org/2024/1/e47600) | Lee EWJ, Tan WW, Pham BTP, Kawaja A, Theng YL. Addressing data absenteeism and technology chauvinism in the use of gamified wearable gloves among older adults: moderated usability study. JMIR Serious Games. 2024;12:e47600. doi:10.2196/47600 | Singapore – Participants recruited from an older adult activity center. | Older adults aged 50 years and above, including individuals with and without a history of stroke. | One-hour usability sessions including gameplay, survey and interviews; combination of quantitative usability scale and qualitative thematic interviews | 19 older adults (11 with stroke history, 8 without). Average age 66.8 years; 53% female. Ethnicity: 68% Chinese, 21% Malay, 5% Burmese, 5% Singh. 74% below A-Level education | Participants used a wearable glove integrated with “Chinatown Race” exergame; completed System Usability Scale (SUS, α = .95), then participated in thematic interviews analysed bottom-up for motivations and barriers | Competition motivates use: Gamification elements like points and collectibles encouraged rehabilitation adherence.  Helplessness from technical difficulties: Unfamiliarity with glove controls led to feelings of helplessness and demotivation.  Stroke survivors require more assistance: Those with stroke history needed greater technical help compared to non-stroke participants.  Comfort influences adoption: Comfort level was especially important for stroke survivors.  Different priorities between stroke vs non-stroke users: Stroke survivors focused on movement controls, while non-stroke participants focused on aesthetics.  Aversion to new tech among stroke survivors: Prior rehab experience fostered scepticism toward novel tools.  Gameplay difficulty balancing needed: Games that were too difficult risked demotivating participants.  Equity and representation in design: Addressing data absenteeism and involving older adults in co-design was crucial. | Small sample size  One-session exposure only  Volunteer bias possible  Health conditions not fully accounted |
| [[51]](https://bmcdigitalhealth.biomedcentral.com/articles/10.1186/s44247-024-00127-3) | Leenen JPL, Jordens YJ, Wegman A, et al. Experiences of nurses working in novel virtual care centres in the Netherlands: a qualitative study. BMC Digital Health. 2024;2:70. doi:10.1186/s44247-024-00127-3 | The Netherlands – Virtual care centres in four teaching hospitals. | Registered nurses employed in hospital-based virtual care centres who used information and communication technologies at work and were able to speak and understand Dutch. | Semi-structured interviews with virtual care centre (VCC) nurses; analysis mapped to the capability, opportunity, motivation-behaviour (COM-B) model | 13 nurses (all female, mean age 45 ± 8 years; mean 24 years work experience, 1.6 years in VCC) | Conducted February-July 2023 with VCC nurses from 4 Dutch teaching hospitals; interviews lasted about 36 minutes, transcribed verbatim, thematically analysed (Braun and Clarke six-stage approach) using Atlas.ti, with member checking for validation | Changing nurse-patient relationship: Nurses maintained warm, supportive relationships despite remote care, but needed persuasion and empathy skills.  Transformation of care role: Nurses acted as bridges, developing new digital pathways and driving innovation.  Communication skills essential: Virtual care requires motivational interviewing, careful listening and directive counselling.  General and clinical knowledge needed: Broad nursing knowledge and prior clinical experience were crucial for VCC work.  Education and training gap: Nurses arranged their own training, highlighting the need for structured programmes.  Need for protocol development: Clear, consistent and tested protocols guided effective care delivery.  Technological preconditions: Usability, integration and reducing double registration were major challenges.  Collaboration with other professionals: Gaining trust of hospital colleagues was vital for adoption.  Impact on nurses: Work was less physically demanding but sedentary; autonomy and variety increased satisfaction.  Impact on patients: Patients felt safer at home and more empowered, though compliance and willingness varied.  Impact on hospital: Virtual care freed hospital capacity and increased efficiency, but required upfront investment. | Only female nurses included  Perspectives limited to nurses only  Care pathways not differentiated  COM-B framing may have constrained interpretation |
| [[52]](https://www.nature.com/articles/s41746-023-00924-6) | Lee VV, Vijayakumar S, Ng WY, et al. Personalization and localization as key expectations of digital health intervention in women pre- to post-pregnancy. npj Digit Med. 2023;6:183. doi:10.1038/s41746-023-00924-6 | Singapore – Recruitment from the National University Hospital and the community in Singapore. | Women who were trying to conceive, currently pregnant, or up to two years postbirth; eligibility included English fluency and age 21–45 years; exclusion criteria included cognitive impairment, psychiatric disorder, significant hearing impairment, and assisted conception. | Semi-structured qualitative interviews exploring women’s digital health needs and expectations across preconception, pregnancy and post-birth phases; inductive thematic analysis | 44 women (13 pre-pregnancy, 16 during pregnancy, 15 post-pregnancy). Age range 21-40 years, mean 31.6 years; ethnicity: 75% Chinese, 9.1% Malay, 9.1% Indian, 6.8% others | Explored participants’ experiences with digital health tools, healthcare journeys, motivators and barriers for adoption. Interviews audio-recorded, transcribed and analysed inductively by 5 researchers | Need for data-driven personalised interactions: Women valued adaptive tracking with actionable feedback relevant to their phase.  Timeliness of feedback: Timely, phase-specific feedback enhanced engagement and personalisation.  Journey organisation through milestones: Women wanted pregnancy and child milestones to guide decision-making.  Privacy trade-off: Willingness to share personal data depended on perceived benefits and source credibility.  Trust in government and hospitals: Women trusted digital health interventions more if backed by hospitals or government.  Access to local healthcare resources: Women wanted apps that connected them with local services.  Understanding local healthcare processes: Confusion about healthcare navigation created demand for localised guidance.  Localised lifestyle guidance: Women wanted culturally relevant diet and lifestyle information.  Community and peer connection: Sharing personal anecdotes among women created reassurance and normalisation. | Singapore-specific cultural context may limit generalisability  Privacy-personalisation paradox requires more research  Did not test interventions, only explored expectations  Volunteer and tech-savvy sample bias |
| [[53]](https://humanfactors.jmir.org/2024/1/e58641) | Leong QY, Lee VV, Ng WY, Vijayakumar S, Lau NY, Mauritzon I, et al. Older adults’ perspectives and experiences with digital health in Singapore: qualitative study. JMIR Hum Factors. 2024;11:e58641. doi:10.2196/58641 | Singapore. | Older adults aged 60–80 years in Singapore with experience using health-related technologies; participants were fluent in English and from Chinese, Indian, and Malay ethnic groups. | Semi-structured interviews exploring older adults’ experiences and perspectives of digital health adoption and use in Singapore; Braun and Clarke’s six-step thematic analysis | 16 older adults aged 60-80 (mean 66.9 years; 69% female; 56% Chinese, 31% Indian, 13% Malay). Conditions included hypertension, diabetes, hyperlipidaemia, cardiovascular disease and others | Recruitment via purposive sampling (social media, outreach); interviews recorded, transcribed verbatim, inductively coded by team; themes refined collaboratively; ethical approval obtained; participants reimbursed | Support in developing digital health literacy: Older adults needed education, workshops and personalised support to overcome barriers.  Accessibility challenges: Poor memory, slower cognition and lack of support made adoption harder.  Trust in multiple sources: Trust was built through recommendations from doctors, family, friends and institutions.  Loss of trust from cybersecurity concerns: Fear of phishing and misuse of Singpass data reduced confidence.  Incentives as motivators: Monetary rewards, free devices and gamification encouraged adoption.  Observable health outcomes drive sustained use: Users continued only if they saw results.  Intrinsic drive to be healthy: Desire for longevity and holistic well-being motivated use.  Need for personalised and localised content: Generic or Western-based information reduced relevance.  Telehealth valued for convenience, but preference for in-person remains: Telehealth was seen as good for mobility-limited or sick individuals. | English-only participants excluded non-English speakers  Recruitment via social media and outreach may miss vulnerable groups  Volunteer bias possible  Singapore-specific context limits generalisability |
| [[54]](https://www.mdpi.com/2227-9067/11/10/1215) | Linder L, Utendorfer H, Oliveros B, Gilliland S, Tiase VL, Altizer R. Usability evaluation of the revised Color Me Healthy symptom assessment app: perspectives of children and parents. Children (Basel). 2024;11(10):1215. doi:10.3390/children11101215 | United States – Ambulatory oncology clinic at Primary Children’s Hospital, Salt Lake City, Utah. | School-age children aged 6–12 years receiving cancer treatment and their parent or legal guardian; participants were required to speak and understand English and be physically and cognitively able to complete study procedures. | Iterative usability testing of app revisions using cognitive walkthroughs, usability logs, Technology Acceptance Model (TAM) perceived usefulness surveys and interviews | 14 child-parent dyads (14 children, median age 8.5 years, range 6-12; 14 parents, median age 38.5 years, range 34-49) | Children and parents tested app features (symptom reporting, pain localisation, data review). TAM perceived usefulness measured. Qualitative interviews captured experiences, analysed with content analysis | High perceived usefulness: Parents rated the app highly useful for supporting symptom recognition and response.  Ease of use improved with iteration: Children and parents required fewer prompts after refinements, showing learnability.  Computer self-efficacy and confidence: Both groups showed confidence in navigating the interface.  Playfulness and engagement: Children enjoyed customising avatars and interacting with features, increasing engagement.  Perceived enjoyment: Children and parents endorsed features like drawing and customisation as enjoyable.  Job relevance for parents and children: The app was seen as relevant for symptom tracking, supporting recall and easing reporting burden.  Result demonstrability: Graphs and history features helped visualise and interpret symptoms.  Developmental relevance: Younger children benefited from emoji-based responses; older children valued data reflection.  Parental perceptions of value: Parents saw the app as beneficial for caregiving and understanding children’s experiences.  Gamification enhances motivation: Requests for badges, animations and rewards show the importance of gamification. | Single site recruitment  Limited racial or ethnic diversity  Small sample size, though aligned with usability study norms  Diagnosis-specific sample (mostly leukaemia)  More mothers than fathers participated |
| [[55]](https://academic.oup.com/heapol/article/37/8/943/6545194) | Liverani M, Ir P, Perel P, Khan M, Balabanova D, Wiseman V. Assessing the potential of wearable health monitors for health system strengthening in low- and middle-income countries: a prospective study of technology adoption in Cambodia. Health Policy Plan. 2022;37(8):943-51. doi:10.1093/heapol/czac019 | Cambodia – Stakeholder interviews conducted in Phnom Penh, focused on health system strengthening in a low- and middle-income country context. | National and international stakeholders with expertise in noncommunicable diseases and/or involvement in mHealth programmes, including Ministry of Health managers, NGO staff, and representatives of multilateral organizations. | Guided by the non-adoption, abandonment, scale-up, spread, sustainability (NASSS) framework, adapted for the low- and middle-income country context | 18 stakeholders (7 Ministry of Health managers, 3 local NGO directors, 4 international NGO managers, 4 officers from multilateral organisations, including WHO or World Bank) | Interviews with national and international stakeholders (March-October 2019, Phnom Penh); demonstration of watch-type wearable (blood pressure, heart rate, sleep, steps, calories). Literature review of non-communicable diseases and m-health in Cambodia for contextual insights | Wearables valuable for non-communicable disease risk surveys: Most feasible current use is population-level data collection.  Potential for patient monitoring in future: Long-term telemonitoring is possible but faces feasibility issues.  Health information system limitations: Existing systems are not designed for non-communicable diseases and the integration of wearable data.  Accuracy and technical reliability concerns: Questions about accuracy, battery life and resilience limited trust.  Need for open APIs and localisation: Customisation was seen as critical but limited by proprietary restrictions.  Adopter challenges: Uptake is harder among the elderly, farmers and those unfamiliar with tech; training is needed.  Equity concerns: Risk of reinforcing inequalities if poorer groups cannot afford wearables.  Infrastructure limitations: Internet and electricity gaps restricted usability, especially in rural areas.  Policy and data protection needs: Weak regulatory framework and lack of health data privacy laws.  Sustainability challenges: Donor fragmentation, lack of coordination and limited national ownership threaten long-term adoption. | Prospective and exploratory (not real-world tested)  Limited to the Cambodia context  Did not evaluate cost-effectiveness or outcomes  Technology demonstration only, not actual deployment  Potential bias from donor and stakeholder perspectives |
| [[56]](https://aging.jmir.org/2024/1/e52292) | Lu SY, Yoon S, Yee WQ, Ngiam NHW, Ng KYY, Low LL. Experiences of a community-based digital intervention among older people living in a low-income neighborhood: qualitative study. JMIR Aging. 2024;7:e52292. doi:10.2196/52292 | Singapore – Public rental housing estates in low-income neighborhoods during the COVID-19 pandemic period. | Older adults aged ≥60 years who completed a community-based digital intervention and lived in public rental housing. | Semi-structured in-depth interviews with older adults after completing Project Wire Up, a home-based, volunteer-led smartphone literacy programme | 19 participants (≥60 years; 63% female; 84% Chinese; 79% lived alone; 32% no formal education) | Conducted Dec 2020-Mar 2021 in English and local dialects; audio-recorded, transcribed, translated; thematic analysis with NVivo; reflexive and constant comparative coding; COREQ checklist applied | Age-related decline and internalised ageism: Physical and cognitive decline, along with negative self-perceptions, hindered learning and sustained use.  Self-imposed stigma and social precarity: Illiteracy, language barriers and social status reinforced feelings of inadequacy and reluctance to learn.  Technological design as exclusion: Smartphones’ design (small fonts, sensitivity, complex apps) conflicted with older adults’ priorities and needs.  Perceived irrelevance of technology: Smartphones were seen as unnecessary or irrelevant to daily life and the priorities of older age.  Social support and volunteer relationships: Interactions with volunteers and learning communication features created social connectedness.  Improved communication and social connection: Learning video calls and apps helped maintain relationships and reduce isolation.  Need for relevance and customisation: Relevance to participants’ lived experiences and goals was essential for sustained motivation.  Importance of addressing ageism and building strengths: Interventions should dismantle ageist stereotypes and empower older adults.  Programme continuity and practice needed: Lack of continued practice reduced motivation for sustained use post-intervention. | Did not analyse subgroup differences (e.g. sex, health, age profiles)  Singapore-specific context may limit transferability  Focused only on older adults in low-income neighbourhoods  Cross-sectional, post-intervention only |
| [[57]](https://www.jmir.org/2024/1/e50457/) | Manning JB, Blandford A, Edbrooke-Childs J. Facilitators of and barriers to teachers’ engagement with consumer technologies for stress management: qualitative study. J Med Internet Res. 2024;26:e50457. doi:10.2196/50457 | England – Educational settings involving secondary school teachers. | Secondary school teachers in England engaging with consumer technologies for stress management. | Longitudinal experiential qualitative study using reflexive thematic analysis informed by interpretive phenomenological analysis. Teachers trialled their chosen consumer technology over the summer and autumn terms (up to 27 weeks) | 8 teachers (heads or assistant heads of year) from one South London multi-academy trust. 6 continued into the extended autumn study | Workshops helped teachers choose a technology (Withings Steel HR smartwatch, Wysa chatbot, Daylio app, Teacher Tapp app). Data came from interviews (32-76 minutes), surveys and feedback forms. NVivo and SimpleMind used for coding and thematic analysis | Convenience of passive data collection: Wearables automatically logged activities, allowing teachers to engage without extra effort.  Brevity of interaction supports usability: Quick and simple interactions reduced burden and made use feasible in busy contexts.  Discreet design encourages use: Subtle appearance and unobtrusive notifications allowed teachers to use technology without stigma.  Automated reminders and feedback facilitated reflection: Prompts and congratulatory messages encouraged awareness and self-care.  Data visualisation enabled stress awareness: Visual patterns and quantifiable metrics helped teachers connect stress to specific contexts.  Educational and supportive features broadened benefits: Chatbot interactions provided language to support colleagues and educational insights.  Lack of instructions hindered adoption: Teachers struggled due to poor setup information, relying on trial and error and YouTube.  Concerns about accuracy and normal data standards: Users worried about discrepancies and the absence of benchmarks for comparison.  Connectivity and accessibility issues: Limited Bluetooth range and phone proximity created difficulties.  Competing workplace demands limited use: Teachers found it difficult to engage with technology in the school environment.  Need for supportive school culture: Teachers emphasised the importance of normalising stress management and providing private spaces. | Possible digital placebo effect due to research participation  Pandemic context influenced adoption and comparison  Limited autonomy in technology choice (restricted to taxonomy list)  Lack of broader technology types (mostly tracking, not treatment or entertainment)  Sample not fully representative (small, self-selecting, mostly female) |
| [[58]](https://www.mdpi.com/2227-9032/13/11/1222) | Marinescu ȘA, Oncioiu I, Ghibanu A-I. The digital transformation of healthcare through intelligent technologies: a path dependence-augmented–unified theory of acceptance and use of technology model for clinical decision support systems. Healthcare (Basel). 2025;13(11):1222. doi:10.3390/healthcare13111222 | Romania – Public and private healthcare settings. | Healthcare professionals, including specialist physicians, resident doctors, and nurses, working across multiple clinical specialties and exposed to AI-based clinical decision support systems. | Structured questionnaire based on an adapted path dependence-augmented UTAUT model, integrating TAM, UTAUT, information systems success and path dependence-augmented UTAUT factors | 440 healthcare professionals (radiology 95, oncology 75, cardiology 70, emergency medicine 65, neurology 35, endocrinology 25, gastroenterology 25, pulmonology 20, surgery 20, anaesthesiology 15) | Measures included perceived benefits, technology effort cost, attitudes, social or institutional influence, perceived control or transparency and intention to adopt AI clinical decision support systems (AI-CDSSs). Data analysed using PLS-SEM with SmartPLS 4; reliability, validity and path analysis conducted | Perceived benefits drive adoption: Utility in improving diagnostics, reducing errors and optimising decision-making strongly predicted adoption intention.  Technology effort cost discourages adoption: High costs in infrastructure, training and workflow changes reduced positive attitudes and intention.  Positive attitudes predict intention: Favourable perceptions significantly shaped adoption.  Benefits shape attitudes: Recognised clinical advantages increased favourable attitudes.  Costs undermine benefits: Perceived effort costs reduced perceived value of AI-CDSSs.  Social and institutional influence encourages adoption: Peer, organisational and guideline support fostered acceptance and intention.  Transparency and control build trust: Explainability and clinician oversight boosted confidence and adoption.  Mediating role of attitudes: Positive views amplified the effects of benefits, influence and transparency on adoption. Implementation needs: Intuitive design, pilot programmes and training reduce barriers and enhance adoption. | Romania-only sample limits generalisability  Potential selection bias (AI-familiar professionals)  Cross-sectional design  Did not assess cost-effectiveness or long-term impact |
| [[59]](https://bmcpsychiatry.biomedcentral.com/articles/10.1186/s12888-021-03426-5) | Melia R, Monahan L, Duggan J, et al. Exploring the experiences of mental health professionals engaged in the adoption of mobile health technology in Irish mental health services. BMC Psychiatry. 2021;21:412. doi:10.1186/s12888-021-03426-5 | Ireland – Irish public mental health services (HSE) in the West and Mid-West regions. | Mental health professionals and clinician managers working in child and adolescent, adult mental health, and primary care psychology services. | Semi-structured interviews with mental health professionals using Appreciative Inquiry to explore enablers and barriers to m-health adoption; inductive-deductive thematic analysis | 15 mental health professionals (5 psychologists in adult mental health, 3 psychologists and 2 psychiatrists in CAMHS, 3 psychologists in primary care child psychology, 2 psychologists in adult primary care) | Conducted July-October 2019 across Irish public mental health services; purposive sampling; interviews lasted 20-60 minutes, audio-recorded, transcribed, coded in NVivo; reflexivity maintained with reflective journalling | Improved service accessibility: Mobile apps enhanced access, engagement (especially youth) and crisis support.  Engagement for hard-to-reach populations: Digital tools helped those uncomfortable with face-to-face services.  Risk of over-accessibility for children: Concerns about unintended smartphone exposure.  Apps as “transitional objects”: Viewed as adjuncts to therapy, extending connection beyond sessions.  Therapeutic relationship remains central: Professionals emphasised in-person care as primary.  Integration with health systems needed: Apps required compatibility with IT systems and training.  Trust in technology: Usability, reliability and safeguarding concerns.  Trust in organisation: Fears tech may replace rather than complement services.  Training and research gaps: Professionals wanted guidance, evidence and training. | Qualitative design lacks precise causal or practical action points  Sample bias toward early adopters  Limited to psychology or psychiatry professionals  Ireland-only context limits generalisability |
| [[60]](https://www.frontiersin.org/journals/public-health/articles/10.3389/fpubh.2025.1542974/full) | Meraya AM, Khardali A, Ahmad S, Hoshaibari AZ, Alameer AA, Jaafari RY, et al. Telehealth perceptions and associated factors among older adults with chronic conditions in Saudi Arabia: a comparative study of users and non-users. Front Public Health. 2025;13:1542974. doi:10.3389/fpubh.2025.1542974 | Saudi Arabia – Jazan region. | Older adults aged 60 years and above residing in Jazan with at least one chronic condition. | Used the Technology Acceptance Model (TAM) framework to examine predictors of behavioural intention to use telehealth, with attitudes as a mediator; data analysed with structural equation modelling | 466 older adults (≥60 years) with ≥1 chronic condition in Jazan, Saudi Arabia; 256 telehealth users and 210 non-users | Survey measured TAM constructs (perceived usefulness, perceived ease of use, social impact, facilitating conditions, attitude, behavioural intention), facilitators (user-friendly design), barriers (internet, fear or dislike, appointment availability). Validated through pilot testing and Cronbach’s α > 0.7 | Perceived usefulness shapes attitude: Telehealth was seen as useful for health management, safety and quality of life.  Facilitating conditions strongly predict attitude: Resource availability, guidance and support encouraged positive attitudes.  Attitude mediates intention to use: Positive attitudes fully mediated the effects of usefulness and conditions on intention.  Ease of use and social impact not significant: Neither ease of use nor peer influence predicted adoption.  Interface simplicity as facilitator: Age-friendly visuals, minimal buttons and multimodal guidance enhanced adoption.  Systemic and cultural barriers: Appointment unavailability, internet issues and fear or dislike hindered adoption.  Demographic influences: Higher education, professional work history and health insurance increased adoption.  Policy and training needs: Subsidies, infrastructure and training for older adults are recommended. | Jazan-specific context limits generalisability  Snowball sampling may cause bias  No data on frequency of telehealth use  Barriers or facilitators analysed descriptively only  Questionnaire limited to closed-ended options  TAM may not generalise across all contexts |
| [[61]](https://www.tandfonline.com/doi/10.1080/03091902.2022.2089249?url_ver=Z39.88-2003&rfr_id=ori:rid:crossref.org&rfr_dat=cr_pub%20%200pubmed) | Moody L, Wood E, Needham A, Booth A, Jimenez-Aranda A, Tindale W. Identifying individual enablers and barriers to the use of digital technology for the self-management of long-term conditions by older adults. J Med Eng Technol. 2022;46(6):448-61. doi:10.1080/03091902.2022.2089249 | United Kingdom – NHS and university-affiliated settings (Coventry University; Sheffield Teaching Hospitals NHS Foundation Trust; University of Sheffield). | Older adults aged approximately 65 years and above with long-term conditions (diabetes, dementia, and chronic kidney disease), and an expert interview panel comprising condition-specific and technology experts. | Three systematic reviews of qualitative studies (diabetes, dementia, chronic kidney disease), followed by first-round Delphi interviews with experts; framework synthesis used (Senior Technology Acceptance Model (STAM)) | 14 expert panellists (6 dementia, 7 diabetes, 5 chronic kidney disease; 2 tech developers; 9 academics; 6 health professionals; 3 with lived experience) | Reviews: chronic kidney disease (22 studies), dementia (26 studies), diabetes (11 reviews). Delphi: one-to-one Zoom interviews with experts (2021), thematically analysed | Challenges of ageing: Older adults’ technology use is influenced by age-related factors such as digital literacy, motivation and co-morbidities.  Attitudes towards technology: Older adults value usefulness over novelty and perceive time cost in learning.  Denial of need: Many older adults avoid or delay using technology by underestimating their condition or needs.  Family and carer influence: Families often drive adoption, provide technical support and act as motivators.  Trust in technology and professionals: Trust in family, healthcare professionals and system reliability is crucial for adoption.  Information overload vs appropriateness: The quantity, timing and framing of information affect adoption.  Need for personalisation: Older adults require information and functions tailored to individual needs and preferences.  Financial barriers: Poverty and the cost of devices, maintenance and connectivity hinder adoption.  Support needs: Successful use requires onboarding, continuous support and guidance for both patients and carers.  Privacy and data sharing concerns: Older adults worry about big data, AI and the selling of patient data.  Accessible and aspirational design: Older adults reject clinical-looking devices; design must reduce stigma and increase desirability. | Gerontological focus largely missing  Tech design often not age-friendly  Prospective and exploratory only  Delphi only round 1 completed  UK-specific sample and funding context |
| [[62]](https://journals.sagepub.com/doi/10.1177/20552076241247196?url_ver=Z39.88-2003&rfr_id=ori:rid:crossref.org&rfr_dat=cr_pub%20%200pubmed) | Moody L, Wood E, Needham A, Booth A, Tindale W. Exploring how the design and provision of digital self-management technology can improve the uptake by older adults with chronic kidney disease, diabetes and dementia: a modified e-Delphi study. Digital Health. 2024;10:20552076241247196. doi:10.1177/20552076241247196 | United Kingdom – Context includes the NHS and UK-based institutions involved in technology development and provision. | Expert Delphi panel comprising individuals with lived experience, clinical expertise, academic expertise, and technology development/design expertise related to chronic kidney disease, diabetes, dementia, and digital self-management technologies for older adults. | Multi-stage Delphi with online semi-structured interviews (round 1) and two structured online surveys (rounds 2 and 3) to build expert consensus across chronic kidney disease, diabetes and dementia | 14 experts (6 dementia, 7 diabetes, 5 chronic kidney disease; 2 technology developers; 9 academics; 6 health professionals; 3 with lived experience). 11 of 14 completed all rounds | Round 1: Zoom semi-structured interviews → thematic analysis. Round 2: 26 statements rated (Likert 1-10) for consensus. Round 3: 7 unresolved statements re-rated. Consensus is defined as variance <4 | Limited digital skills and confidence: A major barrier to adoption is the lack of skills and confidence among older adults.  Financial barriers: Poverty and cost hinder access, maintenance and connectivity.  Importance of onboarding: Initial setup and training are crucial for building trust and long-term use.  Ongoing support and reassessment: Continuous support and reassessment of changing needs are essential.  Adaptability over one-size-fits-all: Technology must be adaptable to individual lives and homes.  Control over data sharing: Users want autonomy in deciding who can access their health data.  Trust in institutions: Older adults seek reassurance from trusted organisations like the NHS.  Data sharing benefits: Sharing data with clinical teams improves collaboration and communication.  Privacy and peer sharing: Respect for privacy encourages users to share data with peer groups for support.  Stigma and design issues: Clinical-looking or disability-friendly devices increase stigma and reduce adoption.  Diversity and inclusion gaps: Current technology development lacks diversity and inclusivity. | Small panel size (14 experts)  Lack of lived experience of dementia  Online-only due to pandemic  Did not directly consider multimorbidity scenarios |
| [[63]](https://journals.sagepub.com/doi/10.1177/20552076251321698?url_ver=Z39.88-2003&rfr_id=ori:rid:crossref.org&rfr_dat=cr_pub%20%200pubmed) | Mwaka ES, Bazzeketa D, Mirembe J, Emoru RD, Twimukye A, Kivumbi A. Barriers to and enhancement of the utilization of digital mental health interventions in low-resource settings: perceptions of young people in Uganda. Digital Health. 2025;11:20552076251321698. doi:10.1177/20552076251321698 | Uganda – Participants drawn from nine public and private universities; authors affiliated with institutions in Kampala. | Young people aged 18–25 years; university students from diverse academic programmes who owned smartphones and had considered seeking help for mental health problems. | Explored young people’s perceptions of barriers and solutions to digital mental health (DMH) in low-resource settings using an inductive thematic approach | 50 young people (62% male, median age 24, range 21-25; 54% medical students; enrolled from 9 universities) | Six face-to-face focus group discussions, each with 8-9 participants, using a semi-structured guide co-developed with the EMDIYA network. Sessions were audio-recorded, transcribed verbatim, coded in NVivo and analysed thematically | Awareness of DMH solutions: Young people are aware of DMH through apps, podcasts, blogs and social media.  Preference for human touch and consistency: Users want apps that combine AI with real human interaction and long-term support.  Connectivity and device barriers: Lack of smartphones, internet-enabled gadgets and poor connectivity hinder use.  Inequitable access and literacy barriers: DMH mainly benefits urban, literate groups, excluding rural and illiterate populations.  Financial barriers: High cost of apps and payment methods limit adoption.  Stigma and negative perceptions: Cultural beliefs, fear of judgment and misconceptions hinder openness.  App-related challenges: Poor usability, irrelevant content and lack of localisation discourage use.  Privacy and confidentiality concerns: Users worry about personal data safety and trust in app providers.  Public engagement and awareness: Need for campaigns to educate and create awareness of DMH.  Government role and regulation: More funding, regulation and toll-free access are needed.  Training health workers: Capacity building at all levels, especially village health teams.  Preference for free and affordable apps: Young people want free, simple and accessible apps. | Sample limited to university students  Possible moderator and participant bias  Social desirability bias risk  Snowball sampling may bias recruitment |
| [[64]](https://journals.sagepub.com/doi/10.1177/20552076211064406?url_ver=Z39.88-2003&rfr_id=ori:rid:crossref.org&rfr_dat=cr_pub%20%200pubmed) | Namatovu HK, Oyana TJ, Sol HG. Barriers to eHealth adoption in routine antenatal care practices: perspectives of expectant mothers in Uganda—a qualitative study using the unified theory of acceptance and use of technology model. Digit Health. 2021;7:20552076211064406. doi:10.1177/20552076211064406 | Uganda – Kampala, including low-income suburbs/parishes served by six selected health facilities. | Expectant mothers aged 18–49 years residing in Kampala and surrounding areas, able to speak English or Luganda. | Semi-structured face-to-face interviews based on Unified Theory of Acceptance and Use of Technology (UTAUT) constructs (performance expectancy, effort expectancy, social influence, facilitating conditions, behavioural intention); interpretive epistemology with inductive reasoning under critical realism | 115 expectant mothers aged 18-49, selected from six health facilities in Kampala, Uganda | Conducted at participants’ homes in English or Luganda; average 45 minutes; audio-recorded and transcribed; thematic or template analysis using AQUAD 7; coding validated by multiple researchers and participants | Limited perceived utility: Mothers saw little value in current e-health systems for maternal health.  Usability issues: Some applications were difficult to navigate with unfriendly interfaces.  Lack of spousal support: Husbands discouraged use of e-health.  No user involvement: Systems were developed without engaging mothers, leading to low uptake.  Lack of direct link to providers: Systems failed to connect mothers to doctors.  No training before use: Absence of training created barriers to adoption.  Lack of technical support: Users were frustrated when systems failed.  Irresponsive healthcare providers: Providers lacked time, motivation or digital skills.  High data costs: Internet bundles were unaffordable for most mothers.  Poor internet connectivity: Slow and unreliable internet hindered usage.  Electricity challenges: Load-shedding and high tariffs limited device usage.  Lack of devices: Many mothers lacked smartphones or computers.  Illiteracy: Low literacy and language barriers prevented adoption.  Limited knowledge of benefits: Mothers did not see the value of e-health for antenatal care.  Misconceptions about systems: Users doubted sustainability due to failed pilots.  Lack of trust: Fear of privacy breaches reduced willingness to adopt. | High dropout of participants after initial consent  Barriers on healthcare provider side not examined  Narrow focus on barriers rather than facilitators |
| [[65]](https://onlinelibrary.wiley.com/doi/10.1155/2024/5578056) | Nigatu AM, Yilma TM, Gezie LD, Gebrewold Y, Gullslett MK, Mengiste SA, et al. Barriers and facilitators experienced during the implementation of web-based teleradiology system in public hospitals of the Northwest Ethiopia: an interpretive description study. Int J Telemed Appl. 2024;2024:5578056. doi:10.1155/2024/5578056 | Ethiopia – Public hospitals in the South Gondar Zone, Northwest Ethiopia (Amhara Regional State). | Healthcare professionals and administrators involved in the implementation of a web-based teleradiology system, including hospital managers, physicians, surgeons, and radiologists. | Interpretive description approach with abductive thematic analysis; guided by the technology-organisation-environment (TOE) framework; reflexivity applied to address the researcher’s insider role | 17 participants (hospital managers, physicians, emergency surgeons, radiologists; 15 male, 2 female; median age 30) | Semi-structured in-depth interviews (average 55 minutes), in Amharic then translated to English; pilot-tested guide; open-ended and probe questions; audio-recorded, transcribed, anonymised; analysed in Open Code 4.02 | Technological barriers: Poor ICT infrastructure, unstable networks, server downtime and lack of notifications hindered system performance.  Need for technical support: Lack of timely onsite experts disrupted system use.  Organisational barriers: Inadequate funding, power instability, shortage of radiologists and technical staff and weak administrative support threatened sustainability.  Environmental barriers: Lack of regulations, unclear system ownership and weak institutionalisation created sustainability risks.  Individual barriers: Low awareness, lack of readiness, limited technical skills and resistance to adoption impeded use.  Workflow barriers: Delays in consultations and report submissions, and poor system integration caused inefficiency.  Improved service efficiency: Teleradiology enhanced quality, reduced misdiagnosis and saved time and costs.  Accessibility and collaboration: Web-based access enabled remote consultation, collaboration and continuous learning.  Trusted ecosystem: Standardised reporting, confidentiality and user trust promoted adoption. | Perspectives mainly from managers, not a full healthcare professional spectrum  Patient perspective absent  Context-specific, limited generalisability |
| [[66]](https://www.mdpi.com/1660-4601/19/20/13714) | O’Connor SR, Treanor C, Ward E, Wickens RA, O’Connell A, Culliford LA, et al. Patient acceptability of home monitoring for neovascular age-related macular degeneration reactivation: a qualitative study. Int J Environ Res Public Health. 2022;19(20):13714. doi:10.3390/ijerph192013714 | United Kingdom – Recruitment conducted at five MONARCH study sites across the UK. | Adults with neovascular age-related macular degeneration enrolled in the MONARCH study, including patients, close family members or informal carers, and healthcare professionals involved in training and support for home monitoring. | Semi-structured interviews exploring acceptability and adherence to weekly home monitoring for neovascular age-related macular degeneration; analysis guided by Unified Theory of Acceptance and Use of Technology (UTAUT), Technology Acceptance Model (TAM), Theoretical Framework of Acceptability (TFA) and Senior Technology Acceptance Model (STAM) | 78 patients (63 regular testers, 14 irregular, 1 non-tester), 11 carers, 9 healthcare professionals → total 98 participants | Conducted October 2018-September 2020; average 36 minutes; in homes (51), clinics (4), telephone (45); audio-recorded, transcribed, coded with NVivo; directed content analysis combining deductive and inductive coding | Ownership and control: Patients saw home monitoring as giving them personal control over vision health.  Reduced clinic visits: Home monitoring is seen as beneficial if it could reduce clinic appointments.  Barriers in care pathways: Concerns about the inability to access clinics despite home monitoring.  Overcoming technology hesitancy: Some saw home monitoring as necessary despite unfamiliarity with devices.  Need for refresher training: Patients struggled to recall training without refreshers.  Engagement via feedback: Tests with feedback were preferred as motivating.  Habits and routine: Regular home monitoring is feasible when integrated into routines and reminders.  Family and carer support: Relatives often provided technical and motivational help.  Practical barriers: Device setup, testing conditions or uncertainty about data transmission caused frustration.  Health and social barriers: Tremors, fatigue, caregiving responsibilities disrupted home monitoring. | Mix of phone vs face-to-face interviews may have influenced data  Sample leaned toward adherent or early adopters  Results may not capture perspectives of those resistant to home monitoring |
| [[67]](https://www.mdpi.com/1999-5903/15/6/191) | Patrickson B, Musker M, Thorpe D, van Kasteren Y, Bidargaddi N, Consumer and Carer Advisory Group (CCAG). In-depth co-design of mental health monitoring technologies by people with lived experience. Future Internet. 2023;15(6):191. doi:10.3390/fi15060191 | Australia – Work conducted within the Australian context, including consultation in Adelaide, South Australia. | People with lived experience as mental health care consumers and/or informal carers, including individuals with chronic mental illness and varying levels of engagement with the mental health system. | In-depth consumer and carer co-design consultation over 10 focus group workshops (2 hours each) between 2019-2021; grounded qualitative content analysis and thematic synthesis | A varying number of consumers and carers per workshop (documented in the appendix; participants fluctuated across sessions) | Facilitated focus group workshops (face to face or virtual), recorded, transcribed, thematically analysed. Iterative reflective analysis and validation with participants | Limited access to devices and data: Consumers faced barriers from a lack of devices and affordable data.  Limited access to trusted clinicians: Discontinuity of care reduced confidence in systems.  Need for personalisation and adaptability: Systems must adapt to unique conditions, medication regimes and digital literacy levels.  Consumer disempowerment and surveillance concerns: Mandatory treatment led to concealment and resistance.  Importance of ongoing consent: Consumers emphasised transparent, explicit, ongoing consent for digital monitoring.  Carer involvement and boundaries: Carers wanted involvement but highlighted the need for clear consent and boundaries.  Poor information on medication effects: Consumers lacked access to clear information on side effects and interactions.  Self-monitoring challenges: Risk of harmful over-monitoring, sustainability issues and mixed feelings about carer involvement.  Value of peer and carer support: Carer contributions and peer involvement strengthen wellness and recovery.  Need for humanised care: Consumers wanted systems that respect dignity and avoid objectification as “data.”  Digital therapeutic relationships: Technology should enhance collaboration, not enforce compliance. | Small, high-functioning panel may not represent wider population  Need broader demographic diversity for co-design  Context limited to Australian mental healthcare system  Consumer-carer dynamics complex; requires sensitive boundary setting |
| [[68]](https://www.frontiersin.org/journals/digital-health/articles/10.3389/fdgth.2024.1275569/full) | Posselt J, Baumann E, Dierks ML. A qualitative interview study of patients’ attitudes towards and intention to use digital interventions for depressive disorders on prescription. Front Digit Health. 2024;6:1275569. doi:10.3389/fdgth.2024.1275569 | Germany – Primary care setting provided by general practitioners. | Adult patients (≥18 years) with mild or moderate depressive disorders receiving primary care and not currently undergoing specialist or psychotherapeutic treatment. | Semi-structured interviews exploring acceptance of digital therapeutics (DTx) for depressive disorders; analysis guided by UTAUT determinants: performance expectancy, effort expectancy, social influence (GPs) and behavioural intention | 17 patients with mild or moderate depression (11 female, 6 male; depression duration: <1 year = 3, 1-5 years = 3, >10 years = 11; varied education and urban or suburban residence) | Conducted January-June 2022 in German; 24-42 minutes; stimuli video with DTx examples; audio-recorded, transcribed verbatim, anonymised; coded using MAXQDA; intercoder reliability checks; ethical approval from Hannover Medical University | Bridging waiting times: Patients valued DTx for coping and self-management during long waits for therapy.  Evidence-based and trustworthy information: DTx is considered more reliable than generic internet sources.  Access to professional help: Patients expected chat or communication tools to provide guidance.  Anonymity and stigma reduction: DTx is valued for privacy in mental health contexts.  Not a substitute for psychotherapy: DTx seen as helpful but less effective than in-person care.  Concerns of negative effects: Digital tools may trigger or worsen mental health symptoms.  Flexibility and convenience: Patients valued low-threshold access compared to traditional therapy.  Digital literacy and access issues: Less tech-savvy groups may face barriers.  GP trust and recommendations: GP advice strongly influenced DTx uptake for some patients.  GP limitations: Some patients felt GPs lacked knowledge or empathy about digital tools.  Structural role of GPs: GPs are seen as gatekeepers for prescriptions, even if not always consulted.  Intention varies by disease stage: Interest in DTx depended on health status and coping stage.  Long-term experience reduces need: Patients with long therapy history saw little added value. | Intention to use studied, not actual usage  Possible selection bias toward tech-affine patients  Language translation may affect nuance  Small, exploratory sample limits generalisability |
| [[69]](https://onlinelibrary.wiley.com/doi/10.1111/nhs.12939) | Rochmawati E, Kamilah F, Iskandar AC. Acceptance of e-health technology among older people: a qualitative study. Nurs Health Sci. 2022;24(2):437-46. doi:10.1111/nhs.12939 | Indonesia – Suburban primary clinic (FKTP) in central Indonesia. | Adults over 45 years of age with diabetes or hypertension receiving primary healthcare services. | Exploratory qualitative method using semi-structured interviews to explore perceptions of older people regarding health technology acceptance | 11 participants (mean age 66.9; 63.3% female; 45.5% diabetes, 54.5% hypertension; mixed education levels) | Semi-structured, in-depth, face-to-face interviews (36-102 minutes, average 65 minutes), conducted at homes (9) and clinic (2); interviews audio-recorded, transcribed, analysed with NVivo 20; member checking and peer debriefing ensured trustworthiness | Demand for care and monitoring: Older adults recognised their health problems and wanted ongoing monitoring.  Seeking health information: Participants actively searched information from Google, YouTube, radio, newspapers and doctors.  Preference for face-to-face interaction: Many resisted digital health and preferred direct clinic visits.  Limited digital skills: Many older adults lacked the ability to use apps and smartphones.  Openness to technology: Participants acknowledged potential benefits of devices like smart bands and apps.  Family support: Children and caregivers played key roles in teaching and helping older adults use technology.  Comfort and ease of use: Devices must be physically comfortable, adjustable and simple to use.  Design preferences: Older adults preferred appropriate colours, large fonts and simple layouts. | Exclusion of perspectives of caregivers and healthcare professionals  Possible loss of meaning due to translation from Bahasa or Javanese  Simplification of concepts during interviews may alter meaning  Risk of incorrect translation reducing validity  Limited generalisability of findings |
| [[70]](https://onlinelibrary.wiley.com/doi/10.1155/2024/2667732) | Saeed H, Scahill S, Kim J, Moyaen R, Natarajan D, Soga A, et al. Pharmacist perceptions and future scope of telepharmacy in New Zealand: a qualitative exploration. Int J Telemed Appl. 2024;2024:2667732. doi:10.1155/2024/2667732 | New Zealand. | Registered and practicing pharmacists in New Zealand. | Semi-structured interviews grounded in critical realism, exploring perceptions of telepharmacy, benefits, barriers and scope of practice; themes analysed using inductive thematic analysis, then mapped to the Clinical Adoption Framework (CAF) | 23 registered pharmacists (69.6% female; 82.6% under 40; 69.6% Asian; 65.2% <10 years practice; 69.6% community pharmacists) | Semi-structured interviews via Zoom (July-August 2022), audio recorded and transcribed; pilot interviews conducted but excluded; demographic survey collected; analysed with NVivo 13 using inductive and deductive thematic analysis | Technical barriers: Setup time, glitches, cybersecurity and privacy issues reduced efficiency.  Variability in use: Exposure and uptake differed by pharmacy type and business model.  Expanded service potential: Telepharmacy enabled remote consultations, refills, chart reviews and counselling.  Accessibility and convenience: Useful for mobility-limited patients and those isolating.  Limits of remote care: Lack of physical exams and non-verbal cues restricted effectiveness.  Rapport and empathy concerns: Harder to build trust virtually compared to face-to-face.  Patient barriers: Device, internet, literacy, disability and language issues limited adoption.  Resistance to change: Older pharmacists and managers preferred traditional methods.  Strategic planning needed: Implementation required feasibility studies, feedback and standardisation.  Infrastructure gaps: Need for a unified health database, better platforms and user-friendly systems.  Workforce and training needs: Staff shortages, workload and lack of telehealth training hindered use.  Legal and policy barriers: Lack of supportive laws, unclear policies and inadequate guidance.  Governance and support gaps: Lack of guidance and poor communication from the government created challenges.  Funding needs: Lack of remuneration and financial support threatened sustainability.  Value for public health: Telepharmacy could reduce disease spread and expand rural care.  Societal shift: COVID-19 normalised telehealth and increased acceptance. | Convenience sampling may cause bias and limit representativeness  Self-selection bias: participants are more interested in telepharmacy  Demographic skew: young, Asian, early-career pharmacists dominate  Limited exploration of rural or underserved contexts  Excluded perspectives of policymakers and leaders  CAF did not cover all relevant categories |
| [[71]](https://bmcpublichealth.biomedcentral.com/articles/10.1186/s12889-024-20265-7) | Sampene AK, Li C, Wiredu J. Unravelling the shift: exploring consumers’ adoption or resistance of e-pharmacy through behavioural reasoning theory. BMC Public Health. 2024;24:2789. doi:10.1186/s12889-024-20265-7 | South Africa – Western Cape region, including George, Laingsberg, and Mossel Bay local municipalities. | Consumers with prior experience using e-pharmacy services residing in the Western Cape region of South Africa. | Qualitative design applying behavioural reasoning theory (BRT) to explore reasons for (RFA) and reasons against (RAA) e-pharmacy adoption in South Africa; thematic analysis with Atlas.ti and NVivo | 28 participants (16 adopters, 12 non-adopters; diverse in age, gender, income, education and geography; majority male 58% and Black African 64.3%) | In-depth interviews across three municipalities in Western Cape (George, Laingsberg, Mossel Bay). Open-ended questions, audio recorded, notes taken. Data analysed with Atlas.ti and NVivo; saturation achieved | Convenience and accessibility: e-pharmacy helped overcome geographic barriers and busy schedules.  Prescription management: Digital platforms simplified managing chronic medications.  Cost and affordability: Lower prices, discounts and transparency attracted adoption.  Logistics and timely delivery: Doorstep delivery and tracking features enhanced confidence.  Trust and security concerns: Fear of counterfeit medicines and loss of pharmacist interaction reduced adoption.  Regulatory and legal uncertainties: Absence of clear frameworks created apprehension.  Lack of internet access: Poor connectivity and high costs hindered rural users.  Privacy concerns: Fears of hacking and data breaches reduced trust. | Limited representation of South Africa’s cultural and socio-economic diversity  Reliance on self-reported data may cause bias  Need for broader and more representative sampling  Lack of longitudinal data to capture evolving attitudes  Need for comparative cross-country studies |
| [[72]](https://mhealth.jmir.org/2024/1/e48345) | Schroeder T, Haug M, Georgiou A, Seaman K, Gewald H. Evidence of how physicians and their patients adopt mHealth apps in Germany: exploratory qualitative study. JMIR Mhealth Uhealth. 2024;12:e48345. doi:10.2196/48345 | Germany – Data collected in Germany. | Physicians who prescribe DiGAs / mHealth apps; potential patients aged 50 years and older (older users / potential users of a DiGA). | Exploratory qualitative design using semi-structured interviews with physicians and patients; guided by constructs from unified theory of acceptance and use of technology (UTAUT) and health belief model (HBM); data analysed with interpretative phenomenological analysis (IPA) and thematic coding using NVivo 10 | 28 physicians (various specialties, age 35-68, male and female) and 30 patients (all ≥50 years, balanced gender, diverse health backgrounds) | Semi-structured interviews (20-45 minutes), conducted in German, recorded, transcribed, translated into English; iterative coding framework developed inductively, validated by research team | Lack of information for physicians: Doctors lacked knowledge about DiGAs and their benefits, creating mistrust.  Lack of IT resources: Poor digital infrastructure limited prescription potential.  Lack of certification: Absence of official medical certification reduced physician confidence.  Lack of physician involvement: Doctors wanted more engagement post-prescription and in development.  Lack of financial incentives: Low reimbursement discouraged doctors from prescribing DiGAs.  Physician digital literacy divide: Doctors varied in openness and competence with digital tools.  Patient lack of information: Many patients had never heard of DiGAs and lacked awareness.  Self-efficacy (tech and health): Patients expressed both confidence in using apps and in managing health.  Perceived threat: Disease severity and susceptibility motivated adoption.  Trust in physician: Doctor-patient trust was a precondition for considering DiGAs  Privacy and control: Patients wanted autonomy in data sharing but trusted certified systems.  Outcome expectations: Patients valued reduced visits, better monitoring and more information.  Facilitating conditions: Technical and health support were important for sustained use.  Physician ageism and bias: Doctors often exclude older patients, assuming low digital skills. | Lack of generalisability due to exploratory design  Many physicians had limited or no experience with DiGAs  Participants’ perceptions may shift over time as DiGAs become mainstream  Need for longitudinal and cross-national studies  Second-order adoption (physicians as gatekeepers) and digital ageism need more research |
| [[73]](https://journals.sagepub.com/doi/10.1177/20552076251326160) | Sezgin E, Jackson DI, Kaufman K, Skeens MA, Gerhardt CA, Moscato E. Perceptions about the use of virtual assistants for seeking health information among caregivers of young childhood cancer survivors. Digital Health. 2025;11:20552076251326160. doi:10.1177/20552076251326160 | United States – Caregivers recruited from a large pediatric academic medical center in the Midwest, including metropolitan, rural, and Appalachian regions. | Caregivers of young childhood cancer survivors, including biological parents, grandparents, adoptive parents, and legal guardians; some from underserved communities (rural/Appalachian). | Semi-structured interviews and focus groups with caregivers of young childhood cancer survivors; caregivers interacted with a prototype web-based virtual assistant (VA) to provide feedback on usability, utility and feasibility | 10 caregivers (9 female, 1 male; mostly married; diverse education and income; 9 White, 1 Black; 1 Hispanic or Latino; from metropolitan, rural and Appalachian areas) | Focus group (n=7) and interviews (n=3), 30-60 minutes; participants tested VA through scenarios (late effects, return to school, history, emotional impact), asked questions, gave feedback; audio recorded, transcribed, coded using NVivo with in-vivo coding; thematic analysis (Braun and Clarke) | Flexible interaction modes: Caregivers preferred multimodal (voice and text) options depending on context.  Organisational support: Caregivers used smart technologies for reminders, organisation and scheduling.  Need for personalisation and memory: Caregivers wanted VAs to recall past queries and personalise responses.  Language and accessibility: Multi-language options and educational support for low-literacy users are valued.  Search fatigue and cognitive burden: Caregivers were overwhelmed by excessive online searching.  Desire for specific, evidence-based content: Caregivers wanted niche, accurate oncology-related answers.  Reducing burden: VAs are perceived as tools to reduce exhaustion and provide quick answers.  Integration with health systems: Caregivers trusted VAs linked to hospitals and clinicians.  Data security and confidentiality: Caregivers emphasised secure, private VA systems.  Ethical guidance and escalation: VAs should direct users when professional consultation is needed.  Risk of over-reliance: Concerns that caregivers may trust VA guidance too much without consulting doctors.  Rural and underserved needs: Rural caregivers saw greater value for access and learning. | Participants may be biased towards technology (already involved in advisory boards, tech-inclined)  Lack of diverse representation (racial or ethnic minorities under-represented)  Focused only on caregivers, excluding healthcare providers’ perspectives  Short prototype interaction time limited depth of insights  Risk of social desirability bias in responses |
| [[74]](https://www.researchgate.net/publication/336011768_Exploring_the_barriers_and_facilitators_for_the_use_of_digital_health_technologies_for_the_management_of_COPD_A_qualitative_study_of_clinician_perceptions) | Slevin P, Kessie T, Cullen J, Butler MW, Donnelly SC, Caulfield B. Exploring the barriers and facilitators for the use of digital health technologies for the management of COPD: a qualitative study of clinician perceptions. QJM. 2020;113(3):163-72. doi:10.1093/qjmed/hcz241 | Ireland – Recruitment from two Dublin university hospitals and general practitioner clinics in the greater Dublin area. | Practicing healthcare professionals providing care to COPD patients, including general practitioners and hospital-based respiratory clinicians. | One-to-one semi-structured interviews with healthcare professionals providing care to COPD patients; thematic analysis using NVivo 12 | 32 healthcare professionals (8 GPs, 4 respiratory specialist nurses, 4 physiotherapists, 8 non-consultant hospital doctors, 8 respiratory consultant physicians) | Semi-structured interviews (30-60 minutes), audio recorded, transcribed verbatim, anonymised; thematic analysis conducted inductively, codebook developed iteratively; data saturation confirmed | Data quality concerns: Clinicians doubted the accuracy and calibration of patient devices.  Need for evidence-based care: Lack of strong clinical evidence and guidelines prevented adoption.  Resource constraints: Limited staff, time and infrastructure hindered data integration.  Patient digital literacy barriers: Low digital literacy and cognitive issues created risks.  Training and education as facilitator: Pulmonary rehab and outreach programmes can provide digital health technology training.  Value of initial conversations: Clinician-patient discussions at the setup stage are critical for engagement.  Improving healthcare professionals’ digital literacy: Clinicians emphasised their own need for digital training.  Personalised prescribing: Suitability of digital tools should depend on individual patient characteristics.  Human contact still needed: Some patients prefer direct clinician interaction over devices. | Limited generalisability due to sample size and local context  Resource and system variability across hospitals may affect transferability  Need for more longitudinal and co-design studies  Current evidence for digital health technology effectiveness in COPD is still lacking |
| [[75]](https://formative.jmir.org/2022/1/e28895/) | Tennant R, Allana S, Mercer K, Burns CM. Exploring the experiences of family caregivers of children with special health care needs to inform the design of digital health systems: formative qualitative study. JMIR Form Res. 2022;6(1):e28895. doi:10.2196/28895 | North America – Conducted remotely in Canada and the United States. | Family caregivers (aged ≥18 years) of children with special health care needs receiving home care; children defined as having chronic conditions, mental health issues, medication-related problems, and/or social vulnerability. | Semi-structured interviews guided by storytelling principles in narrative medicine; inductive thematic analysis conducted with NVivo 12 and Excel | 7 family caregivers (all female, aged 33-40; 2 from the USA, 5 from Canada; caregiving experience 4-18 years; children with complex needs using ventilators, feeding tubes, orthotics, wheelchairs, etc.) | Audio-recorded interviews conducted remotely during COVID-19 (June-September 2020); field notes completed; data transcribed, anonymised, coded; triangulation by multiple coders and researchers; data saturation reached after 7 interviews | Continuous learning: Caregivers constantly sought knowledge from professionals, media, peers and self-learning.  Burden of documentation: Caregivers felt pressure to maintain extensive paper-based records, which was exhausting.  Desire for digital solutions: Caregivers expressed interest in digitising but found apps unusable or rigid.  Information sharing pressure: Caregivers struggled to filter and deliver the right information at the right time.  Training new caregivers: Teaching new caregivers consumed significant time and effort.  Communication with schools: Caregivers struggled with inconsistent methods and unsafe practices.  Bureaucratic burden: Paperwork, payroll and hiring processes added to stress.  Pandemic impact: COVID-19 reduced support services and increased caregiver isolation.  Design recommendations: Tools must reduce duplication, adapt to changing needs, support customisation and be intuitive. | Small, exploratory sample limits generalisability  Limited US representation compared with Canadian participants  COVID-19 context influenced recruitment and caregiving practices  Exclusion of financial and system-level impacts in depth  Lack of diagnosis-specific or long-term implications analysis |
| [[76]](https://nursing.jmir.org/2023/1/e39815) | Vercell A, Taylor S, Yorke J, Dowding D. Assessing the acceptability of home blood monitoring for patients with cancer who are receiving systemic anticancer therapy from a patient, caregiver, and clinician perspective: focus group and interview study. JMIR Nurs. 2023;6:e39815. doi:10.2196/39815 | United Kingdom – Recruitment from The Christie NHS Foundation Trust, Manchester (single NHS site). | Patients receiving systemic anticancer therapy aged ≥16 years with adequate English proficiency and treatment within past 12 months; caregivers of eligible patients; healthcare professionals employed at the site including specialist registrars, fellows, consultants, nurses, and lab/IT experts. | Focus groups and semi-structured interviews with patients, caregivers and healthcare professionals to explore the acceptability of a home blood monitoring device for systemic anticancer therapy; analysis guided by unified theory of acceptance and use of technology (UTAUT) | 47 participants (23 patients, 6 caregivers, 18 healthcare professionals) | Conducted over 19 months (May 2019-December 2020). Three patient or caregiver focus groups, two healthcare professional focus groups and 12 individual interviews. Sessions included prototype device testing (capillary sample, cuvette insertion). Discussions covered self-testing, expectations, responsibilities and result reporting. Audio-recorded, transcribed, coded using framework method | Desire for self-management and control: Patients valued access to results to manage their care.  Mixed views on viewing results: Some patients valued seeing results, while others felt anxious.  Convenience and reduced hospital burden: Home monitoring is seen as more convenient than hospital visits.  Fear and stress around venous testing: Finger-prick testing preferred over venous draws.  Ease of use and training: Device adoption depends on simplicity, training and feedback.  Device design and stigma: Patients wanted discreet, non-clinical devices.  Safety and backup systems: Concerns about device failure, out-of-range results and support availability.  Social influence and equity of access: Concerns about elderly, digitally naïve or unsupported patients.  Integration into care pathways: Importance of real-time reporting, clinician review time and interoperability.  Need for human contact: Some patients still preferred in-person reassurance and clinician interpretation. | Single-site study limits generalisability  Recruitment biased towards likely adopters  Pandemic impacted recruitment and caregiver involvement  Participants may already be open to home monitoring |
| [[77]](https://aisel.aisnet.org/icis2024/ishealthcare/ishealthcare/18/?utm_source=aisel.aisnet.org%2Ficis2024%2Fishealthcare%2Fishealthcare%2F18&utm_medium=PDF&utm_campaign=PDFCoverPages) | Weeratunge SK, Bain C, Burstein F, Evans J. Can patient portals support digital health equity for culturally and linguistically diverse communities in Victoria: an exploratory study. In: ICIS 2024 Proceedings. 2024. Available from: <https://aisel.aisnet.org/icis2024/ishealthcare/ishealthcare/18> | Australia – Victorian healthcare system; Melbourne/Victoria context. | Culturally and Linguistically Diverse (CALD) health consumers in Victoria; participants born overseas or with parents/grandparents born overseas; had experience with the Victorian healthcare system. | Semi-structured interviews with culturally and linguistically diverse (CALD) participants in Victoria, analysed inductively and deductively using the digital health equity framework (DHEF) | 14 CALD participants (diverse cultural backgrounds; majority Sri Lankan, others from Vietnam, China, Iran, Saudi Arabia, East Timor, Georgia; 6 over 60 years old; varied English or digital literacy levels; mix of Medicare and private insurance) | Semi-structured interviews (45-60 minutes); 8 in-person, 6 via Zoom. Audio recorded, transcribed, and thematically analysed with inductive and deductive coding (Braun and Clarke, 2012; Proudfoot, 2023). Data saturation at 13 interviews. Pilot test conducted for refinement | Low awareness and limited use: CALD participants had little exposure to patient portals, even when available.  Digital literacy gaps: Seniors expressed difficulty with digital tools, while younger people were more confident.  Privacy concerns: Participants worried about sensitive information being online.  Language barriers: Limited English proficiency and lack of translation support restricted use.  Preference for in-person care: Many valued the physical presence of clinicians despite portal benefits.  Family and carer support: Many relied on relatives to navigate systems due to language or tech barriers.  Organisational barriers: Hospitals often did not offer portals even when available.  Cultural and social norms: Some preferred clinicians of the same cultural background.  Policy and system gaps: Lack of mandates and weak evaluation limited portal rollout.  Cost burden for migrants: Non-Medicare patients struggled with costs, which portals could potentially reduce.  Desire for features: Participants suggested linking portals to MyGov, multilingual support and immediate benefits. | Small sample, exploratory design  Lack of provider or implementer perspectives  Under-representation of some CALD groups  Potential bias from recruitment method  Limited prior portal use among participants restricts insights |
| [[78]](https://humanfactors.jmir.org/2024/1/e51972) | Zainal H, Hui XX, Thumboo J, Fong W, Yong FK. Patients’ expectations of doctors’ clinical competencies in the digital health care era: qualitative semistructured interview study among patients. JMIR Hum Factors. 2024;11:e51972. doi:10.2196/51972 | Singapore – Outpatient clinics of a public healthcare cluster. | Patients from outpatient clinics with various chronic conditions (cancer, cardiovascular disease, diabetes, kidney, neurological, autoimmune); spanning age bands 20–39, 40–59, ≥60; diverse ethnic, socioeconomic, and educational backgrounds. | Ethnographic qualitative design with individual semi-structured interviews; inductive thematic analysis following Braun and Clarke’s six-step process | 25 patients (14 male, 11 female; ages 20-39 = 8, 40-59 = 10, ≥60 = 7; varied ethnicity, socio-economic status, education; chronic conditions such as cancer, diabetes, kidney disease, gout, rheumatoid arthritis, neurological disease) | 22 interviews via Zoom, 3 in-person; ~40 minutes each; pilot-tested with 5 patients; purposive sampling for diversity of conditions and ages; audio recorded, transcribed, anonymised; thematic analysis performed independently by researchers with consensus | Guidance and collaboration with apps: Patients wanted doctors to guide them on safe apps and work as partners.  Doctor knowledge of apps: Patients expected doctors to be familiar with health apps like HealthHub.  Doctor engagement with device data: Patients wanted doctors to review and explain wearable or device results.  Data privacy and cybersecurity: Patients worried about the insecure sharing of scans via WhatsApp.  Teleconsultation attention and safety: Patients expected undivided attention and safe, accurate care.  Escalation and holistic assessment: Doctors should know when to refer and assess patients holistically.  Efficiency vs eye contact: Patients valued efficiency over eye contact when using EHRs.  Empathy regardless of technology: Patients emphasised that empathy should remain central.  Younger patients expect AI competence: Younger participants wanted doctors trained in AI for accuracy.  Older patients concerned about reliability: Older participants distrusted digital health technologies and preferred doctor oversight. | Small sample size limits generalisability  Selection bias possible from recruitment strategy  Lack of language diversity (all English-speaking)  Exclusion of some emerging technologies (e.g. ChatGPT)  Findings may be context-specific to Singapore’s system |
| [[79]](https://www.liebertpub.com/doi/10.1089/tmj.2022.0527?url_ver=Z39.88-2003&rfr_id=ori%3Arid%3Acrossref.org&rfr_dat=cr_pub++0pubmed) | Almathami HKY, Win KT, Vlahu-Gjorgievska E. Empirical evidence of internal and external factors influencing users’ motivation toward teleconsultation use. Telemed J E Health. 2024;30(1):141-56. doi:10.1089/tmj.2022.0527 | Saudi Arabia – Users of the Ministry of Health teleconsultation system (“Sehha application”). | Consumers aged >18 years using the Sehha application; reported demographics included gender, age group, education, employment status, and province. | Online cross-sectional survey based on self-determination theory; constructs mapped into attitude, performance expectancy (PE), subjective norm (SN), external facilitating conditions (EFC), internal facilitating conditions (IFC), autonomy, competence, relatedness, motivation and future motivation | 471 participants (59% female; majority aged 25-34; 61% bachelor’s degree; 40% employed full-time; 76% from Mecca province) | Non-random voluntary participation via social media invitation; validated 51-item questionnaire on 7-point Likert scale; available in English and Arabic; data analysed with SPSS 27 and SmartPLS 3.3 for descriptive and multigroup analysis | Saving time and cost: Teleconsultation reduced travel, waiting and consultation expenses.  Accessibility and convenience: Patients valued anytime, anywhere access.  Social influence: Family, friends and doctors’ recommendations motivated use.  Ease of use: Users found the application simple and similar to other familiar apps.  Internet and device access: High-speed internet, quiet spaces and smartphones were essential.  Training and orientation: Orientation and guidance improved adoption.  Trust in provider and data security: Users trusted the Ministry of Health as the provider.  Autonomy and control: Users valued freedom to choose consultation mode and control data.  Competence and communication: Patients felt capable of explaining health conditions online.  Relatedness and connection: Users felt connected to doctors and peers.  Motivation from recommendations and health needs: Users were motivated by family, doctors and the desire to understand their health condition.  Future motivation: Strong desire to connect with the same or preferred doctor for follow-up.  Demographic neutrality: Age, gender, education and employment did not affect motivation. | Convenience sampling may exclude non-social-media users  Reliance on self-reported measures introduces bias  Free public healthcare context in Saudi Arabia limits transferability |
| [[80]](https://www.emerald.com/tqm/article-abstract/34/6/1772/377388/Toward-digital-transformation-in-healthcare-a?redirectedFrom=fulltext) | Binci D, Palozzi G, Scafarto F. Toward digital transformation in healthcare: a framework for remote monitoring adoption. TQMJ. 2022;34(6):1772-99. doi:10.1108/TQM-04-2021-0109 | Italy – Cardiology Department, Casilino General Hospital, Rome. | Medical staff involved in the remote monitoring project; physicians provided perspectives on patient attitudes toward remote monitoring for chronic cardiac disease management. | Single-case study at Casilino General Hospital (Rome) using grounded theory to frame remote monitoring (RM) adoption for chronic cardiac disease management; applied input-process-output (I-P-O) model | 12 key informants (head of cardiology, telemedicine coordinators, general physicians, coordinators of surgical or research activities; average 17 years cardiology experience, 8 years telemedicine) | Open-ended interviews (average 1 hour), verbatim transcription, NVivo 12 coding; ~150 raw concepts grouped into 5 main themes; supplemented with ≥200 hours observation of patient-clinician interactions and secondary documents (internal guidelines, Ministry of Health and Heart Rhythm Society protocols) | RM enablers: Clinicians were motivated by efficiency, awareness of RM benefits and improved process quality.  Digital culture: Age, digital skills and patient literacy shaped adoption attitudes.  Structure flexibility: Adoption required horizontal integration, coordination and flexible organisational structures.  Reaction to change: Resistance arose from fear, lack of skills and data burden, but could be reduced by support and clarity.  RM outcomes: Adoption improved clinical decision-making, early detection and patient quality of life. | Qualitative design limits replication and is interpretive  Mono-centric study limits generalisability  Larger-scale and multi-site research needed |
| [[81]](https://www.emerald.com/jstp/article-abstract/32/2/105/254615/I-m-no-expert-but-Consumer-use-of-supportive?redirectedFrom=fulltext) | Bocking H, Russell-Bennett R, Letheren K. I’m no expert, but … ? Consumer use of supportive digital tools in health services. J Serv Theory Pract. 2022;32(2):105-31. doi:10.1108/JSTP-09-2020-0225 | Australia – Fieldwork on a university campus in a metropolitan city. | Consumers aged ≥18 interested in two preventative health services using supportive digital tools (skin-cancer mole checks, STI checks); no pre-existing diagnosis of skin cancer or STI; age range 20–58; 15 female/15 male. | Semi-structured interviews conducted after participants trialled supportive digital tools in preventive health contexts (skin cancer mole checks, STI checks); analysis guided by social support theory and passive-interactive-proactive (PIP) framework | 30 participants (15 female, 15 male; aged 20-58; no pre-existing diagnosis of skin cancer or STI; all capable of basic tech use) | Participants trialled tools on tablets (websites, apps, reminders, symptom checkers). Semi-structured interviews (~1 hour) immediately after use. Conversational tone; coded deductively (theory-led) and inductively (emerging themes). Thematic analysis, hybrid coding approach | Lack of motivation to initiate search: Consumers knew the importance of preventive checks but showed apathy towards initiating action  Desire for control and monitoring: Consumers valued tools that allowed them to track health and initiate discussions with professionals.  Avoiding overuse of the health system: Consumers avoided seeking doctors unless necessary, viewing preventive checks as a resource strain.  Information alone is insufficient: Informational support without actionable guidance was seen as unhelpful.  Hierarchy of social support: Consumers prioritised instrumental support over informational, emotional or esteem support.  Personalisation and proactivity desired: Interactivity without personalisation was viewed as “just information”.  Positive response to tracking and reminders: Apps enabling photo monitoring, reminders and proactive features increased motivation. | Single geographic location limits transferability  Restricted to two health contexts (skin cancer, STI)  Pre-loaded digital tools may not reflect full market  Excluded gamification features  Open internet search produced variability |
| [[82]](https://aisel.aisnet.org/cgi/viewcontent.cgi?article=1102&context=pacis2024) | Mukherjee S, Das S. Willingness to digitalize health records: an Indian perspective. In: PACIS 2024 Proceedings. 2024. Available from: <https://aisel.aisnet.org/pacis2024/track11_healthit/track11_healthit/8> | India – Study conducted in India. | Indians described as non-adopters (pre-adoption phase) exploring intention to use government ABHA app; participants included students from graduation, post-graduation, and doctorate levels; interviews conducted in English and Bengali. | Semi-structured interviews guided by diffusion of innovation (DOI) constructs (relative advantage, compatibility, observability) and Bélanger and Carter’s trust framework (trust in government, trust in internet) | 12 participants (students from varied disciplines, India; 10 interviews in English, 2 in Bengali; mix of postgraduates and doctorates) | Participants explored ABHA app and official video prior to interviews; interviews lasted 15-20 minutes; audio recorded, transcribed, translated where needed; coded manually and in NVivo 14 (auto-coding, word cloud); convenience and purposive sampling | Interoperability benefit: Digital records reduce the burden of carrying paper documents and improve coordination.  Accessibility during emergencies: Digitisation helps avoid delays and risks in emergencies.  Environmental friendliness: Reducing paper use was seen as a major motivator.  Compatibility with lifestyle: Smartphones, internet use and the COVID lockdown experience made digitisation easier to adopt.  Observability through reviews: Positive electronic word of mouth and reviews influenced intention modestly.  Trust in government: Confidence in a government-backed app encouraged adoption.  Limited trust in the internet: Concerns about cyber risks reduced confidence in the platform. | Findings context-specific to India, limited transferability  Focus on pre-adoption phase; real-world usage not studied  Student sample, small size limits generalisability  Absence of comprehensive empirical evidence |
