# Supplementary material for: Advancing foundational models in digital health technology adoption: A systematic literature review of multidisciplinary factors
Source: PLOS Digit Health. 2026 Sep 2;5(9):e0001690. doi: 10.1371/journal.pdig.0001690 (PMC13537594; doi:10.1371/journal.pdig.0001690)
Supplement: Supplementary file 7 [file pdig.0001690.s005.docx]

**S3 Table. Details of quotations and codes**

| **ID** | **Quotation Content** | **Codes** |
| --- | --- | --- |
| 1:2 | Patients struggled with jargon and acronyms, which limited understanding. | Psychological: Confusion about medical acronyms Psychological: Difficulty with medical jargon Psychological: Health literacy level |
| 1:3 | Patients were unsure about the meaning of results and what actions to take. | Psychological: Difficulty in test result interpretation |
| 1:4 | Patients found it difficult to locate or access updated information in the portal. | Technological: Navigation complexity |
| 1:5 | Graphical displays hindered interpretation; some preferred raw numbers. | Psychological: Graph interpretation difficulties Psychological: Preference for data presentation Psychological: Preference for numeric results |
| 1:6 | Patients noticed wrong details such as addresses, appointment times, and misclassified or false test results. | Health: Clinical information errors Technological: Accuracy of patient information within system Technological: Administrative information errors |
| 1:7 | Missing details such as appointments and test results limited usefulness of the portal. | Environmental: Completeness of administrative information Health: Clinical information completeness Technological: Completeness of patient information within system |
| 1:8 | Parents aged 26–35 years showed higher willingness to adopt mHealth compared to younger parents. | Psychological: Influence of age |
| 1:9 | Higher attention to the COVID-19 pandemic increased willingness. | Psychological: Pandemic awareness influence |
| 1:10 | Advice from medical professionals strongly influenced adoption. | Social: HCP recommendations |
| 1:11 | Parents with children suffering from chronic illness were most likely to adopt. | Health: Chronic disease management needs Health: Family health needs |
| 1:12 | Longer daily internet use increased likelihood of adoption. | Psychological: Internet use duration |
| 1:13 | mHealth users were more willing to adopt during COVID-19. | Psychological: Prior DHT experience |
| 1:14 | The strongest predictor of intention was the perceived usefulness of eConsulta in improving efficiency and patient relationships. | Environmental: Improvement of HCP workflow efficiency Psychological: HCP perceived benefits Social: HCP–patient relationship improvement |
| 1:15 | Habitual or broader use of eConsulta increased likelihood of continued adoption. | Social: Types of habitual HCP use of DHT |
| 1:16 | Influence from patients, colleagues, and institutions shaped adoption decisions. | Environmental: Institutional influence Social: Colleagues influence Social: Patient influence |
| 1:17 | Prior familiarity with ICTs and telemedicine boosted adoption. | Psychological: Prior DHT experience Psychological: Prior ICT familiarity |
| 1:18 | Gender differences were significant, especially among physicians | Cultural: Influence of gender |
| 1:19 | Higher levels of institutional implementation encouraged adoption. | Environmental: Institutional implementation maturity |
| 1:20 | Type of professional role influenced adoption differently (physicians vs nurses). | Social: Type of HCP role |
| 1:21 | For nurses, perceived benefits and level of use were strongest predictors, environmental pressure also mattered. | Environmental: Institutional influence Psychological: HCP level of DHT use Psychological: HCP perceived benefits Social: Colleagues influence Social: Patient influence |
| 1:22 | For physicians, perceived benefits, gender, type of use, environmental pressure, and ease of use were significant. | Cultural: Influence of gender Environmental: Institutional influence Psychological: HCP perceived benefits Social: Patient influence Social: Peer influence Social: Types of HCP DHT use Technological: HCP ease of use |
| 1:26 | External encouragement (family, friends, or coworkers) significantly impacted adoption. | Social: Coworkers encouragement Social: Family encouragement Social: Friends encouragement |
| 1:27 | Availability of resources and support encouraged adoption. | Social: Availability of HCP support Social: Technical support Technological: Availability of technical resources |
| 1:30 | Strongest predictor; belief in improved work performance and service quality increased adoption. | Environmental: Improvement of HCP work performance Health: Improvement of healthcare service quality Psychological: HCP performance expectancy |
| 1:31 | Ease of learning and using telemonitoring enhanced intention. | Psychological: HCP effort expectancy Technological: HCP ease of use Technological: Learnability of DHT |
| 1:32 | Availability of resources and infrastructure positively influenced adoption. | Technological: Availability of DHT infrastructure Technological: Availability of technical resources |
| 1:33 | Established use patterns increased intention to use telemonitoring. | Psychological: HCP habitual DHT use |
| 1:37 | Limited hospital e-health system presence reduced access. | Technological: Availability of DHT system in hospitals |
| 1:38 | Higher education and income linked with better experience and attitude toward e-health. | Environmental: Socioeconomic status Psychological: Education level |
| 1:39 | Positive experience with e-health linked to better attitude. | Psychological: Prior DHT experience |
| 1:41 | Strong agreement that telemedicine saves time, money, and increases access. | Environmental: Improvement of specialized care access Environmental: User money savings Environmental: User time savings Psychological: User attitude toward DHT |
| 1:42 | Half of participants found telemedicine video consultations complex. | Psychological: User perceived complexity |
| 1:43 | Higher literacy scores linked to higher intention. | Psychological: Digital literacy level |
| 1:45 | Self-tracking adoption began through gifting, imitation, and informal learning. | Cultural: Access to device through gifting Social: Family modeling effect |
| 1:46 | Negative parental influence shaped problematic relationships with self-tracking. | Social: Parental influence |
| 1:47 | Young people relied on Google/social media for self-tracking guidance. | Psychological: Reliance on social media for health information |
| 1:48 | Devices provided satisfaction, nudges, and motivation to continue body work. | Psychological: Device-driven motivation Psychological: User satisfaction Technological: Device behavioural nudges |
| 1:51 | Sharing self-tracking data fostered competition, validation, and motivation. | Social: Peer validation through data sharing Social: Social competition through data sharing Social: Social motivation through data sharing |
| 1:52 | Strongest predictor of adoption; users prioritized information access over technical performance. | Psychological: User health information-seeking behaviour Psychological: User priority for information access over technical performance |
| 1:53 | Positive effect on adoption intention through peer and community encouragement. | Social: Community support Social: Peer support |
| 1:55 | Telemedicine empowered patients and strengthened doctor-patient relationships. | Psychological: Patient empowerment through self-care Social: HCP–patient relationship enhancement |
| 1:56 | AI and telemedicine reduced costs, duplication, and time burdens. | Environmental: Cost reduction in healthcare delivery Environmental: HCP time savings Technological: Reduction of HCP work duplication |
| 1:57 | Remote care reduced exposure and enabled outbreak detection. | Health: Benefits of infection control Health: Outbreak pattern detection |
| 1:58 | Telemedicine supported continuity of care; AI enabled predictive monitoring. | Health: HCP-enabled chronic illness monitoring Health: HCP-enabled continuity of care Technological: HCP-enabled health alerts prediction |
| 1:59 | Lack of face-to-face contact raised diagnostic risks. | Health: Risk of misdiagnosis in remote consultations Health: Risk of misinformation in remote consultations |
| 1:60 | Biases, regulation, and trust issues limited adoption. | Environmental: Availability of data integrity regulations Environmental: Availability of data safety regulations Environmental: Strictness of data integrity regulations Environmental: Strictness of data safety regulations Psychological: Trust in DHT Technological: Bias in AI algorithms |
| 1:61 | High costs and lack of insurance coverage restricted access. | Environmental: Implementation costs of DHT Environmental: Lack of insurance reimbursement |
| 1:62 | Data storage, workflow disruption, and small-institution gaps hindered adoption. | Environmental: Capacity gaps in small institutions Technological: Data storage capacity limitations Technological: HCP workflow integration |
| 1:63 | Professionals feared loss of autonomy and resisted disruption. | Psychological: HCP resistance to change Social: HCP autonomy concerns |
| 1:64 | Lack of technical expertise limited effective use. | Psychological: HCP technical skill level |
| 1:65 | Awareness campaigns and flexible training boosted adoption. | Social: DHT awareness campaigns Social: HCP DHT training |
| 1:66 | Engaging providers in design fostered ownership and alignment. | Environmental: Ownership in DHT implementation Social: HCP engagement in DHT design |
| 1:67 | Financial and non-financial incentives encouraged adoption. | Environmental: Financial incentives Environmental: Non-financial incentives for HCP stakeholders |
| 1:68 | Convenience, patient-doctor communication, and health monitoring improved satisfaction. | Environmental: User convenience in healthcare access Health: User health monitoring support Social: HCP-patient communication enhancement |
| 1:69 | Patients feared data misuse and loss of empathy. | Cultural: Human touch preference in AI decision-making systems Psychological: Fear of data misuse |
| 1:70 | Ease of use significantly improved intention to adopt m-health apps. | Psychological: User effort expectancy Technological: User ease of use |
| 1:72 | Self-tracking became controlling, obsessive, and linked to negative mental health | Health: Negative mental health impact of self-tracking Health: Overuse of self-tracking Psychological: Obsessive self-tracking |
| 1:73 | Exposure to influencers created unrealistic body ideals. | Social: Social media influence on body image Social: Unrealistic body ideals from influencers |
| 1:74 | Strongest predictor of intention; patients perceived PHRs as useful. | Psychological: User perceived usefulness Psychological: User performance expectancy |
| 1:75 | Ease of use increased intention to adopt. | Psychological: User effort expectancy Technological: User ease of use |
| 2:1 | ITQ had the highest positive effect on users’ satisfaction and their continuous intention to use M-health. | Social: HCP-patient interaction quality |
| 2:2 | SQ significantly influenced both user satisfaction and continuous intention to use M-health. | Technological: System quality |
| 2:3 | IFQ was significant for user satisfaction but had no effect on continuance intention. | Health: Quality of health information in DHT system |
| 2:4 | SQ emphasized availability and accessibility, enabling anytime/anywhere health services. | Technological: Accessibility of DHT Technological: Availability of DHT |
| 2:5 | Majority of patients adopted eHealth mainly out of necessity, not interest. | Environmental: Pandemic-driven necessity |
| 2:6 | More than half indicated they would not continue using eHealth after the pandemic. | Psychological: Post-pandemic intention to use DHT |
| 2:7 | Significant portion of participants were worried about confidentiality of health data. | Health: Concern over health data confidentiality |
| 2:8 | Few participants were highly interested, with most only somewhat or not interested. | Psychological: Interest in DHT |
| 2:9 | Most patients used eHealth for diagnosis, advice, and treatment. | Psychological: Preferred use of DHT |
| 2:10 | Many disagreed that eHealth was secure or easy to share information. | Health: Data security and privacy concerns Technological: Difficulty in sharing information via eHealth systems |
| 2:11 | Age and gender differences influenced future adoption likelihood. | Cultural: Influence of gender Psychological: Influence of age |
| 2:12 | Enjoyment from using chatbots significantly improves satisfaction among older adults. | Psychological: User enjoyment Psychological: User hedonic motivation |
| 2:13 | Adequate infrastructure and support encourage higher satisfaction. | Social: Adequacy of organizational support Technological: Adequacy of technical resources |
| 2:15 | Belief in chatbots’ usefulness significantly increases satisfaction. | Psychological: User perceived usefulness Psychological: User performance expectancy |
| 2:17 | Meeting expectations increases satisfaction with chatbots. | Psychological: User confirmation of chatbot expectations |
| 2:18 | Data protection strongly affects adoption. | Health: Data security and privacy concerns |
| 2:19 | Higher satisfaction leads to stronger continuance intention. | Psychological: User satisfaction |
| 2:23 | Intention to use significantly led to actual DCT tool usage. | Psychological: User intention to use |
| 2:24 | Trust in government significantly increased intention to use DCT tools. | Environmental: Trust in government |
| 2:25 | Social influence (peer behaviour and recommendations) significantly increased actual DCT usage. | Social: Peer influence Social: Peer recommendations |
| 2:26 | COVID-19-induced anxiety significantly shaped technology acceptance, mediated by social influence. | Psychological: Pandemic-induced anxiety |
| 2:27 | Privacy concerns and event-related fear influenced adoption through social influence mediation. | Health: Data security and privacy concerns Health: Fear of pandemic |
| 2:30 | Opinions of relatives, friends, and providers shape adoption. | Social: Family influence Social: HCP influence Social: Peer influence |
| 2:31 | Higher literacy improves adoption, trust, and performance expectancy. | Psychological: Digital literacy level |
| 2:32 | Confidence in platforms increases adoption. | Psychological: Confidence in telemedicine platforms |
| 2:33 | Adoption depended on professionals, older people, and caregivers being familiar with and committed to technology use, and perceiving clear benefits. | Psychological: Commitment to DHT use Psychological: Familiarity with DHT Psychological: User perceived benefits |
| 2:34 | Technology enabled rehabilitation, independence, resource allocation, safety, and social connection. | Environmental: Improvement of healthcare resource allocation efficiency Health: Rehabilitation support and tracking Health: Support for safe independent living Health: User safety assurance Social: Social connection improvement |
| 2:35 | Both professionals and older people faced early challenges but adapted over time. | Technological: Adaptation to DHT over time Technological: User ease of use |
| 2:36 | Acceptance and trust developed when technology was safe and automatic. | Health: User perceived safety in DHT Psychological: Trust in DHT Psychological: User attitude toward DHT Technological: Automatic DHT functions |
| 2:37 | Suspicion, resistance, and perceived lack of benefit reduced willingness to adopt. | Psychological: Suspicion toward DHT Psychological: User perceived benefits Psychological: User resistance to change |
| 2:38 | Professionals, older people, and caregivers were unsure about future continuation due to cost and availability. | Environmental: Uncertainty about DHT availability Environmental: Uncertainty about funding sustainability Environmental: Uncertainty about technology affordability Environmental: Uncertainty of post-implementation sustainability |
| 2:39 | Professionals, older people, and caregivers gained technical, guiding, and coping skills. | Psychological: Development of home care coping skills Social: HCP guiding skills development Social: HCP technical skill development Social: User technical skill development |
| 2:40 | Provided real-time data on wellbeing, care practices, and new possibilities. | Health: Real-time care practice monitoring Health: Real-time health monitoring |
| 2:41 | Created new work processes, improved coping, and allowed procedure modification. | Health: Improvement of home care coping procedures Technological: HCP workflow change Technological: System adaptability to evolving needs |
| 2:42 | Reduced need for physical visits and repetitive checks. | Environmental: Benefit of HCP travel reduction Environmental: HCP workload efficiency improvement |
| 2:43 | Professionals, older people, and caregivers were motivated by improved independence, safety, and resource efficiency. | Environmental: Improvement of healthcare resource allocation efficiency Health: Support for safe independent living Health: User perceived safety in DHT |
| 2:45 | Familiarity was limited, with most only using glucometers. | Psychological: Familiarity with DHT |
| 2:46 | In-person care and information were strongly preferred over digital channels. | Cultural: Preference for in-person consultations |
| 2:47 | Cost, ignorance, and religious beliefs as barriers: These hindered adoption of digital tools like glucometers and apps. | Cultural: Religious beliefs Environmental: Financial expense of DHT Psychological: Awareness of DHT |
| 2:48 | Nearly all participants owned phones, mostly Android with internet access, offering opportunity for mHealth. | Environmental: Internet accessibility Environmental: Mobile phone ownership |
| 2:49 | Widely used, though not fully leveraged for health communication. | Social: Underutilization of social platforms for health |
| 2:50 | Community and interpersonal networks remain trusted channels. | Social: Trust in community or family sources Social: Trust in interpersonal health information |
| 2:51 | Local design and integration into routine practices could enhance adoption. | Cultural: Localized design for context Psychological: Integration of DHT into self-care routines |
| 2:52 | Motivation was driven by nutrition interest and commitment to the project. | Psychological: Personal interest in nutrition Social: Engagement in research |
| 2:53 | Frailty and low perceived relevance limited adoption. | Health: Individual health condition Psychological: User perceived relevance |
| 2:55 | Regular users reported greater awareness of food intake. | Psychological: User habitual DHT use |
| 2:56 | Continuous logging felt restrictive or tiring for certain participants. | Health: Nonmaleficence and potential harms Psychological: User fatigue in DHT use |
| 2:57 | Desire to contribute to research encouraged participation. | Social: Engagement in research |
| 2:59 | Guidance from healthcare professionals and family engagement facilitated sustained adoption. | Social: Availability of HCP support Social: Family support |
| 2:60 | Regular use habits most strongly influenced intentions to adopt Pulsara™. | Psychological: HCP habitual DHT use |
| 2:61 | Ease of use significantly increased clinicians’ intentions to adopt. | Psychological: HCP effort expectancy Technological: HCP ease of use |
| 2:62 | Belief in usefulness for patient care and workflow efficiency drove adoption. | Environmental: Improvement of HCP workflow efficiency Health: Improvement of HCP patient care Psychological: HCP perceived usefulness Psychological: HCP performance expectancy |
| 2:63 | Change-ready organisations were more likely to succeed with adoption. | Environmental: Organisational readiness to change |
| 2:64 | Infrastructure and familiar equipment eased adoption, but connectivity issues created barriers. | Environmental: Internet connectivity Psychological: HCP familiarity with DHT Technological: Availability of DHT infrastructure |
| 2:65 | Users faced access problems that reduced ease of adoption. | Technological: Authentication and access difficulties |
| 2:66 | Poor quality in moving vehicles affected adoption confidence. | Technological: Data quality |
| 2:67 | Lack of digital literacy and insufficient training reduced adoption. | Psychological: Digital literacy level Social: HCP DHT training |
| 2:68 | Users reported timelier, more standardised, and accessible information. | Health: Timeliness of health information for HCP Social: HCP access to patient health information Technological: Standardization of health information for HCP |
| 2:69 | Retaining usual communication systems during trial created inefficiencies | Technological: HCP work duplication |
| 2:70 | Caregivers found Zoom/Skype easy due to COVID-19 use for family, church, and medical visits. | Psychological: Prior DHT experience |
| 2:71 | Some struggled with setup, functionality, and camera positioning. | Technological: Usability and setup difficulties |
| 2:72 | Most participants reported trackers were user-friendly and motivating. | Technological: User ease of use |
| 2:73 | Wearing devices constantly and frequent notifications felt annoying. | Psychological: Device wearability burden Psychological: Notification fatigue |
| 2:74 | Some saw them as useful reminders, others as annoying or unnecessary. | Psychological: Message overload Technological: Supportive reminders |
| 2:75 | Coaching added accountability, motivation, and professional validation. | Health: Expert guidance and reassurance Psychological: Availability of professional coaching Psychological: Coach-driven motivation Psychological: Coach-mediated accountability |
| 2:76 | Fitting with daily PA lifestyle, internal motivation, and available technical support encouraged adoption. | Psychological: Internal motivation Psychological: User lifestyle fit Social: Technical support |
| 2:77 | Some felt too old for new tech, while others with prior computer experience were confident. | Psychological: Influence of age Psychological: Prior computer experience |
| 2:78 | When HF patients had good tech experiences, caregivers were more willing to try. | Social: Patient influence |
| 2:79 | Poor Internet connections made video sessions frustrating and unreliable. | Environmental: Internet connectivity |
| 2:80 | Simplicity and ease-of-use positively affect satisfaction. | Psychological: User effort expectancy Technological: User ease of use |
| 2:81 | Perceived usefulness had a significant positive effect on intention to use DCT tools. | Psychological: User perceived usefulness |
| 2:82 | Perceived ease-of-use had a significant positive impact on adoption intention. | Technological: User ease of use |
| 2:83 | Users adopt telemedicine if they perceive it as useful and beneficial. | Psychological: User perceived benefits Psychological: User perceived usefulness Psychological: User performance expectancy |
| 2:84 | Ease of use drives intention to use telemedicine. | Psychological: User effort expectancy Technological: User ease of use |
| 2:85 | Even those without prior technology experience managed to use the app. | Technological: User ease of use |
| 2:86 | Most respondents had tertiary education, creating opportunities for digital health education and co-design. | Psychological: Education level |
| 3:1 | Ease of use, wearability, portability, convenience, and seamless syncing motivate engagement, while usability failures lead to disengagement. | Environmental: User convenience in health tracking Technological: Device portability for active lifestyle Technological: Seamless device-app data synchronization Technological: User ease of use Technological: Wearability comfort |
| 3:2 | Health data collection, monitoring, dashboard tracking, gamification, and statistics encourage sustained engagement and re-engagement. | Technological: Dashboard-based data visualization Technological: Gamification Technological: User health data monitoring function |
| 3:3 | Attractive interface, ergonomic design, and unobtrusiveness matter when users consider returning to or upgrading devices. | Technological: Ergonomic comfort of wearables Technological: Interface attractiveness Technological: Unobtrusive device design |
| 3:4 | Intrinsic motivators (fitness, wellness, stress reduction) and extrinsic motivators (team, competition, obligations) encourage use. | Psychological: External motivation Psychological: Intrinsic motivation Psychological: Personal fitness improvement goals Psychological: Social competition motivation Psychological: Stress-reduction motivation Psychological: Wellness motivation for self-care Social: Motivation for team participation Social: Social obligations pressure |
| 3:5 | mHealth devices help users recognize health behaviours, evaluate performance, and increase accountability. | Health: User health performance evaluation Psychological: User accountability in health behaviours Psychological: User self-recognition of health behaviours |
| 3:7 | Customer support, peer support, team interaction, and community enhance motivation and uptake. | Social: Community support Social: Peer support Social: Support services for users Social: Team profile enhancement |
| 3:8 | Family, work, illness, short battery life, and device failures disrupt engagement, leading to limited use or self-management. | Environmental: User work time constraints Health: Individual health condition Social: Family responsibilities Technological: Battery life Technological: Device malfunction |
| 3:9 | Physicians are more willing to adopt Digital Therapeutics (DTx) when they perceive them as beneficial for patient care and work effectiveness. | Environmental: Improvement of HCP work effectiveness Health: Improvement of HCP patient care Psychological: HCP perceived benefits Psychological: HCP perceived usefulness |
| 3:10 | Simplicity and intuitiveness of DTx increase perceptions of their effectiveness. | Technological: HCP ease of use Technological: Preference for intuitive DHT Technological: Preference for simple DHT |
| 3:11 | Organizational openness to innovation makes physicians view DTx as less complex. | Cultural: Organizational innovation culture Psychological: Openness to new technologies |
| 3:12 | Structured regulatory frameworks from associations enhance perceived benefits of DTx. | Environmental: Support from structured regulatory frameworks |
| 3:13 | Physicians’ views are influenced by peers and respected colleagues in scientific associations. | Social: Peer influence |
| 3:14 | Age, professional role, sense of belonging to scientific societies, and risk propensity significantly shape acceptance. | Environmental: Scientific society membership Psychological: Influence of age Psychological: Risk propensity Social: HCP professional role |
| 3:15 | Older adults are more likely to adopt wearable devices when they believe these devices improve daily life and health management. | Psychological: User perceived usefulness Psychological: User performance expectancy |
| 3:16 | Ease of learning and operating devices increases willingness to adopt. | Psychological: User effort expectancy Technological: Learnability of wearable devices Technological: User ease of operation |
| 3:17 | Family, peers, and social networks encourage device use. | Social: Peer support Social: Social network support |
| 3:18 | Availability of resources, knowledge, and technical support enhances acceptance. | Psychological: DHT knowledge gaps Social: Technical support Technological: Availability of technical resources |
| 3:19 | Enjoyment and pleasure in using wearables boost willingness. | Psychological: User enjoyment Psychological: User hedonic motivation |
| 3:20 | Cost-effectiveness is a critical determinant for older adults. | Psychological: User cost-benefit perception |
| 3:21 | Higher DHL improves understanding and willingness to use devices. | Psychological: Digital health literacy level |
| 3:23 | Willingness to try new tech enhances both usefulness and ease of use perceptions. | Psychological: Openness to new technologies |
| 3:24 | Anxiety and unease reduce both usefulness and ease of use perceptions. | Psychological: User burden of device use Psychological: User technology-related anxiety |
| 3:25 | Privacy and safety concerns reduce ease of use perceptions. | Health: Data security and privacy concerns |
| 3:26 | Older adults with probable AD require reminders and assistance from family members to use mobile app-based cognitive assessments. | Social: Family assistance support Social: Family reminder support |
| 3:27 | Memory decline, emotional distress, and physical health issues hinder engagement with digital cognitive testing. | Health: Memory decline Health: Physical health issues Psychological: User emotional distress during use |
| 3:28 | Small fonts, unclear images, login issues, crashes, and non-intuitive interface make app usage difficult for older adults with AD. | Technological: Authentication and access difficulties Technological: Device malfunction Technological: Font size Technological: Image clarity and quality Technological: Intuitive interface design |
| 3:29 | Some participants joined the study to help future dementia research, showing research-related motivations beyond personal benefit. | Psychological: Altruistic motivation Social: Engagement in research |
| 3:30 | Carers expected the app to help with memory problems and communication in daily life. | Psychological: Expectation of daily life communication aid Psychological: Expectation of daily life memory aid |
| 3:31 | Some saw potential for long-term communication support and independence as dementia progresses. | Health: Support for safe independent living Psychological: Maintenance of independence in daily living Psychological: User future-oriented motivation Social: Daily life communication support |
| 3:32 | Participants hoped the app could bring order to daily tasks and caregiving routines. | Social: Care coordination support Technological: User aid in daily task management |
| 3:33 | Some participants expressed fear and anxiety about learning new technology, leading to dropout. | Psychological: User technology-related anxiety |
| 3:34 | Some users preferred calendars, post-its, or existing devices over the app, seeing duplication of effort. | Cultural: User preference for traditional tools Technological: Duplication of effort in DHT use |
| 3:35 | Carers felt time and effort needed to use the app conflicted with daily caregiving responsibilities. | Environmental: User time conflicts Psychological: Input effort conflicts Social: Caregiving workload burden |
| 3:36 | Declining memory and health reduced ability to engage with the app over time. | Health: Individual health condition Health: Memory decline |
| 3:37 | Lack of cross-linking features, speed, and diary limitations reduced usefulness and hindered adoption. | Technological: Cross-feature integration Technological: DHT functionality gaps |
| 3:38 | For a few, the app helped maintain identity, daily activities, and future-proofing information. | Psychological: Maintenance of independence in daily living Social: User personhood support Technological: Health information repository |
| 3:39 | Family, peers, and socially important people significantly shape older adults’ intention to use public eHealth services. | Social: Family influence Social: Peer influence Social: Support from close acquaintances |
| 3:40 | Government support and perceived health benefits strongly predict intention to adopt public eHealth services. | Environmental: Government support Psychological: User perceived health benefits |
| 3:41 | Trustworthiness, user-friendly interfaces, and policy initiatives make older adults more secure and confident in eHealth use. | Environmental: Availability of privacy policies Environmental: Trust in government Technological: User-friendly system interface |
| 3:42 | Adoption grows when eHealth is perceived as beneficial, popular, and socially/culturally endorsed. | Cultural: Cultural acceptance Psychological: User perceived benefits Social: Social endorsement within communities |
| 3:43 | Volunteer programs and encouragement from close acquaintances increase adoption confidence. | Social: Support from close acquaintances Social: Volunteer support program for DHT service |
| 3:44 | PU significantly impacts intention to adopt digital health. | Psychological: HCP perceived usefulness |
| 3:45 | Willingness to try new technologies increases adoption. | Psychological: Openness to new technologies |
| 3:46 | Healthy lifestyle beliefs increase perception of digital health tools’ value. | Psychological: Healthy lifestyle orientation Psychological: User health beliefs |
| 3:47 | Ease of use strongly enhances perceived usefulness and indirectly adoption. | Technological: User ease of use |
| 3:48 | Peer and social group opinions strongly predict adoption. | Social: Peer influence Social: Reference group influence |
| 3:49 | Strong frameworks and adaptability increase ease and adoption. | Environmental: Inclusive DHT framework Technological: System adaptability to evolving needs |
| 3:50 | Online resources negatively impact ease of use. | Psychological: Influence of online health information |
| 3:51 | Patients as organizations produce and manage health data, increasing engagement and autonomy. | Psychological: Patient empowerment through health data management Technological: Patient-generated health data |
| 3:52 | Ease of using smartphone fitness apps significantly increases intention to adopt. | Psychological: User effort expectancy Technological: User ease of use |
| 3:53 | Opinions of family, friends, and colleagues strongly affect adoption decisions. | Social: Family influence Social: Peer influence |
| 3:54 | Perceived financial benefits versus costs positively influence adoption. | Psychological: User cost-benefit perception |
| 3:55 | Past use habits strongly influence intention to continue adoption. | Psychological: Past fitness app use habits |
| 3:56 | Willingness to experiment with new technologies most strongly predicts adoption. | Psychological: Openness to new technologies |
| 3:57 | Confidence enhances effort expectancy, which influences adoption. | Psychological: Digital confidence |
| 3:58 | Lack of clear framework, telemedicine rules, and data usage guidelines delay digital transformation. | Environmental: Availability of data governance rules Technological: Availability of DHT regulations |
| 3:59 | Poor internet connectivity, outdated systems, and rural inequalities reduce effectiveness. | Environmental: Internet connectivity Environmental: Outdated healthcare systems Environmental: Rural inequalities |
| 3:60 | Budget limitations slow investment in robust digital health systems. | Environmental: National funding allocation |
| 3:61 | Strict regulations slow adoption despite importance for trust and innovation. | Environmental: Strictness of data integrity regulations Environmental: Strictness of data safety regulations |
| 3:62 | Low digital skills and resistance to change block effective adoption. | Cultural: Resistance to change in healthcare Psychological: Digital health literacy level |
| 3:63 | Lack of coordination and standardized practices across healthcare providers slows digitalization. | Environmental: Fragmented healthcare practices Environmental: Institutional interoperability gap |
| 3:64 | Training future professionals and policymakers is essential. | Environmental: Policymaker DHT training Social: HCP DHT training |
| 3:65 | Citizens need awareness initiatives before adoption. | Social: DHT awareness campaigns |
| 3:66 | Stakeholders believe digitalization improves access, prevention, transparency, and efficiency. | Environmental: Healthcare access improvement Environmental: Improvement in efficiency of healthcare delivery Environmental: Improvement in transparency of healthcare decisions Environmental: Transparency in financial resource allocation Health: Disease prevention benefits Psychological: User perceived benefits |
| 3:67 | Time-saving, efficiency, and usefulness of telemedicine apps increase perceived value and acceptance. | Environmental: Improvement of healthcare service efficiency Environmental: User time savings Psychological: User perceived usefulness Psychological: User relative advantage |
| 3:68 | Alignment with users’ healthcare practices, beliefs, and values enhances perceived value and acceptance. | Health: Alignment with user health practices Psychological: User value compatibility |
| 3:69 | Difficulty of use reduces perceived value and public acceptance. | Psychological: User perceived complexity |
| 3:70 | Opportunities to try out apps reduce uncertainty and enhance acceptance. | Environmental: Opportunity for usage testing |
| 3:71 | Visibility of positive outcomes builds trust and motivates use. | Psychological: Outcome visibility |
| 3:73 | Goals for exercise, nutrition, sleep, weight, and heart rate motivate sustained use, self-management, and re-engagement. | Psychological: Exercise goal setting Psychological: Health self-management goal setting Psychological: Heart rate goal setting Psychological: Nutrition goal setting Psychological: Sleep goal setting Psychological: Weight management goal setting |
| 3:74 | Positive attitudes boost perceived usefulness and ease of use. | Psychological: User attitude toward DHT |
| 4:1 | Older adults (≥65) were less likely to use online medical records. | Psychological: Influence of age |
| 4:2 | Black and Hispanic patients had significantly higher nonuse rates. | Cultural: Ethnic disparity Cultural: Racial disparity |
| 4:3 | Cancer patients were more likely to use online medical records. | Health: Disease management needs |
| 4:4 | Most nonusers preferred speaking directly to their healthcare providers. | Cultural: Preference for in-person consultations |
| 4:5 | Many respondents felt they did not need online records. | Psychological: User perceived lack of need |
| 4:6 | Minorities and older adults expressed greater concern about privacy. | Health: Data security and privacy concerns |
| 4:7 | Older adults felt uncomfortable with technology use. | Psychological: Digital comfort |
| 4:8 | Patients struggled with logging in and managing multiple records. | Technological: Authentication and access difficulties |
| 4:9 | Female patients were more likely to use online records; older, Black, and Hispanic patients were less likely. | Cultural: Ethnic disparity Cultural: Influence of gender Cultural: Racial disparity Psychological: Influence of age |
| 4:10 | Clinicians cited difficulties with usability, interoperability, and “click burden.” | Technological: Cross-system interoperability Technological: DHT usability issues Technological: DHT workflow efficiency Technological: Within-system interoperability |
| 4:11 | Long-practicing clinicians resisted shifting from traditional decision-making to CDS tools. | Psychological: HCP resistance to change |
| 4:12 | High workload and short patient visits left little time for CDS tool use. | Environmental: HCP time constraints Environmental: HCP workload constraints |
| 4:13 | Lack of seamless EMR integration discouraged use. | Technological: Tool–system integration |
| 4:14 | Adoption depended on hospital/committee approval, limiting autonomy. | Environmental: Organizational approval dependency |
| 4:15 | Organizational champions encouraged adoption and peer uptake. | Environmental: Leadership endorsement Environmental: Presence of organizational champions |
| 4:16 | Hands-on support, guides, and training sessions reduced adoption barriers. | Social: HCP DHT training Social: Technical support |
| 4:17 | Clinicians demanded strong clinical evidence of effectiveness before adoption. | Health: HCP validation requirement (evidence-based) |
| 4:18 | Tools needed simple, efficient, and minimal-click designs to be practical. | Technological: HCP efficient information retrieval Technological: Minimal interaction requirements in DHT use Technological: Preference for simple DHT |
| 4:19 | Clinicians feared bias, malpractice liability, and overreliance on CDS tools. | Environmental: HCP malpractice liability concern Health: Risk of HCP overreliance on DHT Social: HCP concern about DHT algorithmic bias Social: HCP fear of racial and ethnic bias in DHT use |
| 4:20 | Pilot programs and staged rollouts were seen as more successful. | Environmental: Preference for phased rollout strategy Social: HCP preference for pilot testing |
| 4:21 | Nurses’ belief that digital health care enhances job performance strongly predicts acceptance. | Health: HCP job performance enhancement Psychological: HCP performance expectancy |
| 4:22 | Organizational and technical support significantly increase intention to adopt. | Environmental: Organizational support Social: Technical support |
| 4:23 | Nurses’ willingness to try new technologies has a direct positive effect on intention. | Psychological: Openness to new technologies |
| 4:24 | Opportunities to try digital health care tools enhance performance expectancy and facilitating conditions. | Environmental: Opportunity for usage testing |
| 4:25 | Perceived difficulty negatively affects performance expectancy, effort expectancy, and facilitating conditions, reducing acceptance. | Psychological: Perceived complexity by HCP |
| 4:26 | Nurses viewed WeChat as useful for frequent micro-interactions and continuous support beyond clinic hours. | Health: HCP-enabled continuity of care Psychological: HCP perceived usefulness Social: HCP-enabled patient communication |
| 4:27 | Nurses felt constant availability pressures, creating role ambiguity and emotional burden. | Psychological: HCP emotional workload strain Social: Erosion of HCP role boundaries |
| 4:28 | Nurses feared that digital text-based exchanges diluted empathy compared to face-to-face care. | Social: HCP concern about reduced empathy in digital communication |
| 4:29 | Lack of policies, guidelines, and formal training forced nurses to improvise digital communication practices. | Environmental: Digital communication policy for HCP availability Social: HCP training for digital communication |
| 4:30 | Nurses stressed verifying information and managing patient privacy on WeChat. | Social: HCP concern over patient data security and privacy Social: HCP content credibility in digital communication |
| 4:31 | Extended family members often participated in chats, sometimes conflicting with medical advice. | Social: Conflict of family involvement with medical advice |
| 4:32 | Tech-savvy mothers embraced WeChat, while less skilled women struggled with basic features. | Psychological: Digital literacy level Technological: Navigation complexity |
| 4:33 | Infographics, color-coding, and voice messages improved comprehension. | Technological: Multimedia aids for comprehension |
| 4:34 | Pregnant women valued WeChat for saving travel time and wages. | Environmental: User money savings Environmental: User time savings |
| 4:35 | Older or less educated women felt hesitant, embarrassed, or marginalized. | Psychological: Education level Psychological: Influence of age Social: User perceived marginalization |
| 4:36 | Both nurses and patients agreed hospital-approved content enhanced trust. | Environmental: Trust in hospital-approved content |
| 4:37 | Patients expected fast replies, while nurses struggled with workload. | Environmental: HCP workload response gap Psychological: User expectation for fast replies |
| 4:38 | Both groups recommended digital office hours, content libraries, and training to improve sustainability. | Social: HCP DHT training Social: Structured digital office hours Technological: Provision of health content library |
| 4:40 | Many participants hesitated to sign up because they were unsure if the DMHI would be useful or easy to use. | Psychological: Uncertainty about ease of use Psychological: Uncertainty of usefulness |
| 4:41 | Early engagement was hindered when participants forgot about the program or could not find time to use it. | Environmental: User time constraints Psychological: User forgetfulness in DHT use |
| 4:42 | Users expressed that human contact was still essential alongside digital solutions. | Social: User desire for human support in DHT use |
| 4:43 | Stigma prevented individuals from engaging with digital interventions. | Cultural: Stigma barrier to DHT use |
| 4:44 | Limited understanding of DMHIs and therapy reduced adoption. | Psychological: DHT knowledge gaps |
| 4:45 | Participants wanted interventions tailored to their personal context. | Psychological: User desire for personalized care |
| 4:46 | Ongoing, changing mental health needs shaped adoption decisions. | Health: Evolving mental health needs |
| 4:47 | Older adults valued smart bracelets with safe use, real-time feedback, correct data, comfortable wear, and clear screen. | Health: User health data accuracy Health: User perceived safety in DHT Technological: Real-time information feedback Technological: Screen visibility and readability Technological: Wearability comfort |
| 4:48 | Smart bracelets helped increase health awareness, satisfy curiosity, support relaxation, and enhance technology understanding. | Psychological: Curiosity satisfaction Psychological: Enhanced understanding of DHT Psychological: Increasing health awareness Psychological: Relaxation support |
| 4:49 | Users aimed for healthier bodies, improved quality of life, and a sense of social belonging. | Psychological: Goal of quality-of-life enhancement Psychological: Health improvement goal Social: Value of social belonging |
| 4:50 | Comfort and readability were critical due to physical and sensory changes in older age. | Technological: Screen visibility and readability Technological: Wearability comfort |
| 4:51 | Real-time feedback and activity monitoring encouraged healthy behaviour. | Technological: Real-time information feedback Technological: User activity monitoring |
| 4:52 | Devices supported social connections, reduced loneliness, and enhanced well-being. | Psychological: Enhancement of emotional well-being Social: Social connection support |
| 4:53 | Few devices were tailored to older users’ needs; fast tech updates made adoption harder. | Environmental: Rapid technology turnover Technological: Age-friendly design |
| 4:54 | Older adults worried about sensitive health and social data being used/shared. | Health: Data security and privacy concerns Psychological: Fear of data misuse |
| 4:55 | Lack of awareness, data risks, weak leadership, and unclear policies form the foundation of adoption challenges. | Environmental: Government policy gaps Environmental: Lack of leadership support Health: Data security and privacy concerns Psychological: Awareness of AI value |
| 4:56 | Competitive pressures, patient trust, and weak partnerships directly influenced adoption choices. | Environmental: Competitive pressure Psychological: Trust in AI service Social: HCP-vendor relationships |
| 4:57 | Complexity of AI systems, skepticism of AI capabilities, and medically derived risks hindered acceptance. | Health: Medically derived risks Psychological: Skepticism about AI capabilities Technological: Complexity of AI systems |
| 4:58 | Lack of talent, financial costs, and inability to share information were key internal barriers. | Environmental: Financial cost barrier Environmental: Skilled workforce shortage Technological: Limitations in information sharing |
| 4:59 | Geographical disparities and unclear hospital ownership complicated AI implementation. | Environmental: Geographical disparities Environmental: Unclear hospital ownership |
| 4:60 | Information sharing limitations were identified as the strongest bottleneck in adoption. | Technological: Limitations in information sharing |
| 4:61 | Meeting elderly patients’ complex needs and gaining trust were crucial for successful adoption. | Psychological: Requirement for trust in AI service Technological: Tailored to user complex needs |
| 4:62 | Staff emphasized that smartwatch data must be embedded into daily routines, not add extra burden. | Technological: HCP workflow integration requirement |
| 4:64 | Residents accepted smartwatches as part of modern life, comparing them to regular watches. | Cultural: Social normalization of DHT |
| 4:66 | Residents and staff valued real-time tracking of heart rate, oxygen, sleep, and activity. | Health: Value of real-time health metric monitoring |
| 4:67 | Families wanted access to smartwatch data to monitor loved ones and reduce uncertainty. | Social: Family access to user data |
| 4:68 | Staff highlighted smartwatch alerts for illness detection and rehab tracking as highly useful. | Health: HCP-enabled rehabilitation tracking Technological: HCP-enabled health alerts prediction |
| 4:71 | Users emphasized the need for clear, reliable, and efficient feedback from devices. | Social: HCP clear feedback need Social: HCP efficiency feedback need Social: HCP reliable feedback need Technological: Feedback quality from DHT use |
| 4:72 | Devices were seen to enhance CPR quality and patient survival. | Health: Clinical benefits Psychological: Outcome expectancy |
| 4:74 | Simplicity, lightweight design, and user-friendly features encouraged adoption. | Technological: Lightweight device design Technological: Simple device design Technological: User-friendly features |
| 4:75 | Overly complex feedback discouraged users, especially laypersons. | Technological: Feedback complexity |
| 4:77 | Adoption often followed institutional directives, not personal choice. | Environmental: Institutional directives |
| 4:78 | Regular training and technical support were essential for acceptance. | Social: HCP DHT training Social: Technical support |
| 4:79 | High cost discouraged adoption, particularly in resource-limited settings. | Environmental: Financial expense of DHT |
| 4:80 | Interoperability across settings and devices supported adoption. | Technological: Interoperability across devices Technological: Interoperability across settings |
| 4:81 | Participants questioned accuracy of digital BP devices compared to manual ones. | Psychological: Skepticism about DHT accuracy |
| 4:82 | Lack of awareness, trust, and comfort hindered adoption of teleconsultation. | Psychological: Awareness of DHT Psychological: Digital comfort Psychological: Trust in DHT |
| 4:83 | Informal providers were trusted more than hospital staff for accessibility and attentiveness. | Social: Preference for accessible local providers Social: Trust in informal care |
| 4:84 | BP devices were often shared within communities, reinforcing social ties but with boundaries. | Social: Community-based device intermediation |
| 4:85 | Transport, lost wages, and companion costs added burdens even when services were free. | Environmental: Hidden costs of digital care |
| 4:87 | Relatives supported with remedies, physical help, and technology use. | Social: Family support |
| 4:88 | Residents faced difficulties with step tracking, reading displays, and syncing devices. | Technological: Device syncing difficulties Technological: Difficulty in display readability Technological: Difficulty in step tracking Technological: User technical usability issues |
| 4:89 | Some residents worried about who could access their health data. | Environmental: Concern over authority access to data |
| 4:90 | Residents were excited and open, despite age-related digital divide concerns. | Psychological: Concern about age-related digital divide Psychological: Openness to new technologies Psychological: User attitude toward DHT |
| 4:91 | Mobility limitations, Bluetooth connectivity issues, and small displays threatened adoption. | Health: Mobility limitations Technological: Device–system connectivity Technological: Display size limitations |
| 4:92 | Users wanted devices usable in different environments, weather conditions, and for all patient groups. | Environmental: Weather adaptability Social: User inclusivity Technological: Environmental usability |
| 4:93 | Social reinforcement from respected peers encouraged adoption. | Social: Peer support |
| 4:94 | Household responsibilities and dependence on men limited women’s access to care. | Cultural: Influence of gender Social: Household responsibility |
| 5:2 | Minimal interaction and simple positioning made the device easy to use. | Technological: Minimal interaction in DHT use Technological: Navigation complexity Technological: User ease of use |
| 5:3 | Comfort varied by personal preference, but strap irritation and size were noted. | Health: Risk of strap irritation Technological: Device size Technological: Wearability comfort |
| 5:4 | Patients trusted it was collecting data but wanted some feedback. | Psychological: User desire for feedback provision Psychological: User perceived lack of direct usefulness |
| 5:5 | Wearing for a week was acceptable, but longer caused reluctance. | Health: User burden of continuous wear Health: Wear-time tolerance limit |
| 5:6 | Users trusted clinicians and universities but distrusted commercial companies. | Environmental: Trust in institutions Psychological: Distrust in commercial providers Social: Trust in healthcare professionals |
| 5:7 | Technology seen as a bridge to improve patient–clinician communication. | Technological: HCP-patient communication tool |
| 5:11 | Data security and fear of surveillance were significant issues, especially with ML monitoring. | Health: Data security and privacy concerns Psychological: Fear of surveillance |
| 5:12 | Employees worried dSMIs may not reduce stress or might worsen it. | Health: Nonmaleficence and potential harms |
| 5:13 | Participants didn’t fully understand how tech could help their condition. | Psychological: Uncertainty about DHT role in health management |
| 5:14 | Illness progression forced lifestyle changes influencing openness to tech. | Health: Illness-forced lifestyle change |
| 5:15 | Employees stressed the importance of voluntary use and control over interaction with dSMIs. | Cultural: Emphasis on voluntary use Psychological: User autonomy in DHT use Psychological: User control over engagement |
| 5:17 | Fears of stigmatization at work due to visible use of dSMIs. | Social: Workplace stigma risk from visibility |
| 5:18 | Some employees preferred nondigital methods, resisting more digital engagement. | Cultural: Preference for non-digital stress coping strategies |
| 5:19 | Desire for seamless integration with existing devices and apps. | Technological: Seamless integration with existing devices and apps |
| 5:20 | Preference for simple, intuitive design and adaptability. | Technological: Adaptability design Technological: Intuitive interface design Technological: Preference for simple DHT |
| 5:22 | COVID-19 forced older adults to adopt eHealth due to shutdowns and distancing. | Environmental: Pandemic-driven adoption |
| 5:23 | Fear of infection motivated eHealth use | Health: Fear of infection |
| 5:24 | Convenience and better communication made eHealth attractive. | Environmental: User convenience in healthcare access Psychological: User perceived usefulness Social: HCP-patient communication enhancement |
| 5:27 | Family and broader social movements encouraged adoption. | Social: Family influence Social: Social network support |
| 5:28 | Fear of data misuse, especially Medicare and SSN. | Psychological: Fear of data misuse |
| 5:29 | Fear of unnecessary messages discouraged adoption. | Psychological: Fear of unnecessary messages |
| 5:30 | Limited skills caused anxiety in use | Psychological: Digital literacy level Psychological: User technology-related anxiety |
| 5:31 | Typing and device use were effortful. | Health: Physical health issues Psychological: Effortful typing and device use |
| 5:32 | Absence of eHealth use among peers reduced motivation. | Social: Peer norms |
| 5:33 | Costly equipment and poor internet restricted access. | Environmental: Cost of equipment or device Environmental: Internet connectivity Technological: Availability of DHT infrastructure |
| 5:34 | Students who found MHAs helpful were more likely to accept them. | Psychological: User perceived usefulness |
| 5:35 | Ease of use raised perceived usefulness, which then increased acceptance. | Technological: User ease of use |
| 5:36 | Higher stigma was linked to lower utilization of traditional services. | Cultural: Stigma barrier to DHT use |
| 5:37 | MHAs were valued for confidentiality compared to face-to-face care. | Technological: Confidential self-management affordances |
| 5:39 | Optimistic older adults were more confident in using DHT and saw it as beneficial. | Psychological: Personal optimism |
| 5:40 | Older adults with higher innovativeness found DHT easier to use. | Psychological: Personal innovativeness |
| 5:41 | Feeling overwhelmed by technology discouraged adoption. | Psychological: Digital comfort |
| 5:42 | Older adults who found DHT easy to use also found it more useful. | Technological: User ease of use |
| 5:43 | Attitudes shaped by usefulness and ease of use predicted intention. | Psychological: User attitude toward DHT |
| 5:44 | High literacy enhanced optimism and innovativeness while weakening discomfort effects. | Psychological: Digital health literacy level Psychological: Digital literacy level |
| 5:45 | Married and higher-income older adults had higher eHealth literacy. | Environmental: Socioeconomic status Social: Marital status |
| 5:46 | Education and guidance could help reduce fear and enhance skills. | Social: User DHT education Social: User DHT guidance |
| 5:47 | Older adults with more limitations had lower access to digital tools. | Health: User barriers due to functional limitations |
| 5:48 | Those with more limitations were more likely to adopt video calls with providers. | Health: Functional limitations driven adoption |
| 5:49 | Prior consistent internet use supported telehealth adoption. | Psychological: Prior internet use |
| 5:50 | Social support increased likelihood of telehealth use among those with limitations. | Social: Family support Social: Peer support |
| 5:51 | Without supportive design and access, older adults with limitations risk being excluded. | Technological: Inclusive design Technological: Supportive design |
| 5:52 | CARES app valued for improving ART service delivery and data management. | Psychological: HCP perceived usefulness Psychological: Perceived value in service delivery Psychological: Perceived value of data management |
| 5:53 | Interface design similar to EMRS improved usability. | Psychological: HCP familiarity with DHT Technological: Encrypted synchronization across DHTs |
| 5:55 | Continued use partly driven by expectations from supervisors and developers. | Social: Expectation from supervisors Technological: Expectations of developers |
| 5:56 | Users stayed hopeful about future improvements and relevance for their work. | Psychological: User hopefulness for future improvements Psychological: User hopefulness for work relevance |
| 5:59 | Expansion to other health areas and interoperability with EMRS critical for success. | Environmental: Requirement for service expansion Technological: Interoperability between service |
| 5:60 | Improved access, cost efficiency, timely consultations, monitoring, and prescription management encouraged use. | Environmental: Cost efficiency for facilities and parties Environmental: Rural healthcare access Health: Timely consultations and monitoring Technological: Digital prescription management |
| 5:61 | High costs limited adoption across facilities. | Environmental: Financial expense of DHT |
| 5:62 | Registration requirements and data transfer standards created adoption challenges. | Environmental: Data transfer standards Environmental: Regulatory registration requirements |
| 5:63 | Lack of infrastructure and inputs limited adoption in rural areas. | Technological: Availability of DHT infrastructure |
| 5:64 | Supply-side adoption depended on innovation and access to financial resources. | Environmental: Dependence on vendor innovation Environmental: Financial resources accessibility |
| 5:65 | Environmental factors such as political and institutional backing strongly shaped adoption. | Environmental: Political and institutional backing |
| 5:66 | Trust required robust data protection in telemedicine use. | Health: Data privacy and security needs |
| 5:67 | Faith-based and private facilities showed stronger adoption than public facilities due to resources and autonomy. | Environmental: Facility resource autonomy |
| 5:69 | Better access increases adoption of health technologies. | Technological: Accessibility of DHT |
| 5:70 | Access helps mitigate inequities in care. | Environmental: Reduction of healthcare disparities |
| 5:71 | Use of apps, telehealth, and EHRs improves equity. | Environmental: Reduction of healthcare disparities |
| 5:72 | Higher socioeconomic status boosts access to devices and internet. | Environmental: Internet accessibility Environmental: Socioeconomic status Technological: Accessibility of DHT |
| 5:73 | Structural racism, discrimination, and digital redlining worsen inequities. | Cultural: Systemic racism Environmental: Digital redlining |
| 5:74 | Cost, literacy, and infrastructure gaps reduce adoption. | Environmental: Affordability for DHT Psychological: General literacy constraints Technological: Availability of DHT infrastructure |
| 5:78 | Those with stroke history needed greater technical help compared to non-stroke participants. | Social: Technical assistance needs |
| 5:79 | Comfort level was especially important for stroke survivors. | Technological: Wearability comfort |
| 5:80 | Stroke survivors focused on movement controls, while non-stroke participants focused on aesthetics. | Psychological: Types of priorities in DHT use Psychological: Usefulness of DHT Technological: DHT aesthetics |
| 5:81 | Prior rehab experience fostered skepticism toward novel tools. | Psychological: Skepticism from prior rehabilitation experience |
| 5:82 | Games that were too difficult risked demotivating participants. | Technological: Gameplay difficulty balance need |
| 5:83 | Addressing data absenteeism and involving older adults in co-design was crucial. | Cultural: Representation to avoid biased DHT development Social: User engagement in DHT design |
| 5:84 | Calls for certified oversight, transparency, and employer responsibility. | Environmental: Oversight by certified companies Environmental: Transparency requirements in evaluation Social: Employer responsibility for employee well-being |
| 5:85 | eHealth enabled more direct and documented communication. | Social: HCP-patient communication enhancement |
| 5:86 | Repetition and prior ICT experience improved confidence. | Psychological: Prior ICT experience |
| 5:87 | App slowness, resets, and data errors undermined efficiency. | Technological: App slowness Technological: Data errors Technological: System reset disruption |
| 5:88 | Infrastructure, training, and stakeholder alignment essential for long-term adoption. | Environmental: Stakeholder alignment Social: HCP DHT training Technological: Availability of DHT infrastructure |
| 5:89 | Targeted strategies, training, and clear policies were required to enable adoption. | Environmental: Clear data protection policies Environmental: Clear DHT regulations Environmental: Targeted financial and infrastructure strategies Social: Continuous HCP training |
| 5:90 | Iterative involvement of HCWs and feedback loops improved design | Social: HCP engagement in DHT design |
| 5:91 | Broadband, subsidies, and training can reduce disparities. | Environmental: Broadband policy and community program Environmental: Policy and community programs addressing digital disparities Environmental: Subsidy policies and community programs Environmental: Training policies and community programs |
| 5:92 | Gamification elements like points and collectibles encouraged rehabilitation adherence. | Technological: Gamification elements |
| 5:93 | Unfamiliarity with glove controls led to feelings of helplessness and demotivation. | Psychological: Familiarity with DHT |
| 6:1 | Nurses maintained warm, supportive relationships despite remote care, but needed persuasion and empathy skills. | Social: Empathic rapport in remote care Social: HCP persuasion and empathy skills in remote care |
| 6:2 | Nurses acted as bridges, developing new digital pathways and driving innovation. | Social: HCP bridging role in digital pathways |
| 6:3 | Virtual care required motivational interviewing, careful listening, and directive counseling. | Psychological: Active listening in remote care Social: Communication skills in remote care Social: Directive counseling in remote care Social: Motivational interviewing in remote care |
| 6:4 | Broad nursing knowledge and prior clinical experience were crucial for VCC work. | Health: Broad clinical knowledge for remote care triage Psychological: Prior bedside experience as capability base |
| 6:5 | Nurses arranged their own training, highlighting the need for structured programs. | Psychological: HCP self-arranged learning Social: HCP DHT training needs |
| 6:6 | Clear, consistent, and tested protocols guided effective care delivery. | Environmental: Need for standardized remote-care protocols |
| 6:7 | Usability, integration, and reducing double registration were major challenges. | Technological: Reduction of double documentation Technological: Technology integration need Technological: Usability of DHT |
| 6:8 | Gaining trust of hospital colleagues was vital for adoption. | Social: Trust-building among HCPs |
| 6:12 | Women valued adaptive tracking with actionable feedback relevant to their phase. | Health: Maternal phase-specific actionable feedback Technological: Adaptive tracking linked to maternal milestones Technological: Need for data-driven personalized interactions |
| 6:13 | Timely, phase-specific feedback enhanced engagement and personalization | Health: Timely maternal phase-specific updates for engagement |
| 6:14 | Women wanted pregnancy and child milestones to guide decision-making. | Health: Pregnancy and child milestone-based guidance |
| 6:15 | Willingness to share personal data depended on perceived benefits and source credibility. | Psychological: Benefit-based privacy decision-making Psychological: Conditional data sharing for personalization |
| 6:16 | Women trusted DHIs more if backed by hospitals or government. | Environmental: Political and institutional backing Environmental: Trust in government Environmental: Trust in hospitals |
| 6:17 | Women wanted apps that connected them with local services. | Social: Connection to local healthcare services |
| 6:18 | Confusion about healthcare navigation created demand for localized guidance. | Social: Localized care navigation guidance |
| 6:19 | Women wanted culturally relevant diet and lifestyle information. | Cultural: Culture-specific lifestyle guidance |
| 6:20 | Sharing personal anecdotes among women created reassurance and normalization. | Social: Peer storytelling for reassurance and normalization |
| 6:22 | Poor memory, slower cognition, and lack of support made adoption harder. | Health: Memory issues Psychological: Cognitive issues Technological: Absence of DHT learning resources and assistance |
| 6:23 | Trust was built through recommendations from doctors, family, friends, and institutions. | Social: Trust through recommendations from HCP, family, peers, and institutions |
| 6:24 | Fear of phishing and misuse of Singpass data reduced confidence. | Psychological: Fear of data misuse Psychological: Fear of phishing |
| 6:25 | Monetary rewards, free devices, and gamification encouraged adoption. | Environmental: Monetary incentives Technological: Gamified rewards |
| 6:26 | Users continued only if they saw results. | Psychological: Outcome observability |
| 6:27 | Desire for longevity and holistic well-being motivated DH use. | Psychological: Desire for longevity and well-being |
| 6:28 | Generic or Western-based information reduced relevance. | Cultural: Provision of localized content Technological: Provision of personalized content |
| 6:29 | Telehealth was seen as good for mobility-limited or sick individuals. | Cultural: Preference for in-person care Environmental: Convenience for mobility-limited individuals Health: Convenience for sick individuals |
| 6:31 | Children and parents required fewer prompts after refinements, showing learnability. | Technological: Learnability of DHT Technological: User ease of use |
| 6:32 | Both groups showed confidence in navigating the interface. | Psychological: Digital confidence Psychological: User computer self-efficacy |
| 6:35 | App was seen as relevant for symptom tracking, supporting recall, and easing reporting burden. | Health: Support for health information recall Health: Symptom tracking Psychological: Responsibility relevance Technological: Ease of health information reporting |
| 6:36 | Graphs and history features helped visualize and interpret symptoms. | Technological: Demonstrability of results Technological: Graph and history features aiding symptom visualization and interpretation |
| 6:37 | Younger children benefited from emoji-based responses; older children valued data reflection. | Psychological: Data reflection Technological: Age-friendly design Technological: Emoji-based responses |
| 6:38 | Parents saw the app as beneficial for caregiving and understanding children’s experiences. | Social: Parental perceptions for value |
| 6:39 | Requests for badges, animations, and rewards show importance of gamification. | Technological: Gamification requests Technological: Requests for animations Technological: Requests for game badges Technological: Requests for game rewards |
| 6:40 | Most feasible current use is population-level data collection. | Health: Use in population-level NCD risk data collection |
| 6:41 | Long-term telemonitoring possible but faces feasibility issues. | Environmental: Constraints on feasibility of long-term telemonitoring |
| 6:42 | Existing HIS not designed for NCDs and integration of wearable data. | Technological: Lack of DHT readiness for NCD data integration |
| 6:43 | Questions about accuracy, battery life, and resilience limited trust. | Psychological: Skepticism about DHT measurement accuracy Technological: Battery life Technological: DHT resilience |
| 6:44 | Customization was seen as critical but limited by proprietary restrictions. | Technological: Open API requirement for local customization |
| 6:45 | Uptake harder among elderly, farmers, and those unfamiliar with tech; training needed. | Psychological: Influence of age Social: Influence of occupation Social: Stakeholder DHT training |
| 6:46 | Risk of reinforcing inequalities if poorer groups cannot afford wearables. | Environmental: Cost of equipment or device |
| 6:47 | Internet and electricity gaps restricted usability, especially in rural areas. | Environmental: Electricity gaps Environmental: Internet connectivity |
| 6:48 | Weak regulatory framework and lack of health data privacy laws. | Environmental: Availability of data integrity regulations Environmental: Availability of data safety regulations Technological: Availability of DHT regulations |
| 6:49 | Donor fragmentation, lack of coordination, and limited national ownership threaten long-term adoption. | Environmental: National ownership limitations Environmental: Risk of program fragmentation |
| 6:50 | Physical and cognitive decline, along with negative self-perceptions, hindered learning and sustained use. | Health: Physical health issues Psychological: Cognitive issues Psychological: Internalized ageism |
| 6:51 | Illiteracy, language barriers, and social status reinforced feelings of inadequacy and reluctance to learn. | Cultural: Language barriers Cultural: Stigma barrier to DHT use Environmental: Social precarity Psychological: General literacy constraints |
| 6:52 | Smartphones’ design (small fonts, sensitivity, complex apps) conflicted with older adults’ priorities and needs. | Health: Screen sensitivity Psychological: User priorities and needs conflict Technological: Apps complexity Technological: Font size Technological: Technological design |
| 6:53 | Smartphones were seen as unnecessary or irrelevant to daily life and priorities of older age. | Psychological: Perception of lifestyle–technology mismatch |
| 6:54 | Interactions with volunteers and learning communication features created social connectedness. | Social: Volunteer tutoring as support scaffold |
| 6:55 | Learning video calls and apps helped maintain relationships and reduce isolation. | Social: Isolation reduction through video call skills |
| 6:56 | Relevance to participants’ lived experiences and goals was essential for sustained motivation. | Psychological: Life goal alignment Psychological: Personally relevant leaning content |
| 6:57 | Interventions should dismantle ageist stereotypes and empower older adults. | Cultural: Anti-ageism pedagogy |
| 6:58 | Lack of continued practice reduced motivation for sustained use post-intervention. | Environmental: DHT program continuity and practice needs |
| 6:59 | Wearables automatically logged activities, allowing teachers to engage without extra effort. | Technological: Convenience of passive data collection Technological: Passive activity logging reducing effort |
| 6:60 | Quick and simple interactions reduced burden and made use feasible in busy contexts. | Environmental: Convenience in busy contexts |
| 6:61 | Subtle appearance and unobtrusive notifications allowed teachers to use technology without stigma. | Technological: Stigma reduction interface |
| 6:62 | Prompts and congratulatory messages encouraged awareness and self-care. | Technological: In-app nudges for reflection and self-care |
| 6:63 | Visual patterns and quantifiable metrics helped teachers connect stress to specific contexts. | Technological: Visual analytics for stress awareness |
| 6:64 | Chatbot interactions provided language to support colleagues and educational insights. | Technological: Chatbot support features |
| 6:65 | Teachers struggled due to poor setup information, relying on trial-and-error and YouTube. | Social: Practical task guidance |
| 6:66 | Users worried about discrepancies and absence of benchmarks for comparison. | Health: Concern about missing data reference values or standards Health: Concerns about inconsistent or conflicting DHT results |
| 6:67 | Limited Bluetooth range and phone proximity created difficulties. | Technological: Bluetooth and phone proximity constraints |
| 6:69 | Teachers emphasized importance of normalizing stress management and providing private spaces. | Cultural: Organizational culture for stress-related privacy Environmental: Provision of private spaces for use |
| 6:70 | Utility in improving diagnostics, reducing errors, and optimizing decision-making strongly predicted adoption intention. | Health: HCP decision-support benefits Health: Improvement of diagnostic accuracy in remote care Psychological: HCP perceived benefits |
| 6:71 | High costs in infrastructure, training, and workflow changes reduced positive attitudes and intention. | Environmental: Financial expenses in DHT implementation Social: HCP DHT training Technological: HCP workflow change |
| 6:74 | Perceived effort costs reduced perceived value of AI-CDSSs. | Psychological: Perceived cost reducing benefit valuation |
| 6:75 | Peer, organizational, and guideline support fostered acceptance and intention. | Environmental: DHT practical guidelines and ethical standards Environmental: Organizational endorsement Social: Peer endorsement |
| 6:77 | Intuitive design, pilot programs, and training reduce barriers and enhance adoption. | Environmental: Availability of pilot programs Social: HCP DHT training Technological: Intuitive interface design |
| 6:80 | Concerns about unintended smartphone exposure. | Health: Overuse risk of smartphone accessibility for children |
| 6:81 | Viewed as adjuncts to therapy, extending connection beyond sessions. | Health: Therapeutic continuity between sessions |
| 6:82 | Professionals emphasized in-person care as primary. | Cultural: Centrality of in-person care |
| 6:83 | Apps required compatibility with IT systems and training. | Social: HCP DHT training Technological: Health system compatibility |
| 6:85 | Fears tech may replace rather than complement services. | Psychological: Fear of technology replacing service for savings |
| 6:87 | Telehealth was seen as useful for health management, safety, and quality of life. | Health: Health management and technology use Psychological: Quality-of-life enhancement Psychological: User perceived usefulness |
| 6:89 | Age-friendly visuals, minimal buttons, and multimodal guidance enhanced adoption. | Technological: Age-friendly design Technological: Multimodal user guidance |
| 6:90 | Appointment unavailability, internet issues, and fear/dislike hindered adoption. | Cultural: Cultural fear or dislike preference Environmental: Internet connectivity Health: Timeliness of access to care and results |
| 6:91 | Higher education, professional work history, and health insurance increased adoption. | Environmental: Health insurance Psychological: Education level Psychological: Professional work history |
| 6:93 | Older adults needed education, workshops, and personalized support to overcome barriers. | Social: Community- and hospital-based DHT workshops Social: One-to-one DHT guidance and troubleshooting Social: Structured DHT teaching and awareness |
| 6:94 | Parents rated the app highly useful for supporting symptom recognition and response | Health: Support for symptom recognition and response Psychological: User perceived usefulness |
| 6:95 | Children enjoyed customizing avatars and interacting with features, increasing engagement. | Technological: Avatar customization Technological: Interactive user experience features |
| 6:96 | Children and parents endorsed features like drawing and customization as enjoyable. | Psychological: User perceived enjoyment Technological: Drawing and customization features Technological: Gamified personalization |
| 6:97 | Teachers found it difficult to engage with technology in the school environment. | Environmental: Competing demands of school and workplace |
| 6:98 | Favourable perceptions significantly shaped adoption. | Social: HCP attitude toward DHT |
| 6:99 | Recognized clinical advantages increased favourable attitudes. | Health: Recognition of clinical advantages |
| 6:100 | Explainability and clinician oversight boosted confidence and adoption. | Psychological: Perceived control and transparency over AI |
| 6:101 | Mobile apps enhanced access, engagement (esp. youth), and crisis support. | Environmental: Improved healthcare service accessibility |
| 6:102 | Digital tools helped those uncomfortable with face-to-face services. | Social: Accessibility for hard-to-reach populations |
| 6:103 | Usability, reliability, and safeguarding concerns | Health: Data security and privacy concerns Psychological: Trust in DHT |
| 6:104 | Professionals wanted guidance, evidence, and training. | Environmental: Support from DHT policies and protocols Health: Clinical and implementation evidence Social: HCP DHT training |
| 6:105 | Resource availability, guidance, and support encouraged positive attitudes. | Social: Availability of help in DHT usage Social: Instructional aids and professional demonstration guidance Technological: Availability of technical resources |
| 6:106 | Subsidies, infrastructure, and training for older adults recommended. | Environmental: Financial subsidies for DHT implementation Social: HCP DHT training Social: User DHT training Technological: Availability of DHT infrastructure |
| 7:1 | Older adults’ technology use is influenced by age-related factors such as digital literacy, motivation, and co-morbidities. | Health: Comorbidity-related barriers Psychological: Age-related reluctance and decline in motivation Psychological: Digital health literacy level |
| 7:2 | Older adults value usefulness over novelty, and perceive time cost in learning. | Psychological: Perceived time cost of learning DHT Psychological: Priority of usefulness over novelty |
| 7:3 | Many older adults avoid or delay using technology by underestimating their condition or needs. | Psychological: Barriers from denial of health condition |
| 7:4 | Families often drive adoption, provide technical support, and act as motivators. | Social: Family technical assistance support |
| 7:5 | Trust in family, healthcare professionals, and system reliability is crucial for adoption. | Social: Trust in family Social: Trust in healthcare professionals Technological: Trust in system reliability |
| 7:7 | Older adults require information and functions tailored to individual needs and preferences. | Technological: DHT functions tailored to individuals needs and preferences Technological: Provision of personalized health information |
| 7:8 | Poverty and cost of devices, maintenance, and connectivity hinder adoption. | Environmental: Affordability for DHT Environmental: Cost for DHT maintenance Environmental: Cost of equipment or device Technological: Technical connectivity |
| 7:10 | Older adults worry about big data, AI, and selling of patient data. | Health: Data security and privacy concerns Psychological: Fear of data misuse |
| 7:11 | Older adults reject clinical-looking devices; design must reduce stigma and increase desirability. | Cultural: Stigma from clinical looking devices Technological: Interface attractiveness |
| 7:15 | Continuous support and reassessment of changing needs are essential. | Psychological: Reassessment of changing needs Social: Continuous user support |
| 7:16 | Technology must be adaptable to individual lives and homes. | Psychological: Adaptability to daily life and environments |
| 7:17 | Users want autonomy in deciding who can access their health data. | Health: User control over health data Psychological: User autonomy in data sharing |
| 7:19 | Older adults seek reassurance from trusted organisations like NHS. | Environmental: Reassurance from trusted organizations |
| 7:20 | Sharing data with clinical teams improves collaboration and communication. | Social: Data sharing through HCP collaboration Social: Improved patient-provider communication |
| 7:21 | Respect for privacy encourages users to share data with peer groups for support. | Social: Motivation from private group data exchange Social: Peer support through safe sharing |
| 7:22 | Clinical-looking or disability-friendly devices increase stigma and reduce adoption. | Cultural: Stigma from disability-oriented design Technological: Age-friendly design |
| 7:24 | Young people are aware of DMH through apps, podcasts, blogs, and social media. | Social: DHT awareness through social media |
| 7:25 | Users want apps that combine AI with real human interaction and long-term support. | Cultural: Preference for hybrid AI–human interaction Psychological: Support for consistent and continuous use |
| 7:26 | Lack of smartphones, internet-enabled gadgets, and poor connectivity hinder use. | Environmental: Internet connectivity Environmental: Internet-enabled devices Environmental: Smartphone ownership |
| 7:27 | DMH mainly benefits urban, literate groups, excluding rural and illiterate populations. | Environmental: Rural inequalities Psychological: General literacy constraints |
| 7:28 | High cost of apps and payment methods limit adoption. | Environmental: Barrier to accessibility of payment methods Environmental: Cost of apps |
| 7:29 | Cultural beliefs, fear of judgment, and misconceptions hinder openness. | Cultural: Cultural stigma against mental illness Psychological: Fear of judgement in DHT use |
| 7:30 | Poor usability, irrelevant content, and lack of localization discourage use. | Cultural: Content irrelevance to local context Cultural: Language barriers Technological: Usability barrier from poor design |
| 7:31 | Users worry about personal data safety and trust in app providers. | Health: Confidentiality concerns in sensitive apps Psychological: Distrust in app providers |
| 7:32 | Need for campaigns to educate and create awareness of DMH. | Social: Community sensitization on DHT Social: DHT awareness campaigns |
| 7:33 | More funding, regulation, and toll-free access are needed. | Environmental: Affordable healthcare access Environmental: Government funding support Environmental: Support from structured regulatory frameworks |
| 7:34 | Capacity building at all levels, especially village health teams. | Social: HCP DHT training |
| 7:35 | Young people want free, simple, and accessible apps. | Environmental: Preference for free apps |
| 7:37 | Some applications were difficult to navigate with unfriendly interfaces. | Technological: DHT usability issues Technological: Navigation complexity |
| 7:38 | Husbands discouraged use of eHealth. | Social: Family-level resistance Social: Negative spousal influence |
| 7:39 | Systems were developed without engaging mothers, leading to low uptake. | Social: Co-design with specific groups |
| 7:40 | Systems failed to connect mothers to doctors. | Social: Failure in HCP-patient communication channel |
| 7:42 | Users were frustrated when systems failed. | Social: Technical support |
| 7:43 | Providers lacked time, motivation, or digital skills. | Environmental: HCP time constraints Psychological: HCP motivation to use Social: HCP digital skills gap |
| 7:44 | Internet bundles were unaffordable for most mothers. | Environmental: Internet bundle cost |
| 7:45 | Slow and unreliable internet hindered usage. | Environmental: Internet connectivity |
| 7:46 | Load-shedding and high tariffs limited device usage. | Environmental: Electricity gaps |
| 7:47 | Many mothers lacked smartphones or computers. | Environmental: Computer ownership Environmental: Smartphone ownership |
| 7:48 | Low literacy and language barriers prevented adoption. | Cultural: Language barriers Psychological: General literacy constraints |
| 7:49 | Mothers did not see the value of eHealth for ANC. | Psychological: Awareness of DHT value |
| 7:50 | Users doubted sustainability due to failed pilots. | Psychological: Doubt about DHT sustainability due to failed pilots |
| 7:51 | Fear of privacy breaches reduced willingness to adopt. | Psychological: Fear of privacy breaches |
| 7:52 | Poor ICT infrastructure, unstable networks, server downtime, and lack of notifications hindered system performance. | Environmental: Internet connectivity Environmental: Quality of available ICT infrastructure Technological: Absence of notifications Technological: Server downtime |
| 7:53 | Lack of timely onsite experts disrupted system use. | Technological: Availability of onsite technical expertise |
| 7:54 | Inadequate funding, power instability, shortage of radiologists and technical staff, and weak administrative support threatened sustainability. | Environmental: Budget constraint Environmental: Electricity gaps Environmental: Shortage of HCPs and technical staff Social: Availability of administrative support |
| 7:55 | Lack of regulations, unclear system ownership, and weak institutionalization created sustainability risks. | Environmental: Unclear ownership of systems Environmental: Weak institutionalization Technological: Availability of DHT regulations |
| 7:56 | Low awareness, lack of readiness, limited technical skills, and resistance to adoption impeded use. | Psychological: DHT adoption readiness Psychological: HCP resistance to adoption Social: HCP awareness of DHT |
| 7:57 | Delays in consultations and report submissions, and poor system integration caused inefficiency. | Social: Delays in consultations and report submissions Technological: Poor system integration |
| 7:59 | Web-based access enabled remote consultation, collaboration, and continuous learning. | Environmental: International collaboration Social: Access to remote consultation Social: Continuous HCP learning |
| 7:60 | Standardized reporting, confidentiality, and user trust promoted adoption. | Health: Assurances of confidentiality Technological: Standardized reporting Technological: Trust in data security |
| 7:61 | Patients saw HM as giving them personal control over vision health. | Psychological: Patient empowerment through self-monitoring |
| 7:62 | HM seen as beneficial if it could reduce clinic appointments. | Environmental: Convenience through reduced visits and travel |
| 7:64 | Some saw HM as necessary despite unfamiliarity with devices. | Psychological: Digital literacy adaptation Psychological: Overcoming hesitancy due to necessity |
| 7:66 | Tests with feedback (MBT) were preferred as motivating. | Psychological: Feedback motivating continued use |
| 7:67 | Regular HM feasible when integrated into routines and reminders. | Psychological: Integration into health routines Psychological: Reminder-based adherence |
| 7:68 | Relatives often provided technical and motivational help. | Social: Relatives’ motivational help Social: Relatives’ technical assistance |
| 7:69 | Device setup, testing conditions, or uncertainty about data transmission caused frustration. | Environmental: Testing environment barriers Technological: Barriers in account setup and management Technological: Uncertainty of data transmission |
| 7:70 | Tremors, fatigue, caregiving responsibilities disrupted HM. | Health: Physical health issues Psychological: Caregiving competing priorities |
| 7:72 | Discontinuity of care reduced confidence in systems. | Social: Lack of continuity with HCP Social: Trust erosion due to care gaps |
| 7:73 | Systems must adapt to unique conditions, medication regimes, and digital literacy levels. | Psychological: Adaptability to diverse needs Technological: Personalisation for diverse needs Technological: Usability for diverse needs |
| 7:74 | Mandatory treatment led to concealment and resistance. | Psychological: Loss of autonomy from surveillance Psychological: Resistance and concealment behaviours |
| 7:75 | Consumers emphasized transparent, explicit, ongoing consent for digital monitoring. | Cultural: Transparency for personhood Health: Consent and boundary setting |
| 7:76 | Carers wanted involvement but highlighted the need for clear consent and boundaries. | Health: Consent boundary setting Social: Demand for carer involvement |
| 7:77 | Consumers lacked access to clear information on side effects and interactions. | Health: Education gap on side effects Health: Lack of medication information |
| 7:78 | Risk of harmful over-monitoring, sustainability issues, and mixed feelings about carer involvement. | Environmental: Sustainability concern Health: Risk of over-monitoring |
| 7:79 | Carer contributions and peer involvement strengthen wellness and recovery. | Social: Peer support Social: Recognition of carer contributions |
| 7:80 | Consumers wanted systems that respect dignity and avoid objectification as “data.” | Cultural: Preservation of dignity Health: Person-centred digital care |
| 7:81 | Technology should enhance collaboration, not enforce compliance. | Social: HCP-patient collaboration enhancement Social: Shared responsibility in care |
| 7:82 | Patients valued DTx for coping and self-management during long waits for therapy. | Psychological: Coping support during waiting periods Psychological: Self-management during therapy waiting periods |
| 7:83 | DTx considered more reliable than generic internet sources. | Health: Provision of secure and validated information Health: Trust in evidence-based and safe content |
| 7:84 | Patients expected chat or communication tools to provide guidance. | Social: Professional guidance via chat or tools |
| 7:85 | DTx valued for privacy in mental health contexts. | Psychological: Preference for anonymity in digital health use Social: Benefit of stigma reduction |
| 7:86 | DTx seen as helpful but less effective than in-person care. | Cultural: Preference for face-to-face care Health: Nonmaleficence and potential harms |
| 7:87 | Digital tools may trigger or worsen mental health symptoms. | Health: Nonmaleficence and potential harms |
| 7:89 | Less tech-savvy groups may face barriers | Psychological: Digital health literacy level |
| 7:92 | GPs seen as gatekeepers for prescriptions, even if not always consulted. | Social: HCP as prescription gatekeeper |
| 7:93 | Interest in DTx depended on health status and coping stage. | Health: Influence of disease stage Psychological: Disease coping stage |
| 7:95 | Older adults recognized their health problems and wanted ongoing monitoring. | Health: Chronic disease management needs Health: Demand for ongoing care monitoring |
| 7:96 | Participants actively searched information from Google, YouTube, radio, newspapers, and doctors. | Psychological: Multi-source information seeking |
| 7:97 | Many resisted digital health and preferred direct clinic visits. | Cultural: Preference for in-person visits Cultural: Resistance to digital substitutes |
| 7:98 | Many older adults lacked ability to use apps and smartphones. | Psychological: Digital literacy level |
| 7:99 | Participants acknowledged potential benefits of devices like smart bands and apps. | Psychological: User perceived benefits |
| 7:100 | Children and caregivers played key roles in teaching and helping older adults use technology. | Social: Family assistance support |
| 7:103 | Setup time, glitches, cybersecurity, and privacy issues reduced efficiency. | Health: Data security and privacy concerns Technological: Barriers in account setup and management Technological: Cybersecurity concern |
| 7:104 | Exposure and uptake differed by pharmacy type and business model. | Environmental: Business model limitations in service scope Technological: Workflow fit |
| 7:105 | Telepharmacy enabled remote consultations, refills, chart reviews, and counselling. | Environmental: Service expansion opportunity Social: Continuity of remote consultation |
| 7:106 | Useful for mobility-limited patients and those isolating. | Environmental: Convenience during isolation or quarantine Environmental: Convenience for mobility-limited individuals |
| 7:107 | Lack of physical exams and non-verbal cues restricted effectiveness. | Health: Limited diagnostic capacity in remote care Social: Risk of miscommunication |
| 7:108 | Harder to build trust virtually compared to face-to-face. | Psychological: Empathy challenges with DHT Social: Reduced rapport in virtual care |
| 7:110 | Older pharmacists and managers preferred traditional methods. | Cultural: HCP preference for traditional tools Psychological: Influence of age |
| 7:112 | Need for unified health database, better platforms, and user-friendly systems | Technological: Lack of unified health database Technological: Platform integration gap |
| 7:113 | Staff shortages, workload, and lack of telehealth training hindered use. | Environmental: HCP workforce shortage Social: HCP DHT training |
| 7:115 | Lack of guidance and poor communication from government created challenges. | Environmental: Government guidance Environmental: Institutional support |
| 7:116 | Lack of remuneration and financial support threatened sustainability. | Environmental: Availability of financial support Environmental: Remuneration availability |
| 7:117 | Telepharmacy could reduce disease spread and expand rural care. | Environmental: Rural healthcare access Health: Disease prevention benefits |
| 7:118 | COVID-19 normalized telehealth and increased acceptance. | Cultural: Pandemic-driven social acceptance |
| 7:119 | The quantity, timing, and framing of information affects adoption. | Health: Appropriateness of health information Health: Timing of health information provision Psychological: Framing of health information Psychological: Health information overload Technological: Quantity of health information |
| 7:120 | Successful use requires onboarding, continuous support, and guidance for both patients and carers. | Social: User continuous support on DHT use Social: User DHT guidance Social: User DHT training |
| 7:121 | A major barrier to adoption is lack of skills and confidence among older adults. | Psychological: Digital confidence Psychological: Digital literacy level |
| 7:122 | Poverty and cost hinder access, maintenance, and connectivity. | Environmental: Affordability for DHT Environmental: Cost for DHT maintenance Environmental: Cost of equipment or device Technological: Technical connectivity |
| 7:123 | Initial setup and training are crucial for building trust and long-term use. | Social: User onboarding training |
| 7:124 | Current technology development lacks diversity and inclusivity. | Cultural: Gaps in diversity and inclusion |
| 7:125 | Mothers saw little value in current eHealth systems for maternal health. | Psychological: User perceived utility |
| 7:126 | Absence of training created barriers to adoption. | Social: User pre-usage training |
| 7:127 | Teleradiology enhanced quality, reduced misdiagnosis, and saved time and costs. | Environmental: HCP time savings Environmental: HCP travel cost savings Health: Improvement of diagnostic accuracy in remote care Health: Improvement of healthcare service quality |
| 7:128 | Concerns about inability to access clinics despite HM. | Social: Appointment access delays Technological: Limited system responsiveness |
| 7:129 | Patients struggled to recall training without refreshers. | Social: Refresher training need |
| 7:130 | Consumers faced barriers from lack of devices and affordable data. | Environmental: Affordable data barrier Environmental: DHT device availability |
| 7:131 | Patients valued low-threshold access compared to traditional therapy. | Environmental: Low-threshold entry to care Social: Advantage of flexible access |
| 7:132 | GP advice strongly influenced DTx uptake for some patients. | Social: HCP recommendations |
| 7:133 | Some patients felt GPs lacked knowledge or empathy about digital tools. | Psychological: Empathy challenges with DHT Psychological: HCP knowledge on DHT |
| 7:134 | Patients with long therapy history saw little added value. | Health: Long-term therapy experience |
| 7:135 | Devices must be physically comfortable, adjustable, and simple to use. | Technological: Ease of adjustment for usability Technological: Wearability comfort |
| 7:136 | Older adults preferred appropriate colors, large fonts, and simple layouts. | Technological: Preference for simple and accessible design Technological: Visual usability considerations |
| 7:137 | Implementation required feasibility studies, feedback, and standardisation. | Environmental: Implementation planning need Social: Feedback for service design |
| 7:139 | Device, internet, literacy, disability, and language issues limited adoption. | Cultural: Language and cultural accessibility Environmental: Internet connectivity and affordability Health: Disability-related barriers Psychological: Digital literacy level Technological: Availability and compatibility of technical devices |
| 7:140 | Lack of supportive laws, unclear policies, and inadequate guidance. | Environmental: DHT policy Environmental: Professional guidance and standards for DHT Environmental: Supportive legal frameworks for DHT |
| 8:1 | E-Pharmacy helped overcome geographic barriers and busy schedules. | Environmental: Convenience in busy contexts Environmental: Rural healthcare access |
| 8:2 | Digital platforms simplified managing chronic medications. | Health: Support for medication adherence Technological: Digital prescription management |
| 8:3 | Lower prices, discounts, and transparency attracted adoption. | Environmental: Benefit of price transparency Environmental: Cost savings through discounts |
| 8:4 | Doorstep delivery and tracking features enhanced confidence. | Environmental: Reliability of doorstep delivery Psychological: Reassurance from tracking features |
| 8:5 | Fear of counterfeit medicines and loss of pharmacist interaction reduced adoption. | Health: Risk of counterfeit drugs Social: Reduced pharmacist interaction |
| 8:7 | Poor connectivity and high costs hindered rural users. | Environmental: Internet connectivity Environmental: Internet cost barrier |
| 8:8 | Fears of hacking and data breaches reduced trust. | Psychological: Fear of data breaches Psychological: Fear of hacking |
| 8:9 | Doctors lacked knowledge about DiGAs and their benefits, creating mistrust. | Psychological: HCP knowledge on DHT |
| 8:11 | Absence of official medical certification reduced physician confidence. | Health: Absence of medical certification |
| 8:12 | Doctors wanted more engagement post-prescription and in development. | Social: HCP exclusion from design Social: Limited post-prescription role |
| 8:13 | Low reimbursement discouraged doctors from prescribing DiGAs. | Environmental: Insufficient reimbursement Environmental: Low prescribing incentive |
| 8:14 | Doctors varied in openness and competence with digital tools. | Psychological: Digital health literacy level Psychological: Openness to new technologies |
| 8:15 | Many patients had never heard of DiGAs and lacked awareness. | Psychological: Awareness of DHT |
| 8:16 | Patients expressed both confidence in using apps and in managing health. | Psychological: Health self-efficacy Psychological: Technology self-efficacy |
| 8:17 | Disease severity and susceptibility motivated adoption. | Health: Disease severity Psychological: Perceived susceptibility |
| 8:19 | Patients wanted autonomy in data sharing but trusted certified systems. | Health: Trust in medically certified systems Psychological: User autonomy in data sharing |
| 8:20 | Patients valued reduced visits, better monitoring, and more information. | Environmental: Convenience through reduced visits and travel Health: Health monitoring benefits Technological: Provision of more health information |
| 8:21 | Technical and health support were important for sustained use. | Social: Availability of health support Social: Technical support |
| 8:22 | Doctors often excluded older patients, assuming low digital skills. | Social: Ageism among HCP |
| 8:23 | Caregivers preferred multimodal (voice + text) options depending on context. | Technological: Availability of multimodal user modes |
| 8:24 | Caregivers used smart technologies for reminders, organization, and scheduling. | Environmental: Organizational support Social: Scheduling support Technological: Reminder facilitation |
| 8:25 | Caregivers wanted VAs to recall past queries and personalize responses. | Technological: Personalized responses Technological: Search history need |
| 8:27 | Caregivers were overwhelmed by excessive online searching. | Psychological: Search fatigue |
| 8:28 | Caregivers wanted niche, accurate oncology-related answers. | Health: Evidence-based guidance Health: Request for accurate oncology-specific information |
| 8:29 | VAs perceived as tools to reduce exhaustion and provide quick answers. | Social: Reduction of caregiving burden Technological: Convenience of quick answers |
| 8:31 | Caregivers emphasized secure, private VA systems. | Health: Assurances of confidentiality Health: Data security and privacy concerns |
| 8:33 | Concerns that caregivers may trust VA guidance too much without consulting doctors. | Health: Risk of carer over-reliance on DHT Psychological: Misinformation vulnerability |
| 8:34 | Rural caregivers saw greater value for access and learning. | Environmental: Rural healthcare access |
| 8:35 | Clinicians doubted the accuracy and calibration of patient devices. | Health: Data reliability concern Technological: Device calibration issue |
| 8:38 | Low digital literacy and cognitive issues created risks. | Psychological: Cognitive issues Psychological: Digital health literacy level |
| 8:46 | Caregivers expressed interest in digitizing but found apps unusable or rigid. | Psychological: Interest in DHT Technological: App usability limitations |
| 8:51 | COVID-19 reduced support services and increased caregiver isolation. | Environmental: Impacts of COVID-19 on home care services Social: Caregiver isolation |
| 8:52 | Tools must reduce duplication, adapt to changing needs, support customization, and be intuitive. | Technological: Need for customizable design Technological: Real-time linkage with providers |
| 8:53 | Patients valued access to results to manage their care. | Psychological: Patient empowerment through self-care |
| 8:54 | Some patients valued seeing results, others felt anxious. | Psychological: Anxiety from health data Psychological: Challenge of result interpretation |
| 8:55 | Home monitoring seen as more convenient than hospital visits. | Environmental: Convenience through reduced visits and travel Health: Convenience of home testing |
| 8:56 | Device adoption depends on simplicity, training, and feedback. | Social: User DHT training Social: User training with feedback Technological: Preference for simple DHT |
| 8:57 | Patients wanted discreet, non-clinical devices. | Cultural: Preference for non-clinical design Psychological: Stigma avoidance |
| 8:59 | Concerns about elderly, digitally naive, or unsupported patients. | Environmental: Digital divide barrier Environmental: Equity of access issue |
| 8:60 | Importance of real-time reporting, clinician review time, and interoperability. | Social: Clinician review workload Technological: Cross-system interoperability |
| 8:61 | Some patients still preferred in-person reassurance and clinician interpretation. | Cultural: Preference for in-person clinician interpretation Cultural: Preference for in-person reassurance |
| 8:62 | CALD participants had little exposure to patient portals, even when available. | Psychological: Awareness of DHT |
| 8:63 | Seniors expressed difficulty with digital tools, while younger people were more confident. | Psychological: Age-based confidence gap Psychological: Digital health literacy level |
| 8:65 | Limited English proficiency and lack of translation support restricted use. | Cultural: Language barriers Cultural: Translation needs |
| 8:68 | Hospitals often did not offer portals even when available. | Environmental: Organizational access gap Technological: Portal availability and utilization |
| 8:69 | CALD participants sometimes preferred clinicians of same cultural background. | Cultural: Preference for cultural concordance |
| 8:71 | Non-Medicare patients struggled with costs, which portals could potentially reduce. | Environmental: Relief of cost burden for migrants |
| 8:74 | Patients expected doctors to be familiar with health apps like HealthHub. | Social: Expectation of HCP DHT knowledge |
| 8:78 | Doctors should know when to refer and assess patients holistically. | Environmental: Expectation of escalation protocols Health: Holistic health assessment need |
| 8:79 | Patients valued efficiency over eye contact when using EHRs. | Cultural: Preference for efficiency over eye contact |
| 8:80 | Patients emphasized empathy should remain central. | Social: Empathic rapport in remote care |
| 8:81 | Younger participants wanted doctors trained in AI for accuracy. | Social: Expectation of HCP AI competence |
| 8:84 | Patients valued anytime, anywhere access. | Technological: Accessibility of DHT Technological: Availability of DHT |
| 8:86 | Users found the application simple and similar to other familiar apps. | Psychological: Familiarity through DHT similarity Technological: Preference for simple DHT |
| 8:87 | High-speed internet, quiet spaces, and smartphones were essential. | Environmental: Device availability need Environmental: Internet connectivity |
| 8:89 | Users trusted the Ministry of Health as provider. | Environmental: Institutional trust Health: Data security and privacy concerns |
| 8:90 | Users valued freedom to choose consultation mode and control data. | Health: User control over health data Psychological: Consultation choice autonomy |
| 8:91 | Patients felt capable of explaining health conditions online. | Psychological: Online communication competence Psychological: Patient self-efficacy |
| 8:92 | Users felt connected to doctors and peers. | Social: Patient-HCP relatedness Social: Peer connection via app |
| 8:93 | Users were motivated by family, doctors, and desire to understand their health condition. | Psychological: Health condition awareness driven adoption Social: HCP recommendations |
| 8:94 | Strong desire to connect with the same or preferred doctor for follow-up. | Psychological: Continuity of care motivation Social: Preferred access to doctor |
| 8:95 | Clinicians were motivated by efficiency, awareness of RM benefits, and improved process quality. | Environmental: Improvement of HCP workflow efficiency Psychological: Awareness of DHT |
| 8:97 | Adoption required horizontal integration, coordination, and flexible organizational structures. | Environmental: Flexible organizational structures Technological: Need for horizontal integration |
| 8:98 | Resistance arose from fear, lack of skills, and data burden, but could be reduced by support and clarity. | Psychological: Fear of technology as a barrier Social: User DHT training |
| 8:101 | Consumers valued tools that allowed them to track health and initiate discussions with professionals. | Health: User control over health data Psychological: Patient empowerment through self-monitoring |
| 8:104 | Consumers prioritized instrumental support (how-to, action guidance) over informational, emotional, or esteem support. | Social: Preference for support hierarchy Social: Priority for instrumental support |
| 8:106 | Apps enabling photo monitoring, reminders, and proactive features increased motivation. | Psychological: Reminder-driven motivation Technological: Ease of photo tracking |
| 8:108 | Digitization helps avoid delays and risks in emergencies. | Health: Emergency access benefit |
| 8:111 | Positive eWOM and reviews influenced intention modestly. | Social: Influence of peer reviews Social: Observability of electronic word-of-mouth (eWOM) |
| 8:112 | Confidence in a government-backed app encouraged adoption. | Environmental: Government credibility Environmental: Institutional trust |
| 8:113 | Concerns about cyber risks reduced confidence in the platform. | Psychological: Cybersecurity risk perception Psychological: Internet trust limitations |
| 8:114 | Absence of clear frameworks created apprehension. | Environmental: Absence of structured regulatory frameworks |
| 8:115 | Poor digital infrastructure limited prescription potential. | Technological: Availability of digital infrastructure |
| 8:116 | Doctor-patient trust was a precondition for considering DiGAs. | Social: Doctor-patient trust |
| 8:117 | Multi-language options and educational support for low-literacy users valued. | Social: Educational support needs Technological: Multi-language options |
| 8:118 | Caregivers trusted VAs linked to hospitals and clinicians. | Environmental: Institutional endorsement as source of trust Psychological: Perceived reliability of health information |
| 8:119 | VAs should direct users when professional consultation is needed. | Health: System-generated safety alerts for user Health: User ethical escalation prompts |
| 8:120 | Lack of strong clinical evidence and guidelines prevented adoption. | Health: Lack of clinical evidence |
| 8:121 | Limited staff, time, and infrastructure hindered data integration. | Environmental: HCP time constraints Environmental: HCP workforce shortage Technological: Availability of DHT infrastructure |
| 8:123 | Pulmonary rehab and outreach programs can provide DHT training. | Social: User DHT training |
| 8:124 | Clinician–patient discussions at setup stage critical for engagement. | Social: HCP–patient onboarding conversations |
| 8:125 | Clinicians emphasized their own need for digital training. | Social: HCP DHT training needs |
| 8:126 | Suitability of DHT should depend on individual patient characteristics. | Technological: Personalization tailoring need |
| 8:127 | Some patients prefer direct clinician interaction over devices. | Cultural: Preference for in-person interaction |
| 8:128 | Caregivers constantly sought knowledge from professionals, media, peers, and self-learning. | Psychological: Knowledge-seeking behaviour |
| 8:129 | Caregivers felt pressure to maintain extensive paper-based records, which was exhausting | Environmental: Caregiver paper record burden |
| 8:130 | Caregivers struggled to filter and deliver the right info at the right time. | Psychological: Information-sharing burden |
| 8:131 | Teaching new caregivers consumed significant time and effort. | Social: Caregiver training burden in information management |
| 8:132 | Caregivers struggled with inconsistent methods and unsafe practices. | Social: Inconsistent communication practices Social: Unsafe school communication |
| 8:133 | Paperwork, payroll, and hiring processes added to stress. | Environmental: Administrative load |
| 8:134 | Concerns about device failure, out-of-range results, and support availability. | Environmental: Requirement for safety nets Technological: Backup system needs |
| 8:135 | Participants worried about sensitive information being online. | Health: Data security and privacy concerns Psychological: Fear of data misuse |
| 8:136 | Many participants valued physical presence of clinicians despite portal benefits. | Cultural: Preference for in-person care |
| 8:137 | Many relied on relatives to navigate systems due to language/tech barriers. | Social: Relatives' support in navigating systems |
| 8:138 | Lack of mandates and weak evaluation limited portal rollout. | Environmental: DHT policy Technological: Availability of DHT regulations |
| 8:139 | Participants suggested linking portals to MyGov, multilingual support, and immediate benefits. | Technological: Cross-system interoperability Technological: Immediate output visibility Technological: Multilingual support |
| 8:140 | Patients wanted doctors to guide them on safe apps and work as partners. | Social: HCP guidance requirement for DHT use Social: HCP-patient collaboration expectation |
| 8:141 | Patients wanted doctors to review and explain wearable/device results. | Social: HCP guidance on anomaly explanation Social: HCP guidance on result review |
| 8:142 | Patients worried about insecure sharing of scans via WhatsApp. | Health: Data security and privacy concerns |
| 8:143 | Patients expected undivided attention and safe, accurate care. | Social: Attention and safety needs in remote consultation |
| 8:144 | Older participants distrusted DHTs and preferred doctor oversight. | Psychological: Skepticism toward AI Psychological: Trust in DHT |
| 8:145 | Teleconsultation reduced travel, waiting, and consultation expenses. | Environmental: User cost savings Environmental: User time savings |
| 8:146 | Family, friends, and doctors’ recommendations motivated use. | Social: Family recommendations Social: HCP recommendations Social: Peer recommendations |
| 8:147 | Orientation and guidance improved adoption. | Social: Orientation facilitation |
| 8:148 | Age, digital skills, and patient literacy shaped adoption attitudes. | Psychological: Digital literacy level Psychological: Influence of age |
| 8:150 | Consumers avoided seeking doctors unless absolutely necessary, viewing preventive checks as resource strain. | Cultural: Attitude toward resource conservation Psychological: Concern over system burden |
| 8:151 | Informational support without actionable guidance was seen as unhelpful. | Psychological: Frustration from information overload Psychological: Guidance need for action |
| 8:152 | Interactivity without personalization was viewed as “just information.” | Social: Expectation of proactive support Technological: Personalization interaction requirement |
| 8:153 | Digital records reduce the burden of carrying paper documents and improve coordination. | Social: Improved care coordination Technological: Simplification of record management |
| 8:154 | Reducing paper use was seen as a major motivator. | Environmental: Paper reduction benefit |
| 8:155 | Smartphones, internet use, and COVID lockdown experience made digitization easier to adopt. | Psychological: Digital readiness facilitator Psychological: Lifestyle integration |
