# Supplementary material for: Advancing foundational models in digital health technology adoption: A systematic literature review of multidisciplinary factors
Source: PLOS Digit Health. 2026 Sep 2;5(9):e0001690. doi: 10.1371/journal.pdig.0001690 (PMC13537594; doi:10.1371/journal.pdig.0001690)
Supplement: Supplementary file 8 [file pdig.0001690.s006.docx]

**S4. Table. Overview of methodological appraisal of included studies by study design (MMAT)**

| **Study ID** | **Full reference** | **Comments** |
| --- | --- | --- |
| **Qualitative studies** | | |
| [[8]](https://journals.sagepub.com/doi/full/10.1177/00380385231218695) | Alevizou P, Michaelidou N, Daskalopoulou A, Appiah-Campbell R. Self-tracking among young people: lived experiences, tensions and bodily outcomes. Sociology. 2024;58(4):947-64. doi:10.1177/00380385231218695 | All MMAT criteria were met; the study demonstrates high methodological quality by employing an interpretivist life story approach with 23 in-depth interviews and coherent reflexive thematic analysis. |
| [[10]](https://journals.plos.org/digitalhealth/article?id=10.1371/journal.pdig.0000586) | Alkaabi A, Elsori D. Navigating digital frontiers in UAE healthcare: a qualitative exploration of healthcare professionals’ and patients’ experiences with AI and telemedicine. PLOS Digit Health. 2025;4(4):e0000586. doi:10.1371/journal.pdig.0000586 | All MMAT criteria were met; high methodological quality is demonstrated through an appropriate qualitative exploration of AI and telemedicine, supported by semi-structured interviews and coherent thematic analysis. |
| [[20]](https://pmc.ncbi.nlm.nih.gov/articles/PMC10990475/) | Baik D, Reeder B, Coats H, Baker C, Jankowski C. Perceptions and attitudes toward a proposed digital health physical activity program among older family caregivers of persons with heart failure: a qualitative study. Inform Health Soc Care. 2023;48(3):239-51. doi:10.1080/17538157.2023.2227704 | All MMAT criteria were met; the study uses a qualitative descriptive design to assess caregiver perceptions, supported by directed content analysis guided by the UTAUT model. |
| [[21]](https://www.jmir.org/2020/9/e15307/) | Burns K, Nicholas R, Beatson A, Chamorro-Koc M, Blackler A, Gottlieb U. Identifying mobile health engagement stages: interviews and observations for developing brief message content. J Med Internet Res. 2020;22(9):e15307. doi:10.2196/15307 | All MMAT criteria were met; engagement stages are explored through robust deductive-inductive thematic analysis of interviews and observations with inter-rater reliability check. |
| [[24]](https://www.mdpi.com/2076-3425/15/5/464) | Chen ML, Hsieh KL, Hsieh SL, Hsieh MC, Lo DCT, Hsu JL. Barriers to using mobile app-based cognitive testing in older adults with probable Alzheimer’s disease: a qualitative study. Brain Sci. 2025;15(5):464. doi:10.3390/brainsci15050464 | All MMAT criteria were met; barriers to app-based cognitive testing are investigated via semi-structured interviews and thematic analysis validated through investigator triangulation. |
| [[25]](https://journals.sagepub.com/doi/10.1177/20552076231173560) | Cheraghi-Sohi S, Davies K, Gordon L, Jones H, Sanders C, Ong BN. A study to explore the usefulness of a mobile health application to support people with mild cognitive and/or communication impairment due to dementia and their carers. Digit Health. 2023;9:20552076231173560. doi:10.1177/20552076231173560 | All MMAT criteria were met; phased data collection from interviews and focus groups is mapped to the Domestication of Technology Model. |
| [[32]](https://journals.sagepub.com/doi/10.1177/20552076251334043) | Goldberg DG, Soylu TG, Hoffman CF, Kishton RE, Cronholm PF. Clinicians’ perspectives on the adoption and implementation of EMR-integrated clinical decision support tools in primary care. Digit Health. 2025;11:20552076251334043. doi:10.1177/20552076251334043 | All MMAT criteria were met; clinician perceptions are explored through semi-structured interviews informed by theoretical models like UTAUT and DOI. |
| [[34]](https://bmcnurs.biomedcentral.com/articles/10.1186/s12912-025-03108-7) | He Y, Fan G, Fan G, et al. Exploring nurse and patient perspectives on WeChat-based prenatal education in Chinese public hospitals: a qualitative inquiry. BMC Nurs. 2025;24:459. doi:10.1186/s12912-025-03108-7 | All MMAT criteria were met; nurse and patient perspectives are captured through an exploratory design and thematic analysis substantiated by participant quotations. |
| [[36]](https://www.mdpi.com/2227-9032/8/4/494) | Jeng MY, Yeh TM, Pai FY. Analyzing older adults’ perceived values of using smart bracelets by means–end chain. Healthcare (Basel). 2020;8(4):494. doi:10.3390/healthcare8040494 | Most MMAT criteria were met; a Means-End Chain approach is used, though the interpretation is not fully substantiated because the abstract matrices lack participant quotations to verify findings. |
| [[38]](https://journals.lww.com/cinjournal/abstract/2025/05000/feasibility_and_acceptability_of_smartwatches_for.11.aspx) | Johnson AH, Lee K, Reeder B, Popejoy L, Vogelsmeier A. Feasibility and acceptability of smartwatches for use by nursing home residents. CIN Comput Inform Nurs. 2025;43(5):e01245. doi:10.1097/CIN.0000000000001245 | All MMAT criteria were met; smartwatches in nursing homes are assessed through interviews with diverse stakeholders, with findings robustly derived via manual thematic analysis. |
| [[39]](https://journals.lww.com/cinjournal/fulltext/2024/08000/user_needs_and_factors_associated_with_the.6.aspx) | Kahsay DT, Salanterä S, Tommila M, Liukas T, Rosio R, Diji AK, et al. User needs and factors associated with the acceptability of audiovisual feedback devices for chest compression monitoring in cardiopulmonary resuscitation. CIN Comput Inform Nurs. 2024;42(8):583-92. doi:10.1097/CIN.0000000000001126 | All MMAT criteria were met; user needs for CPR devices are explored using semi-structured interviews based on UTAUT constructs and structured matrix analysis. |
| [[40]](https://dl.acm.org/doi/10.1145/3609327) | Karnatak N, Loughrin B, Kuo TA, Mateu-Silvernail O, Medhi Thies I, Thies W, et al. Is it even giving the correct reading or not? How trust and relationships mediate blood pressure management in India. ACM Trans Comput Hum Interact. 2023;30(6):90. doi:10.1145/3609327 | All MMAT criteria were met; blood pressure management is explored through an inductive, iterative analysis process involving multiple coder. |
| [[43]](https://www.frontiersin.org/journals/public-health/articles/10.3389/fpubh.2025.1531173/full) | Kim B, Jeong CH, Blood E, Arthanat S, Corvini M, Golomski C, et al. Multilevel factors influencing eHealth adoption among older adults during the pandemic. Front Public Health. 2025;13:1531173. doi:10.3389/fpubh.2025.1531173 | All MMAT criteria were met; eHealth adoption factors are explored using theoretical frameworks, with findings robustly derived from remote interviews and constant comparison analysis. |
| [[47]](https://journals.plos.org/plosone/article?id=10.1371/journal.pone.0303416) | Kiruthu-Kamamia C, Berner-Rodoreda A, O’Bryan G, Sande O, Huwa J, Thawani A, et al. Acceptability and feasibility of a mobile electronic medical record system for community-based antiretroviral therapy in Lilongwe, Malawi: a rapid qualitative analysis. PLoS One. 2025;20(5):e0303416. doi:10.1371/journal.pone.0303416 | All MMAT criteria were met; Rapid Qualitative Analysis is applied to semi-structured interviews to explore the acceptability of the CARES prototype. |
| [[51]](https://bmcdigitalhealth.biomedcentral.com/articles/10.1186/s44247-024-00127-3) | Leenen JPL, Jordens YJ, Wegman A, et al. Experiences of nurses working in novel virtual care centres in the Netherlands: a qualitative study. BMC Digital Health. 2024;2:70. doi:10.1186/s44247-024-00127-3 | All MMAT criteria were met; nurses’ perceptions are explored via interviews guided by COM-B and UTAUT models, analysed through Braun & Clarke’s six-stage thematic analysis. |
| [[52]](https://www.nature.com/articles/s41746-023-00924-6) | Lee VV, Vijayakumar S, Ng WY, et al. Personalization and localization as key expectations of digital health intervention in women pre- to post-pregnancy. npj Digit Med. 2023;6:183. doi:10.1038/s41746-023-00924-6 | All MMAT criteria were met; women's digital health needs are explored through inductive thematic analysis involving multi-step coding and discrepancy resolution. |
| [[53]](https://humanfactors.jmir.org/2024/1/e58641) | Leong QY, Lee VV, Ng WY, Vijayakumar S, Lau NY, Mauritzon I, et al. Older adults’ perspectives and experiences with digital health in Singapore: qualitative study. JMIR Hum Factors. 2024;11:e58641. doi:10.2196/58641 | All MMAT criteria were met; older adults' perspectives are explored through thematic analysis that utilizes verbatim transcription and inductive independent coding. |
| [[55]](https://academic.oup.com/heapol/article/37/8/943/6545194) | Liverani M, Ir P, Perel P, Khan M, Balabanova D, Wiseman V. Assessing the potential of wearable health monitors for health system strengthening in low- and middle-income countries: a prospective study of technology adoption in Cambodia. Health Policy Plan. 2022;37(8):943-51. doi:10.1093/heapol/czac019 | All MMAT criteria were met; the NASSS framework guides the thematic content analysis of in-depth interviews regarding technology adoption in Cambodia. |
| [[56]](https://aging.jmir.org/2024/1/e52292) | Lu SY, Yoon S, Yee WQ, Ngiam NHW, Ng KYY, Low LL. Experiences of a community-based digital intervention among older people living in a low-income neighborhood: qualitative study. JMIR Aging. 2024;7:e52292. doi:10.2196/52292 | All MMAT criteria were met; older people's experiences are explored through "generic qualitative methodology" and constant comparative analysis of in-depth interviews. |
| [[57]](https://www.jmir.org/2024/1/e50457/) | Manning JB, Blandford A, Edbrooke-Childs J. Facilitators of and barriers to teachers’ engagement with consumer technologies for stress management: qualitative study. J Med Internet Res. 2024;26:e50457. doi:10.2196/50457 | All MMAT criteria were met; teachers' technology engagement is investigated through reflexive thematic analysis of multiple data sources. |
| [[59]](https://bmcpsychiatry.biomedcentral.com/articles/10.1186/s12888-021-03426-5) | Melia R, Monahan L, Duggan J, et al. Exploring the experiences of mental health professionals engaged in the adoption of mobile health technology in Irish mental health services. BMC Psychiatry. 2021;21:412. doi:10.1186/s12888-021-03426-5 | All MMAT criteria were met; Appreciative Inquiry and Braun & Clarke’s thematic analysis are utilized to capture mental health professionals' narratives. |
| [[61]](https://www.tandfonline.com/doi/10.1080/03091902.2022.2089249?url_ver=Z39.88-2003&rfr_id=ori:rid:crossref.org&rfr_dat=cr_pub%20%200pubmed) | Moody L, Wood E, Needham A, Booth A, Jimenez-Aranda A, Tindale W. Identifying individual enablers and barriers to the use of digital technology for the self-management of long-term conditions by older adults. J Med Eng Technol. 2022;46(6):448-61. doi:10.1080/03091902.2022.2089249 | Most MMAT criteria were met; while evidence syntheses are combined with interviews, interpretation is not fully substantiated due to a lack of adequate participant quotations. |
| [[63]](https://journals.sagepub.com/doi/10.1177/20552076251321698?url_ver=Z39.88-2003&rfr_id=ori:rid:crossref.org&rfr_dat=cr_pub%20%200pubmed) | Mwaka ES, Bazzeketa D, Mirembe J, Emoru RD, Twimukye A, Kivumbi A. Barriers to and enhancement of the utilization of digital mental health interventions in low-resource settings: perceptions of young people in Uganda. Digital Health. 2025;11:20552076251321698. doi:10.1177/20552076251321698 | All MMAT criteria were met; digital mental health barriers are explored via a descriptive study supported by focus group discussions and iterative coder checking. |
| [[64]](https://journals.sagepub.com/doi/10.1177/20552076211064406?url_ver=Z39.88-2003&rfr_id=ori:rid:crossref.org&rfr_dat=cr_pub%20%200pubmed) | Namatovu HK, Oyana TJ, Sol HG. Barriers to eHealth adoption in routine antenatal care practices: perspectives of expectant mothers in Uganda—a qualitative study using the unified theory of acceptance and use of technology model. Digit Health. 2021;7:20552076211064406. doi:10.1177/20552076211064406 | All MMAT criteria were met; thematic template analysis based on UTAUT constructs is used to explore eHealth adoption barriers among expectant mother. |
| [[65]](https://onlinelibrary.wiley.com/doi/10.1155/2024/5578056) | Nigatu AM, Yilma TM, Gezie LD, Gebrewold Y, Gullslett MK, Mengiste SA, et al. Barriers and facilitators experienced during the implementation of web-based teleradiology system in public hospitals of the Northwest Ethiopia: an interpretive description study. Int J Telemed Appl. 2024;2024:5578056. doi:10.1155/2024/5578056 | All MMAT criteria were met; an interpretive description approach based on the TOE framework is employed, with findings derived through reflexive thematic analysis. |
| [[66]](https://www.mdpi.com/1660-4601/19/20/13714) | O’Connor SR, Treanor C, Ward E, Wickens RA, O’Connell A, Culliford LA, et al. Patient acceptability of home monitoring for neovascular age-related macular degeneration reactivation: a qualitative study. Int J Environ Res Public Health. 2022;19(20):13714. doi:10.3390/ijerph192013714 | All MMAT criteria were met; home monitoring acceptability is investigated through a directed analysis approach utilizing both deductive and inductive coding. |
| [[67]](https://www.mdpi.com/1999-5903/15/6/191) | Patrickson B, Musker M, Thorpe D, van Kasteren Y, Bidargaddi N, Consumer and Carer Advisory Group (CCAG). In-depth co-design of mental health monitoring technologies by people with lived experience. Future Internet. 2023;15(6):191. doi:10.3390/fi15060191 | All MMAT criteria were met; findings are derived from co-design focus groups via grounded qualitative content analysis and meta-ethnographic thematic synthesi. |
| [[68]](https://www.frontiersin.org/journals/digital-health/articles/10.3389/fdgth.2024.1275569/full) | Posselt J, Baumann E, Dierks ML. A qualitative interview study of patients’ attitudes towards and intention to use digital interventions for depressive disorders on prescription. Front Digit Health. 2024;6:1275569. doi:10.3389/fdgth.2024.1275569 | All MMAT criteria were met; patient perspectives on digital interventions are captured through qualitative content analysis using a deductive-inductive coding scheme. |
| [[69]](https://onlinelibrary.wiley.com/doi/10.1111/nhs.12939) | Rochmawati E, Kamilah F, Iskandar AC. Acceptance of e-health technology among older people: a qualitative study. Nurs Health Sci. 2022;24(2):437-46. doi:10.1111/nhs.12939 | All MMAT criteria were met; health technology perceptions are explored utilizing a six-phase thematic analysis managed with NVivo and substantiated by participant quotations. |
| [[70]](https://onlinelibrary.wiley.com/doi/10.1155/2024/2667732) | Saeed H, Scahill S, Kim J, Moyaen R, Natarajan D, Soga A, et al. Pharmacist perceptions and future scope of telepharmacy in New Zealand: a qualitative exploration. Int J Telemed Appl. 2024;2024:2667732. doi:10.1155/2024/2667732 | All MMAT criteria were met; pharmacists’ perceptions are investigated through inductive thematic analysis organized by the Clinical Adoption Framework. |
| [[71]](https://bmcpublichealth.biomedcentral.com/articles/10.1186/s12889-024-20265-7) | Sampene AK, Li C, Wiredu J. Unravelling the shift: exploring consumers’ adoption or resistance of e-pharmacy through behavioural reasoning theory. BMC Public Health. 2024;24:2789. doi:10.1186/s12889-024-20265-7 | All MMAT criteria were met; e-pharmacy adoption is explored through thematic analysis using Atlas.ti, ensuring coherence with Behavioural Reasoning Theory. |
| [[72]](https://mhealth.jmir.org/2024/1/e48345) | Schroeder T, Haug M, Georgiou A, Seaman K, Gewald H. Evidence of how physicians and their patients adopt mHealth apps in Germany: exploratory qualitative study. JMIR Mhealth Uhealth. 2024;12:e48345. doi:10.2196/48345 | All MMAT criteria were met; mHealth app adoption is investigated using interpretative phenomenological analysis (IPA) and an inductive coding framework. |
| [[73]](https://journals.sagepub.com/doi/10.1177/20552076251326160) | Sezgin E, Jackson DI, Kaufman K, Skeens MA, Gerhardt CA, Moscato E. Perceptions about the use of virtual assistants for seeking health information among caregivers of young childhood cancer survivors. Digital Health. 2025;11:20552076251326160. doi:10.1177/20552076251326160 | All MMAT criteria were met; caregiver perceptions of virtual assistants are explored through thematic analysis with inter-coder agreement. |
| [[74]](https://www.researchgate.net/publication/336011768_Exploring_the_barriers_and_facilitators_for_the_use_of_digital_health_technologies_for_the_management_of_COPD_A_qualitative_study_of_clinician_perceptions) | Slevin P, Kessie T, Cullen J, Butler MW, Donnelly SC, Caulfield B. Exploring the barriers and facilitators for the use of digital health technologies for the management of COPD: a qualitative study of clinician perceptions. QJM. 2020;113(3):163-72. doi:10.1093/qjmed/hcz241 | All MMAT criteria were met; clinician perceptions are explored via an inductive, cyclical thematic analysis using NVivo 12 with saturation. |
| [[75]](https://formative.jmir.org/2022/1/e28895/) | Tennant R, Allana S, Mercer K, Burns CM. Exploring the experiences of family caregivers of children with special health care needs to inform the design of digital health systems: formative qualitative study. JMIR Form Res. 2022;6(1):e28895. doi:10.2196/28895 | All MMAT criteria were met; family caregivers are studied utilizing inductive thematic analysis involving triangulation and data saturation. |
| [[76]](https://nursing.jmir.org/2023/1/e39815) | Vercell A, Taylor S, Yorke J, Dowding D. Assessing the acceptability of home blood monitoring for patients with cancer who are receiving systemic anticancer therapy from a patient, caregiver, and clinician perspective: focus group and interview study. JMIR Nurs. 2023;6:e39815. doi:10.2196/39815 | All MMAT criteria were met; multi-stakeholder perspectives are analyzed through a framework analysis guided by the UTAUT model. |
| [[77]](https://aisel.aisnet.org/icis2024/ishealthcare/ishealthcare/18/?utm_source=aisel.aisnet.org%2Ficis2024%2Fishealthcare%2Fishealthcare%2F18&utm_medium=PDF&utm_campaign=PDFCoverPages) | Weeratunge SK, Bain C, Burstein F, Evans J. Can patient portals support digital health equity for culturally and linguistically diverse communities in Victoria: an exploratory study. In: ICIS 2024 Proceedings. 2024. Available from: https://aisel.aisnet.org/icis2024/ishealthcare/ishealthcare/18 | All MMAT criteria were met; the Digital Health Equity Framework is used to frame both the data collection and the thematic analysis of healthcare interactions. |
| [[78]](https://humanfactors.jmir.org/2024/1/e51972) | Zainal H, Hui XX, Thumboo J, Fong W, Yong FK. Patients’ expectations of doctors’ clinical competencies in the digital health care era: qualitative semistructured interview study among patients. JMIR Hum Factors. 2024;11:e51972. doi:10.2196/51972 | All MMAT criteria were met; patient expectations are examined through inductive thematic analysis following Braun and Clarke’s process. |
| [[80]](https://www.emerald.com/tqm/article-abstract/34/6/1772/377388/Toward-digital-transformation-in-healthcare-a?redirectedFrom=fulltext) | Binci D, Palozzi G, Scafarto F. Toward digital transformation in healthcare: a framework for remote monitoring adoption. TQMJ. 2022;34(6):1772-99. doi:10.1108/TQM-04-2021-0109 | All MMAT criteria were met; a grounded approach with constant comparison methods is employed to explore variables related to remote monitoring adoption. |
| [[81]](https://www.emerald.com/jstp/article-abstract/32/2/105/254615/I-m-no-expert-but-Consumer-use-of-supportive?redirectedFrom=fulltext) | Bocking H, Russell-Bennett R, Letheren K. I’m no expert, but … ? Consumer use of supportive digital tools in health services. J Serv Theory Pract. 2022;32(2):105-31. doi:10.1108/JSTP-09-2020-0225 | All MMAT criteria were met; consumer perceptions are examined through a hybrid thematic analysis utilizing both deductive and inductive codes based on social support theory. |
| [[82]](https://aisel.aisnet.org/cgi/viewcontent.cgi?article=1102&context=pacis2024) | Mukherjee S, Das S. Willingness to digitalize health records: an Indian perspective. In: PACIS 2024 Proceedings. 2024. Available from: https://aisel.aisnet.org/pacis2024/track11_healthit/track11_healthit/8 | All MMAT criteria were met; enablers of digitalising health records are explored through manual and NVivo coding until data saturation was reached. |
| **Quantitative non-randomized** | | |
| [[46]](https://pmc.ncbi.nlm.nih.gov/articles/PMC9790857/) | Kim YK, Ang S. Older adults with functional limitations and their use of telehealth during COVID-19. Res Aging. 2023;45(9-10):609-19. doi:10.1177/01640275221147642 | All MMAT criteria were met; the study utilizes the nationally representative National Health and Aging Trends Study with applied survey weights to ensure representativeness, with minimal missing outcome data and adequate control for confounders. |
| [[60]](https://www.frontiersin.org/journals/public-health/articles/10.3389/fpubh.2025.1542974/full) | Meraya AM, Khardali A, Ahmad S, Hoshaibari AZ, Alameer AA, Jaafari RY, et al. Telehealth perceptions and associated factors among older adults with chronic conditions in Saudi Arabia: a comparative study of users and non-users. Front Public Health. 2025;13:1542974. doi:10.3389/fpubh.2025.1542974 | Few MMAT criteria were met; purposive and snowball sampling compromised representativeness, and confounders were not adequately accounted for. Additionally, the exposure definition lacked necessary detail and outcome data completeness was unclear. |
| **Quantitative descriptive** | | |
| [[2]](https://mhealth.jmir.org/2021/1/e23155/) | Yang S, Chen Y, Zhou L, Huang Y, Dai J. Willingness to adopt mHealth among Chinese parents during the COVID-19 outbreak: cross-sectional questionnaire study. JMIR Mhealth Uhealth. 2021;9(1):e23155. doi:10.2196/23155 | Few MMAT criteria were met; the social media sampling strategy is unclear, making representativeness impossible to determine, and the risk of nonresponse bias cannot be assessed as non-respondents were not reported. |
| [[3]](https://www.jmir.org/2021/6/e28944) | Saigí-Rubió F, Vidal-Alaball J, Torrent-Sellens J, Jiménez-Zarco A, López Segui F, Carrasco Hernandez M, et al. Determinants of Catalan public primary care professionals’ intention to use digital clinical consultations (eConsulta) in the post–COVID-19 context: mixed methods study. J Med Internet Res. 2021;23(6):e28944. doi:10.2196/28944 | Some MMAT criteria were met; while a census approach was used, the low response rate casts doubt on the sample's representativeness, and the risk of nonresponse bias cannot be determined. |
| [[4]](https://www.frontiersin.org/journals/medicine/articles/10.3389/fmed.2024.1421559/full) | Abebe SA, Endehabtu BF, Walle AD, Hailu DT, Yeshaneh AT, Dres EA, et al. Intention to use personal health record system and its predictors among chronic patients enrolled at public hospitals in Bahir Dar city, northwest Ethiopia: using modified UTAUT2 model. Front Med (Lausanne). 2024;11:1421559. doi:10.3389/fmed.2024.1421559 | All MMAT criteria were met; a representative stratified sampling strategy was used, and the risk of nonresponse bias is low due to a high response rate (98.27%). |
| [[5]](https://www.frontiersin.org/journals/health-services/articles/10.3389/frhs.2025.1460077/full) | Alameraw TA, Asemahagn MA, Gashu KD, Walle AD, Kelkay JM, Mitiku AB, et al. Intention to use telemonitoring for chronic illness management and its associated factors among nurses and physicians at public hospitals in Bahir Dar, northwest Ethiopia: using a modified UTAUT2 model. Front Health Serv. 2025;5:1460077. doi:10.3389/frhs.2025.1460077 | All MMAT criteria were met; simple random sampling ensured representativeness, and the risk of nonresponse bias is low due to a very high response rate (93.7%). |
| [[6]](https://www.mdpi.com/2078-2489/13/11/509) | Alanazi F, Gay V, Alturki R. Poor compliance of diabetic patients with AI-enabled e-health self-care management in Saudi Arabia. Information. 2022;13(11):509. doi:10.3390/info13110509 | Few MMAT criteria were met; the sampling strategy is unclear, making representativeness impossible to determine, and the risk of nonresponse bias cannot be assessed due to missing rate data. |
| [[7]](https://www.frontiersin.org/journals/public-health/articles/10.3389/fpubh.2024.1385713/full) | Aldekhyyel RN, Alshuaibi F, Alsaaid O, Bin Moammar F, Alanazy T, Namshah A, et al. Exploring behavioral intention to use telemedicine services post COVID-19: a cross-sectional study in Saudi Arabia. Front Public Health. 2024;12:1385713. doi:10.3389/fpubh.2024.1385713 | Few MMAT criteria were met; reliance on convenience sampling resulted in a non-representative sample, and the risk of nonresponse bias cannot be determined as no response rate was reported. |
| [[9]](https://etasr.com/index.php/ETASR/article/view/8747) | Aljohani N. Digital health transformation in Saudi Arabia: examining the impact of health information seeking on m-health adoption during the COVID-19 pandemic. Eng Technol Appl Sci Res. 2025;15(1):19933-40. doi: 10.48084/etasr.8747 | Few MMAT criteria were met; social media sampling resulted in a non-representative sample skewed towards younger users, and the risk of nonresponse bias cannot be determined. |
| [[11]](https://www.iieta.org/journals/ijsdp/paper/10.18280/ijsdp.200309) | Al-Shami SA, Kusmantini T, Purwandari S, Sharif MS, Doheir M, Rashid N, et al. System service quality factors and their impact on user satisfaction and continuance intention to use m-health: the moderating influence of monetary cost. Int J Sustain Dev Plan. 2025;20(3):1015-27. doi:10.18280/ijsdp.200309 | Some MMAT criteria were met; systematic random sampling was used, but the sample was restricted to a single healthcare setting, and the risk of nonresponse bias cannot be determined. |
| [[12]](https://www.mdpi.com/1660-4601/19/7/4289) | Alsharif AH. Attitudes of patients with chronic diseases toward management eHealth applications systems in post-COVID-19 times. Int J Environ Res Public Health. 2022;19(7):4289. doi:10.3390/ijerph19074289 | Few MMAT criteria were met; social media sampling resulted in a non-representative sample, and the risk of nonresponse bias is high given the significant number of incomplete responses. |
| [[13]](https://peerj.com/articles/cs-2773/) | Al-Somali SA. Integrating artificial intelligence (AI) in healthcare: advancing older adults’ health management in Saudi Arabia through AI-powered chatbots. PeerJ Comput Sci. 2025;11:e2773. doi:10.7717/peerj-cs.2773 | Some MMAT criteria were met; convenience sampling via WhatsApp resulted in a non-representative sample biased toward digitally literate users, and the risk of nonresponse bias cannot be determined. |
| [[14]](https://journals.sagepub.com/doi/10.1177/20552076251336271) | Alsyouf A, Al-Momani AM, Alsubahi N, et al. Predicting digital contact tracing tool adoption during COVID-19 from the perspective of TAM: the role of trust, fear, privacy, anxiety, and social media. Digit Health. 2025;11:20552076251336271. doi:10.1177/20552076251336271 | Few MMAT criteria were met; social media convenience sampling led to a non-representative sample skewed towards younger individuals, and the risk of nonresponse bias cannot be determined. |
| [[15]](https://e-journal.unair.ac.id/JISEBI/article/view/41371) | Alviani R, Purwandari B, Eitiveni I, Purwaningsih M. Factors affecting adoption of telemedicine for virtual healthcare services in Indonesia. J Inf Syst Eng Bus Intell. 2023;9(1):47-69. doi:10.20473/jisebi.9.1.47-69 | Few MMAT criteria were met; convenience sampling resulted in a non-representative sample with selection bias, and the risk of nonresponse bias cannot be determined. |
| [[17]](https://diabetes.jmir.org/2024/1/e56756) | Arueyingho O, Aprioku JS, Marshall P, O’Kane AA. Insights into sociodemographic influences on type 2 diabetes care and opportunities for digital health promotion in Port Harcourt, Nigeria: quantitative study. JMIR Diabetes. 2024;9:e56756. doi:10.2196/56756 | Few MMAT criteria were met; snowball and convenience sampling resulted in a non-representative sample skewed towards high-income groups, and the risk of nonresponse bias cannot be determined. |
| [[22]](https://journals.plos.org/plosone/article?id=10.1371/journal.pone.0303302) | Carrera A, Lettieri E, Lietti G, Martignoni S, Sgarbossa C, Cafazzo J. Therapies go digital. What drives physicians’ acceptance? PLoS One. 2024;19(5):e0303302. doi:10.1371/journal.pone.0303302 | Few MMAT criteria were met; the voluntary web-based survey method makes it impossible to determine if the sample is representative, and the risk of nonresponse bias cannot be determined. |
| [[23]](https://www.frontiersin.org/journals/public-health/articles/10.3389/fpubh.2024.1449594/full) | Wu C, Lim GG. Investigating older adults users’ willingness to adopt wearable devices by integrating the technology acceptance model (UTAUT2) and the Technology Readiness Index theory. Front Public Health. 2024;12:1449594. doi:10.3389/fpubh.2024.1449594 | Few MMAT criteria were met; snowball and convenience sampling resulted in a non-representative sample due to selection bias, and the risk of nonresponse bias cannot be determined. |
| [[26]](https://journals.sagepub.com/doi/10.1177/21582440241228639) | Lu C-C, Tsai-Lin T-F. Are older adults special in adopting public eHealth service initiatives? The modified model of UTAUT. SAGE Open. 2024;14(1). doi:10.1177/21582440241228639 | Few MMAT criteria were met; the sampling method resulted in a non-representative sample with a severe gender skew (80.6% female), and the risk of nonresponse bias cannot be determined. |
| [[27]](https://journals.sagepub.com/doi/10.1177/11795972241277292) | Das Gupta A. Conceptualizing patient as an organization with the adoption of digital health. Biomed Eng Comput Biol. 2024;15:11795972241277292. doi:10.1177/11795972241277292 | Few MMAT criteria were met; the lack of specific recruitment descriptions makes it impossible to determine sample representativeness, and the risk of nonresponse bias cannot be assessed. |
| [[28]](https://www.researchgate.net/publication/338187344_Consumer_adoption_of_smartphone_fitness_apps_an_extended_UTAUT2_perspective) | Dhiman N, Arora N, Dogra N, Gupta A. Consumer adoption of smartphone fitness apps: an extended UTAUT2 perspective. J Indian Bus Res. 2020;12(3):363-88. doi:10.1108/JIBR-05-2018-0158 | Some MMAT criteria were met; while the risk of nonresponse bias was low based on wave analysis, purposive sampling of gym members resulted in a non-representative sample. |
| [[29]](https://pharmacia.pensoft.net/article/108957/) | Dimitrova M, Dimitrov R, Ahchiyska K, Nikolaeva M, Ganova M, Petrova G. Digital health in Bulgaria: imagination or possible reality? Pharmacia. 2023;70(3):649-55. doi:10.3897/pharmacia.70.e108957 | Few MMAT criteria were met; significant reporting gaps regarding the target population prevent assessment of representativeness, and the risk of nonresponse bias cannot be determined. |
| [[30]](https://www.frontiersin.org/journals/public-health/articles/10.3389/fpubh.2023.1325031/full) | Liu D, Son S, Cao J. The determinants of public acceptance of telemedicine apps: an innovation diffusion perspective. Front Public Health. 2023;11:1325031. doi:10.3389/fpubh.2023.1325031 | Few MMAT criteria were met; intercept sampling failed to capture the older adult demographic, and the risk of nonresponse bias cannot be fully determined due to missing total rate data. |
| [[31]](https://www.jmir.org/2021/2/e24767/) | Elkefi S, Yu Z, Asan O. Online medical record nonuse among patients: data analysis study of the 2019 Health Information National Trends Survey. J Med Internet Res. 2021;23(2):e24767. doi:10.2196/24767 | Most MMAT criteria were met; a nationally representative survey (HINTS) was utilised with appropriate weighting, though the low response rate (30.20%) raises potential bias concerns. |
| [[33]](https://journals.healio.com/doi/10.3928/00220124-20241107-03?url_ver=Z39.88-2003&rfr_id=ori:rid:crossref.org&rfr_dat=cr_pub%20%200pubmed) | Han J, Kim M, Ryu KH, Shin K. Acceptance of digital health care technology and the role of nursing education. J Contin Educ Nurs. 2024;55(12):575-87. doi:10.3928/00220124-20241107-03 | Few MMAT criteria were met; convenience sampling from a single cancer specialty hospital resulted in a non-representative sample, and the risk of nonresponse bias cannot be determined. |
| [[37]](https://journals.sagepub.com/doi/10.1177/20552076221126034?url_ver=Z39.88-2003&rfr_id=ori:rid:crossref.org&rfr_dat=cr_pub%20%200pubmed) | Yang J, Luo B, Zhao C, Zhang H. Artificial intelligence healthcare service resources adoption by medical institutions based on TOE framework. Digit Health. 2022;8:20552076221126034. doi:10.1177/20552076221126034 | Most MMAT criteria were met; the risk of nonresponse bias was low (100% response rate), though the population frame was not explicitly defined to confirm representativeness. |
| [[44]](https://www.liebertpub.com/doi/10.1089/tmj.2021.0418?url_ver=Z39.88-2003&rfr_id=ori%3Arid%3Acrossref.org&rfr_dat=cr_pub++0pubmed) | Kim HM, Xu Y, Wang Y. Overcoming the mental health stigma through m-health apps: results from the Healthy Minds Study. Telemed J E Health. 2022;28(10):1534-40. doi:10.1089/tmj.2021.0418 | Few MMAT criteria were met; the authors acknowledge the sample does not reflect the target population, and response rates were not reported to assess nonresponse bias. |
| [[45]](https://www.jmir.org/2023/1/e41915) | Kim S, Chow BC, Park S, Liu H. The usage of digital health technology among older adults in Hong Kong and the role of technology readiness and eHealth literacy: path analysis. J Med Internet Res. 2023;25:e41915. doi:10.2196/41915 | Few MMAT criteria were met; web-based convenience sampling systematically excluded non-internet users, and the risk of nonresponse bias cannot be determined. |
| [[49]](https://www.frontiersin.org/journals/public-health/articles/10.3389/fpubh.2025.1547189/full) | Larnyo E, Tettegah S, Nutakor JA, Addai-Dansoh S, Arboh F. Technology access, use, socioeconomic status, and healthcare disparities among African Americans in the US. Front Public Health. 2025;13:1547189. doi:10.3389/fpubh.2025.1547189 | Most MMAT criteria were met; the stratified HINTS sampling frame ensured generalisation to the target population, though the risk of nonresponse bias cannot be determined for the specific subsample. |
| [[58]](https://www.mdpi.com/2227-9032/13/11/1222) | Marinescu ȘA, Oncioiu I, Ghibanu A-I. The digital transformation of healthcare through intelligent technologies: a path dependence-augmented–unified theory of acceptance and use of technology model for clinical decision support systems. Healthcare (Basel). 2025;13(11):1222. doi:10.3390/healthcare13111222 | Some MMAT criteria were met; the risk of nonresponse bias was low (81.5% response rate), though contradictory claims about sampling methods make representativeness difficult to determine. |
| [[79]](https://www.liebertpub.com/doi/10.1089/tmj.2022.0527?url_ver=Z39.88-2003&rfr_id=ori%3Arid%3Acrossref.org&rfr_dat=cr_pub++0pubmed) | Almathami HKY, Win KT, Vlahu-Gjorgievska E. Empirical evidence of internal and external factors influencing users’ motivation toward teleconsultation use. Telemed J E Health. 2024;30(1):141-56. doi:10.1089/tmj.2022.0527 | No MMAT criteria were met; significant reporting deficiencies prevent the assessment of sampling, analysis, or the risk of nonresponse bias. |
| **Mixed methods** | | |
| [[1]](https://formative.jmir.org/2021/2/e19074/) | Freise L, Neves AL, Flott K, Harrison P, Kelly J, Darzi A, et al. Assessment of patients’ ability to review electronic health record information to identify potential errors: cross-sectional web-based survey. JMIR Form Res. 2021;5(2):e19074. doi:10.2196/19074 | Few MMAT criteria were met; while the qualitative component was high quality, the quantitative component suffered from a low response rate (23.5%) preventing assessment of nonresponse bias, and evidence of effective data integration is lacking. |
| [[16]](https://bmchealthservres.biomedcentral.com/articles/10.1186/s12913-023-10368-z) | Anttila M, Koivisto J, Luoma ML, et al. How to adopt technologies in home care: a mixed methods study on user experiences and change of home care in Finland. BMC Health Serv Res. 2023;23:1342. doi:10.1186/s12913-023-10368-z | Few MMAT criteria were met; while integration was effective, the quantitative component relied on convenience sampling and non-pilot-tested measurements, and the risk of nonresponse bias cannot be assessed as rates were not reported. |
| [[18]](https://sigmapubs.onlinelibrary.wiley.com/doi/10.1111/jnu.12619) | Aure CF, Kluge A, Moen A. Older adults’ engagement in technology-mediated self-monitoring of diet: a mixed-method study. J Nurs Scholarsh. 2021;53(1):25-34. doi:10.1111/jnu.12619 | Some MMAT criteria were met; integration was effective, but the quantitative component was limited by convenience sampling and a small sample size, and the risk of nonresponse bias was high due to significant attrition (36%). |
| [[19]](https://onlinelibrary.wiley.com/doi/10.1111/jep.13923) | Bagot KL, Bladin CF, Vu M, Bernard S, Smith K, Hocking G, et al. Factors influencing the successful implementation of a novel digital health application to streamline multidisciplinary communication across multiple organisations for emergency care. J Eval Clin Pract. 2024;30(2):184-98. doi:10.1111/jep.13923 | Some MMAT criteria were met; integration was effective, but the quantitative component demonstrated reporting deficiencies regarding the total eligible population, preventing assessment of representativeness and nonresponse bias. |
| [[35]](https://dl.acm.org/doi/10.1145/3635472) | Jardine J, Nadal C, Robinson S, Enrique A, Hanratty M, Doherty G. Between rhetoric and reality: real-world barriers to uptake and early engagement in digital mental health interventions. ACM Trans Comput Hum Interact. 2024;31(2):27. doi:10.1145/3635472 | Few MMAT criteria were met; while integration was effective, the quantitative component used inappropriate measures and resulted in a skewed sample, with a high risk of nonresponse bias due to self-selection. |
| [[41]](https://journals.sagepub.com/doi/10.1177/20552076221150745) | Keogh A, Alcock L, Brown P, Buckley E, Brozgol M, Gazit E, et al. Acceptability of wearable devices for measuring mobility remotely: observations from the Mobilise-D technical validation study. Digit Health. 2023;9:20552076221150745. doi:10.1177/20552076221150745 | All MMAT criteria were met; the quantitative component achieved a 95.5% response rate with validated measures, ensuring the sample was representative, and was effectively integrated with coherent qualitative findings. |
| [[42]](https://www.jmir.org/2023/1/e44131) | Kerr JI, Naegelin M, Benk M, von Wangenheim F, Meins E, Viganò E, et al. Investigating employees’ concerns and wishes regarding digital stress management interventions with value sensitive design: mixed methods study. J Med Internet Res. 2023;25:e44131. doi:10.2196/44131 | Few MMAT criteria were met; while integration was effective, admitted self-selection bias affected the quantitative sample, self-developed scales required validation, and nonresponse bias cannot be determined. |
| [[48]](https://journals.sagepub.com/doi/10.1177/11786329241284401?url_ver=Z39.88-2003&rfr_id=ori:rid:crossref.org&rfr_dat=cr_pub%20%200pubmed) | Kitole FA, Shukla S. Cloud horizons: strengthening rural healthcare through telemedicine’s digital canopy. Health Serv Insights. 2024;17:11786329241284401. doi:10.1177/11786329241284401 | Some MMAT criteria were met; triangulation was effective, though the quantitative component relied on purposive sampling that was not representative, and measures were self-reported without validation. |
| [[50]](https://games.jmir.org/2024/1/e47600) | Lee EWJ, Tan WW, Pham BTP, Kawaja A, Theng YL. Addressing data absenteeism and technology chauvinism in the use of gamified wearable gloves among older adults: moderated usability study. JMIR Serious Games. 2024;12:e47600. doi:10.2196/47600 | Some MMAT criteria were met; integration was effective, but the quantitative component was limited by a very small sample size (N=19) and convenience sampling, and the risk of nonresponse bias remains unassessed. |
| [[54]](https://www.mdpi.com/2227-9067/11/10/1215) | Linder L, Utendorfer H, Oliveros B, Gilliland S, Tiase VL, Altizer R. Usability evaluation of the revised Color Me Healthy symptom assessment app: perspectives of children and parents. Children (Basel). 2024;11(10):1215. doi:10.3390/children11101215 | Some MMAT criteria were met; integration was effective, though the quantitative sample was small (N=14) and lacked diversity, and the risk of nonresponse bias remains uncertain as eligible families were not reported. |
| [[62]](https://journals.sagepub.com/doi/10.1177/20552076241247196?url_ver=Z39.88-2003&rfr_id=ori:rid:crossref.org&rfr_dat=cr_pub%20%200pubmed) | Moody L, Wood E, Needham A, Booth A, Tindale W. Exploring how the design and provision of digital self-management technology can improve the uptake by older adults with chronic kidney disease, diabetes and dementia: a modified e-Delphi study. Digital Health. 2024;10:20552076241247196. doi:10.1177/20552076241247196 | Some MMAT criteria were met; sequential integration was effective, but the quantitative component was limited by a small, non-diverse expert panel, and the risk of nonresponse bias remains uncertain due to unanalysed attrition. |
