# Supplementary material for: Advancing foundational models in digital health technology adoption: A systematic literature review of multidisciplinary factors
Source: PLOS Digit Health. 2026 Sep 2;5(9):e0001690. doi: 10.1371/journal.pdig.0001690 (PMC13537594; doi:10.1371/journal.pdig.0001690)
Supplement: Supplementary file 9 [file pdig.0001690.s007.docx]

**S1 Appendix. Thematic analysis matrix for digital health technology adoption factors**

| **Main RQ:** What technological, psychological, social, cultural, health-related, and environmental factors influencing the adoption of digital health technologies reflected in existing technology adoption models?  **Aim of research:** To thematically synthesise and critically examine multidisciplinary factors influencing digital health technology adoption, and to identify conceptual gaps and limitations within existing technology adoption models.  **Research inquiry:** Systematic literature review with thematic analysis using a hybrid deductive-inductive coding strategy.  **Informants:** The population consists of primary empirical studies examining digital health technology adoption among patients, healthcare professionals, the general public, older adults, and mixed stakeholder groups across diverse health settings. | | | | | |
| --- | --- | --- | --- | --- | --- |
| **Research Questions** | **Deductive codes / Theories** | **Strategy of inquiry** | **Inductive codes** | | |
|  |  |  | **Coding** | **Category** | **Final theme** |
| **Sub RQ 1:** What technological, psychological, social, cultural, health-related, and environmental factors influence the adoption of digital health technologies? | - Technological factors - Psychological factors - Social factors - Cultural factors - Health-related factors - Environmental factors - The six domains guided the initial coding process rather than serving as fixed deductive coding frameworks. - Findings that did not align with these domains were coded inductively and incorporated into subsequent categories and themes. - Organisational readiness, workforce capacity, governance, affordability and equity emerged inductively as cross-cutting concepts during coding. - These concepts were synthesised within the final themes and were not treated as additional analytical domains. | - Systematic literature review conducted in accordance with PRISMA 2020 guidelines - Protocol registered in PROSPERO with transparently documented amendments Eligibility guided by the SPIDER framework (mapped to PICOS for screening) - Structured thematic analysis informed by Braun and Clarke's principles, operationalised using the Thematic Analysis Matrix and managed in ATLAS.ti | **Technological factors:**   - HCP-patient communication tool - Real-time linkage with providers - Demonstrability of results - Immediate output visibility - Visual analytics for stress awareness - App slowness - Backup system needs - Data storage capacity limitations - Device malfunction - DHT functionality gaps - DHT resilience - Limited system responsiveness - Server downtime - System quality - System reset disruption - Trust in system reliability - Confidential self-management affordances - Cybersecurity concern - Encrypted synchronization across DHTs - Trust in data security - Uncertainty of data transmission - Dashboard-based data visualization - Ease of health information reporting - Graph and history features aiding symptom visualization and interpretation - Provision of health content library - Provision of more health information - Quantity of health information - Search history need - Bias in AI algorithms - Complexity of AI systems - Availability of DHT regulations - Standardization of health information for HCP - Accuracy of patient information within system - Administrative information errors - Completeness of patient information within system - Data errors - Data quality - Device calibration issue - Difficulty in step tracking - Health information repository - Patient-generated health data - HCP efficient information retrieval - HCP workflow change - HCP workflow integration - HCP workflow integration requirement - HCP-enabled health alerts prediction - Bluetooth and phone proximity constraints - Cross-feature integration - Cross-system interoperability - Device syncing difficulties - Device–system connectivity - Difficulty in sharing information via eHealth systems - Interoperability across devices - Interoperability across settings - Interoperability between service - Lack of unified health database - Limitations in information sharing - Need for horizontal integration - Open API requirement for local customization - Platform integration gap - Poor system integration - Seamless device-app data synchronization - Seamless integration with existing devices and apps - Standardized reporting - Technology integration need - Tool–system integration - Within-system interoperability - Adequacy of technical resources - Availability of onsite technical expertise - Availability of technical resources - Lack of DHT readiness for NCD data integration - DHT workflow efficiency - Duplication of effort in DHT use - Expectations of developers - HCP work duplication - Reduction of double documentation - Reduction of HCP work duplication - Simplification of record management - Workflow fit - HCP ease of use - Availability of DHT system in hospitals - Digital prescription management - Health system compatibility - Accessibility of DHT - Availability and compatibility of technical devices - Availability of DHT - Availability of DHT infrastructure - Availability of digital infrastructure - Convenience of quick answers - Portal availability and utilization - Technical connectivity - Adaptive tracking linked to maternal milestones - Automatic DHT functions - Avatar customization - Device behavioural nudges - DHT functions tailored to individuals needs and preferences - Drawing and customization features - Feedback complexity - Feedback quality from DHT use - Gameplay difficulty balance need - Gamification - Gamification elements - Gamification requests - Gamified personalization - Gamified rewards - In-app nudges for reflection and self-care - Need for customizable design - Need for data-driven personalized interactions - Personalization interaction requirement - Personalization tailoring need - Personalized responses - Provision of personalized content - Provision of personalized health information - Real-time information feedback - Requests for game badges - Requests for game rewards - Tailored to user complex needs - Absence of DHT learning resources and assistance - Multimedia aids for comprehension - Multimodal user guidance - Requests for animations - Adaptability design - Adaptation to DHT over time - App usability limitations - Apps complexity - Authentication and access difficulties - Barriers in account setup and management - DHT aesthetics - DHT usability issues - Difficulty in display readability - Ease of adjustment for usability - Emoji-based responses - Font size - Image clarity and quality - Interactive user experience features - Interface attractiveness - Intuitive interface design - Learnability of DHT - Learnability of wearable devices - Minimal interaction requirements in DHT use - Navigation complexity - Preference for intuitive DHT - Preference for simple DHT - Screen visibility and readability - System adaptability to evolving needs - Technological design - Usability and setup difficulties - Usability barrier from poor design - Usability of DHT - User ease of operation - User ease of use - User technical usability issues - User-friendly features - User-friendly system interface - Visual usability considerations - Age-friendly design - Availability of multimodal user modes - Battery life - Convenience of passive data collection - Device portability for active lifestyle - Device size - Display size limitations - Ergonomic comfort of wearables - Inclusive design - Lightweight device design - Minimal interaction in DHT use - Multi-language options - Multilingual support - Passive activity logging reducing effort - Personalisation for diverse needs - Preference for simple and accessible design - Simple device design - Stigma reduction interface - Supportive design - Unobtrusive device design - Usability for diverse needs - Wearability comfort - Ease of photo tracking - Environmental usability - User activity monitoring - User aid in daily task management - User health data monitoring function - Absence of notifications - Chatbot support features - Reminder facilitation - Supportive reminders   **Psychological factors:**   - Obsessive self-tracking - User value compatibility - Weight management goal setting - Fear of unnecessary messages - Message overload - Notification fatigue - Reminder-based adherence - Reminder-driven motivation - User forgetfulness in DHT use - Age-based confidence gap - Anxiety from health data - Digital comfort - Digital confidence - Fear of technology as a barrier - Health self-efficacy - Patient empowerment through health data management - Patient empowerment through self-care - Patient empowerment through self-monitoring - Patient self-efficacy - Self-management during therapy waiting periods - Technology self-efficacy - User computer self-efficacy - User emotional distress during use - User fatigue in DHT use - User resistance to change - User technology-related anxiety - Data reflection - Desire for longevity and well-being - Development of home care coping skills - Disease coping stage - Enhancement of emotional well-being - Exercise goal setting - Expectation of daily life communication aid - Expectation of daily life memory aid - Goal of quality-of-life enhancement - Health improvement goal - Health self-management goal setting - Healthy lifestyle orientation - Heart rate goal setting - Integration into health routines - Integration of DHT into self-care routines - Internet use duration - Life goal alignment - Lifestyle integration - Maintenance of independence in daily living - Nutrition goal setting - Outcome expectancy - Perceived susceptibility - Perceived value in service delivery - Perception of lifestyle–technology mismatch - Personal fitness improvement goals - Personal interest in nutrition - Priority of usefulness over novelty - Quality-of-life enhancement - Reassessment of changing needs - Relaxation support - Sleep goal setting - Types of priorities in DHT use - Uncertainty about DHT role in health management - User accountability in health behaviours - User health beliefs - User lifestyle fit - User perceived benefits - User perceived health benefits - User perceived lack of need - User performance expectancy - User priorities and needs conflict - User relative advantage - User self-recognition of health behaviours - User effort expectancy - User perceived complexity - Adaptability to diverse needs - Device wearability burden - Effortful typing and device use - Input effort conflicts - User burden of device use - Age-related reluctance and decline in motivation - Altruistic motivation - Coach-driven motivation - Coach-mediated accountability - Commitment to DHT use - Continuity of care motivation - Curiosity satisfaction - Device-driven motivation - External motivation - HCP habitual DHT use - Health condition awareness driven adoption - Increasing health awareness - Interest in DHT - Internal motivation - Intrinsic motivation - Knowledge-seeking behaviour - Openness to new technologies - Overcoming hesitancy due to necessity - Past fitness app use habits - Personal innovativeness - Personal optimism - Preferred use of DHT - Risk propensity - Stress-reduction motivation - User attitude toward DHT - User enjoyment - User future-oriented motivation - User habitual DHT use - User hedonic motivation - User hopefulness for future improvements - User hopefulness for work relevance - User intention to use - User perceived enjoyment - Wellness motivation for self-care - Adaptability to daily life and environments - Awareness of DHT value - Concern about age-related digital divide - Education level - Fear of judgement in DHT use - General literacy constraints - Influence of age - Influence of online health information - Internalized ageism - Pandemic awareness influence - Pandemic-induced anxiety - Post-pandemic intention to use DHT - Professional work history - Reliance on social media for health information - Resistance and concealment behaviours - Stigma avoidance - Perceived cost reducing benefit valuation - User cost-benefit perception - Availability of professional coaching - Coping support during waiting periods - Feedback motivating continued use - Reassurance from tracking features - Social competition motivation - User confirmation of chatbot expectations - User desire for feedback provision - User desire for personalized care - User satisfaction - Awareness of DHT - DHT knowledge gaps - Digital health literacy level - Digital literacy adaptation - Digital literacy level - Enhanced understanding of DHT - Guidance need for action - Health literacy level - Personally relevant leaning content - Prior computer experience - Prior ICT experience - Prior ICT familiarity - Prior internet use - Familiarity through DHT similarity - Familiarity with DHT - Perceived time cost of learning DHT - Prior DHT experience - Uncertainty about ease of use - Distrust in app providers - Distrust in commercial providers - Internet trust limitations - Loss of autonomy from surveillance - Perceived value of data management - Suspicion toward DHT - Trust in DHT - Uncertainty of usefulness - Usefulness of DHT - User autonomy in data sharing - User autonomy in DHT use - User control over engagement - User perceived lack of direct usefulness - User perceived usefulness - User perceived utility - Skepticism about DHT accuracy - Skepticism about DHT measurement accuracy - Concern over system burden - HCP emotional workload strain - HCP perceived benefits - HCP perceived usefulness - HCP performance expectancy - Information-sharing burden - DHT adoption readiness - Digital readiness facilitator - Doubt about DHT sustainability due to failed pilots - Fear of technology replacing service for savings - HCP effort expectancy - HCP familiarity with DHT - HCP knowledge on DHT - HCP level of DHT use - HCP motivation to use - HCP resistance to adoption - HCP resistance to change - HCP self-arranged learning - HCP technical skill level - Perceived complexity by HCP - Prior bedside experience as capability base - Skepticism from prior rehabilitation experience - Support for consistent and continuous use - Barriers from denial of health condition - Caregiving competing priorities - Cognitive issues - Active listening in remote care - Empathy challenges with DHT - Online communication competence - User expectation for fast replies - Outcome observability - Outcome visibility - Cybersecurity risk perception - Fear of data breaches - Fear of data misuse - Fear of hacking - Fear of phishing - Fear of privacy breaches - Fear of surveillance - Preference for anonymity in digital health use - Challenge of result interpretation - Confusion about medical acronyms - Difficulty in test result interpretation - Difficulty with medical jargon - Framing of health information - Frustration from information overload - Graph interpretation difficulties - Health information overload - Misinformation vulnerability - Multi-source information seeking - Perceived reliability of health information - Preference for data presentation - Preference for numeric results - Search fatigue - User health information-seeking behaviour - User perceived relevance - User priority for information access over technical performance - Awareness of AI value - Perceived control and transparency over AI - Requirement for trust in AI service - Skepticism about AI capabilities - Skepticism toward AI - Trust in AI service - Responsibility relevance - Benefit-based privacy decision-making - Conditional data sharing for personalization - Confidence in telemedicine platforms - Consultation choice autonomy   **Social factors:**   - Attention and safety needs in remote consultation - HCP preference for pilot testing - Structured digital office hours - Unsafe school communication - Refresher training need - Structured DHT teaching and awareness - Technical assistance needs - Technical support - User continuous support on DHT use - User DHT education - User DHT guidance - User DHT training - User onboarding training - User pre-usage training - User technical skill development - User training with feedback - Volunteer tutoring as support scaffold - Accessibility for hard-to-reach populations - Co-design with specific groups - User inclusivity - Motivation for team participation - Motivation from private group data exchange - Social motivation through data sharing - Types of habitual HCP use of DHT - Value of social belonging - Daily life communication support - Scheduling support - Parental perceptions for value - Patient influence - Peer connection via app - Peer endorsement - Peer influence - Peer norms - Peer recommendations - Peer storytelling for reassurance and normalization - Peer support - Peer support through safe sharing - Peer validation through data sharing - Priority for instrumental support - Recognition of carer contributions - Reduction of caregiving burden - Reference group influence - Relatives’ motivational help - Relatives' support in navigating systems - Relatives’ technical assistance - Social connection improvement - Social connection support - Social endorsement within communities - Social media influence on body image - Social network support - Social obligations pressure - Support from close acquaintances - Trust in community or family sources - Trust in family - Trust in informal care - Underutilization of social platforms for health - Unrealistic body ideals from influencers - Workplace stigma risk from visibility - Expectation of proactive support - Feedback for service design - Social competition through data sharing - User engagement in DHT design - Availability of help in DHT usage - Continuous user support - Educational support needs - Instructional aids and professional demonstration guidance - One-to-one DHT guidance and troubleshooting - Orientation facilitation - Practical task guidance - Professional guidance via chat or tools - Community support - Community- and hospital-based DHT workshops - Community-based device intermediation - DHT awareness campaigns - Employer responsibility for employee well-being - Preference for support hierarchy - Support services for users - Volunteer support program for DHT service - Access to remote consultation - Advantage of flexible access - Appointment access delays - Availability of administrative support - Availability of health support - Delays in consultations and report submissions - Ageism among HCP - Benefit of stigma reduction - Caregiver isolation - Caregiver training burden in information management - Caregiving workload burden - Colleagues influence - Conflict of family involvement with medical advice - Coworkers encouragement - Demand for carer involvement - DHT awareness through social media - Expectation from supervisors - Family assistance support - Family encouragement - Family influence - Family modeling effect - Family recommendations - Family reminder support - Family responsibilities - Family support - Family technical assistance support - Family-level resistance - Friends encouragement - Household responsibility - Influence of occupation - Influence of peer reviews - Marital status - Negative spousal influence - Observability of electronic word-of-mouth (eWOM) - Parental influence - Trust through recommendations from HCP, family, peers, and institutions - Clinician review workload - HCP efficiency feedback need - Team profile enhancement - Adequacy of organizational support - HCP engagement in DHT design - HCP exclusion from design - HCP-vendor relationships - Availability of HCP support - Continuous HCP learning - Continuous HCP training - Erosion of HCP role boundaries - Expectation of HCP AI competence - Expectation of HCP DHT knowledge - HCP as prescription gatekeeper - HCP attitude toward DHT - HCP autonomy concerns - HCP awareness of DHT - HCP DHT training - HCP DHT training needs - HCP digital skills gap - HCP guidance on anomaly explanation - HCP guidance on result review - HCP guidance requirement for DHT use - HCP guiding skills development - HCP professional role - HCP technical skill development - HCP training for digital communication - Stakeholder DHT training - Trust-building among HCPs - Type of HCP role - Types of HCP DHT use - Care coordination support - Connection to local healthcare services - Data sharing through HCP collaboration - Engagement in research - HCP access to patient health information - HCP bridging role in digital pathways - Improved care coordination - Limited post-prescription role - Reduced pharmacist interaction - Community sensitization on DHT - Communication skills in remote care - Directive counseling in remote care - Empathic rapport in remote care - Failure in HCP-patient communication channel - HCP clear feedback need - HCP concern about reduced empathy in digital communication - HCP content credibility in digital communication - HCP persuasion and empathy skills in remote care - HCP reliable feedback need - HCP-enabled patient communication - HCP-patient collaboration enhancement - HCP-patient collaboration expectation - HCP-patient communication enhancement - HCP-patient interaction quality - HCP–patient onboarding conversations - Improved patient-provider communication - Inconsistent communication practices - Isolation reduction through video call skills - Motivational interviewing in remote care - Patient-HCP relatedness - Reduced rapport in virtual care - Risk of miscommunication - Trust in interpersonal health information - User desire for human support in DHT use - Continuity of remote consultation - HCP–patient relationship enhancement - HCP–patient relationship improvement - Lack of continuity with HCP - Preferred access to doctor - HCP concern over patient data security and privacy - Localized care navigation guidance - HCP concern about DHT algorithmic bias - HCP fear of racial and ethnic bias in DHT use - Shared responsibility in care - User perceived marginalization - User personhood support - Doctor-patient trust - Family access to user data - HCP influence - HCP recommendations - Preference for accessible local providers - Trust erosion due to care gaps - Trust in healthcare professionals   **Cultural factors:**   - Preference for non-clinical design - Language and cultural accessibility - Language barriers - Localized design for context - Translation needs - Culture-specific lifestyle guidance - Preference for efficiency over eye contact - Preference for in-person clinician interpretation - Preference for in-person care - Preference for in-person consultations - Preference for in-person interaction - Preference for in-person reassurance - Preference for in-person visits - Content irrelevance to local context - Provision of localized content - Human touch preference in AI decision-making systems - Preference for hybrid AI–human interaction - Ethnic disparity - Gaps in diversity and inclusion - Preservation of dignity - Racial disparity - Representation to avoid biased DHT development - Systemic racism - Emphasis on voluntary use - Preference for face-to-face care - Transparency for personhood - Organizational culture for stress-related privacy - Organizational innovation culture - Attitude toward resource conservation - Anti-ageism pedagogy - HCP preference for traditional tools - Resistance to change in healthcare - Resistance to digital substitutes - Centrality of in-person care - Access to device through gifting - Cultural acceptance - Cultural fear or dislike preference - Cultural stigma against mental illness - Influence of gender - Pandemic-driven social acceptance - Preference for cultural concordance - Preference for non-digital stress coping strategies - Religious beliefs - Social normalization of DHT - Stigma barrier to DHT use - Stigma from clinical looking devices - Stigma from disability-oriented design - User preference for traditional tools   **Health-related factors:**   - Absence of medical certification - Benefits of infection control - Clinical and implementation evidence - Evidence-based guidance - Fear of infection - HCP validation requirement (evidence-based) - Improvement of diagnostic accuracy in remote care - Lack of clinical evidence - Limited diagnostic capacity in remote care - Medically derived risks - Negative mental health impact of self-tracking - Overuse of self-tracking - Overuse risk of smartphone accessibility for children - Recognition of clinical advantages - Risk of carer over-reliance on DHT - Risk of counterfeit drugs - Risk of misdiagnosis in remote consultations - Risk of misinformation in remote consultations - Risk of over-monitoring - System-generated safety alerts for user - Trust in evidence-based and safe content - Trust in medically certified systems - User perceived safety in DHT - User safety assurance - Clinical benefits - Disease prevention benefits - Health monitoring benefits - Holistic health assessment need - Improvement of healthcare service quality - Real-time health monitoring - Support for symptom recognition and response - User health monitoring support - Value of real-time health metric monitoring - HCP-enabled continuity of care - Long-term therapy experience - Therapeutic continuity between sessions - Assurances of confidentiality - Concern over health data confidentiality - Confidentiality concerns in sensitive apps - Data privacy and security needs - Data security and privacy concerns - Provision of secure and validated information - Appropriateness of health information - Education gap on side effects - Lack of medication information - Quality of health information in DHT system - Request for accurate oncology-specific information - Support for health information recall - Timing of health information provision - Concern about missing data reference values or standards - Nonmaleficence and potential harms - User ethical escalation prompts - Consent and boundary setting - Consent boundary setting - User control over health data - Clinical information completeness - Clinical information errors - Concerns about inconsistent or conflicting DHT results - Data reliability concern - User health data accuracy - HCP decision-support benefits - HCP job performance enhancement - Improvement of HCP patient care - Broad clinical knowledge for remote care triage - Risk of HCP overreliance on DHT - HCP-enabled chronic illness monitoring - HCP-enabled rehabilitation tracking - Real-time care practice monitoring - Rehabilitation support and tracking - Use in population-level NCD risk data collection - Chronic disease management needs - Convenience for sick individuals - Convenience of home testing - Demand for ongoing care monitoring - Disease management needs - Emergency access benefit - Evolving mental health needs - Timeliness of access to care and results - Timeliness of health information for HCP - Timely consultations and monitoring - Comorbidity-related barriers - Disability-related barriers - Family health needs - Fear of pandemic - Functional limitations driven adoption - Maternal phase-specific actionable feedback - Memory decline - Memory issues - Mobility limitations - Outbreak pattern detection - Physical health issues - Pregnancy and child milestone-based guidance - Timely maternal phase-specific updates for engagement - User barriers due to functional limitations - Expert guidance and reassurance - Person-centred digital care - Risk of strap irritation - Screen sensitivity - User burden of continuous wear - Wear-time tolerance limit - Alignment with user health practices - Disease severity - Health management and technology use - Illness-forced lifestyle change - Improvement of home care coping procedures - Individual health condition - Influence of disease stage - Support for safe independent living - Symptom tracking - User health performance evaluation - Support for medication adherence   **Environmental factors:**   - Constraints on feasibility of long-term telemonitoring - Digital communication policy for HCP availability - Expectation of escalation protocols - Need for standardized remote-care protocols - Oversight by certified companies - Professional guidance and standards for DHT - Requirement for safety nets - Monetary incentives - Non-financial incentives for HCP stakeholders - Rapid technology turnover - Sustainability concern - Competing demands of school and workplace - Convenience in busy contexts - User convenience in health tracking - User time conflicts - User time constraints - User work time constraints - Low-threshold entry to care - Mobile phone ownership - Quality of available ICT infrastructure - Reliability of doorstep delivery - Requirement for service expansion - Rural healthcare access - Service expansion opportunity - Smartphone ownership - Uncertainty about DHT availability - User convenience in healthcare access - Caregiver paper record burden - Competitive pressure - Convenience during isolation or quarantine - Digital divide barrier - Geographical disparities - Impacts of COVID-19 on home care services - Pandemic-driven adoption - Pandemic-driven necessity - Rural inequalities - Social precarity - Socioeconomic status - Affordability for DHT - Affordable data barrier - Affordable healthcare access - Availability of financial support - Barrier to accessibility of payment methods - Cost of apps - Cost of equipment or device - Cost savings through discounts - Financial cost barrier - Financial expense of DHT - Financial resources accessibility - Health insurance - Hidden costs of digital care - Internet bundle cost - Internet cost barrier - Preference for free apps - Relief of cost burden for migrants - Uncertainty about technology affordability - Convenience for mobility-limited individuals - Inclusive DHT framework - Weather adaptability - Financial incentives - Improvement of healthcare resource allocation efficiency - Improvement of healthcare service efficiency - Insufficient reimbursement - Lack of insurance reimbursement - Low prescribing incentive - Paper reduction benefit - Remuneration availability - Uncertainty about funding sustainability - Uncertainty of post-implementation sustainability - User cost savings - User money savings - User time savings - HCP malpractice liability concern - HCP workforce shortage - Fragmented healthcare practices - Outdated healthcare systems - Risk of program fragmentation - Availability of pilot programs - Broadband policy and community program - DHT program continuity and practice needs - Financial subsidies for DHT implementation - Government funding support - Government support - International collaboration - National funding allocation - Opportunity for usage testing - Policy and community programs addressing digital disparities - Policymaker DHT training - Subsidy policies and community programs - Targeted financial and infrastructure strategies - Training policies and community programs - Computer ownership - Device availability need - DHT device availability - Electricity gaps - Healthcare access improvement - Improved healthcare service accessibility - Improvement of specialized care access - Internet accessibility - Internet connectivity - Internet connectivity and affordability - Internet-enabled devices - HCP workload response gap - Improvement of HCP work effectiveness - Improvement of HCP work performance - Data transfer standards - Institutional interoperability gap - Capacity gaps in small institutions - Facility resource autonomy - Flexible organizational structures - Implementation planning need - Institutional directives - Institutional implementation maturity - Institutional influence - Institutional support - Lack of leadership support - Leadership endorsement - Organisational readiness to change - Organizational access gap - Organizational approval dependency - Organizational endorsement - Organizational support - Preference for phased rollout strategy - Presence of organizational champions - Shortage of HCPs and technical staff - Skilled workforce shortage - Stakeholder alignment - Testing environment barriers - Weak institutionalization - Administrative load - Benefit of HCP travel reduction - Benefit of price transparency - Budget constraint - Business model limitations in service scope - Convenience through reduced visits and travel - Cost efficiency for facilities and parties - Cost for DHT maintenance - Cost reduction in healthcare delivery - Dependence on vendor innovation - Financial expenses in DHT implementation - HCP travel cost savings - HCP workload efficiency improvement - Implementation costs of DHT - Improvement in efficiency of healthcare delivery - Improvement of HCP workflow efficiency - Availability of privacy policies - Clear data protection policies - Concern over authority access to data - Provision of private spaces for use - Absence of structured regulatory frameworks - Availability of data governance rules - Availability of data integrity regulations - Availability of data safety regulations - Clear DHT regulations - DHT policy - DHT practical guidelines and ethical standards - Digital redlining - Equity of access issue - Government guidance - Government policy gaps - National ownership limitations - Ownership in DHT implementation - Political and institutional backing - Reduction of healthcare disparities - Regulatory registration requirements - Strictness of data integrity regulations - Strictness of data safety regulations - Support from DHT policies and protocols - Support from structured regulatory frameworks - Supportive legal frameworks for DHT - Transparency in financial resource allocation - Unclear hospital ownership - Unclear ownership of systems - Government credibility - Improvement in transparency of healthcare decisions - Institutional endorsement as source of trust - Institutional trust - Reassurance from trusted organizations - Scientific society membership - Transparency requirements in evaluation - Trust in government - Trust in hospital-approved content - Trust in hospitals - Trust in institutions - Completeness of administrative information - HCP time constraints - HCP time savings - HCP workload constraints | **Category 1: Stability and reliability of DHT systems**   - App slowness - Backup system needs - Data storage capacity limitations - Device malfunction - DHT functionality gaps - DHT resilience - Limited system responsiveness - Server downtime - System quality - System reset disruption - Trust in system reliability   **Category 2: Fairness and transparency of AI in DHT**   - Human touch preference in AI decision-making systems - Preference for hybrid AI–human interaction - Awareness of AI value - Perceived control and transparency over AI - Requirement for trust in AI service - Skepticism about AI capabilities - Skepticism toward AI - Trust in AI service - HCP concern about DHT algorithmic bias - Bias in AI algorithms - Complexity of AI systems   **Category 3: Reminder and scheduling functions in DHT**   - Support for medication adherence - Fear of unnecessary messages - Message overload - Notification fatigue - Reminder-based adherence - Reminder-driven motivation - User forgetfulness in DHT use - Scheduling support - Absence of notifications - Chatbot support features - Reminder facilitation - Supportive reminders   **Category 4: Clinical effectiveness and health outcomes of DHT**   - Visual analytics for stress awareness - Clinical benefits - Disease prevention benefits - Health monitoring benefits - Holistic health assessment need - Improvement of healthcare service quality - Real-time health monitoring - Support for symptom recognition and response - User health monitoring support - Value of real-time health metric monitoring - Outcome observability - Outcome visibility - Demonstrability of results - Immediate output visibility   **Category 5: Continuity of care through remote consultations**   - Preference for in-person care - Preference for in-person consultations - Preference for in-person interaction - Preference for in-person reassurance - Preference for in-person visits - HCP-enabled continuity of care - Long-term therapy experience - Therapeutic continuity between sessions - Continuity of remote consultation - HCP–patient relationship enhancement - HCP–patient relationship improvement - Lack of continuity with HCP - Preferred access to doctor   **Category 6: Accuracy and completeness of DHT data**   - Completeness of administrative information - Clinical information completeness - Clinical information errors - Concerns about inconsistent or conflicting DHT results - Data reliability concern - User health data accuracy - Skepticism about DHT accuracy - Skepticism about DHT measurement accuracy - Accuracy of patient information within system - Administrative information errors - Completeness of patient information within system - Data errors - Data quality - Device calibration issue - Difficulty in step tracking - Health information repository - Patient-generated health data   **Category 7: User confidence and self-efficacy in DHT**   - Age-based confidence gap - Anxiety from health data - Digital comfort - Digital confidence - Fear of technology as a barrier - Health self-efficacy - Patient empowerment through health data management - Patient empowerment through self-care - Patient empowerment through self-monitoring - Patient self-efficacy - Self-management during therapy waiting periods - Technology self-efficacy - User computer self-efficacy - User emotional distress during use - User fatigue in DHT use - User resistance to change - User technology-related anxiety   **Category 8: Affordability of DHT devices and services**   - Affordability for DHT - Affordable data barrier - Affordable healthcare access - Availability of financial support - Barrier to accessibility of payment methods - Cost of apps - Cost of equipment or device - Cost savings through discounts - Financial cost barrier - Financial expense of DHT - Financial resources accessibility - Health insurance - Hidden costs of digital care - Internet bundle cost - Internet cost barrier - Preference for free apps - Relief of cost burden for migrants - Uncertainty about technology affordability - Perceived cost reducing benefit valuation - User cost-benefit perception   **Category 9: Integration of DHT into healthcare systems**   - Centrality of in-person care - Fragmented healthcare practices - Outdated healthcare systems - Risk of program fragmentation - HCP-enabled chronic illness monitoring - HCP-enabled rehabilitation tracking - Real-time care practice monitoring - Rehabilitation support and tracking - Use in population-level NCD risk data collection - Care coordination support - Connection to local healthcare services - Data sharing through HCP collaboration - Engagement in research - HCP access to patient health information - HCP bridging role in digital pathways - Improved care coordination - Limited post-prescription role - Reduced pharmacist interaction - Availability of DHT system in hospitals - Digital prescription management - Health system compatibility   **Category 10: Impact of DHT on healthcare professionals’ workload and performance**   - HCP time constraints - HCP time savings - HCP workload constraints - HCP workload response gap - Improvement of HCP work effectiveness - Improvement of HCP work performance - HCP decision-support benefits - HCP job performance enhancement - Improvement of HCP patient care - Concern over system burden - HCP emotional workload strain - HCP perceived benefits - HCP perceived usefulness - HCP performance expectancy - Information-sharing burden - Clinician review workload - HCP efficiency feedback need - HCP efficient information retrieval - HCP workflow change - HCP workflow integration - HCP workflow integration requirement - HCP-enabled health alerts prediction   **Category 11: Interoperability of devices and systems in healthcare**   - Data transfer standards - Institutional interoperability gap - Team profile enhancement - Bluetooth and phone proximity constraints - Cross-feature integration - Cross-system interoperability - Device syncing difficulties - Device–system connectivity - Difficulty in sharing information via eHealth systems - Interoperability across devices - Interoperability across settings - Interoperability between service - Lack of unified health database - Limitations in information sharing - Need for horizontal integration - Open API requirement for local customization - Platform integration gap - Poor system integration - Seamless device-app data synchronization - Seamless integration with existing devices and apps - Standardized reporting - Technology integration need - Tool–system integration - Within-system interoperability   **Category 12: Policy and program support for DHT adoption**   - Availability of pilot programs - Broadband policy and community program - DHT program continuity and practice needs - Financial subsidies for DHT implementation - Government funding support - Government support - International collaboration - National funding allocation - Opportunity for usage testing - Policy and community programs addressing digital disparities - Policymaker DHT training - Subsidy policies and community programs - Targeted financial and infrastructure strategies - Training policies and community programs - Support for consistent and continuous use - Community sensitization on DHT - Community support - Community- and hospital-based DHT workshops - Community-based device intermediation - DHT awareness campaigns - Employer responsibility for employee well-being - Preference for support hierarchy - Support services for users - Volunteer support program for DHT service   **Category 13: Privacy and security of user health data**   - Availability of privacy policies - Clear data protection policies - Concern over authority access to data - Provision of private spaces for use - Assurances of confidentiality - Concern over health data confidentiality - Confidentiality concerns in sensitive apps - Data privacy and security needs - Data security and privacy concerns - Provision of secure and validated information - Cybersecurity risk perception - Fear of data breaches - Fear of data misuse - Fear of hacking - Fear of phishing - Fear of privacy breaches - Fear of surveillance - Preference for anonymity in digital health use - HCP concern over patient data security and privacy - Confidential self-management affordances - Cybersecurity concern - Encrypted synchronization across DHTs - Trust in data security - Uncertainty of data transmission   **Category 14: Quality of remote patient–provider interactions**   - Real-time linkage with providers - Preference for efficiency over eye contact - Preference for in-person clinician interpretation - Active listening in remote care - Empathy challenges with DHT - Online communication competence - User expectation for fast replies - Communication skills in remote care - Directive counseling in remote care - Empathic rapport in remote care - Failure in HCP-patient communication channel - HCP clear feedback need - HCP concern about reduced empathy in digital communication - HCP content credibility in digital communication - HCP persuasion and empathy skills in remote care - HCP reliable feedback need - HCP-enabled patient communication - HCP-patient collaboration enhancement - HCP-patient collaboration expectation - HCP-patient communication enhancement - HCP-patient interaction quality - HCP–patient onboarding conversations - Improved patient-provider communication - Inconsistent communication practices - Isolation reduction through video call skills - Motivational interviewing in remote care - Patient-HCP relatedness - Reduced rapport in virtual care - Risk of miscommunication - Trust in interpersonal health information - User desire for human support in DHT use - HCP-patient communication tool   **Category 15: Organizational readiness, culture, and resources for DHT**   - Organizational culture for stress-related privacy - Organizational innovation culture - Capacity gaps in small institutions - Facility resource autonomy - Flexible organizational structures - Implementation planning need - Institutional directives - Institutional implementation maturity - Institutional influence - Institutional support - Lack of leadership support - Leadership endorsement - Organisational readiness to change - Organizational access gap - Organizational approval dependency - Organizational endorsement - Organizational support - Preference for phased rollout strategy - Presence of organizational champions - Shortage of HCPs and technical staff - Skilled workforce shortage - Stakeholder alignment - Testing environment barriers - Weak institutionalization - DHT adoption readiness - Digital readiness facilitator - Adequacy of organizational support - HCP engagement in DHT design - HCP exclusion from design - HCP-vendor relationships - Adequacy of technical resources - Availability of onsite technical expertise - Availability of technical resources - Lack of DHT readiness for NCD data integration   **Category 16: Quality and relevance of health information in DHT**   - Content irrelevance to local context - Provision of localized content - Appropriateness of health information - Education gap on side effects - Lack of medication information - Quality of health information in DHT system - Request for accurate oncology-specific information - Support for health information recall - Timing of health information provision - Challenge of result interpretation - Confusion about medical acronyms - Difficulty in test result interpretation - Difficulty with medical jargon - Framing of health information - Frustration from information overload - Graph interpretation difficulties - Health information overload - Misinformation vulnerability - Multi-source information seeking - Perceived reliability of health information - Preference for data presentation - Preference for numeric results - Search fatigue - User health information-seeking behaviour - User perceived relevance - User priority for information access over technical performance - Localized care navigation guidance - Dashboard-based data visualization - Ease of health information reporting - Graph and history features aiding symptom visualization and interpretation - Provision of health content library - Provision of more health information - Quantity of health information - Search history need   **Category 17: Clinical evidence and safety protocols for DHT**   - Constraints on feasibility of long-term telemonitoring - Digital communication policy for HCP availability - Expectation of escalation protocols - Need for standardized remote-care protocols - Oversight by certified companies - Professional guidance and standards for DHT - Requirement for safety nets - Absence of medical certification - Benefits of infection control - Clinical and implementation evidence - Evidence-based guidance - Fear of infection - HCP validation requirement (evidence-based) - Improvement of diagnostic accuracy in remote care - Lack of clinical evidence - Limited diagnostic capacity in remote care - Medically derived risks - Negative mental health impact of self-tracking - Overuse of self-tracking - Overuse risk of smartphone accessibility for children - Recognition of clinical advantages - Risk of carer over-reliance on DHT - Risk of counterfeit drugs - Risk of misdiagnosis in remote consultations - Risk of misinformation in remote consultations - Risk of over-monitoring - System-generated safety alerts for user - Trust in evidence-based and safe content - Trust in medically certified systems - User perceived safety in DHT - User safety assurance - Obsessive self-tracking - Attention and safety needs in remote consultation - HCP preference for pilot testing - Structured digital office hours - Unsafe school communication   **Category 18: Economic costs, efficiency, and sustainability of DHT**   - Attitude toward resource conservation - Administrative load - Benefit of HCP travel reduction - Benefit of price transparency - Budget constraint - Business model limitations in service scope - Convenience through reduced visits and travel - Cost efficiency for facilities and parties - Cost for DHT maintenance - Cost reduction in healthcare delivery - Dependence on vendor innovation - Financial expenses in DHT implementation - HCP travel cost savings - HCP workload efficiency improvement - Implementation costs of DHT - Improvement in efficiency of healthcare delivery - Improvement of HCP workflow efficiency - Improvement of healthcare resource allocation efficiency - Improvement of healthcare service efficiency - Insufficient reimbursement - Lack of insurance reimbursement - Low prescribing incentive - Paper reduction benefit - Remuneration availability - Uncertainty about funding sustainability - Uncertainty of post-implementation sustainability - User cost savings - User money savings - User time savings - Doubt about DHT sustainability due to failed pilots - DHT workflow efficiency - Duplication of effort in DHT use - Expectations of developers - HCP work duplication - Reduction of double documentation - Reduction of HCP work duplication - Simplification of record management - Workflow fit   **Category 19: Engagement features: feedback, personalization, and gamification**   - Availability of professional coaching - Coping support during waiting periods - Feedback motivating continued use - Reassurance from tracking features - Social competition motivation - User confirmation of chatbot expectations - User desire for feedback provision - User desire for personalized care - User satisfaction - Expectation of proactive support - Feedback for service design - Social competition through data sharing - User engagement in DHT design - Automatic DHT functions - Avatar customization - Device behavioural nudges - DHT functions tailored to individuals needs and preferences - Drawing and customization features - Feedback complexity - Feedback quality from DHT use - Gameplay difficulty balance need - Gamification - Gamification elements - Gamification requests - Gamified personalization - Gamified rewards - In-app nudges for reflection and self-care - Need for customizable design - Need for data-driven personalized interactions - Personalization interaction requirement - Personalization tailoring need - Personalized responses - Provision of personalized content - Provision of personalized health information - Real-time information feedback - Requests for game badges - Requests for game rewards - Tailored to user complex needs   **Category 20: User capability, literacy, and support for DHT adoption**   - Expert guidance and reassurance - Awareness of DHT - DHT knowledge gaps - Digital health literacy level - Digital literacy adaptation - Digital literacy level - Enhanced understanding of DHT - Guidance need for action - Health literacy level - Personally relevant leaning content - Prior computer experience - Prior ICT experience - Prior ICT familiarity - Prior internet use - Availability of help in DHT usage - Continuous user support - Educational support needs - Instructional aids and professional demonstration guidance - One-to-one DHT guidance and troubleshooting - Orientation facilitation - Practical task guidance - Professional guidance via chat or tools - Refresher training need - Structured DHT teaching and awareness - Technical assistance needs - Technical support - User continuous support on DHT use - User DHT education - User DHT guidance - User DHT training - User onboarding training - User pre-usage training - User technical skill development - User training with feedback - Volunteer tutoring as support scaffold - Absence of DHT learning resources and assistance - Multimedia aids for comprehension - Multimodal user guidance - Requests for animations   **Category 21: Regulatory, ethical, and equity standards for DHT**   - Ethnic disparity - Gaps in diversity and inclusion - Preservation of dignity - Racial disparity - Representation to avoid biased DHT development - Systemic racism - Absence of structured regulatory frameworks - Availability of data governance rules - Availability of data integrity regulations - Availability of data safety regulations - Clear DHT regulations - DHT policy - DHT practical guidelines and ethical standards - Digital redlining - Equity of access issue - Government guidance - Government policy gaps - National ownership limitations - Ownership in DHT implementation - Political and institutional backing - Reduction of healthcare disparities - Regulatory registration requirements - Strictness of data integrity regulations - Strictness of data safety regulations - Support from DHT policies and protocols - Support from structured regulatory frameworks - Supportive legal frameworks for DHT - Transparency in financial resource allocation - Unclear hospital ownership - Unclear ownership of systems - Concern about missing data reference values or standards - Nonmaleficence and potential harms - User ethical escalation prompts - Responsibility relevance - HCP fear of racial and ethnic bias in DHT use - Shared responsibility in care - User perceived marginalization - User personhood support - Availability of DHT regulations - Standardization of health information for HCP   **Category 22: Ease of use and interface design of DHT**   - Preference for non-clinical design - Familiarity through DHT similarity - Familiarity with DHT - Perceived time cost of learning DHT - Prior DHT experience - Uncertainty about ease of use - User effort expectancy - User perceived complexity - Adaptability design - Adaptation to DHT over time - App usability limitations - Apps complexity - Authentication and access difficulties - Barriers in account setup and management - DHT aesthetics - DHT usability issues - Difficulty in display readability - Ease of adjustment for usability - Emoji-based responses - Font size - Image clarity and quality - Interactive user experience features - Interface attractiveness - Intuitive interface design - Learnability of DHT - Learnability of wearable devices - Minimal interaction requirements in DHT use - Navigation complexity - Preference for intuitive DHT - Preference for simple DHT - Screen visibility and readability - System adaptability to evolving needs - Technological design - Usability and setup difficulties - Usability barrier from poor design - Usability of DHT - User ease of operation - User ease of use - User technical usability issues - User-friendly features - User-friendly system interface - Visual usability considerations   **Category 23: Inclusive and user-friendly device design**   - Language and cultural accessibility - Language barriers - Localized design for context - Translation needs - Convenience for mobility-limited individuals - Inclusive DHT framework - Weather adaptability - Person-centred digital care - Risk of strap irritation - Screen sensitivity - User burden of continuous wear - Wear-time tolerance limit - Adaptability to diverse needs - Device wearability burden - Effortful typing and device use - Input effort conflicts - User burden of device use - Accessibility for hard-to-reach populations - Co-design with specific groups - User inclusivity - Age-friendly design - Availability of multimodal user modes - Battery life - Convenience of passive data collection - Device portability for active lifestyle - Device size - Display size limitations - Ergonomic comfort of wearables - Inclusive design - Lightweight device design - Minimal interaction in DHT use - Multi-language options - Multilingual support - Passive activity logging reducing effort - Personalisation for diverse needs - Preference for simple and accessible design - Simple device design - Stigma reduction interface - Supportive design - Unobtrusive device design - Usability for diverse needs - Wearability comfort   **Category 24: Trust in providers and user control over data**   - Emphasis on voluntary use - Preference for face-to-face care - Transparency for personhood - Government credibility - Improvement in transparency of healthcare decisions - Institutional endorsement as source of trust - Institutional trust - Reassurance from trusted organizations - Scientific society membership - Transparency requirements in evaluation - Trust in government - Trust in hospital-approved content - Trust in hospitals - Trust in institutions - Consent and boundary setting - Consent boundary setting - User control over health data - Benefit-based privacy decision-making - Conditional data sharing for personalization - Confidence in telemedicine platforms - Consultation choice autonomy - Distrust in app providers - Distrust in commercial providers - Internet trust limitations - Loss of autonomy from surveillance - Perceived value of data management - Suspicion toward DHT - Trust in DHT - Uncertainty of usefulness - Usefulness of DHT - User autonomy in data sharing - User autonomy in DHT use - User control over engagement - User perceived lack of direct usefulness - User perceived usefulness - User perceived utility - Doctor-patient trust - Family access to user data - HCP influence - HCP recommendations - Preference for accessible local providers - Trust erosion due to care gaps - Trust in healthcare professionals - Trust through recommendations from HCP, family, peers, and institutions   **Category 25: Motivation and habit formation in DHT use**   - Financial incentives - Monetary incentives - Non-financial incentives for HCP stakeholders - Rapid technology turnover - Sustainability concern - Age-related reluctance and decline in motivation - Altruistic motivation - Coach-driven motivation - Coach-mediated accountability - Commitment to DHT use - Continuity of care motivation - Curiosity satisfaction - Device-driven motivation - External motivation - HCP habitual DHT use - Health condition awareness driven adoption - Increasing health awareness - Interest in DHT - Internal motivation - Intrinsic motivation - Knowledge-seeking behaviour - Openness to new technologies - Overcoming hesitancy due to necessity - Past fitness app use habits - Personal innovativeness - Personal optimism - Preferred use of DHT - Risk propensity - Stress-reduction motivation - User attitude toward DHT - User enjoyment - User future-oriented motivation - User habitual DHT use - User hedonic motivation - User hopefulness for future improvements - User hopefulness for work relevance - User intention to use - User perceived enjoyment - Wellness motivation for self-care - Motivation for team participation - Motivation from private group data exchange - Social motivation through data sharing - Types of habitual HCP use of DHT - Value of social belonging   **Category 26: Access to healthcare services and technology**   - Access to device through gifting - Computer ownership - Device availability need - DHT device availability - Electricity gaps - Healthcare access improvement - Improved healthcare service accessibility - Improvement of specialized care access - Internet accessibility - Internet connectivity - Internet connectivity and affordability - Internet-enabled devices - Low-threshold entry to care - Mobile phone ownership - Quality of available ICT infrastructure - Reliability of doorstep delivery - Requirement for service expansion - Rural healthcare access - Service expansion opportunity - Smartphone ownership - Uncertainty about DHT availability - User convenience in healthcare access - Chronic disease management needs - Convenience for sick individuals - Convenience of home testing - Demand for ongoing care monitoring - Disease management needs - Emergency access benefit - Evolving mental health needs - Timeliness of access to care and results - Timeliness of health information for HCP - Timely consultations and monitoring - Access to remote consultation - Advantage of flexible access - Appointment access delays - Availability of administrative support - Availability of health support - Delays in consultations and report submissions - Accessibility of DHT - Availability and compatibility of technical devices - Availability of DHT - Availability of DHT infrastructure - Availability of digital infrastructure - Convenience of quick answers - Portal availability and utilization - Technical connectivity   **Category 27: Capacity and attitudes of healthcare professionals toward DHT**   - Anti-ageism pedagogy - HCP preference for traditional tools - Resistance to change in healthcare - Resistance to digital substitutes - HCP malpractice liability concern - HCP workforce shortage - Broad clinical knowledge for remote care triage - Risk of HCP overreliance on DHT - Fear of technology replacing service for savings - HCP effort expectancy - HCP familiarity with DHT - HCP knowledge on DHT - HCP level of DHT use - HCP motivation to use - HCP resistance to adoption - HCP resistance to change - HCP self-arranged learning - HCP technical skill level - Perceived complexity by HCP - Prior bedside experience as capability base - Skepticism from prior rehabilitation experience - Availability of HCP support - Continuous HCP learning - Continuous HCP training - Erosion of HCP role boundaries - Expectation of HCP AI competence - Expectation of HCP DHT knowledge - HCP as prescription gatekeeper - HCP attitude toward DHT - HCP autonomy concerns - HCP awareness of DHT - HCP DHT training - HCP DHT training needs - HCP digital skills gap - HCP guidance on anomaly explanation - HCP guidance on result review - HCP guidance requirement for DHT use - HCP guiding skills development - HCP professional role - HCP technical skill development - HCP training for digital communication - Stakeholder DHT training - Trust-building among HCPs - Type of HCP role - Types of HCP DHT use - HCP ease of use   **Category 28: Fit of DHT with users’ lifestyles, goals, and values**   - Culture-specific lifestyle guidance - Competing demands of school and workplace - Convenience in busy contexts - User convenience in health tracking - User time conflicts - User time constraints - User work time constraints - Alignment with user health practices - Disease severity - Health management and technology use - Illness-forced lifestyle change - Improvement of home care coping procedures - Individual health condition - Influence of disease stage - Support for safe independent living - Symptom tracking - User health performance evaluation - Adaptability to daily life and environments - Awareness of DHT value - Data reflection - Desire for longevity and well-being - Development of home care coping skills - Disease coping stage - Enhancement of emotional well-being - Exercise goal setting - Expectation of daily life communication aid - Expectation of daily life memory aid - Goal of quality-of-life enhancement - Health improvement goal - Health self-management goal setting - Healthy lifestyle orientation - Heart rate goal setting - Integration into health routines - Integration of DHT into self-care routines - Internet use duration - Life goal alignment - Lifestyle integration - Maintenance of independence in daily living - Nutrition goal setting - Outcome expectancy - Perceived susceptibility - Perceived value in service delivery - Perception of lifestyle–technology mismatch - Personal fitness improvement goals - Personal interest in nutrition - Priority of usefulness over novelty - Quality-of-life enhancement - Reassessment of changing needs - Relaxation support - Sleep goal setting - Types of priorities in DHT use - Uncertainty about DHT role in health management - User accountability in health behaviours - User health beliefs - User lifestyle fit - User perceived benefits - User perceived health benefits - User perceived lack of need - User performance expectancy - User priorities and needs conflict - User relative advantage - User self-recognition of health behaviours - User value compatibility - Weight management goal setting - Daily life communication support - Ease of photo tracking - Environmental usability - User activity monitoring - User aid in daily task management - User health data monitoring function   **Category 29: Social, cultural, and equity factors affecting DHT use**   - Cultural acceptance - Cultural fear or dislike preference - Cultural stigma against mental illness - Influence of gender - Pandemic-driven social acceptance - Preference for cultural concordance - Preference for non-digital stress coping strategies - Religious beliefs - Social normalization of DHT - Stigma barrier to DHT use - Stigma from clinical looking devices - Stigma from disability-oriented design - User preference for traditional tools - Caregiver paper record burden - Competitive pressure - Convenience during isolation or quarantine - Digital divide barrier - Geographical disparities - Impacts of COVID-19 on home care services - Pandemic-driven adoption - Pandemic-driven necessity - Rural inequalities - Social precarity - Socioeconomic status - Comorbidity-related barriers - Disability-related barriers - Family health needs - Fear of pandemic - Functional limitations driven adoption - Maternal phase-specific actionable feedback - Memory decline - Memory issues - Mobility limitations - Outbreak pattern detection - Physical health issues - Pregnancy and child milestone-based guidance - Timely maternal phase-specific updates for engagement - User barriers due to functional limitations - Barriers from denial of health condition - Caregiving competing priorities - Cognitive issues - Concern about age-related digital divide - Education level - Fear of judgement in DHT use - General literacy constraints - Influence of age - Influence of online health information - Internalized ageism - Pandemic awareness influence - Pandemic-induced anxiety - Post-pandemic intention to use DHT - Professional work history - Reliance on social media for health information - Resistance and concealment behaviours - Stigma avoidance - Ageism among HCP - Benefit of stigma reduction - Caregiver isolation - Caregiver training burden in information management - Caregiving workload burden - Colleagues influence - Conflict of family involvement with medical advice - Coworkers encouragement - Demand for carer involvement - DHT awareness through social media - Expectation from supervisors - Family assistance support - Family encouragement - Family influence - Family modeling effect - Family recommendations - Family reminder support - Family responsibilities - Family support - Family technical assistance support - Family-level resistance - Friends encouragement - Household responsibility - Influence of occupation - Influence of peer reviews - Marital status - Negative spousal influence - Observability of electronic word-of-mouth (eWOM) - Parental influence - Parental perceptions for value - Patient influence - Peer connection via app - Peer endorsement - Peer influence - Peer norms - Peer recommendations - Peer storytelling for reassurance and normalization - Peer support - Peer support through safe sharing - Peer validation through data sharing - Priority for instrumental support - Recognition of carer contributions - Reduction of caregiving burden - Reference group influence - Relatives’ motivational help - Relatives' support in navigating systems - Relatives’ technical assistance - Social connection improvement - Social connection support - Social endorsement within communities - Social media influence on body image - Social network support - Social obligations pressure - Support from close acquaintances - Trust in community or family sources - Trust in family - Trust in informal care - Underutilization of social platforms for health - Unrealistic body ideals from influencers - Workplace stigma risk from visibility - Adaptive tracking linked to maternal milestones | **Theme 1: Clinical Effectiveness and Quality of Care**   - Category 4: Clinical effectiveness and health outcomes of DHT - Category 5: Continuity of care through remote consultations - Category 14: Quality of remote patient–provider interactions - Category 17: Clinical evidence and safety protocols for DHT   **Theme 2: Trust, Privacy and Governance**   - Category 1: Stability and reliability of DHT systems - Category 2: Fairness and transparency of AI in DHT - Category 6: Accuracy and completeness of DHT data - Category 13: Privacy and security of user health data - Category 16: Quality and relevance of health information in DHT - Category 21: Regulatory, ethical, and equity standards for DHT - Category 24: Trust in providers and user control over data   **Theme 3: Integration, Workforce and Sustainability**   - Category 9: Integration of DHT into healthcare systems - Category 10: Impact of DHT on healthcare professionals’ workload and performance - Category 11: Interoperability of devices and systems in healthcare - Category 15: Organizational readiness, culture, and resources for DHT - Category 18: Economic costs, efficiency, and sustainability of DHT - Category 27: Capacity and attitudes of healthcare professionals toward DHT   **Theme 4: Access, Equity and Affordability**   - Category 8: Affordability of DHT devices and services - Category 12: Policy and program support for DHT adoption - Category 26: Access to healthcare services and technology - Category 29: Social, cultural, and equity factors affecting DHT use   **Theme 5: Usability, Engagement and User Empowerment**   - Category 3: Reminder and scheduling functions in DHT - Category 7: User confidence and self-efficacy in DHT - Category 19: Engagement features: feedback, personalization, and gamification - Category 20: User capability, literacy, and support for DHT adoption - Category 22: Ease of use and interface design of DHT - Category 23: Inclusive and user-friendly device design - Category 25: Motivation and habit formation in DHT use - Category 28: Fit of DHT with users’ lifestyles, goals, and values |
