# Supplementary material for: Advancing foundational models in digital health technology adoption: A systematic literature review of multidisciplinary factors
Source: PLOS Digit Health. 2026 Sep 2;5(9):e0001690. doi: 10.1371/journal.pdig.0001690 (PMC13537594; doi:10.1371/journal.pdig.0001690)
Supplement: Supplementary file 10 [file pdig.0001690.s008.docx]

**S2 Appendix. Item-level MMAT assessment of included studies**

# **Qualitative Studies**

| [[8]](https://journals.sagepub.com/doi/full/10.1177/00380385231218695) Alevizou P, Michaelidou N, Daskalopoulou A, Appiah-Campbell R. Self-tracking among young people: lived experiences, tensions and bodily outcomes. Sociology. 2024;58(4):947-64. doi:10.1177/00380385231218695 | | | |
| --- | --- | --- | --- |
| **Methodological quality criteria** | | **Responses** | **Comments** |
| S1. | Are there clear research questions? | Yes |  |
| S2. | Do the collected data allow to address the research questions? | Yes |  |
| 1.1 | Is the qualitative approach appropriate to answer the research question? | Yes |  |
| 1.2 | Are the qualitative data collection methods adequate to address the research question? | Yes |  |
| 1.3 | Are the findings adequately derived from data? | Yes |  |
| 1.4 | Is the interpretation of result sufficiently substantiated by data? | Yes |  |
| 1.5 | Is there coherence between qualitative data sources, collection, analysis and interpretation? | Yes |  |

| [[10]](https://journals.plos.org/digitalhealth/article?id=10.1371/journal.pdig.0000586) Alkaabi A, Elsori D. Navigating digital frontiers in UAE healthcare: a qualitative exploration of healthcare professionals’ and patients’ experiences with AI and telemedicine. PLOS Digit Health. 2025;4(4):e0000586. Doi:10.1371/journal.pdig.0000586 | | | |
| --- | --- | --- | --- |
| **Methodological quality criteria** | | **Responses** | **Comments** |
| S1. | Are there clear research questions? | Yes |  |
| S2. | Do the collected data allow to address the research questions? | Yes |  |
| 1.1 | Is the qualitative approach appropriate to answer the research question? | Yes |  |
| 1.2 | Are the qualitative data collection methods adequate to address the research question? | Yes |  |
| 1.3 | Are the findings adequately derived from data? | Yes |  |
| 1.4 | Is the interpretation of result sufficiently substantiated by data? | Yes |  |
| 1.5 | Is there coherence between qualitative data sources, collection, analysis and interpretation? | Yes |  |

| [[20]](https://pmc.ncbi.nlm.nih.gov/articles/PMC10990475/) Baik D, Reeder B, Coats H, Baker C, Jankowski C. Perceptions and attitudes toward a proposed digital health physical activity program among older family caregivers of persons with heart failure: a qualitative study. Inform Health Soc Care. 2023;48(3):239-51. Doi:10.1080/17538157.2023.2227704 | | | |
| --- | --- | --- | --- |
| **Methodological quality criteria** | | **Responses** | **Comments** |
| S1. | Are there clear research questions? | Yes |  |
| S2. | Do the collected data allow to address the research questions? | Yes |  |
| 1.1 | Is the qualitative approach appropriate to answer the research question? | Yes |  |
| 1.2 | Are the qualitative data collection methods adequate to address the research question? | Yes |  |
| 1.3 | Are the findings adequately derived from data? | Yes |  |
| 1.4 | Is the interpretation of result sufficiently substantiated by data? | Yes |  |
| 1.5 | Is there coherence between qualitative data sources, collection, analysis and interpretation? | Yes |  |

| [[21]](https://www.jmir.org/2020/9/e15307/) Burns K, Nicholas R, Beatson A, Chamorro-Koc M, Blackler A, Gottlieb U. Identifying mobile health engagement stages: interviews and observations for developing brief message content. J Med Internet Res. 2020;22(9):e15307. doi:10.2196/15307 | | | |
| --- | --- | --- | --- |
| **Methodological quality criteria** | | **Responses** | **Comments** |
| S1. | Are there clear research questions? | Yes |  |
| S2. | Do the collected data allow to address the research questions? | Yes |  |
| 1.1 | Is the qualitative approach appropriate to answer the research question? | Yes |  |
| 1.2 | Are the qualitative data collection methods adequate to address the research question? | Yes |  |
| 1.3 | Are the findings adequately derived from data? | Yes |  |
| 1.4 | Is the interpretation of result sufficiently substantiated by data? | Yes |  |
| 1.5 | Is there coherence between qualitative data sources, collection, analysis and interpretation? | Yes |  |

| [[24]](https://www.mdpi.com/2076-3425/15/5/464) Chen ML, Hsieh KL, Hsieh SL, Hsieh MC, Lo DCT, Hsu JL. Barriers to using mobile app-based cognitive testing in older adults with probable Alzheimer’s disease: a qualitative study. Brain Sci. 2025;15(5):464. doi:10.3390/brainsci15050464 | | | |
| --- | --- | --- | --- |
| **Methodological quality criteria** | | **Responses** | **Comments** |
| S1. | Are there clear research questions? | Yes |  |
| S2. | Do the collected data allow to address the research questions? | Yes |  |
| 1.1 | Is the qualitative approach appropriate to answer the research question? | Yes |  |
| 1.2 | Are the qualitative data collection methods adequate to address the research question? | Yes |  |
| 1.3 | Are the findings adequately derived from data? | Yes |  |
| 1.4 | Is the interpretation of result sufficiently substantiated by data? | Yes |  |
| 1.5 | Is there coherence between qualitative data sources, collection, analysis and interpretation? | Yes |  |

| [[25]](https://journals.sagepub.com/doi/10.1177/20552076231173560) Cheraghi-Sohi S, Davies K, Gordon L, Jones H, Sanders C, Ong BN. A study to explore the usefulness of a mobile health application to support people with mild cognitive and/or communication impairment due to dementia and their carers. Digit Health. 2023;9:20552076231173560. Doi:10.1177/20552076231173560 | | | |
| --- | --- | --- | --- |
| **Methodological quality criteria** | | **Responses** | **Comments** |
| S1. | Are there clear research questions? | Yes |  |
| S2. | Do the collected data allow to address the research questions? | Yes |  |
| 1.1 | Is the qualitative approach appropriate to answer the research question? | Yes |  |
| 1.2 | Are the qualitative data collection methods adequate to address the research question? | Yes |  |
| 1.3 | Are the findings adequately derived from data? | Yes |  |
| 1.4 | Is the interpretation of result sufficiently substantiated by data? | Yes |  |
| 1.5 | Is there coherence between qualitative data sources, collection, analysis and interpretation? | Yes |  |

| [[32]](https://journals.sagepub.com/doi/10.1177/20552076251334043) Goldberg DG, Soylu TG, Hoffman CF, Kishton RE, Cronholm PF. Clinicians’ perspectives on the adoption and implementation of EMR-integrated clinical decision support tools in primary care. Digit Health. 2025;11:20552076251334043. doi:10.1177/20552076251334043 | | | |
| --- | --- | --- | --- |
| **Methodological quality criteria** | | **Responses** | **Comments** |
| S1. | Are there clear research questions? | Yes |  |
| S2. | Do the collected data allow to address the research questions? | Yes |  |
| 1.1 | Is the qualitative approach appropriate to answer the research question? | Yes |  |
| 1.2 | Are the qualitative data collection methods adequate to address the research question? | Yes |  |
| 1.3 | Are the findings adequately derived from data? | Yes |  |
| 1.4 | Is the interpretation of result sufficiently substantiated by data? | Yes |  |
| 1.5 | Is there coherence between qualitative data sources, collection, analysis and interpretation? | Yes |  |

| [[34]](https://bmcnurs.biomedcentral.com/articles/10.1186/s12912-025-03108-7) He Y, Fan G, Fan G, et al. Exploring nurse and patient perspectives on WeChat-based prenatal education in Chinese public hospitals: a qualitative inquiry. BMC Nurs. 2025;24:459. Doi:10.1186/s12912-025-03108-7 | | | |
| --- | --- | --- | --- |
| **Methodological quality criteria** | | **Responses** | **Comments** |
| S1. | Are there clear research questions? | Yes |  |
| S2. | Do the collected data allow to address the research questions? | Yes |  |
| 1.1 | Is the qualitative approach appropriate to answer the research question? | Yes |  |
| 1.2 | Are the qualitative data collection methods adequate to address the research question? | Yes |  |
| 1.3 | Are the findings adequately derived from data? | Yes |  |
| 1.4 | Is the interpretation of result sufficiently substantiated by data? | Yes |  |
| 1.5 | Is there coherence between qualitative data sources, collection, analysis and interpretation? | Yes |  |

| [[36]](https://www.mdpi.com/2227-9032/8/4/494) Jeng MY, Yeh TM, Pai FY. Analyzing older adults’ perceived values of using smart bracelets by means–end chain. Healthcare (Basel). 2020;8(4):494. Doi:10.3390/healthcare8040494 | | | |
| --- | --- | --- | --- |
| **Methodological quality criteria** | | **Responses** | **Comments** |
| S1. | Are there clear research questions? | Yes |  |
| S2. | Do the collected data allow to address the research questions? | Yes |  |
| 1.1 | Is the qualitative approach appropriate to answer the research question? | Yes |  |
| 1.2 | Are the qualitative data collection methods adequate to address the research question? | Yes |  |
| 1.3 | Are the findings adequately derived from data? | Yes |  |
| 1.4 | Is the interpretation of result sufficiently substantiated by data? | No | The results are presented as abstract factors, matrices, and maps without participant quotations to verify that definitions (e.g., “Comfortable wear”) accurately reflect participants’ words. |
| 1.5 | Is there coherence between qualitative data sources, collection, analysis and interpretation? | Yes |  |

| [[38]](https://journals.lww.com/cinjournal/abstract/2025/05000/feasibility_and_acceptability_of_smartwatches_for.11.aspx) Johnson AH, Lee K, Reeder B, Popejoy L, Vogelsmeier A. Feasibility and acceptability of smartwatches for use by nursing home residents. CIN Comput Inform Nurs. 2025;43(5):e01245. Doi:10.1097/CIN.0000000000001245 | | | |
| --- | --- | --- | --- |
| **Methodological quality criteria** | | **Responses** | **Comments** |
| S1. | Are there clear research questions? | Yes |  |
| S2. | Do the collected data allow to address the research questions? | Yes |  |
| 1.1 | Is the qualitative approach appropriate to answer the research question? | Yes |  |
| 1.2 | Are the qualitative data collection methods adequate to address the research question? | Yes |  |
| 1.3 | Are the findings adequately derived from data? | Yes |  |
| 1.4 | Is the interpretation of result sufficiently substantiated by data? | Yes |  |
| 1.5 | Is there coherence between qualitative data sources, collection, analysis and interpretation? | Yes |  |

| [[39]](https://journals.lww.com/cinjournal/fulltext/2024/08000/user_needs_and_factors_associated_with_the.6.aspx) Kahsay DT, Salanterä S, Tommila M, Liukas T, Rosio R, Diji AK, et al. User needs and factors associated with the acceptability of audiovisual feedback devices for chest compression monitoring in cardiopulmonary resuscitation. CIN Comput Inform Nurs. 2024;42(8):583-92. Doi:10.1097/CIN.0000000000001126 | | | |
| --- | --- | --- | --- |
| **Methodological quality criteria** | | **Responses** | **Comments** |
| S1. | Are there clear research questions? | Yes |  |
| S2. | Do the collected data allow to address the research questions? | Yes |  |
| 1.1 | Is the qualitative approach appropriate to answer the research question? | Yes |  |
| 1.2 | Are the qualitative data collection methods adequate to address the research question? | Yes |  |
| 1.3 | Are the findings adequately derived from data? | Yes |  |
| 1.4 | Is the interpretation of result sufficiently substantiated by data? | Yes |  |
| 1.5 | Is there coherence between qualitative data sources, collection, analysis and interpretation? | Yes |  |

| [[40]](https://dl.acm.org/doi/10.1145/3609327) Karnatak N, Loughrin B, Kuo TA, Mateu-Silvernail O, Medhi Thies I, Thies W, et al. Is it even giving the correct reading or not? How trust and relationships mediate blood pressure management in India. ACM Trans Comput Hum Interact. 2023;30(6):90. Doi:10.1145/3609327 | | | |
| --- | --- | --- | --- |
| **Methodological quality criteria** | | **Responses** | **Comments** |
| S1. | Are there clear research questions? | Yes |  |
| S2. | Do the collected data allow to address the research questions? | Yes |  |
| 1.1 | Is the qualitative approach appropriate to answer the research question? | Yes |  |
| 1.2 | Are the qualitative data collection methods adequate to address the research question? | Yes |  |
| 1.3 | Are the findings adequately derived from data? | Yes |  |
| 1.4 | Is the interpretation of result sufficiently substantiated by data? | Yes |  |
| 1.5 | Is there coherence between qualitative data sources, collection, analysis and interpretation? | Yes |  |

| [[43]](https://www.frontiersin.org/journals/public-health/articles/10.3389/fpubh.2025.1531173/full) Kim B, Jeong CH, Blood E, Arthanat S, Corvini M, Golomski C, et al. Multilevel factors influencing eHealth adoption among older adults during the pandemic. Front Public Health. 2025;13:1531173. doi:10.3389/fpubh.2025.1531173 | | | |
| --- | --- | --- | --- |
| **Methodological quality criteria** | | **Responses** | **Comments** |
| S1. | Are there clear research questions? | Yes |  |
| S2. | Do the collected data allow to address the research questions? | Yes |  |
| 1.1 | Is the qualitative approach appropriate to answer the research question? | Yes |  |
| 1.2 | Are the qualitative data collection methods adequate to address the research question? | Yes |  |
| 1.3 | Are the findings adequately derived from data? | Yes |  |
| 1.4 | Is the interpretation of result sufficiently substantiated by data? | Yes |  |
| 1.5 | Is there coherence between qualitative data sources, collection, analysis and interpretation? | Yes |  |

| [[47]](https://journals.plos.org/plosone/article?id=10.1371/journal.pone.0303416) Kiruthu-Kamamia C, Berner-Rodoreda A, O’Bryan G, Sande O, Huwa J, Thawani A, et al. Acceptability and feasibility of a mobile electronic medical record system for community-based antiretroviral therapy in Lilongwe, Malawi: a rapid qualitative analysis. PLoS One. 2025;20(5):e0303416. Doi:10.1371/journal.pone.0303416 | | | |
| --- | --- | --- | --- |
| **Methodological quality criteria** | | **Responses** | **Comments** |
| S1. | Are there clear research questions? | Yes |  |
| S2. | Do the collected data allow to address the research questions? | Yes |  |
| 1.1 | Is the qualitative approach appropriate to answer the research question? | Yes |  |
| 1.2 | Are the qualitative data collection methods adequate to address the research question? | Yes |  |
| 1.3 | Are the findings adequately derived from data? | Yes |  |
| 1.4 | Is the interpretation of result sufficiently substantiated by data? | Yes |  |
| 1.5 | Is there coherence between qualitative data sources, collection, analysis and interpretation? | Yes |  |

| [[51]](https://bmcdigitalhealth.biomedcentral.com/articles/10.1186/s44247-024-00127-3) Leenen JPL, Jordens YJ, Wegman A, et al. Experiences of nurses working in novel virtual care centres in the Netherlands: a qualitative study. BMC Digital Health. 2024;2:70. Doi:10.1186/s44247-024-00127-3 | | | |
| --- | --- | --- | --- |
| **Methodological quality criteria** | | **Responses** | **Comments** |
| S1. | Are there clear research questions? | Yes |  |
| S2. | Do the collected data allow to address the research questions? | Yes |  |
| 1.1 | Is the qualitative approach appropriate to answer the research question? | Yes |  |
| 1.2 | Are the qualitative data collection methods adequate to address the research question? | Yes |  |
| 1.3 | Are the findings adequately derived from data? | Yes |  |
| 1.4 | Is the interpretation of result sufficiently substantiated by data? | Yes |  |
| 1.5 | Is there coherence between qualitative data sources, collection, analysis and interpretation? | Yes |  |

| [[52]](https://www.nature.com/articles/s41746-023-00924-6) Lee VV, Vijayakumar S, Ng WY, et al. Personalization and localization as key expectations of digital health intervention in women pre- to post-pregnancy. Npj Digit Med. 2023;6:183. Doi:10.1038/s41746-023-00924-6 | | | |
| --- | --- | --- | --- |
| **Methodological quality criteria** | | **Responses** | **Comments** |
| S1. | Are there clear research questions? | Yes |  |
| S2. | Do the collected data allow to address the research questions? | Yes |  |
| 1.1 | Is the qualitative approach appropriate to answer the research question? | Yes |  |
| 1.2 | Are the qualitative data collection methods adequate to address the research question? | Yes |  |
| 1.3 | Are the findings adequately derived from data? | Yes |  |
| 1.4 | Is the interpretation of result sufficiently substantiated by data? | Yes |  |
| 1.5 | Is there coherence between qualitative data sources, collection, analysis and interpretation? | Yes |  |

| [[53]](https://humanfactors.jmir.org/2024/1/e58641) Leong QY, Lee VV, Ng WY, Vijayakumar S, Lau NY, Mauritzon I, et al. Older adults’ perspectives and experiences with digital health in Singapore: qualitative study. JMIR Hum Factors. 2024;11:e58641. Doi:10.2196/58641 | | | |
| --- | --- | --- | --- |
| **Methodological quality criteria** | | **Responses** | **Comments** |
| S1. | Are there clear research questions? | Yes |  |
| S2. | Do the collected data allow to address the research questions? | Yes |  |
| 1.1 | Is the qualitative approach appropriate to answer the research question? | Yes |  |
| 1.2 | Are the qualitative data collection methods adequate to address the research question? | Yes |  |
| 1.3 | Are the findings adequately derived from data? | Yes |  |
| 1.4 | Is the interpretation of result sufficiently substantiated by data? | Yes |  |
| 1.5 | Is there coherence between qualitative data sources, collection, analysis and interpretation? | Yes |  |

| [[55]](https://academic.oup.com/heapol/article/37/8/943/6545194) Liverani M, Ir P, Perel P, Khan M, Balabanova D, Wiseman V. Assessing the potential of wearable health monitors for health system strengthening in low- and middle-income countries: a prospective study of technology adoption in Cambodia. Health Policy Plan. 2022;37(8):943-51. Doi:10.1093/heapol/czac019 | | | |
| --- | --- | --- | --- |
| **Methodological quality criteria** | | **Responses** | **Comments** |
| S1. | Are there clear research questions? | Yes |  |
| S2. | Do the collected data allow to address the research questions? | Yes |  |
| 1.1 | Is the qualitative approach appropriate to answer the research question? | Yes |  |
| 1.2 | Are the qualitative data collection methods adequate to address the research question? | Yes |  |
| 1.3 | Are the findings adequately derived from data? | Yes |  |
| 1.4 | Is the interpretation of result sufficiently substantiated by data? | Yes |  |
| 1.5 | Is there coherence between qualitative data sources, collection, analysis and interpretation? | Yes |  |

| [[56]](https://aging.jmir.org/2024/1/e52292) Lu SY, Yoon S, Yee WQ, Ngiam NHW, Ng KYY, Low LL. Experiences of a community-based digital intervention among older people living in a low-income neighborhood: qualitative study. JMIR Aging. 2024;7:e52292. Doi:10.2196/52292 | | | |
| --- | --- | --- | --- |
| **Methodological quality criteria** | | **Responses** | **Comments** |
| S1. | Are there clear research questions? | Yes |  |
| S2. | Do the collected data allow to address the research questions? | Yes |  |
| 1.1 | Is the qualitative approach appropriate to answer the research question? | Yes |  |
| 1.2 | Are the qualitative data collection methods adequate to address the research question? | Yes |  |
| 1.3 | Are the findings adequately derived from data? | Yes |  |
| 1.4 | Is the interpretation of result sufficiently substantiated by data? | Yes |  |
| 1.5 | Is there coherence between qualitative data sources, collection, analysis and interpretation? | Yes |  |

| [[57]](https://www.jmir.org/2024/1/e50457/) Manning JB, Blandford A, Edbrooke-Childs J. Facilitators of and barriers to teachers’ engagement with consumer technologies for stress management: qualitative study. J Med Internet Res. 2024;26:e50457. doi:10.2196/50457 | | | |
| --- | --- | --- | --- |
| **Methodological quality criteria** | | **Responses** | **Comments** |
| S1. | Are there clear research questions? | Yes |  |
| S2. | Do the collected data allow to address the research questions? | Yes |  |
| 1.1 | Is the qualitative approach appropriate to answer the research question? | Yes |  |
| 1.2 | Are the qualitative data collection methods adequate to address the research question? | Yes |  |
| 1.3 | Are the findings adequately derived from data? | Yes |  |
| 1.4 | Is the interpretation of result sufficiently substantiated by data? | Yes |  |
| 1.5 | Is there coherence between qualitative data sources, collection, analysis and interpretation? | Yes |  |

| [[59]](https://bmcpsychiatry.biomedcentral.com/articles/10.1186/s12888-021-03426-5) Melia R, Monahan L, Duggan J, et al. Exploring the experiences of mental health professionals engaged in the adoption of mobile health technology in Irish mental health services. BMC Psychiatry. 2021;21:412. doi:10.1186/s12888-021-03426-5 | | | |
| --- | --- | --- | --- |
| **Methodological quality criteria** | | **Responses** | **Comments** |
| S1. | Are there clear research questions? | Yes |  |
| S2. | Do the collected data allow to address the research questions? | Yes |  |
| 1.1 | Is the qualitative approach appropriate to answer the research question? | Yes |  |
| 1.2 | Are the qualitative data collection methods adequate to address the research question? | Yes |  |
| 1.3 | Are the findings adequately derived from data? | Yes |  |
| 1.4 | Is the interpretation of result sufficiently substantiated by data? | Yes |  |
| 1.5 | Is there coherence between qualitative data sources, collection, analysis and interpretation? | Yes |  |

| [[61]](https://www.tandfonline.com/doi/10.1080/03091902.2022.2089249?url_ver=Z39.88-2003&rfr_id=ori:rid:crossref.org&rfr_dat=cr_pub%20%200pubmed) Moody L, Wood E, Needham A, Booth A, Jimenez-Aranda A, Tindale W. Identifying individual enablers and barriers to the use of digital technology for the self-management of long-term conditions by older adults. J Med Eng Technol. 2022;46(6):448-61. doi:10.1080/03091902.2022.2089249 | | | |
| --- | --- | --- | --- |
| **Methodological quality criteria** | | **Responses** | **Comments** |
| S1. | Are there clear research questions? | Yes |  |
| S2. | Do the collected data allow to address the research questions? | Yes |  |
| 1.1 | Is the qualitative approach appropriate to answer the research question? | Yes |  |
| 1.2 | Are the qualitative data collection methods adequate to address the research question? | Yes |  |
| 1.3 | Are the findings adequately derived from data? | Yes |  |
| 1.4 | Is the interpretation of result sufficiently substantiated by data? | Can’t tell | The report includes isolated quoted terms (e.g., “clinical,” “medical”) but does not explicitly provide adequate participant quotations to sufficiently substantiate the interpretations and reported findings,. |
| 1.5 | Is there coherence between qualitative data sources, collection, analysis and interpretation? | Yes |  |

| [[63]](https://journals.sagepub.com/doi/10.1177/20552076251321698?url_ver=Z39.88-2003&rfr_id=ori:rid:crossref.org&rfr_dat=cr_pub%20%200pubmed) Mwaka ES, Bazzeketa D, Mirembe J, Emoru RD, Twimukye A, Kivumbi A. Barriers to and enhancement of the utilization of digital mental health interventions in low-resource settings: perceptions of young people in Uganda. Digital Health. 2025;11:20552076251321698. Doi:10.1177/20552076251321698 | | | |
| --- | --- | --- | --- |
| **Methodological quality criteria** | | **Responses** | **Comments** |
| S1. | Are there clear research questions? | Yes |  |
| S2. | Do the collected data allow to address the research questions? | Yes |  |
| 1.1 | Is the qualitative approach appropriate to answer the research question? | Yes |  |
| 1.2 | Are the qualitative data collection methods adequate to address the research question? | Yes |  |
| 1.3 | Are the findings adequately derived from data? | Yes |  |
| 1.4 | Is the interpretation of result sufficiently substantiated by data? | Yes |  |
| 1.5 | Is there coherence between qualitative data sources, collection, analysis and interpretation? | Yes |  |

| [[64]](https://journals.sagepub.com/doi/10.1177/20552076211064406?url_ver=Z39.88-2003&rfr_id=ori:rid:crossref.org&rfr_dat=cr_pub%20%200pubmed) Namatovu HK, Oyana TJ, Sol HG. Barriers to eHealth adoption in routine antenatal care practices: perspectives of expectant mothers in Uganda—a qualitative study using the unified theory of acceptance and use of technology model. Digit Health. 2021;7:20552076211064406. Doi:10.1177/20552076211064406 | | | |
| --- | --- | --- | --- |
| **Methodological quality criteria** | | **Responses** | **Comments** |
| S1. | Are there clear research questions? | Yes |  |
| S2. | Do the collected data allow to address the research questions? | Yes |  |
| 1.1 | Is the qualitative approach appropriate to answer the research question? | Yes |  |
| 1.2 | Are the qualitative data collection methods adequate to address the research question? | Yes |  |
| 1.3 | Are the findings adequately derived from data? | Yes |  |
| 1.4 | Is the interpretation of result sufficiently substantiated by data? | Yes |  |
| 1.5 | Is there coherence between qualitative data sources, collection, analysis and interpretation? | Yes |  |

| [[65]](https://onlinelibrary.wiley.com/doi/10.1155/2024/5578056) Nigatu AM, Yilma TM, Gezie LD, Gebrewold Y, Gullslett MK, Mengiste SA, et al. Barriers and facilitators experienced during the implementation of web-based teleradiology system in public hospitals of the Northwest Ethiopia: an interpretive description study. Int J Telemed Appl. 2024;2024:5578056. Doi:10.1155/2024/5578056 | | | |
| --- | --- | --- | --- |
| **Methodological quality criteria** | | **Responses** | **Comments** |
| S1. | Are there clear research questions? | Yes |  |
| S2. | Do the collected data allow to address the research questions? | Yes |  |
| 1.1 | Is the qualitative approach appropriate to answer the research question? | Yes |  |
| 1.2 | Are the qualitative data collection methods adequate to address the research question? | Yes |  |
| 1.3 | Are the findings adequately derived from data? | Yes |  |
| 1.4 | Is the interpretation of result sufficiently substantiated by data? | Yes |  |
| 1.5 | Is there coherence between qualitative data sources, collection, analysis and interpretation? | Yes |  |

| [[66]](https://www.mdpi.com/1660-4601/19/20/13714) O’Connor SR, Treanor C, Ward E, Wickens RA, O’Connell A, Culliford LA, et al. Patient acceptability of home monitoring for neovascular age-related macular degeneration reactivation: a qualitative study. Int J Environ Res Public Health. 2022;19(20):13714. Doi:10.3390/ijerph192013714 | | | |
| --- | --- | --- | --- |
| **Methodological quality criteria** | | **Responses** | **Comments** |
| S1. | Are there clear research questions? | Yes |  |
| S2. | Do the collected data allow to address the research questions? | Yes |  |
| 1.1 | Is the qualitative approach appropriate to answer the research question? | Yes |  |
| 1.2 | Are the qualitative data collection methods adequate to address the research question? | Yes |  |
| 1.3 | Are the findings adequately derived from data? | Yes |  |
| 1.4 | Is the interpretation of result sufficiently substantiated by data? | Yes |  |
| 1.5 | Is there coherence between qualitative data sources, collection, analysis and interpretation? | Yes |  |

| [[67]](https://www.mdpi.com/1999-5903/15/6/191) Patrickson B, Musker M, Thorpe D, van Kasteren Y, Bidargaddi N, Consumer and Carer Advisory Group (CCAG). In-depth co-design of mental health monitoring technologies by people with lived experience. Future Internet. 2023;15(6):191. Doi:10.3390/fi15060191 | | | |
| --- | --- | --- | --- |
| **Methodological quality criteria** | | **Responses** | **Comments** |
| S1. | Are there clear research questions? | Yes |  |
| S2. | Do the collected data allow to address the research questions? | Yes |  |
| 1.1 | Is the qualitative approach appropriate to answer the research question? | Yes |  |
| 1.2 | Are the qualitative data collection methods adequate to address the research question? | Yes |  |
| 1.3 | Are the findings adequately derived from data? | Yes |  |
| 1.4 | Is the interpretation of result sufficiently substantiated by data? | Yes |  |
| 1.5 | Is there coherence between qualitative data sources, collection, analysis and interpretation? | Yes |  |

| [[68]](https://www.frontiersin.org/journals/digital-health/articles/10.3389/fdgth.2024.1275569/full) Posselt J, Baumann E, Dierks ML. A qualitative interview study of patients’ attitudes towards and intention to use digital interventions for depressive disorders on prescription. Front Digit Health. 2024;6:1275569. Doi:10.3389/fdgth.2024.1275569 | | | |
| --- | --- | --- | --- |
| **Methodological quality criteria** | | **Responses** | **Comments** |
| S1. | Are there clear research questions? | Yes |  |
| S2. | Do the collected data allow to address the research questions? | Yes |  |
| 1.1 | Is the qualitative approach appropriate to answer the research question? | Yes |  |
| 1.2 | Are the qualitative data collection methods adequate to address the research question? | Yes |  |
| 1.3 | Are the findings adequately derived from data? | Yes |  |
| 1.4 | Is the interpretation of result sufficiently substantiated by data? | Yes |  |
| 1.5 | Is there coherence between qualitative data sources, collection, analysis and interpretation? | Yes |  |

| [[69]](https://onlinelibrary.wiley.com/doi/10.1111/nhs.12939) Rochmawati E, Kamilah F, Iskandar AC. Acceptance of e-health technology among older people: a qualitative study. Nurs Health Sci. 2022;24(2):437-46. Doi:10.1111/nhs.12939 | | | |
| --- | --- | --- | --- |
| **Methodological quality criteria** | | **Responses** | **Comments** |
| S1. | Are there clear research questions? | Yes |  |
| S2. | Do the collected data allow to address the research questions? | Yes |  |
| 1.1 | Is the qualitative approach appropriate to answer the research question? | Yes |  |
| 1.2 | Are the qualitative data collection methods adequate to address the research question? | Yes |  |
| 1.3 | Are the findings adequately derived from data? | Yes |  |
| 1.4 | Is the interpretation of result sufficiently substantiated by data? | Yes |  |
| 1.5 | Is there coherence between qualitative data sources, collection, analysis and interpretation? | Yes |  |

| [[70]](https://onlinelibrary.wiley.com/doi/10.1155/2024/2667732) Saeed H, Scahill S, Kim J, Moyaen R, Natarajan D, Soga A, et al. Pharmacist perceptions and future scope of telepharmacy in New Zealand: a qualitative exploration. Int J Telemed Appl. 2024;2024:2667732. Doi:10.1155/2024/2667732 | | | |
| --- | --- | --- | --- |
| **Methodological quality criteria** | | **Responses** | **Comments** |
| S1. | Are there clear research questions? | Yes |  |
| S2. | Do the collected data allow to address the research questions? | Yes |  |
| 1.1 | Is the qualitative approach appropriate to answer the research question? | Yes |  |
| 1.2 | Are the qualitative data collection methods adequate to address the research question? | Yes |  |
| 1.3 | Are the findings adequately derived from data? | Yes |  |
| 1.4 | Is the interpretation of result sufficiently substantiated by data? | Yes |  |
| 1.5 | Is there coherence between qualitative data sources, collection, analysis and interpretation? | Yes |  |

| [[71]](https://bmcpublichealth.biomedcentral.com/articles/10.1186/s12889-024-20265-7) Sampene AK, Li C, Wiredu J. Unravelling the shift: exploring consumers’ adoption or resistance of e-pharmacy through behavioural reasoning theory. BMC Public Health. 2024;24:2789. Doi:10.1186/s12889-024-20265-7 | | | |
| --- | --- | --- | --- |
| **Methodological quality criteria** | | **Responses** | **Comments** |
| S1. | Are there clear research questions? | Yes |  |
| S2. | Do the collected data allow to address the research questions? | Yes |  |
| 1.1 | Is the qualitative approach appropriate to answer the research question? | Yes |  |
| 1.2 | Are the qualitative data collection methods adequate to address the research question? | Yes |  |
| 1.3 | Are the findings adequately derived from data? | Yes |  |
| 1.4 | Is the interpretation of result sufficiently substantiated by data? | Yes |  |
| 1.5 | Is there coherence between qualitative data sources, collection, analysis and interpretation? | Yes |  |

| [[72]](https://mhealth.jmir.org/2024/1/e48345) Schroeder T, Haug M, Georgiou A, Seaman K, Gewald H. Evidence of how physicians and their patients adopt mHealth apps in Germany: exploratory qualitative study. JMIR Mhealth Uhealth. 2024;12:e48345. Doi:10.2196/48345 | | | |
| --- | --- | --- | --- |
| **Methodological quality criteria** | | **Responses** | **Comments** |
| S1. | Are there clear research questions? | Yes |  |
| S2. | Do the collected data allow to address the research questions? | Yes |  |
| 1.1 | Is the qualitative approach appropriate to answer the research question? | Yes |  |
| 1.2 | Are the qualitative data collection methods adequate to address the research question? | Yes |  |
| 1.3 | Are the findings adequately derived from data? | Yes |  |
| 1.4 | Is the interpretation of result sufficiently substantiated by data? | Yes |  |
| 1.5 | Is there coherence between qualitative data sources, collection, analysis and interpretation? | Yes |  |

| [[73]](https://journals.sagepub.com/doi/10.1177/20552076251326160) Sezgin E, Jackson DI, Kaufman K, Skeens MA, Gerhardt CA, Moscato E. Perceptions about the use of virtual assistants for seeking health information among caregivers of young childhood cancer survivors. Digital Health. 2025;11:20552076251326160. Doi:10.1177/20552076251326160 | | | |
| --- | --- | --- | --- |
| **Methodological quality criteria** | | **Responses** | **Comments** |
| S1. | Are there clear research questions? | Yes |  |
| S2. | Do the collected data allow to address the research questions? | Yes |  |
| 1.1 | Is the qualitative approach appropriate to answer the research question? | Yes |  |
| 1.2 | Are the qualitative data collection methods adequate to address the research question? | Yes |  |
| 1.3 | Are the findings adequately derived from data? | Yes |  |
| 1.4 | Is the interpretation of result sufficiently substantiated by data? | Yes |  |
| 1.5 | Is there coherence between qualitative data sources, collection, analysis and interpretation? | Yes |  |

| [[74]](https://www.researchgate.net/publication/336011768_Exploring_the_barriers_and_facilitators_for_the_use_of_digital_health_technologies_for_the_management_of_COPD_A_qualitative_study_of_clinician_perceptions) Slevin P, Kessie T, Cullen J, Butler MW, Donnelly SC, Caulfield B. Exploring the barriers and facilitators for the use of digital health technologies for the management of COPD: a qualitative study of clinician perceptions. QJM. 2020;113(3):163-72. Doi:10.1093/qjmed/hcz241 | | | |
| --- | --- | --- | --- |
| **Methodological quality criteria** | | **Responses** | **Comments** |
| S1. | Are there clear research questions? | Yes |  |
| S2. | Do the collected data allow to address the research questions? | Yes |  |
| 1.1 | Is the qualitative approach appropriate to answer the research question? | Yes |  |
| 1.2 | Are the qualitative data collection methods adequate to address the research question? | Yes |  |
| 1.3 | Are the findings adequately derived from data? | Yes |  |
| 1.4 | Is the interpretation of result sufficiently substantiated by data? | Yes |  |
| 1.5 | Is there coherence between qualitative data sources, collection, analysis and interpretation? | Yes |  |

| [[75]](https://formative.jmir.org/2022/1/e28895/) Tennant R, Allana S, Mercer K, Burns CM. Exploring the experiences of family caregivers of children with special health care needs to inform the design of digital health systems: formative qualitative study. JMIR Form Res. 2022;6(1):e28895. Doi:10.2196/28895 | | | |
| --- | --- | --- | --- |
| **Methodological quality criteria** | | **Responses** | **Comments** |
| S1. | Are there clear research questions? | Yes |  |
| S2. | Do the collected data allow to address the research questions? | Yes |  |
| 1.1 | Is the qualitative approach appropriate to answer the research question? | Yes |  |
| 1.2 | Are the qualitative data collection methods adequate to address the research question? | Yes |  |
| 1.3 | Are the findings adequately derived from data? | Yes |  |
| 1.4 | Is the interpretation of result sufficiently substantiated by data? | Yes |  |
| 1.5 | Is there coherence between qualitative data sources, collection, analysis and interpretation? | Yes |  |

| [[76]](https://nursing.jmir.org/2023/1/e39815) Vercell A, Taylor S, Yorke J, Dowding D. Assessing the acceptability of home blood monitoring for patients with cancer who are receiving systemic anticancer therapy from a patient, caregiver, and clinician perspective: focus group and interview study. JMIR Nurs. 2023;6:e39815. Doi:10.2196/39815 | | | |
| --- | --- | --- | --- |
| **Methodological quality criteria** | | **Responses** | **Comments** |
| S1. | Are there clear research questions? | Yes |  |
| S2. | Do the collected data allow to address the research questions? | Yes |  |
| 1.1 | Is the qualitative approach appropriate to answer the research question? | Yes |  |
| 1.2 | Are the qualitative data collection methods adequate to address the research question? | Yes |  |
| 1.3 | Are the findings adequately derived from data? | Yes |  |
| 1.4 | Is the interpretation of result sufficiently substantiated by data? | Yes |  |
| 1.5 | Is there coherence between qualitative data sources, collection, analysis and interpretation? | Yes |  |

| [[77]](https://aisel.aisnet.org/icis2024/ishealthcare/ishealthcare/18/?utm_source=aisel.aisnet.org%2Ficis2024%2Fishealthcare%2Fishealthcare%2F18&utm_medium=PDF&utm_campaign=PDFCoverPages) Weeratunge SK, Bain C, Burstein F, Evans J. Can patient portals support digital health equity for culturally and linguistically diverse communities in Victoria: an exploratory study. In: ICIS 2024 Proceedings. 2024. Available from: <https://aisel.aisnet.org/icis2024/ishealthcare/ishealthcare/18> | | | |
| --- | --- | --- | --- |
| **Methodological quality criteria** | | **Responses** | **Comments** |
| S1. | Are there clear research questions? | Yes |  |
| S2. | Do the collected data allow to address the research questions? | Yes |  |
| 1.1 | Is the qualitative approach appropriate to answer the research question? | Yes |  |
| 1.2 | Are the qualitative data collection methods adequate to address the research question? | Yes |  |
| 1.3 | Are the findings adequately derived from data? | Yes |  |
| 1.4 | Is the interpretation of result sufficiently substantiated by data? | Yes |  |
| 1.5 | Is there coherence between qualitative data sources, collection, analysis and interpretation? | Yes |  |

| [[78]](https://humanfactors.jmir.org/2024/1/e51972) Zainal H, Hui XX, Thumboo J, Fong W, Yong FK. Patients’ expectations of doctors’ clinical competencies in the digital health care era: qualitative semistructured interview study among patients. JMIR Hum Factors. 2024;11:e51972. Doi:10.2196/51972 | | | |
| --- | --- | --- | --- |
| **Methodological quality criteria** | | **Responses** | **Comments** |
| S1. | Are there clear research questions? | Yes |  |
| S2. | Do the collected data allow to address the research questions? | Yes |  |
| 1.1 | Is the qualitative approach appropriate to answer the research question? | Yes |  |
| 1.2 | Are the qualitative data collection methods adequate to address the research question? | Yes |  |
| 1.3 | Are the findings adequately derived from data? | Yes |  |
| 1.4 | Is the interpretation of result sufficiently substantiated by data? | Yes |  |
| 1.5 | Is there coherence between qualitative data sources, collection, analysis and interpretation? | Yes |  |

| [[80]](https://www.emerald.com/tqm/article-abstract/34/6/1772/377388/Toward-digital-transformation-in-healthcare-a?redirectedFrom=fulltext) Binci D, Palozzi G, Scafarto F. Toward digital transformation in healthcare: a framework for remote monitoring adoption. TQMJ. 2022;34(6):1772-99. Doi:10.1108/TQM-04-2021-0109 | | | |
| --- | --- | --- | --- |
| **Methodological quality criteria** | | **Responses** | **Comments** |
| S1. | Are there clear research questions? | Yes |  |
| S2. | Do the collected data allow to address the research questions? | Yes |  |
| 1.1 | Is the qualitative approach appropriate to answer the research question? | Yes |  |
| 1.2 | Are the qualitative data collection methods adequate to address the research question? | Yes |  |
| 1.3 | Are the findings adequately derived from data? | Yes |  |
| 1.4 | Is the interpretation of result sufficiently substantiated by data? | Yes |  |
| 1.5 | Is there coherence between qualitative data sources, collection, analysis and interpretation? | Yes |  |

| [[81]](https://www.emerald.com/jstp/article-abstract/32/2/105/254615/I-m-no-expert-but-Consumer-use-of-supportive?redirectedFrom=fulltext) Bocking H, Russell-Bennett R, Letheren K. I’m no expert, but … ? Consumer use of supportive digital tools in health services. J Serv Theory Pract. 2022;32(2):105-31. Doi:10.1108/JSTP-09-2020-0225 | | | |
| --- | --- | --- | --- |
| **Methodological quality criteria** | | **Responses** | **Comments** |
| S1. | Are there clear research questions? | Yes |  |
| S2. | Do the collected data allow to address the research questions? | Yes |  |
| 1.1 | Is the qualitative approach appropriate to answer the research question? | Yes |  |
| 1.2 | Are the qualitative data collection methods adequate to address the research question? | Yes |  |
| 1.3 | Are the findings adequately derived from data? | Yes |  |
| 1.4 | Is the interpretation of result sufficiently substantiated by data? | Yes |  |
| 1.5 | Is there coherence between qualitative data sources, collection, analysis and interpretation? | Yes |  |

| [[82]](https://aisel.aisnet.org/cgi/viewcontent.cgi?article=1102&context=pacis2024) Mukherjee S, Das S. Willingness to digitalize health records: an Indian perspective. In: PACIS 2024 Proceedings. 2024. Available from: <https://aisel.aisnet.org/pacis2024/track11_healthit/track11_healthit/8> | | | |
| --- | --- | --- | --- |
| **Methodological quality criteria** | | **Responses** | **Comments** |
| S1. | Are there clear research questions? | Yes |  |
| S2. | Do the collected data allow to address the research questions? | Yes |  |
| 1.1 | Is the qualitative approach appropriate to answer the research question? | Yes |  |
| 1.2 | Are the qualitative data collection methods adequate to address the research question? | Yes |  |
| 1.3 | Are the findings adequately derived from data? | Yes |  |
| 1.4 | Is the interpretation of result sufficiently substantiated by data? | Yes |  |
| 1.5 | Is there coherence between qualitative data sources, collection, analysis and interpretation? | Yes |  |

# **Quantitative Non-Randomized**

| [[46]](https://pmc.ncbi.nlm.nih.gov/articles/PMC9790857/) Kim YK, Ang S. Older adults with functional limitations and their use of telehealth during COVID-19. Res Aging. 2023;45(9-10):609-19. Doi:10.1177/01640275221147642 | | | |
| --- | --- | --- | --- |
| **Methodological quality criteria** | | **Responses** | **Comments** |
| S1. | Are there clear research questions? | Yes |  |
| S2. | Do the collected data allow to address the research questions? | Yes |  |
| 3.1 | Are the participants representative of the target population? | Yes |  |
| 3.2 | Are the measurements appropriate regarding both the outcome and intervention (or exposure)? | Yes |  |
| 3.3 | Are there complete outcome data? | Yes |  |
| 3.4 | Are the co-founders accounted for in the design and analysis? | Yes |  |
| 3.5 | During the study period, is the intervention administered (or exposure occurred) as intended? | Yes |  |

| [[60]](https://www.frontiersin.org/journals/public-health/articles/10.3389/fpubh.2025.1542974/full) Meraya AM, Khardali A, Ahmad S, Hoshaibari AZ, Alameer AA, Jaafari RY, et al. Telehealth perceptions and associated factors among older adults with chronic conditions in Saudi Arabia: a comparative study of users and non-users. Front Public Health. 2025;13:1542974. Doi:10.3389/fpubh.2025.1542974 | | | |
| --- | --- | --- | --- |
| **Methodological quality criteria** | | **Responses** | **Comments** |
| S1. | Are there clear research questions? | Yes |  |
| S2. | Do the collected data allow to address the research questions? | Yes |  |
| 3.1 | Are the participants representative of the target population? | No | The study used purposive and snowball sampling, which the authors acknowledge may introduce selection bias and fail to capture a fully representative sample of older adults with chronic conditions. |
| 3.2 | Are the measurements appropriate regarding both the outcome and intervention (or exposure)? | Yes |  |
| 3.3 | Are there complete outcome data? | Can’t tell | The study does not report the response rate (approached vs completed) or the extent of missing data, which is required to assess non-response or exclusion bias. |
| 3.4 | Are the co-founders accounted for in the design and analysis? | No | The significant differences in occupation, education, and insurance were found between groups, but the SEM analysis description does not state that these variables were included as covariates. |
| 3.5 | During the study period, is the intervention administered (or exposure occurred) as intended? | No | The authors acknowledge lacking frequency data, meaning the definition of “user” (at least one appointment) may not accurately distinguish regular users from one-time users. |

# **Quantitative Descriptive**

| [[2]](https://mhealth.jmir.org/2021/1/e23155/) Yang S, Chen Y, Zhou L, Huang Y, Dai J. Willingness to adopt mHealth among Chinese parents during the COVID-19 outbreak: cross-sectional questionnaire study. JMIR Mhealth Uhealth. 2021;9(1):e23155. Doi:10.2196/23155 | | | |
| --- | --- | --- | --- |
| **Methodological quality criteria** | | **Responses** | **Comments** |
| S1. | Are there clear research questions? | Yes |  |
| S2. | Do the collected data allow to address the research questions? | Yes |  |
| 4.1 | Is the sampling strategy relevant to address the research question? | Can’t tell | The study reports forwarding the survey link to social media groups to “maximize recruitment” but does not explicitly justify this non-probability strategy in relation to the research question. |
| 4.2 | Is the sample representative of the target population? | Can’t tell | The recruitment relied on online social media groups requiring internet access, and the authors explicitly state the sample size was insufficient to ensure representativeness. |
| 4.3 | Are the measurements appropriate? | Yes |  |
| 4.4 | Is the risk of nonresponse bias low? | Can’t tell | An effective response rate of 93.38% is reported for those who started, the study does not report characteristics of non-respondents or reasons for non-participation to assess potential bias. |
| 4.5 | Is the statistical analysis appropriate to answer the research question? | Yes |  |

| [[3]](https://www.jmir.org/2021/6/e28944) Saigí-Rubió F, Vidal-Alaball J, Torrent-Sellens J, Jiménez-Zarco A, López Segui F, Carrasco Hernandez M, et al. Determinants of Catalan public primary care professionals’ intention to use digital clinical consultations (eConsulta) in the post–COVID-19 context: mixed methods study. J Med Internet Res. 2021;23(6):e28944. doi:10.2196/28944 | | | |
| --- | --- | --- | --- |
| **Methodological quality criteria** | | **Responses** | **Comments** |
| S1. | Are there clear research questions? | Yes |  |
| S2. | Do the collected data allow to address the research questions? | Yes |  |
| 4.1 | Is the sampling strategy relevant to address the research question? | Yes |  |
| 4.2 | Is the sample representative of the target population? | Can’t tell | Although demographic characteristics are reported, the response rate was low (~6.3%) and the study does not compare the sample (n=1,189) to the target population data to demonstrate representativeness. |
| 4.3 | Are the measurements appropriate? | Yes |  |
| 4.4 | Is the risk of nonresponse bias low? | Can’t tell | The authors explicitly acknowledge the study is subject to bias from response rates and do not report an analysis comparing respondents to non-respondents. |
| 4.5 | Is the statistical analysis appropriate to answer the research question? | Yes |  |
| ** Although the study claims a mixed-methods design, it does not meet MMAT criteria for mixed methods, as the qualitative component is limited to a literature review rather than primary qualitative data. The study is therefore classified as a quantitative descriptive study, with the literature review serving only as background context. | | | |

| [[4]](https://www.frontiersin.org/journals/medicine/articles/10.3389/fmed.2024.1421559/full) Abebe SA, Endehabtu BF, Walle AD, Hailu DT, Yeshaneh AT, Dres EA, et al. Intention to use personal health record system and its predictors among chronic patients enrolled at public hospitals in Bahir Dar city, northwest Ethiopia: using modified UTAUT2 model. Front Med (Lausanne). 2024;11:1421559. doi:10.3389/fmed.2024.1421559 | | | |
| --- | --- | --- | --- |
| **Methodological quality criteria** | | **Responses** | **Comments** |
| S1. | Are there clear research questions? | Yes |  |
| S2. | Do the collected data allow to address the research questions? | Yes |  |
| 4.1 | Is the sampling strategy relevant to address the research question? | Yes |  |
| 4.2 | Is the sample representative of the target population? | Yes |  |
| 4.3 | Are the measurements appropriate? | Yes |  |
| 4.4 | Is the risk of nonresponse bias low? | Yes |  |
| 4.5 | Is the statistical analysis appropriate to answer the research question? | Yes |  |

| [[5]](https://www.frontiersin.org/journals/health-services/articles/10.3389/frhs.2025.1460077/full) Alameraw TA, Asemahagn MA, Gashu KD, Walle AD, Kelkay JM, Mitiku AB, et al. Intention to use telemonitoring for chronic illness management and its associated factors among nurses and physicians at public hospitals in Bahir Dar, northwest Ethiopia: using a modified UTAUT2 model. Front Health Serv. 2025;5:1460077. Doi:10.3389/frhs.2025.1460077 | | | |
| --- | --- | --- | --- |
| **Methodological quality criteria** | | **Responses** | **Comments** |
| S1. | Are there clear research questions? | Yes |  |
| S2. | Do the collected data allow to address the research questions? | Yes |  |
| 4.1 | Is the sampling strategy relevant to address the research question? | Yes |  |
| 4.2 | Is the sample representative of the target population? | Yes |  |
| 4.3 | Are the measurements appropriate? | Yes |  |
| 4.4 | Is the risk of nonresponse bias low? | Yes |  |
| 4.5 | Is the statistical analysis appropriate to answer the research question? | Yes |  |

| [[6]](https://www.mdpi.com/2078-2489/13/11/509) Alanazi F, Gay V, Alturki R. Poor compliance of diabetic patients with AI-enabled e-health self-care management in Saudi Arabia. Information. 2022;13(11):509. Doi:10.3390/info13110509 | | | |
| --- | --- | --- | --- |
| **Methodological quality criteria** | | **Responses** | **Comments** |
| S1. | Are there clear research questions? | Yes |  |
| S2. | Do the collected data allow to address the research questions? | Yes |  |
| 4.1 | Is the sampling strategy relevant to address the research question? | Can’t tell | The sampling strategy is unclear because the authors claim to have selected patients registered on the CDSS from a composite list provided by Jeddah hospitals, yet the results contradict this inclusion criterion by revealing that only 48.4% of the participants were actually registered in the system. |
| 4.2 | Is the sample representative of the target population? | Can’t tell | The study was restricted to Jeddah, and the authors explicitly note this limits generalisability without providing data to demonstrate representativeness. |
| 4.3 | Are the measurements appropriate? | Yes |  |
| 4.4 | Is the risk of nonresponse bias low? | Can’t tell | Although 210 valid responses were analysed, the study does not report a response rate or conduct an analysis of non-respondents to rule out bias. |
| 4.5 | Is the statistical analysis appropriate to answer the research question? | Yes |  |

| [[7]](https://www.frontiersin.org/journals/public-health/articles/10.3389/fpubh.2024.1385713/full) Aldekhyyel RN, Alshuaibi F, Alsaaid O, Bin Moammar F, Alanazy T, Namshah A, et al. Exploring behavioral intention to use telemedicine services post COVID-19: a cross-sectional study in Saudi Arabia. Front Public Health. 2024;12:1385713. Doi:10.3389/fpubh.2024.1385713 | | | |
| --- | --- | --- | --- |
| **Methodological quality criteria** | | **Responses** | **Comments** |
| S1. | Are there clear research questions? | Yes |  |
| S2. | Do the collected data allow to address the research questions? | Yes |  |
| 4.1 | Is the sampling strategy relevant to address the research question? | No | The study utilised convenience sampling at two specific public venues, which is generally inadequate for achieving a representative sample of all Saudi health consumers. |
| 4.2 | Is the sample representative of the target population? | No | The authors explicitly acknowledge that the study unintendedly focused on a younger demographic (57% aged 18-20) living in Riyadh, limiting the generalisability of the findings. |
| 4.3 | Are the measurements appropriate? | Yes |  |
| 4.4 | Is the risk of nonresponse bias low? | Can’t tell | Although the final sample size (385) is reported, the study does not report a response rate or provide information on non-respondents to assess potential bias. |
| 4.5 | Is the statistical analysis appropriate to answer the research question? | Yes |  |

| [[9]](https://etasr.com/index.php/ETASR/article/view/8747) Aljohani N. Digital health transformation in Saudi Arabia: examining the impact of health information seeking on m-health adoption during the COVID-19 pandemic. Eng Technol Appl Sci Res. 2025;15(1):19933-40. Doi: 10.48084/etasr.8747 | | | |
| --- | --- | --- | --- |
| **Methodological quality criteria** | | **Responses** | **Comments** |
| S1. | Are there clear research questions? | Yes |  |
| S2. | Do the collected data allow to address the research questions? | Yes |  |
| 4.1 | Is the sampling strategy relevant to address the research question? | No | The study utilised convenience sampling via social media (Twitter and WhatsApp), which systematically excludes individuals less active on these platforms. |
| 4.2 | Is the sample representative of the target population? | No | The authors confirm findings primarily reflect younger users (32% aged 18–24; 45% aged 25–34) and admit limited applicability to older groups. |
| 4.3 | Are the measurements appropriate? | Yes |  |
| 4.4 | Is the risk of nonresponse bias low? | Can’t tell | Although 343 valid responses were obtained (85.75% of starts), the study does not report a comparison between respondents and non-respondents to assess potential bias. |
| 4.5 | Is the statistical analysis appropriate to answer the research question? | Yes |  |

| [[11]](https://www.iieta.org/journals/ijsdp/paper/10.18280/ijsdp.200309) Al-Shami SA, Kusmantini T, Purwandari S, Sharif MS, Doheir M, Rashid N, et al. System service quality factors and their impact on user satisfaction and continuance intention to use m-health: the moderating influence of monetary cost. Int J Sustain Dev Plan. 2025;20(3):1015-27. Doi:10.18280/ijsdp.200309 | | | |
| --- | --- | --- | --- |
| **Methodological quality criteria** | | **Responses** | **Comments** |
| S1. | Are there clear research questions? | Yes |  |
| S2. | Do the collected data allow to address the research questions? | Yes |  |
| 4.1 | Is the sampling strategy relevant to address the research question? | Yes |  |
| 4.2 | Is the sample representative of the target population? | No | The participants were drawn from a single healthcare setting (Imperial College London Diabetes Center), and authors admit findings may not apply to other institutions or regions. |
| 4.3 | Are the measurements appropriate? | Yes |  |
| 4.4 | Is the risk of nonresponse bias low? | Can’t tell | A 67% response rate is reported, but the study does not provide an analysis comparing the characteristics of respondents to non-respondents to rule out bias. |
| 4.5 | Is the statistical analysis appropriate to answer the research question? | Yes |  |

| [[12]](https://www.mdpi.com/1660-4601/19/7/4289) Alsharif AH. Attitudes of patients with chronic diseases toward management eHealth applications systems in post-COVID-19 times. Int J Environ Res Public Health. 2022;19(7):4289. Doi:10.3390/ijerph19074289 | | | |
| --- | --- | --- | --- |
| **Methodological quality criteria** | | **Responses** | **Comments** |
| S1. | Are there clear research questions? | Yes |  |
| S2. | Do the collected data allow to address the research questions? | Yes |  |
| 4.1 | Is the sampling strategy relevant to address the research question? | No | The study utilised convenience sampling via social media influencers, which systematically excludes individuals not active on these platforms and limits generalisability. |
| 4.2 | Is the sample representative of the target population? | No | The authors explicitly report that the younger population was overrepresented and acknowledge that generalisation of the findings must be carried out with care. |
| 4.3 | Are the measurements appropriate? | Yes |  |
| 4.4 | Is the risk of nonresponse bias low? | No | The study reports a high number of incomplete responses (138) and the final analytic sample is skewed toward a younger demographic, indicating significant non-observation error. |
| 4.5 | Is the statistical analysis appropriate to answer the research question? | Yes |  |

| [[13]](https://peerj.com/articles/cs-2773/) Al-Somali SA. Integrating artificial intelligence (AI) in healthcare: advancing older adults’ health management in Saudi Arabia through AI-powered chatbots. PeerJ Comput Sci. 2025;11:e2773. Doi:10.7717/peerj-cs.2773 | | | |
| --- | --- | --- | --- |
| **Methodological quality criteria** | | **Responses** | **Comments** |
| S1. | Are there clear research questions? | Yes |  |
| S2. | Do the collected data allow to address the research questions? | Yes |  |
| 4.1 | Is the sampling strategy relevant to address the research question? | Yes |  |
| 4.2 | Is the sample representative of the target population? | No | The study relied on convenience and snowball sampling via WhatsApp; the authors explicitly admit this approach overrepresents digitally literate users and limits generalizability to the broader elderly population. |
| 4.3 | Are the measurements appropriate? | Yes |  |
| 4.4 | Is the risk of nonresponse bias low? | Can’t tell | Although a 74% response rate is reported, the study does not provide an analysis comparing respondents to non-respondents to check for bias. |
| 4.5 | Is the statistical analysis appropriate to answer the research question? | Yes |  |

| [[14]](https://journals.sagepub.com/doi/10.1177/20552076251336271) Alsyouf A, Al-Momani AM, Alsubahi N, et al. Predicting digital contact tracing tool adoption during COVID-19 from the perspective of TAM: the role of trust, fear, privacy, anxiety, and social media. Digit Health. 2025;11:20552076251336271. Doi:10.1177/20552076251336271 | | | |
| --- | --- | --- | --- |
| **Methodological quality criteria** | | **Responses** | **Comments** |
| S1. | Are there clear research questions? | Yes |  |
| S2. | Do the collected data allow to address the research questions? | Yes |  |
| 4.1 | Is the sampling strategy relevant to address the research question? | No | The study utilised convenience sampling via social media and university groups, which does not provide an equal chance of selection and introduces high selection bias for a study aiming to generalise findings to smartphone users or citizens. |
| 4.2 | Is the sample representative of the target population? | No | The demographic data reveal the sample is skewed towards younger (49% aged 18–34), highly educated (54% with degrees), and Western province (59%) residents, and the authors acknowledge the need to assess generalisability. |
| 4.3 | Are the measurements appropriate? | Yes |  |
| 4.4 | Is the risk of nonresponse bias low? | Can’t tell | Although 400 complete surveys were reported, the study does not specify a response rate or provide a comparison between respondents and non-respondents to check for bias. |
| 4.5 | Is the statistical analysis appropriate to answer the research question? | Yes |  |

| [[15]](https://e-journal.unair.ac.id/JISEBI/article/view/41371) Alviani R, Purwandari B, Eitiveni I, Purwaningsih M. Factors affecting adoption of telemedicine for virtual healthcare services in Indonesia. J Inf Syst Eng Bus Intell. 2023;9(1):47-69. doi:10.20473/jisebi.9.1.47-69 | | | |
| --- | --- | --- | --- |
| **Methodological quality criteria** | | **Responses** | **Comments** |
| S1. | Are there clear research questions? | Yes |  |
| S2. | Do the collected data allow to address the research questions? | Yes |  |
| 4.1 | Is the sampling strategy relevant to address the research question? | No | The study utilised a convenience sampling method (online Google Form), which does not provide an equal chance of selection and introduces high selection bias for a study aiming to generalise to the Indonesian population. |
| 4.2 | Is the sample representative of the target population? | No | The authors explicitly state that the small sample size (n=144) cannot represent the population across the various regions of Indonesia. |
| 4.3 | Are the measurements appropriate? | Yes |  |
| 4.4 | Is the risk of nonresponse bias low? | Can’t tell | The study does not report a response rate, nor does it provide an analysis comparing respondents to non-respondents to rule out bias. |
| 4.5 | Is the statistical analysis appropriate to answer the research question? | Yes |  |

| [[17]](https://diabetes.jmir.org/2024/1/e56756) Arueyingho O, Aprioku JS, Marshall P, O’Kane AA. Insights into sociodemographic influences on type 2 diabetes care and opportunities for digital health promotion in Port Harcourt, Nigeria: quantitative study. JMIR Diabetes. 2024;9:e56756. Doi:10.2196/56756 | | | |
| --- | --- | --- | --- |
| **Methodological quality criteria** | | **Responses** | **Comments** |
| S1. | Are there clear research questions? | Yes |  |
| S2. | Do the collected data allow to address the research questions? | Yes |  |
| 4.1 | Is the sampling strategy relevant to address the research question? | No | The study utilised snowball and convenience sampling (e.g., social media, open markets), which implies that not everyone in the target population had an equal chance of being selected. |
| 4.2 | Is the sample representative of the target population? | No | The authors explicitly admit that the recruitment approach led to an “unfair representation” skewed towards high-income and educated groups. |
| 4.3 | Are the measurements appropriate? | Yes |  |
| 4.4 | Is the risk of nonresponse bias low? | Can’t tell | The study does not report an explicit response rate or a comparison between respondents and non-respondents to check for bias. |
| 4.5 | Is the statistical analysis appropriate to answer the research question? | Yes |  |

| [[22]](https://journals.plos.org/plosone/article?id=10.1371/journal.pone.0303302) Carrera A, Lettieri E, Lietti G, Martignoni S, Sgarbossa C, Cafazzo J. Therapies go digital. What drives physicians’ acceptance? PLoS One. 2024;19(5):e0303302. Doi:10.1371/journal.pone.0303302 | | | |
| --- | --- | --- | --- |
| **Methodological quality criteria** | | **Responses** | **Comments** |
| S1. | Are there clear research questions? | Yes |  |
| S2. | Do the collected data allow to address the research questions? | Yes |  |
| 4.1 | Is the sampling strategy relevant to address the research question? | No | The study utilised a voluntary web-based survey (non-probability sampling), which does not provide an equal chance of selection and introduces selection bias. |
| 4.2 | Is the sample representative of the target population? | Can’t tell | The survey was distributed to diabetologists via a leading scientific society, but the study does not explicitly report data demonstrating representativeness or comparing the sample to the population frame. |
| 4.3 | Are the measurements appropriate? | Yes |  |
| 4.4 | Is the risk of nonresponse bias low? | Can’t tell | Although the paper notes blank responses were disregarded, it does not report a response rate or conduct an analysis of non-respondents to assess potential bias. |
| 4.5 | Is the statistical analysis appropriate to answer the research question? | Yes |  |

| [[23]](https://www.frontiersin.org/journals/public-health/articles/10.3389/fpubh.2024.1449594/full) Wu C, Lim GG. Investigating older adults users’ willingness to adopt wearable devices by integrating the technology acceptance model (UTAUT2) and the Technology Readiness Index theory. Front Public Health. 2024;12:1449594. Doi:10.3389/fpubh.2024.1449594 | | | |
| --- | --- | --- | --- |
| **Methodological quality criteria** | | **Responses** | **Comments** |
| S1. | Are there clear research questions? | Yes |  |
| S2. | Do the collected data allow to address the research questions? | Yes |  |
| 4.1 | Is the sampling strategy relevant to address the research question? | No | The study utilised snowball and convenience sampling (online and subway intercepts), which does not provide an equal chance of selection and introduces high selection bias. |
| 4.2 | Is the sample representative of the target population? | No | The authors explicitly acknowledge that the sample does not fully account for cultural and socio-economic differences, and the non-probability method prevents statistical representativeness. |
| 4.3 | Are the measurements appropriate? | Yes |  |
| 4.4 | Is the risk of nonresponse bias low? | Can’t tell | Although invalid questionnaires were excluded, the study does not report a response rate or an explicit assessment of nonresponse bias to rule out error. |
| 4.5 | Is the statistical analysis appropriate to answer the research question? | Yes |  |

| [[26]](https://journals.sagepub.com/doi/10.1177/21582440241228639) Lu C-C, Tsai-Lin T-F. Are older adults special in adopting public eHealth service initiatives? The modified model of UTAUT. SAGE Open. 2024;14(1). Doi:10.1177/21582440241228639 | | | |
| --- | --- | --- | --- |
| **Methodological quality criteria** | | **Responses** | **Comments** |
| S1. | Are there clear research questions? | Yes |  |
| S2. | Do the collected data allow to address the research questions? | Yes |  |
| 4.1 | Is the sampling strategy relevant to address the research question? | No | The study utilised snowball and convenience sampling, which does not provide an equal chance of selection and introduces high selection bias for a study aiming to generalise to older adults in Taiwan. |
| 4.2 | Is the sample representative of the target population? | No | The demographic data show a significant skew (80.6% female) and geographic limitation (recruitment solely from community care stations in Hsinchu), contradicting the authors’ claim of representativeness. |
| 4.3 | Are the measurements appropriate? | Yes |  |
| 4.4 | Is the risk of nonresponse bias low? | Can’t tell | Although incomplete questionnaires were removed, the study does not report a response rate or provide a comparison between respondents and non-respondents to assess potential bias. |
| 4.5 | Is the statistical analysis appropriate to answer the research question? | Yes |  |

| [[27]](https://journals.sagepub.com/doi/10.1177/11795972241277292) Das Gupta A. Conceptualizing patient as an organization with the adoption of digital health. Biomed Eng Comput Biol. 2024;15:11795972241277292. Doi:10.1177/11795972241277292 | | | |
| --- | --- | --- | --- |
| **Methodological quality criteria** | | **Responses** | **Comments** |
| S1. | Are there clear research questions? | Yes |  |
| S2. | Do the collected data allow to address the research questions? | Yes |  |
| 4.1 | Is the sampling strategy relevant to address the research question? | Can’t tell | While the survey was sent to “mobile device users,” the study does not describe the specific selection or recruitment method used. |
| 4.2 | Is the sample representative of the target population? | Can’t tell | Although the paper provides respondent demographics, it does not explicitly define the target population in a way that allows for an assessment of whether the sample reflects it. |
| 4.3 | Are the measurements appropriate? | Yes |  |
| 4.4 | Is the risk of nonresponse bias low? | Can’t tell | The study does not report a response rate, reasons for nonresponse, or any statistical analysis to adjust for or assess potential bias. |
| 4.5 | Is the statistical analysis appropriate to answer the research question? | Yes |  |

| [[28]](https://www.researchgate.net/publication/338187344_Consumer_adoption_of_smartphone_fitness_apps_an_extended_UTAUT2_perspective) Dhiman N, Arora N, Dogra N, Gupta A. Consumer adoption of smartphone fitness apps: an extended UTAUT2 perspective. J Indian Bus Res. 2020;12(3):363-88. Doi:10.1108/JIBR-05-2018-0158 | | | |
| --- | --- | --- | --- |
| **Methodological quality criteria** | | **Responses** | **Comments** |
| S1. | Are there clear research questions? | Yes |  |
| S2. | Do the collected data allow to address the research questions? | Yes |  |
| 4.1 | Is the sampling strategy relevant to address the research question? | No | The study utilised purposive sampling targeting gym members, which is a non-probability method that does not provide an equal chance of selection and introduces selection bias regarding general consumer adoption. |
| 4.2 | Is the sample representative of the target population? | No | The recruitment was limited to gym members in three states of North India, and the authors explicitly admit the findings cannot be generalised beyond this context. |
| 4.3 | Are the measurements appropriate? | Yes |  |
| 4.4 | Is the risk of nonresponse bias low? | Yes |  |
| 4.5 | Is the statistical analysis appropriate to answer the research question? | Yes |  |

| [[29]](https://pharmacia.pensoft.net/article/108957/) Dimitrova M, Dimitrov R, Ahchiyska K, Nikolaeva M, Ganova M, Petrova G. Digital health in Bulgaria: imagination or possible reality? Pharmacia. 2023;70(3):649-55. doi:10.3897/pharmacia.70.e108957 | | | |
| --- | --- | --- | --- |
| **Methodological quality criteria** | | **Responses** | **Comments** |
| S1. | Are there clear research questions? | Yes |  |
| S2. | Do the collected data allow to address the research questions? | Yes |  |
| 4.1 | Is the sampling strategy relevant to address the research question? | Yes |  |
| 4.2 | Is the sample representative of the target population? | Can’t tell | While 380 respondents participated, the study does not define the characteristics or size of the total target population, preventing an assessment of representativeness. |
| 4.3 | Are the measurements appropriate? | Can’t tell | The authors used a 21-item questionnaire but did not report information regarding validity, reliability, or pilot testing. |
| 4.4 | Is the risk of nonresponse bias low? | Can’t tell | The survey was distributed online, and the authors do not report a response rate or provide a comparison between respondents and non-respondents to check for bias. |
| 4.5 | Is the statistical analysis appropriate to answer the research question? | Yes |  |
| ** Although the study claims a “two-step” approach, it does not meet MMAT criteria for mixed methods, as the policy analysis is limited to document review rather than an empirical qualitative component. The study is therefore classified as a quantitative descriptive study, with the policy review serving only as background context. | | | |

| [[30]](https://www.frontiersin.org/journals/public-health/articles/10.3389/fpubh.2023.1325031/full) Liu D, Son S, Cao J. The determinants of public acceptance of telemedicine apps: an innovation diffusion perspective. Front Public Health. 2023;11:1325031. Doi:10.3389/fpubh.2023.1325031 | | | |
| --- | --- | --- | --- |
| **Methodological quality criteria** | | **Responses** | **Comments** |
| S1. | Are there clear research questions? | Yes |  |
| S2. | Do the collected data allow to address the research questions? | Yes |  |
| 4.1 | Is the sampling strategy relevant to address the research question? | No | The study utilised intercept (convenience) sampling at transportation hubs, which is a non-probability method that does not provide an equal chance of selection and introduces high selection bias for a study aiming to generalise to a city’s population. |
| 4.2 | Is the sample representative of the target population? | No | The authors admit that the sample is predominantly aged 18–39 (78%) and fails to capture the full diversity of the population, particularly the older adult demographic. |
| 4.3 | Are the measurements appropriate? | Yes |  |
| 4.4 | Is the risk of nonresponse bias low? | Can’t tell | While a comparison of early versus late respondents showed no significant differences, the study did not report a total response rate (number approached vs. completed) to assess the extent of non-participation. |
| 4.5 | Is the statistical analysis appropriate to answer the research question? | Yes |  |

| [[31]](https://www.jmir.org/2021/2/e24767/) Elkefi S, Yu Z, Asan O. Online medical record nonuse among patients: data analysis study of the 2019 Health Information National Trends Survey. J Med Internet Res. 2021;23(2):e24767. Doi:10.2196/24767 | | | |
| --- | --- | --- | --- |
| **Methodological quality criteria** | | **Responses** | **Comments** |
| S1. | Are there clear research questions? | Yes |  |
| S2. | Do the collected data allow to address the research questions? | Yes |  |
| 4.1 | Is the sampling strategy relevant to address the research question? | Yes |  |
| 4.2 | Is the sample representative of the target population? | Yes |  |
| 4.3 | Are the measurements appropriate? | Yes |  |
| 4.4 | Is the risk of nonresponse bias low? | No | The reported response rate is 30.20%, and the authors explicitly acknowledge in the limitations that this low rate might raise bias concerns. |
| 4.5 | Is the statistical analysis appropriate to answer the research question? | Yes |  |

| [[33]](https://journals.healio.com/doi/10.3928/00220124-20241107-03?url_ver=Z39.88-2003&rfr_id=ori:rid:crossref.org&rfr_dat=cr_pub%20%200pubmed) Han J, Kim M, Ryu KH, Shin K. Acceptance of digital health care technology and the role of nursing education. J Contin Educ Nurs. 2024;55(12):575-87. Doi:10.3928/00220124-20241107-03 | | | |
| --- | --- | --- | --- |
| **Methodological quality criteria** | | **Responses** | **Comments** |
| S1. | Are there clear research questions? | Yes |  |
| S2. | Do the collected data allow to address the research questions? | Yes |  |
| 4.1 | Is the sampling strategy relevant to address the research question? | No | The study utilised convenience sampling, which the MMAT Manual notes does not provide an equal chance of selection, limiting the relevance for investigating acceptance among nurses generally. |
| 4.2 | Is the sample representative of the target population? | No | The participants were recruited from a single cancer specialty hospital, and the authors explicitly state in the limitations that this restricts generalizability to other healthcare institutions. |
| 4.3 | Are the measurements appropriate? | Yes |  |
| 4.4 | Is the risk of nonresponse bias low? | Can’t tell | Although the total number of collected surveys is reported, the study does not provide the number of invited participants or a response rate to allow for an assessment of bias. |
| 4.5 | Is the statistical analysis appropriate to answer the research question? | Yes |  |

| [[37]](https://journals.sagepub.com/doi/10.1177/20552076221126034?url_ver=Z39.88-2003&rfr_id=ori:rid:crossref.org&rfr_dat=cr_pub%20%200pubmed) Yang J, Luo B, Zhao C, Zhang H. Artificial intelligence healthcare service resources adoption by medical institutions based on TOE framework. Digit Health. 2022;8:20552076221126034. Doi:10.1177/20552076221126034 | | | |
| --- | --- | --- | --- |
| **Methodological quality criteria** | | **Responses** | **Comments** |
| S1. | Are there clear research questions? | Yes |  |
| S2. | Do the collected data allow to address the research questions? | Yes |  |
| 4.1 | Is the sampling strategy relevant to address the research question? | Yes |  |
| 4.2 | Is the sample representative of the target population? | Can’t tell | While the study lists the roles of the participating experts, it does not explicitly define the target population or state that these participants are representative of it. |
| 4.3 | Are the measurements appropriate? | Yes |  |
| 4.4 | Is the risk of nonresponse bias low? | Yes |  |
| 4.5 | Is the statistical analysis appropriate to answer the research question? | Yes |  |

| [[44]](https://www.liebertpub.com/doi/10.1089/tmj.2021.0418?url_ver=Z39.88-2003&rfr_id=ori%3Arid%3Acrossref.org&rfr_dat=cr_pub++0pubmed) Kim HM, Xu Y, Wang Y. Overcoming the mental health stigma through m-health apps: results from the Healthy Minds Study. Telemed J E Health. 2022;28(10):1534-40. Doi:10.1089/tmj.2021.0418 | | | |
| --- | --- | --- | --- |
| **Methodological quality criteria** | | **Responses** | **Comments** |
| S1. | Are there clear research questions? | Yes |  |
| S2. | Do the collected data allow to address the research questions? | Yes |  |
| 4.1 | Is the sampling strategy relevant to address the research question? | Yes |  |
| 4.2 | Is the sample representative of the target population? | No | The authors explicitly state that the sample is not nationally representative of all schools or students in the United States. |
| 4.3 | Are the measurements appropriate? | Can’t tell | While constructs are defined, the study relies on single-item measures without explicitly reporting validity or reliability evidence for each measure. |
| 4.4 | Is the risk of nonresponse bias low? | Can’t tell | The study reports analytic inclusion criteria but does not provide response rates, reasons for nonresponse, or adjustments to assess potential bias. |
| 4.5 | Is the statistical analysis appropriate to answer the research question? | Yes |  |

| [[45]](https://www.jmir.org/2023/1/e41915) Kim S, Chow BC, Park S, Liu H. The usage of digital health technology among older adults in Hong Kong and the role of technology readiness and eHealth literacy: path analysis. J Med Internet Res. 2023;25:e41915. Doi:10.2196/41915 | | | |
| --- | --- | --- | --- |
| **Methodological quality criteria** | | **Responses** | **Comments** |
| S1. | Are there clear research questions? | Yes |  |
| S2. | Do the collected data allow to address the research questions? | Yes |  |
| 4.1 | Is the sampling strategy relevant to address the research question? | No | Although the study aimed to assess usage among older adults, it utilised a web-based convenience sampling method, which systematically excludes non-internet users and fails to cover the target population adequately. |
| 4.2 | Is the sample representative of the target population? | No | The authors explicitly state in the limitations that the web-based recruitment excluded older adults less willing to interact with IT, hindering generalizability. |
| 4.3 | Are the measurements appropriate? | Yes |  |
| 4.4 | Is the risk of nonresponse bias low? | Can’t tell | Although the study reports the number of usable responses, it does not provide a response rate (invitations versus approached) or reasons for non-participation to assess potential bias. |
| 4.5 | Is the statistical analysis appropriate to answer the research question? | Yes |  |

| [[49]](https://www.frontiersin.org/journals/public-health/articles/10.3389/fpubh.2025.1547189/full) Larnyo E, Tettegah S, Nutakor JA, Addai-Dansoh S, Arboh F. Technology access, use, socioeconomic status, and healthcare disparities among African Americans in the US. Front Public Health. 2025;13:1547189. Doi:10.3389/fpubh.2025.1547189 | | | |
| --- | --- | --- | --- |
| **Methodological quality criteria** | | **Responses** | **Comments** |
| S1. | Are there clear research questions? | Yes |  |
| S2. | Do the collected data allow to address the research questions? | Yes |  |
| 4.1 | Is the sampling strategy relevant to address the research question? | Yes |  |
| 4.2 | Is the sample representative of the target population? | Yes |  |
| 4.3 | Are the measurements appropriate? | Yes |  |
| 4.4 | Is the risk of nonresponse bias low? | Can’t tell | The risk of nonresponse bias cannot be determined; although a modified Dillman approach was used, the study does not report a specific response rate for the subsample or compare respondents to non-respondents to assess potential bias. |
| 4.5 | Is the statistical analysis appropriate to answer the research question? | Yes |  |

| [[58]](https://www.mdpi.com/2227-9032/13/11/1222) Marinescu ȘA, Oncioiu I, Ghibanu A-I. The digital transformation of healthcare through intelligent technologies: a path dependence-augmented–unified theory of acceptance and use of technology model for clinical decision support systems. Healthcare (Basel). 2025;13(11):1222. Doi:10.3390/healthcare13111222 | | | |
| --- | --- | --- | --- |
| **Methodological quality criteria** | | **Responses** | **Comments** |
| S1. | Are there clear research questions? | Yes |  |
| S2. | Do the collected data allow to address the research questions? | Yes |  |
| 4.1 | Is the sampling strategy relevant to address the research question? | Can’t tell | The article claims to use both “random stratified sampling” and “snowball sampling.” As noted in the MMAT Manual, non-probability sampling (snowball) does not provide an equal chance of selection. |
| 4.2 | Is the sample representative of the target population? | Can’t tell | The authors acknowledge the sample is exclusively from Romania and may favour those familiar with AI, and no data comparing the sample to the broader target population are provided to demonstrate representativeness. |
| 4.3 | Are the measurements appropriate? | Yes |  |
| 4.4 | Is the risk of nonresponse bias low? | Yes |  |
| 4.5 | Is the statistical analysis appropriate to answer the research question? | Yes |  |

| [[79]](https://www.liebertpub.com/doi/10.1089/tmj.2022.0527?url_ver=Z39.88-2003&rfr_id=ori%3Arid%3Acrossref.org&rfr_dat=cr_pub++0pubmed) Almathami HKY, Win KT, Vlahu-Gjorgievska E. Empirical evidence of internal and external factors influencing users’ motivation toward teleconsultation use. Telemed J E Health. 2024;30(1):141-56. doi:10.1089/tmj.2022.0527 | | | |
| --- | --- | --- | --- |
| **Methodological quality criteria** | | **Responses** | **Comments** |
| S1. | Are there clear research questions? | Yes |  |
| S2. | Do the collected data allow to address the research questions? | Yes |  |
| 4.1 | Is the sampling strategy relevant to address the research question? | Can’t tell | The study utilised a non-random sampling technique via social media without explicitly justifying why this strategy is appropriate for the stated aim or addressing the selection bias inherent in non-probability sampling. |
| 4.2 | Is the sample representative of the target population? | Can’t tell | The recruitment was voluntary via social media, potentially excluding those without access, and the authors do not explicitly claim or provide data to demonstrate representativeness. |
| 4.3 | Are the measurements appropriate? | Can’t tell | While the study mentions a modified eDelphi study for validation and reports Cronbach’s alpha, it does not explicitly report specific validity and reliability evidence for each measure beyond general statements. |
| 4.4 | Is the risk of nonresponse bias low? | Can’t tell | The study does not report a response rate, reasons for nonresponse, or any adjustments to assess potential bias. |
| 4.5 | Is the statistical analysis appropriate to answer the research question? | Can’t tell | Although the use of SPSS and SmartPLS is described, the study does not explicitly justify why these specific analyses are appropriate to address the research objectives. |

# **Mixed Methods**

| [[1]](https://formative.jmir.org/2021/2/e19074/) Freise L, Neves AL, Flott K, Harrison P, Kelly J, Darzi A, et al. Assessment of patients’ ability to review electronic health record information to identify potential errors: cross-sectional web-based survey. JMIR Form Res. 2021;5(2):e19074. doi:10.2196/19074 | | | |
| --- | --- | --- | --- |
| **Methodological quality criteria** | | **Responses** | **Comments** |
| S1. | Are there clear research questions? | Yes |  |
| S2. | Do the collected data allow to address the research questions? | Yes |  |
| 1.1 | Is the qualitative approach appropriate to answer the research question? | Yes |  |
| 1.2 | Are the qualitative data collection methods adequate to address the research question? | Yes |  |
| 1.3 | Are the findings adequately derived from data? | Yes |  |
| 1.4 | Is the interpretation of result sufficiently substantiated by data? | Yes |  |
| 1.5 | Is there coherence between qualitative data sources, collection, analysis and interpretation? | Yes |  |
| 4.1 | Is the sampling strategy relevant to address the research question? | Yes |  |
| 4.2 | Is the sample representative of the target population? | Can’t tell | The response rate was 23.5% (210/682). The authors admit demographic data were limited to "keep the survey short," making it impossible to confirm if respondents represent the wider user base. |
| 4.3 | Are the measurements appropriate? | Yes |  |
| 4.4 | Is the risk of nonresponse bias low? | Can’t tell | With a low response rate (23.5%) and no comparison of non-respondents (due to lack of demographic data), the risk of bias cannot be assessed or ruled out. |
| 4.5 | Is the statistical analysis appropriate to answer the research question? | Yes |  |
| 5.1 | Is there an adequate rationale for using a mixed methods design to address the research question? | Yes |  |
| 5.2 | Are the different components of the study effectively integrated to answer the research question? | Can’t tell | While the article notes that qualitative responses supplemented structured metrics, it does not explicitly describe a specific integration procedure, such as a joint display or an explicit mixing approach. |
| 5.3 | Are the outputs of the integration of the quantitative and qualitative components adequately interpreted? | Can’t tell | Although the results present quantitative proportions and qualitative themes with quotes, the article does not present explicit "outputs of integration" such as meta-inferences. |
| 5.4 | Are divergence and inconsistencies between quantitative and qualitative results adequately addressed? | Yes |  |
| 5.5 | Do the different components of the study adhere to the quality criteria of each tradition of the methods involved? | Can’t tell | While the qualitative component reports rigorous methods (e.g., framework analysis, saturation), the quantitative component lacks explicit reporting of quality assurance aligned to quantitative descriptive standards. |
| ** Although the study is primarily a quantitative cross-sectional survey, it meets MMAT criteria for mixed methods due to the inclusion of open-ended questions that were systematically coded and analysed using primary qualitative data. The integration of these qualitative findings with the quantitative results supports classification as a mixed-methods study with a quantitatively dominant design. | | | |

| [[16]](https://bmchealthservres.biomedcentral.com/articles/10.1186/s12913-023-10368-z) Anttila M, Koivisto J, Luoma ML, et al. How to adopt technologies in home care: a mixed methods study on user experiences and change of home care in Finland. BMC Health Serv Res. 2023;23:1342. doi:10.1186/s12913-023-10368-z | | | |
| --- | --- | --- | --- |
| **Methodological quality criteria** | | **Responses** | **Comments** |
| S1. | Are there clear research questions? | Yes |  |
| S2. | Do the collected data allow to address the research questions? | Yes |  |
| 1.1 | Is the qualitative approach appropriate to answer the research question? | Yes |  |
| 1.2 | Are the qualitative data collection methods adequate to address the research question? | Yes |  |
| 1.3 | Are the findings adequately derived from data? | Yes |  |
| 1.4 | Is the interpretation of result sufficiently substantiated by data? | No | The results section summarizes themes but does not provide any verbatim participant quotations to substantiate these interpretations. |
| 1.5 | Is there coherence between qualitative data sources, collection, analysis and interpretation? | Yes |  |
| 4.1 | Is the sampling strategy relevant to address the research question? | Yes |  |
| 4.2 | Is the sample representative of the target population? | No | The study used "convenience sampling". With only ~90 respondents out of 4,500 professionals and admitted difficulties in reaching participants, representativeness is not established. |
| 4.3 | Are the measurements appropriate? | No | The authors explicitly state in the limitations that "team survey questions were not pilot-tested beforehand". |
| 4.4 | Is the risk of nonresponse bias low? | Can’t tell | The study does not report a response rate relative to the total number invited, nor does it compare respondents to non-respondents. |
| 4.5 | Is the statistical analysis appropriate to answer the research question? | Yes |  |
| 5.1 | Is there an adequate rationale for using a mixed methods design to address the research question? | Yes |  |
| 5.2 | Are the different components of the study effectively integrated to answer the research question? | Yes |  |
| 5.3 | Are the outputs of the integration of the quantitative and qualitative components adequately interpreted? | Yes |  |
| 5.4 | Are divergence and inconsistencies between quantitative and qualitative results adequately addressed? | Yes |  |
| 5.5 | Do the different components of the study adhere to the quality criteria of each tradition of the methods involved? | Can’t tell | The qualitative component lacks direct participant quotations, and the quantitative component relies on a small convenience sample with non-pilot-tested questions, indicating potential weaknesses in representativeness and measurement validity. |

| [[18]](https://sigmapubs.onlinelibrary.wiley.com/doi/10.1111/jnu.12619) Aure CF, Kluge A, Moen A. Older adults’ engagement in technology-mediated self-monitoring of diet: a mixed-method study. J Nurs Scholarsh. 2021;53(1):25-34. doi:10.1111/jnu.12619 | | | |
| --- | --- | --- | --- |
| **Methodological quality criteria** | | **Responses** | **Comments** |
| S1. | Are there clear research questions? | Yes |  |
| S2. | Do the collected data allow to address the research questions? | Yes |  |
| 1.1 | Is the qualitative approach appropriate to answer the research question? | Yes |  |
| 1.2 | Are the qualitative data collection methods adequate to address the research question? | Yes |  |
| 1.3 | Are the findings adequately derived from data? | Yes |  |
| 1.4 | Is the interpretation of result sufficiently substantiated by data? | Yes |  |
| 1.5 | Is there coherence between qualitative data sources, collection, analysis and interpretation? | Yes |  |
| 4.1 | Is the sampling strategy relevant to address the research question? | Yes |  |
| 4.2 | Is the sample representative of the target population? | No | The sample size is small ($n=25$) and derived from "convenience sampling" (professionals approached those they "anticipated would benefit"). The authors explicitly state the participants "do not represent the full spectrum". |
| 4.3 | Are the measurements appropriate? | Yes |  |
| 4.4 | Is the risk of nonresponse bias low? | No | There was significant attrition/non-participation. Of 39 recruited, 14 withdrew (36% attrition). No analysis compares the characteristics of those who withdrew vs. those who completed. |
| 4.5 | Is the statistical analysis appropriate to answer the research question? | Yes |  |
| 5.1 | Is there an adequate rationale for using a mixed methods design to address the research question? | Yes |  |
| 5.2 | Are the different components of the study effectively integrated to answer the research question? | Yes |  |
| 5.3 | Are the outputs of the integration of the quantitative and qualitative components adequately interpreted? | Yes |  |
| 5.4 | Are divergence and inconsistencies between quantitative and qualitative results adequately addressed? | Yes |  |
| 5.5 | Do the different components of the study adhere to the quality criteria of each tradition of the methods involved? | Can’t tell | While the qualitative component appears rigorous, the quantitative component suffers from high attrition (36%) and a small, non-representative sample, creating a high risk of selection bias. |
| [[19]](https://onlinelibrary.wiley.com/doi/10.1111/jep.13923) Bagot KL, Bladin CF, Vu M, Bernard S, Smith K, Hocking G, et al. Factors influencing the successful implementation of a novel digital health application to streamline multidisciplinary communication across multiple organisations for emergency care. J Eval Clin Pract. 2024;30(2):184-98. doi:10.1111/jep.13923 | | | |
| **Methodological quality criteria** | | **Responses** | **Comments** |
| S1. | Are there clear research questions? | Yes |  |
| S2. | Do the collected data allow to address the research questions? | Yes |  |
| 1.1 | Is the qualitative approach appropriate to answer the research question? | Yes |  |
| 1.2 | Are the qualitative data collection methods adequate to address the research question? | Yes |  |
| 1.3 | Are the findings adequately derived from data? | Yes |  |
| 1.4 | Is the interpretation of result sufficiently substantiated by data? | Yes |  |
| 1.5 | Is there coherence between qualitative data sources, collection, analysis and interpretation? | Yes |  |
| 4.1 | Is the sampling strategy relevant to address the research question? | Yes |  |
| 4.2 | Is the sample representative of the target population? | Can’t tell | The total size of the eligible target population at these sites is not clearly reported, representativeness cannot be confirmed. |
| 4.3 | Are the measurements appropriate? | Yes |  |
| 4.4 | Is the risk of nonresponse bias low? | Can’t tell | The study does not report a response rate (surveys were voluntary at training sessions), nor does it compare respondents to non-respondents. |
| 4.5 | Is the statistical analysis appropriate to answer the research question? | Yes |  |
| 5.1 | Is there an adequate rationale for using a mixed methods design to address the research question? | Yes |  |
| 5.2 | Are the different components of the study effectively integrated to answer the research question? | Yes |  |
| 5.3 | Are the outputs of the integration of the quantitative and qualitative components adequately interpreted? | Yes |  |
| 5.4 | Are divergence and inconsistencies between quantitative and qualitative results adequately addressed? | Yes |  |
| 5.5 | Do the different components of the study adhere to the quality criteria of each tradition of the methods involved? | Can’t tell | While the qualitative component appears structured, the quantitative component lacks information to assess nonresponse bias or representativeness, limiting the overall quality assessment. |

| [[35]](https://dl.acm.org/doi/10.1145/3635472) Jardine J, Nadal C, Robinson S, Enrique A, Hanratty M, Doherty G. Between rhetoric and reality: real-world barriers to uptake and early engagement in digital mental health interventions. ACM Trans Comput Hum Interact. 2024;31(2):27. doi:10.1145/3635472 | | | |
| --- | --- | --- | --- |
| **Methodological quality criteria** | | **Responses** | **Comments** |
| S1. | Are there clear research questions? | Yes |  |
| S2. | Do the collected data allow to address the research questions? | Yes |  |
| 1.1 | Is the qualitative approach appropriate to answer the research question? | Yes |  |
| 1.2 | Are the qualitative data collection methods adequate to address the research question? | Yes |  |
| 1.3 | Are the findings adequately derived from data? | Yes |  |
| 1.4 | Is the interpretation of result sufficiently substantiated by data? | Yes |  |
| 1.5 | Is there coherence between qualitative data sources, collection, analysis and interpretation? | Yes |  |
| 4.1 | Is the sampling strategy relevant to address the research question? | Yes |  |
| 4.2 | Is the sample representative of the target population? | No | The authors explicitly acknowledge a "skew towards white, educated women" and note that the sample may not represent more diverse groups. |
| 4.3 | Are the measurements appropriate? | No | The questionnaire was "non-standardized". While based on literature/HITAM, it was not pilot-tested or validated. |
| 4.4 | Is the risk of nonresponse bias low? | No | The authors report a "very low response rate" for the email recruitment and acknowledge "inevitable self-selection response bias", indicating a high risk of bias. |
| 4.5 | Is the statistical analysis appropriate to answer the research question? | Yes |  |
| 5.1 | Is there an adequate rationale for using a mixed methods design to address the research question? | Yes |  |
| 5.2 | Are the different components of the study effectively integrated to answer the research question? | Yes |  |
| 5.3 | Are the outputs of the integration of the quantitative and qualitative components adequately interpreted? | Yes |  |
| 5.4 | Are divergence and inconsistencies between quantitative and qualitative results adequately addressed? | Yes |  |
| 5.5 | Do the different components of the study adhere to the quality criteria of each tradition of the methods involved? | Can’t tell | While the qualitative component is rigorous (reflexive thematic analysis), the quantitative component is weak due to a non-standardised questionnaire, very low response rate, and self-selection bias. |

| [[41]](https://journals.sagepub.com/doi/10.1177/20552076221150745) Keogh A, Alcock L, Brown P, Buckley E, Brozgol M, Gazit E, et al. Acceptability of wearable devices for measuring mobility remotely: observations from the Mobilise-D technical validation study. Digit Health. 2023;9:20552076221150745. doi:10.1177/20552076221150745 | | | |
| --- | --- | --- | --- |
| **Methodological quality criteria** | | **Responses** | **Comments** |
| S1. | Are there clear research questions? | Yes |  |
| S2. | Do the collected data allow to address the research questions? | Yes |  |
| 1.1 | Is the qualitative approach appropriate to answer the research question? | Yes |  |
| 1.2 | Are the qualitative data collection methods adequate to address the research question? | Yes |  |
| 1.3 | Are the findings adequately derived from data? | Yes |  |
| 1.4 | Is the interpretation of result sufficiently substantiated by data? | Yes |  |
| 1.5 | Is there coherence between qualitative data sources, collection, analysis and interpretation? | Yes |  |
| 4.1 | Is the sampling strategy relevant to address the research question? | Yes |  |
| 4.2 | Is the sample representative of the target population? | Yes |  |
| 4.3 | Are the measurements appropriate? | Yes |  |
| 4.4 | Is the risk of nonresponse bias low? | Yes |  |
| 4.5 | Is the statistical analysis appropriate to answer the research question? | Yes |  |
| 5.1 | Is there an adequate rationale for using a mixed methods design to address the research question? | Yes |  |
| 5.2 | Are the different components of the study effectively integrated to answer the research question? | Yes |  |
| 5.3 | Are the outputs of the integration of the quantitative and qualitative components adequately interpreted? | Yes |  |
| 5.4 | Are divergence and inconsistencies between quantitative and qualitative results adequately addressed? | Yes |  |
| 5.5 | Do the different components of the study adhere to the quality criteria of each tradition of the methods involved? | Yes |  |

| [[42]](https://www.jmir.org/2023/1/e44131) Kerr JI, Naegelin M, Benk M, von Wangenheim F, Meins E, Viganò E, et al. Investigating employees’ concerns and wishes regarding digital stress management interventions with value sensitive design: mixed methods study. J Med Internet Res. 2023;25:e44131. doi:10.2196/44131 | | | |
| --- | --- | --- | --- |
| **Methodological quality criteria** | | **Responses** | **Comments** |
| S1. | Are there clear research questions? | Yes |  |
| S2. | Do the collected data allow to address the research questions? | Yes |  |
| 1.1 | Is the qualitative approach appropriate to answer the research question? | Yes |  |
| 1.2 | Are the qualitative data collection methods adequate to address the research question? | Yes |  |
| 1.3 | Are the findings adequately derived from data? | Yes |  |
| 1.4 | Is the interpretation of result sufficiently substantiated by data? | Yes |  |
| 1.5 | Is there coherence between qualitative data sources, collection, analysis and interpretation? | Yes |  |
| 4.1 | Is the sampling strategy relevant to address the research question? | Yes |  |
| 4.2 | Is the sample representative of the target population? | No | The authors explicitly acknowledge a "self-selection bias" (recruiting "technology-affine individuals") and note that the sample does not fully match the company demographics (e.g., fewer managers). |
| 4.3 | Are the measurements appropriate? | Can’t tell | The survey used "self-developed scales". While a pre-test and CFA were conducted, the authors explicitly state as a limitation that "more thorough testing is needed to validate these scales". |
| 4.4 | Is the risk of nonresponse bias low? | Can’t tell | The response rate relative to the total number of employees invited is not reported. Furthermore, 25.7% of respondents were excluded for failing manipulation checks, which introduces potential bias. |
| 4.5 | Is the statistical analysis appropriate to answer the research question? | Yes |  |
| 5.1 | Is there an adequate rationale for using a mixed methods design to address the research question? | Yes |  |
| 5.2 | Are the different components of the study effectively integrated to answer the research question? | Yes |  |
| 5.3 | Are the outputs of the integration of the quantitative and qualitative components adequately interpreted? | Yes |  |
| 5.4 | Are divergence and inconsistencies between quantitative and qualitative results adequately addressed? | Yes |  |
| 5.5 | Do the different components of the study adhere to the quality criteria of each tradition of the methods involved? | Can’t tell | While the qualitative component appears rigorous, the quantitative component relies on self-developed scales and excluded a significant portion (25.7%) of participants, raising concerns about measurement validity and selection bias. |

| [[48]](https://journals.sagepub.com/doi/10.1177/11786329241284401?url_ver=Z39.88-2003&rfr_id=ori:rid:crossref.org&rfr_dat=cr_pub%20%200pubmed) Kitole FA, Shukla S. Cloud horizons: strengthening rural healthcare through telemedicine’s digital canopy. Health Serv Insights. 2024;17:11786329241284401. doi:10.1177/11786329241284401 | | | |
| --- | --- | --- | --- |
| **Methodological quality criteria** | | **Responses** | **Comments** |
| S1. | Are there clear research questions? | Yes |  |
| S2. | Do the collected data allow to address the research questions? | Yes |  |
| 1.1 | Is the qualitative approach appropriate to answer the research question? | Yes |  |
| 1.2 | Are the qualitative data collection methods adequate to address the research question? | Yes |  |
| 1.3 | Are the findings adequately derived from data? | Yes |  |
| 1.4 | Is the interpretation of result sufficiently substantiated by data? | Yes |  |
| 1.5 | Is there coherence between qualitative data sources, collection, analysis and interpretation? | Yes |  |
| 4.1 | Is the sampling strategy relevant to address the research question? | Yes |  |
| 4.2 | Is the sample representative of the target population? | No | The facilities were "purposively selected based on accessibility," and participants were selected based on "experience". This non-probability sampling limits representativeness of the broader rural population. |
| 4.3 | Are the measurements appropriate? | No | The authors explicitly state in the limitations section that there was a "lack of pilot testing" and a "reliance on self-reported data" without mention of validation. |
| 4.4 | Is the risk of nonresponse bias low? | Yes |  |
| 4.5 | Is the statistical analysis appropriate to answer the research question? | Yes |  |
| 5.1 | Is there an adequate rationale for using a mixed methods design to address the research question? | Yes |  |
| 5.2 | Are the different components of the study effectively integrated to answer the research question? | Yes |  |
| 5.3 | Are the outputs of the integration of the quantitative and qualitative components adequately interpreted? | Yes |  |
| 5.4 | Are divergence and inconsistencies between quantitative and qualitative results adequately addressed? | Yes |  |
| 5.5 | Do the different components of the study adhere to the quality criteria of each tradition of the methods involved? | Can’t tell | While the qualitative component appears rigorous, the quantitative component lacks pilot testing or reported validation of the instrument, creating a major limitation in measurement appropriateness. |

| [[50]](https://games.jmir.org/2024/1/e47600) Lee EWJ, Tan WW, Pham BTP, Kawaja A, Theng YL. Addressing data absenteeism and technology chauvinism in the use of gamified wearable gloves among older adults: moderated usability study. JMIR Serious Games. 2024;12:e47600. doi:10.2196/47600 | | | |
| --- | --- | --- | --- |
| **Methodological quality criteria** | | **Responses** | **Comments** |
| S1. | Are there clear research questions? | Yes |  |
| S2. | Do the collected data allow to address the research questions? | Yes |  |
| 1.1 | Is the qualitative approach appropriate to answer the research question? | Yes |  |
| 1.2 | Are the qualitative data collection methods adequate to address the research question? | Yes |  |
| 1.3 | Are the findings adequately derived from data? | Yes |  |
| 1.4 | Is the interpretation of result sufficiently substantiated by data? | Yes |  |
| 1.5 | Is there coherence between qualitative data sources, collection, analysis and interpretation? | Yes |  |
| 4.1 | Is the sampling strategy relevant to address the research question? | Yes |  |
| 4.2 | Is the sample representative of the target population? | No | The sample size is very small (N=19) and based on convenience. The authors explicitly state the findings are "educated guesses" and the participants may be "qualitatively different" from the wider population. |
| 4.3 | Are the measurements appropriate? | Yes |  |
| 4.4 | Is the risk of nonresponse bias low? | Can’t tell | There is no information reported regarding how many individuals were approached versus how many agreed to participate, nor any comparison with non-respondents. |
| 4.5 | Is the statistical analysis appropriate to answer the research question? | Yes |  |
| 5.1 | Is there an adequate rationale for using a mixed methods design to address the research question? | Can’t tell | Although the study reports both descriptive and thematic analyses, it does not explicitly state a rationale for mixing methods as a specific design choice. |
| 5.2 | Are the different components of the study effectively integrated to answer the research question? | Yes |  |
| 5.3 | Are the outputs of the integration of the quantitative and qualitative components adequately interpreted? | Yes |  |
| 5.4 | Are divergence and inconsistencies between quantitative and qualitative results adequately addressed? | Yes |  |
| 5.5 | Do the different components of the study adhere to the quality criteria of each tradition of the methods involved? | Can’t tell | While the qualitative component is high quality, the quantitative component is low quality due to a very small sample size (N=19) and acknowledged lack of representativeness, and the overall quality cannot exceed the weakest component. |

| [[54]](https://www.mdpi.com/2227-9067/11/10/1215) Linder L, Utendorfer H, Oliveros B, Gilliland S, Tiase VL, Altizer R. Usability evaluation of the revised Color Me Healthy symptom assessment app: perspectives of children and parents. Children (Basel). 2024;11(10):1215. doi:10.3390/children11101215 | | | |
| --- | --- | --- | --- |
| **Methodological quality criteria** | | **Responses** | **Comments** |
| S1. | Are there clear research questions? | Yes |  |
| S2. | Do the collected data allow to address the research questions? | Yes |  |
| 1.1 | Is the qualitative approach appropriate to answer the research question? | Yes |  |
| 1.2 | Are the qualitative data collection methods adequate to address the research question? | Yes |  |
| 1.3 | Are the findings adequately derived from data? | Yes |  |
| 1.4 | Is the interpretation of result sufficiently substantiated by data? | Yes |  |
| 1.5 | Is there coherence between qualitative data sources, collection, analysis and interpretation? | Yes |  |
| 4.1 | Is the sampling strategy relevant to address the research question? | Yes |  |
| 4.2 | Is the sample representative of the target population? | No | While adequate for usability engineering (identifying bugs), a sample of N=14 is not statistically representative of the target population for a descriptive study. The authors acknowledge limited racial/ethnic diversity and small sample size as limitations. |
| 4.3 | Are the measurements appropriate? | Yes |  |
| 4.4 | Is the risk of nonresponse bias low? | Can’t tell | Although attrition was low (1 withdrawal, 1 decline), the study does not report the total number of eligible families screened or approached via the "waiver of authorization", making it difficult to assess total nonresponse bias. |
| 4.5 | Is the statistical analysis appropriate to answer the research question? | Yes |  |
| 5.1 | Is there an adequate rationale for using a mixed methods design to address the research question? | Yes |  |
| 5.2 | Are the different components of the study effectively integrated to answer the research question? | Yes |  |
| 5.3 | Are the outputs of the integration of the quantitative and qualitative components adequately interpreted? | Yes |  |
| 5.4 | Are divergence and inconsistencies between quantitative and qualitative results adequately addressed? | Yes |  |
| 5.5 | Do the different components of the study adhere to the quality criteria of each tradition of the methods involved? | Can’t tell | While the qualitative component is high quality (rigorous content analysis), the quantitative component relies on a very small sample (N=14) with limited diversity, limiting representativeness. |
| [[62]](https://journals.sagepub.com/doi/10.1177/20552076241247196?url_ver=Z39.88-2003&rfr_id=ori:rid:crossref.org&rfr_dat=cr_pub%20%200pubmed) Moody L, Wood E, Needham A, Booth A, Tindale W. Exploring how the design and provision of digital self-management technology can improve the uptake by older adults with chronic kidney disease, diabetes and dementia: a modified e-Delphi study. Digital Health. 2024;10:20552076241247196. doi:10.1177/20552076241247196 | | | |
| **Methodological quality criteria** | | **Responses** | **Comments** |
| S1. | Are there clear research questions? | Yes |  |
| S2. | Do the collected data allow to address the research questions? | Yes |  |
| 1.1 | Is the qualitative approach appropriate to answer the research question? | Yes |  |
| 1.2 | Are the qualitative data collection methods adequate to address the research question? | Yes |  |
| 1.3 | Are the findings adequately derived from data? | Yes |  |
| 1.4 | Is the interpretation of result sufficiently substantiated by data? | Yes |  |
| 1.5 | Is there coherence between qualitative data sources, collection, analysis and interpretation? | Yes |  |
| 4.1 | Is the sampling strategy relevant to address the research question? | Yes |  |
| 4.2 | Is the sample representative of the target population? | No | The authors explicitly acknowledge that the sample size (N=14) was small and "limit[ed] our ability to analyse the data per participant group," and that "ideally, the panel would have included... at least one panel member with lived experience of dementia". Representativeness of the expert pool is limited. |
| 4.3 | Are the measurements appropriate? | Yes |  |
| 4.4 | Is the risk of nonresponse bias low? | Can’t tell | There was attrition: 14 participants in Round 1, 13 in Round 2, and 11 in Round 3 (78% retention). The study does not compare completers vs non-completers to rule out bias. |
| 4.5 | Is the statistical analysis appropriate to answer the research question? | Yes |  |
| 5.1 | Is there an adequate rationale for using a mixed methods design to address the research question? | Yes |  |
| 5.2 | Are the different components of the study effectively integrated to answer the research question? | Yes |  |
| 5.3 | Are the outputs of the integration of the quantitative and qualitative components adequately interpreted? | Yes |  |
| 5.4 | Are divergence and inconsistencies between quantitative and qualitative results adequately addressed? | Yes |  |
| 5.5 | Do the different components of the study adhere to the quality criteria of each tradition of the methods involved? | Can’t tell | While procedures for analysis and consensus are reported, the article lacks sufficient explicit detail to judge whether all quality expectations for the qualitative and quantitative traditions were fully met. |
