# Supplementary material for: Advancing foundational models in digital health technology adoption: A systematic literature review of multidisciplinary factors
Source: PLOS Digit Health. 2026 Sep 2;5(9):e0001690. doi: 10.1371/journal.pdig.0001690 (PMC13537594; doi:10.1371/journal.pdig.0001690)
Supplement: Supplementary file 12 [file pdig.0001690.s009.docx]

Reviewer’s comment

This paper systematically reviews the multidisciplinary factors influencing the adoption and sustained use of digital health technologies (DHTs) by analyzing 82 studies published between 2020 and 2025. The study holds certain value for the future application of DHTs in the field of public health. It is recommended to revise the paper to further enhance its quality.

1. It is suggested to provide a more detailed explanation of the specific operational procedures for inclusion and exclusion criteria in the methods section, such as how multilingual literature and grey literature were handled, to enhance the transparency and reproducibility of the study.

2. The paper mentions the use of the SPIDER framework, but the description of the mapping relationship between SPIDER and PICOS during the actual screening and data extraction process is somewhat brief. It is recommended to supplement a specific mapping table and application examples.

3. The paper frequently refers to "multidisciplinary factors," but what are the specific roles and weights of psychological, social, and environmental factors in the adoption of DHTs? It is suggested to supplement the above data, as similar content does not seem to be present in the paper.

4. It is recommended to provide additional details on coding and other data governance aspects in the data methodology section, as this will impact the replicability of the study.

5. How can DHTs better ensure data privacy, especially in cases involving AI? It is suggested to discuss this in the discussion and limitation sections. I believe there may be significant differences across regions of the world. 6. The limitations section mentions the insufficiency of sample size and cross-sectional studies, but the suggestions for future research directions are relatively general. It is recommended to propose more actionable suggestions, such as longitudinal studies and intervention studies.
