# Supplementary material for: Advancing foundational models in digital health technology adoption: A systematic literature review of multidisciplinary factors
Source: PLOS Digit Health. 2026 Sep 2;5(9):e0001690. doi: 10.1371/journal.pdig.0001690 (PMC13537594; doi:10.1371/journal.pdig.0001690)
Supplement: Supplementary file 13 [file pdig.0001690.s012.docx]

**Response to Reviewers**

**Manuscript number:** PDIG-D-25-00816

**Manuscript Title:** Advancing foundational models in digital health technology adoption: A systematic literature review of multidisciplinary factors

We sincerely thank the Editor and the Reviewers for their constructive and insightful comments. We have carefully revised the manuscript in response to each point raised. Below, we provide a detailed point-by-point response.

| **Comment** | **Response** | **Page number** |
| --- | --- | --- |
| PROSPERO Registration and Protocol Fidelity  I have carefully reviewed your PROSPERO registration (CRD420251056883, provided as S1 Protocol). The registration explicitly states in the "Additional Information" section that "the findings will be contextualized and critically evaluated for practicability and relevance to Malaysia's healthcare management." However, the submitted manuscript provides only one brief sentence mentioning Malaysian policies (lines 389-391), with no substantive contextualization, transferability analysis, or Malaysia-specific recommendations.  This represents a fundamental disconnect between what was registered and what has been delivered. The manuscript reads as a global review with a tokenistic reference to Malaysia, rather than a review with findings "critically evaluated" for the Malaysian context as promised.  You must address this in one of three ways:  Option A: Substantially revise the manuscript to fulfill the protocol commitment by adding dedicated sections analyzing how each theme applies to Malaysia's healthcare system, what adaptations are needed, and specific recommendations for Malaysian policymakers (MADANI framework, Health White Paper, Digital Economy Blueprint).  Option B: Submit an amendment to PROSPERO removing the Malaysian focus, acknowledge this as a major protocol deviation in the manuscript, and reframe the work as a global review with Malaysia as one example context.  Option C: Acknowledge that the current manuscript does not align with the registered protocol and withdraw for resubmission after conducting the promised Malaysian contextualization.  Additional timing concern: Your protocol indicates work began 23 March 2025 but PROSPERO registration occurred 26 May 2025. This two-month gap means some methodological decisions were made before prospective registration, which reduces the value of registration for preventing bias. While I understand pragmatic constraints, this should be acknowledged as a limitation. | - The manuscript scope was revised from a Malaysia-specific contextual analysis to a global systematic review. **All Malaysia-specific framing and policy references were removed or reframed**. - The PROSPERO registration (CRD420251056883) was amended to remove the stated intention to contextualise findings for Malaysia’s healthcare management, **ensuring consistency with the global evidence base**. - **A protocol amendment and scope-clarification statement was added to explicitly acknowledge and justify the deviation from the original registered protocol**. - Discussion points were revised to ensure implications are framed as globally transferable rather than country-specific. - Clarification was added to acknowledge that protocol development began prior to PROSPERO registration, and this has been explicitly stated as a limitation. - A statement was added clarifying that no study screening, data extraction, risk-of-bias assessment, or data synthesis was conducted before PROSPERO registration . - The PROSPERO record was updated accordingly, and the revised manuscript now fully aligns with the amended registration. | - Introduction: pg. 3 - Materials and methods: pg. 8-9 - Discussion: pg. 34-35 |
| Operational Definition of "Sustained Use" The term "sustained use" appears throughout the manuscript (abstract, methods Table 2, results) but is never operationally defined. What timeframe constitutes "sustained" use? Three months? Six months? One year? How did the 82 included studies define this construct—did they use consistent definitions or was there heterogeneity?  This omission makes it impossible to evaluate whether your data genuinely support conclusions about sustained use versus initial adoption. Please add a clear operational definition in the Methods section and discuss how varying definitions across studies may have affected your synthesis. | - The term “sustained use” was **removed throughout the manuscript** due to heterogeneous and implicit definitions of use duration across included studies. - The manuscript now **focuses exclusively on digital health technology adoption**, which is consistently supported by the extracted data. - The definition and conceptual framing of adoption were **clarified in the Introduction**, with reference to established literature, while deliberately avoiding the introduction of a time-based operational definition. - All references to “sustained use” were **removed or relabelled in Table 2** to reflect adoption-related constructs only. - Revisions were made to **avoid conflation of adoption with post-adoption or long-term engagement constructs** that were not consistently measured across studies. - The PROSPERO registration was **updated to reflect the refined terminology and scope**, ensuring consistency between the registered protocol and the revised manuscript. | - Abstract: pg. 2-3 - Introduction: pg. 4-7 - Materials and methods: pg. 8-13, 16 - Results: pg. 24 - Discussion: pg. 30, 31, 36 |
| Temporal Inconsistencies  There are contradictions regarding your search dates:  Abstract: "published between 2015 and June 2025"  Methods (line 161): "2015 and June 18, 2025"  Methods (line 175-176): "studies from 2015 to 2019 were further excluded"  Results (line 226): studies "between 2020 and 2025"  Table 2: "2015-2025"  PROSPERO: "1 January 2015 and before by 18 June 2025"  Your final dataset appears to include only 2020-2025 studies. If so, why search 2015-2019 at all? This should be clarified throughout the manuscript, particularly in the abstract and Table 2. If the 2015-2019 exclusion was a post-hoc decision, it should be documented as a protocol deviation with justification. | - All temporal references were **reviewed and aligned** across the Abstract, Methods, Results, tables, and PROSPERO record to ensure consistency in the reported search and synthesis period. - The literature search period was **explicitly stated as 1 January 2015 to 18 June 2025**. - A clarification was added that studies published between 2015 and 2019 were **screened but excluded at the full-text stage** due to limited relevance to contemporary digital health adoption contexts. - The final synthesis was **clarified to include only studies published between 2020 and June 2025**, reflecting system-level digital health adoption. - Table 2 was **revised to reflect the final analytical timeframe of 2020-2025**, removing inconsistent or outdated date references. - The PROSPERO record was **updated to reflect the clarified search period and final synthesis timeframe**, ensuring consistency with the revised manuscript. | - Abstract: pg. 2 - Materials and methods: pg. 8-12, 14, 15 |
| Quality Assessment Approach  Lines 207-212 state "A formal risk-of-bias tool was not applied, as the review did not aim to assess intervention outcomes." However, systematic reviews of factors and barriers still benefit from quality appraisal. Tools like the Mixed Methods Appraisal Tool (MMAT) or JBI Critical Appraisal Checklists are designed precisely for non-interventional studies like yours.  Your own limitations section (lines 404-405) acknowledges that "many [studies] involved small samples or cross-sectional self-reported measurements," yet you provide no systematic assessment of how study quality may have influenced your findings. Without this, readers cannot gauge the robustness of your themes.  At minimum, I recommend retrospectively scoring studies based on their reported limitations and conducting a sensitivity analysis showing whether themes are driven by higher vs. lower quality studies. | - **A formal quality appraisal was conducted for all included studies using the Mixed Methods Appraisal Tool (MMAT), version 2018**, appropriate for qualitative, quantitative, and mixed-methods evidence. - **A dedicated subsection was added describing the MMAT appraisal process**, including assessment by study design category and clarification that appraisal focused on methodological clarity and reporting quality rather than outcome-level bias. - In line with MMAT guidance, numerical quality scores were not calculated and studies were not excluded based solely on quality. Instead, **patterns in the extent to which methodological criteria were met were summarised descriptively to support interpretation**. - **An interpretive sensitivity approach was incorporated** to examine whether core themes were supported across studies with higher and moderate methodological clarity, rather than being driven by lower-quality evidence. - The Results were revised to report that **key thematic domains were consistently supported across studies of varying methodological quality**. - The Quality and Bias Considerations section was **substantially revised**, replacing the previous statement that no formal quality appraisal was conducted. | - Materials and methods: pg. 19 - Result: pg. 27 |
| Rigor of Thematic Analysis  Your description of thematic analysis lacks critical methodological details:  How many researchers coded the data independently?  Was inter-rater reliability calculated (e.g., Cohen's kappa, percentage agreement)?  What software was used for coding, if any?  How were disagreements resolved?  What constituted thematic saturation?  The term "supervisory validation" (line 202) is not a recognized method for ensuring qualitative rigor. Standard approaches include inter-coder reliability testing, peer debriefing, or audit trails. Please provide specific details about your analytical process that would allow readers to evaluate its rigor. | - The Data analysis and synthesis section was **revised to provide explicit operational detail on the rigour of the thematic analysis**. - It was clarified that **coding and theme development were conducted by one researcher (primary reviewer)**, rather than multiple independent coders. - The use of **ATLAS.ti (version 24)** **was explicitly reported** to support systematic coding, retrieval, comparison of codes, and documentation of theme development. - The term “supervisory validation” was **removed and replaced with** **peer debriefing**. Clarification was added that emerging codes and provisional themes were discussed with the supervisor to refine interpretations and resolve interpretative differences through discussion. - **Additional detail was added describing how progression from open coding 🡪 axial categories 🡪 final themes were documented using a thematic analysis matrix and supporting tables/figures**. - The criterion for theme completion was **clarified**, indicating that theme development concluded when additional studies no longer contributed substantively new concepts and when themes demonstrated conceptual coherence and stability across sources. | - Materials and methods: pg. 17-19 - S6 Appendix |
| Over-Claims and Unsupported Assertions  Line 306-312: The statement that findings "prove" adoption is influenced by "a broader range of factors than those embraced by existing adoption models" is problematic for several reasons:  First, "prove" is too strong for qualitative synthesis—consider "demonstrate" or "suggest."  Second, Table 1 shows that integrated models already incorporate psychological, social, cultural, and environmental factors.  For example:  TAM-SCT includes usability metrics  UTAUT-SDT includes autonomy, competence, relatedness  TAM-DTPB includes service environment and self-efficacy  Your manuscript does not provide a systematic comparison showing which specific factors you identified are genuinely absent from these integrated models. Without this analysis, the claim about "broader" factors is not adequately supported.  I recommend creating a comparison table mapping your five themes to constructs in existing integrated models, explicitly identifying where gaps exist. | - The term “prove” was **replaced with interpretive qualitative language (e.g., demonstrate, suggest, are influenced by)** to avoid overstatement and align with qualitative synthesis standards. - **Text was added to explicitly acknowledge that several integrated adoption models already incorporate psychological, social, and motivational constructs**. - Claims were **revised to clarify that the findings do not indicate the absence of these domains in existing models, but rather uneven breadth, depth and consistency of operationalisation across models**. - **A new comparison table (Table 4) was added** to systematically map the five synthesized themes against constructs in prominent integrated adoption models. - Table 4 indicates whether each theme is explicitly represented, partially represented, or not represented, **highlighting gaps related to structural, organisational, governance, equity and sustainability factors**. - The Results and Discussion sections were **revised to ensure claims about “broader” factors are explicitly grounded in the systematic comparison presented in Table 4**. - The Discussion was **revised to emphasise complementarity and extension, rather than superiority**, of the synthesized themes relative to existing adoption models. | - Results: pg. 24-25 - Discussion: pg. 28-35 |
| Search Strategy Concerns  Your final search string (Table 3) requires all five domains to be present, which is highly restrictive. For instance, an excellent qualitative study titled "User experiences with mHealth apps among elderly: barriers and facilitators" might be excluded if it doesn't mention "model" or "theory."  Similarly, your Domain 4 focuses exclusively on negative terms (limitation, barrier, problem, issue) but excludes facilitators and enablers. Studies emphasizing success factors may have been missed.  Did you conduct sensitivity analyses to evaluate how many relevant studies your restrictive string might have excluded? If not, this should be acknowledged as a limitation. | - The Search Strategy section **was revised to explain that pilot searches using broader strings retrieved a high volume of poorly aligned records, leading to a decision to prioritise conceptual specificity over sensitivity**. - **Text was added clarifying that the final search strategy required the concurrent presence of all five domains** to ensure relevance to theory-informed adoption analysis and gap identification. - **A statement was added acknowledging that this approach may have under-captured experiential or practice-oriented qualitative studies** that did not explicitly reference theories or models. - Clarification was added that, although Domain 4 emphasised negatively framed terms (e.g., barriers, limitations, problems), **facilitators and enabling factors were still synthesised when reported within included studies**. - **The trade-off between specificity and sensitivity was explicitly acknowledged as a methodological limitation**. - **Broader or experience-centred search approaches were explicitly identified as a direction for future reviews**. | - Materials and methods: pg. 13-14 - Discussion: pg. 28 |
| Table 1 - Missing References  Table 1 lists ten integrated adoption models but provides no citations. Readers cannot access the original works to understand these models in depth. Please add a citations column or footnote with references for each model. | - Table 1 **was revised to include explicit citations for each integrated adoption model listed**. - **A dedicated References column was added to Table 1** to allow direct access to the original sources for each model. - The revision enhances the transparency and interpretability of Table 1 by **clarifying the theoretical foundations and scope of each integrated adoption model**. | - Table 1: pg. 7-8 |
| Missing Citations for Foundational Theories  Your discussion repeatedly references TAM, UTAUT, HBM, SCT, and SDT, but you cite only recent reviews (refs 21-25) rather than the seminal works. Consider adding:  Davis (1989) for TAM  Venkatesh et al. (2003) for UTAUT  Rosenstock (1974) for HBM  Bandura (1986) for SCT  Deci & Ryan (1985) for SDT  This would strengthen your theoretical grounding. | - **Seminal sources underpinning the core theoretical frameworks were added** to ensure appropriate theoretical grounding and attribution. - Citations were added for the original development of major models, including **TAM (Davis, 1989), UTAUT (Venkatesh et al., 2003), HBM (Rosenstock, 1974), SCT (Bandura, 1986), and SDT (Deci & Ryan, 1985)**. - These seminal works were **incorporated alongside recent review articles to balance foundational theory with contemporary scholarship.** | - Introduction: pg. 6 - Discussion: pg. 27 |
| Geographic Representation in References  I noted your reference list includes studies from Uganda, Ethiopia, Saudi Arabia, Singapore, Germany, and many other countries—but I did not identify any Malaysia-specific studies among refs 56-117. If your dataset includes no Malaysian studies, this raises questions about the feasibility of the Malaysian contextualization promised in PROSPERO. Please clarify how many (if any) included studies are from or about Malaysia. | - It was confirmed that **none of the 82 included studies were conducted in or explicitly focused on Malaysia**, as indicated by the country characteristics of the final included studies. - The review was **reframed as a global synthesis of factors influencing digital health technology adoption** due to the absence of Malaysia-specific empirical evidence. - Malaysia-specific references and contextual framing were **removed or reframed as illustrative policy examples**, ensuring alignment with the global evidence base while maintaining relevance to national strategies. - The PROSPERO registration was **amended to reflect the revised global scope and ensure consistency with the final dataset and analytical focus.** | - Methods: pg. 8-9 - Results: pg. 21 - Discussion: pg. 33-34 - S4 Table |
| Formatting and Style  Line 389: The sudden mention of "Malaysia's Ekonomi MADANI" feels disconnected from the preceding 70+ lines of global discussion. This should be better integrated or expanded into a full section.  Consider revising the limitations section to explicitly address: (a) retrospective registration elements, (b) absence of quality appraisal, (c) restrictive search strategy, (d) protocol deviations from PROSPERO commitments. | - References to Malaysia’s Ekonomi MADANI as contextual grounding were **revised to function as an illustrative policy example within a broader discussion of national digital health strategies** (e.g., inclusivity, social wellbeing, digital transformation), without implying country-specific analysis. - **Text was added to explicitly state that the implications discussed are globally transferabl**e, preserving the global scope and improving narrative coherence. - The Limitations section was **expanded and reorganised to explicitly address four methodological issues raised by the reviewer**. - It was clarified that **pilot searching and refinement of search terms occurred prior to PROSPERO registration**, while confirming that formal screening, data extraction, quality appraisal or synthesis were not conducted before registration. - The absence of an intervention-focused risk-of-bias tool was **justified based on the non-interventional aim of the review**, and the use of MMAT for interpretive methodological appraisal was clarified. - A limitation was added acknowledging that the **theory-oriented and keyword-specific search strategy may not have captured all experiential or practice-oriented studies** lacking explicit adoption or theoretical terminology. - Deviations from the registered PROSPERO protocol, including the removal of planned national contextualisation, **were explicitly stated and justified to ensure consistency with the global evidence base**, and confirmed as formally updated prior to submission. | - Materials and methods: pg. 8-9, 19 - Discussion: pg. 33-35 |
| Data Availability  Your supplementary materials (S1-S5) are helpful. However, S3 Table should ideally include columns for country/setting and population characteristics to allow readers to evaluate heterogeneity and assess the Malaysian representation issue noted above. | - S4 Table was **revised to include explicit columns for country/setting and population characteristics for each included study.** - The added columns **enable direct assessment of geographic and population heterogeneity and make explicit the absence of Malaysia specific studies** within the included evidence base. - The main text was **updated to briefly reference the expanded reporting in S4 Table**, with detailed study-level information retained in the supplementary materials to preserve readability. | - Materials and methods: pg. 17 - Results: pg. 21 - S4 Table |
| It is suggested to provide a more detailed explanation of the specific operational procedures for inclusion and exclusion criteria in the methods section, such as how multilingual literature and grey literature were handled, to enhance the transparency and reproducibility of the study. | - The Inclusion and Exclusion Criteria subsection was **revised to explicitly describe the handling of multilingual literature and grey literature**. - Text was added to **justify restriction to English-language, peer-reviewed journal articles** to ensure consistency, feasibility, and reproducibility of screening and synthesis. - A justification was added clarifying that **inclusion of grey literature was excluded at screening as a quality-control measure due to lack of standardised peer review and variable reporting quality.** - A statement was added confirming that **these clarifications are consistent with the predefined and registered protocol and do not alter the analytical scope.** - The manuscript text was **revised accordingly to reflect these methodological justifications clearly.** | - Materials and methods: pg. 8-9, 15-16 |
| The paper mentions the use of the SPIDER framework, but the description of the mapping relationship between SPIDER and PICOS during the actual screening and data extraction process is somewhat brief. It is recommended to supplement a specific mapping table and application examples. | - The Materials and Methods section was **revised to explicitly explain how the SPIDER framework was mapped to PICOS for operational use during screening and data extraction**. - The SPIDER–PICOS eligibility mapping table was retained as Table 2, and **an additional application-focused column was added** to demonstrate how the mapped framework was applied in practice. - The added column in Table 2 **explicitly shows application across title and abstract title-abstract screening, full-text review, and data extraction.** - Short explanatory paragraphs were **added to illustrate how each SPIDER element guided eligibility decisions and data extraction when implemented through the PICOS structure in Covidence.** | - Materials and methods: pg. 9-12 |
| The paper frequently refers to "multidisciplinary factors," but what are the specific roles and weights of psychological, social, and environmental factors in the adoption of DHTs? It is suggested to supplement the above data, as similar content does not seem to be present in the paper. | - The manuscript was **systematically revised across the Methods, Results, and Discussion sections to clarify the specific roles and relative emphasis (“weights”) of psychological, social, and environmental factors**, without introducing new data. - The coding process was **explicitly described as a three-stage analytical workflow, open coding, axial coding, and thematic synthesis, supported by a Thematic Analysis Matrix (TAM)**. - Final themes were **explicitly mapped to analytical domains** (psychological, social, environmental, technological, health, cultural) based on their primary locus of influence, rather than treated as independent thematic outputs. - The relative “weight” of each domain was **defined as frequency-based thematic prominence and breadth of conceptual discussion**, rather than statistical effect sizes. - The Results section was **reorganised to make domain-specific roles explicit**, showing differential concentration of psychological, social/cultural, and environmental/organisational factors across themes. - An integrative summary paragraph was **added to explicitly describe the relative emphasis of psychological, social, and environmental domains across the synthesised themes.** - The Discussion was **revised to interpret these patterns**, distinguishing dominant individual- and relational-level drivers from contextual and structural determinants shaping access, equity, and sustainability. - The Discussion now **explicitly links these findings to imbalances in existing adoption models**, which prioritise individual cognitive constructs while underrepresenting structural and contextual factors. | - Materials and methods: pg. 17-18 - Results: pg. 23-27 - Discussion, pg. 28-30, 32-34 |
| It is recommended to provide additional details on coding and other data governance aspects in the data methodology section, as this will impact the replicability of the study. | - The Materials and Methods section was **expanded to explicitly describe data governance procedures**, including secure storage of extracted data, controlled access to analytical materials, and systematic organisation of screening, extraction, and coding outputs. - A detailed, sequential description of the analytical workflow was **added**, covering familiarisation with extracted findings, first-cycle (open) coding, iterative refinement and grouping of codes, and abstraction into themes and subthemes via axial and thematic synthesis. - The use of ATLAS.ti to support qualitative coding, data organisation, and retrieval was **explicitly stated.** - Additional detail was **added describing how themes and subthemes were iteratively reviewed and refined** through repeated comparison with the original extracted data to ensure internal coherence and clear conceptual boundaries. - The role of peer debriefing via supervisory analytical review was **clarified as a strategy to examine coding logic and thematic interpretations and to minimise individual researcher bias.** - A dedicated subsection was **added explaining how credibility, dependability, confirmability, and transparency were addressed through consistent procedures and explicit reporting of methodological decisions.** | - Materials and methods: pg. 17-18, 20 |
| How can DHTs better ensure data privacy, especially in cases involving AI? It is suggested to discuss this in the discussion and limitation sections. I believe there may be significant differences across regions of the world. | - The Discussion section was **expanded to clarify that data privacy concerns in AI-enabled DHTs extend beyond technical data security to include secondary data use, algorithmic inference, profiling, and opacity in automated decision-making**, and that these features may heighten perceived risk, uncertainty, and trust concerns. - Text was **added to explicitly highlight regional variation in privacy protection**, arising from differences in legal frameworks, regulatory enforcement capacity, cultural norms around data sharing, and digital infrastructure maturity. - The Discussion now **explicitly states that trust-enhancing mechanisms and privacy safeguards effective in one jurisdiction may not be directly transferable to other contexts.** - The Limitations section was **revised to acknowledge that the review did not conduct jurisdiction-specific analyses of data protection laws or AI governance frameworks**, despite synthesising evidence from multiple geographic contexts. - Findings related to privacy and trust were **explicitly framed as context-sensitive rather than universally generalisable.** | - Discussion: pg. 31-32, 35 |
| The limitations section mentions the insufficiency of sample size and cross-sectional studies, but the suggestions for future research directions are relatively general. It is recommended to propose more actionable suggestions, such as longitudinal studies and intervention studies. | - The Limitations and Future Research section was **revised to move beyond general statements and provide actionable methodological directions.** - Explicit recommendations were **added for longitudinal study designs to examine trajectories of adoption, disengagement, and continuity of use over time**, addressing limitations of cross-sectional evidence. - The revised text now **recommends intervention and quasi-experimental studies to empirically assess the impact of technological features, behavioural strategies, and policy interventions on real-world adoption and sustained engagement.** | - Discussion: pg. 35 |

*All page numbers refer to the **Manuscript** document.
