# Supplementary material for: Advancing foundational models in digital health technology adoption: A systematic literature review of multidisciplinary factors
Source: PLOS Digit Health. 2026 Sep 2;5(9):e0001690. doi: 10.1371/journal.pdig.0001690 (PMC13537594; doi:10.1371/journal.pdig.0001690)
Supplement: Supplementary file 14 [file pdig.0001690.s014.docx]

**Response to Reviewers**

**Manuscript number:** PDIG-D-25-00816

**Manuscript Title:** Advancing foundational models in digital health technology adoption: A systematic literature review of multidisciplinary factors

We sincerely thank the Editor and the Reviewers for their constructive and insightful comments. We have carefully revised the manuscript in response to each point raised. Below, we provide a detailed point-by-point response.

| **Comment** | **Response** | **Page number** |
| --- | --- | --- |
| **Background of the study:**  The claims advanced by the authors should be substantiated with empirical evidence, including relevant data, statistics, and credible sources. The authors assert that the adoption of DHT is impeded by persistent design and usability challenges. However, the validity of this claim remains unclear unless it is supported with specific evidence. The authors should clearly identify the design and usability challenges, specifying the countries or regions concerned, as well as the relevant time periods. Furthermore, this assertion should be supported by empirical findings from multiple studies conducted across different countries, including Malaysia. Reliance on secondary sources such as systematic literature reviews (e.g., reference 14) or evidence limited to a single country (e.g., reference 16) is insufficient to substantiate such a broad claim. | - The Introduction was substantially revised to strengthen the empirical justification for claims regarding DHT adoption barriers. - Additional empirical evidence from multiple countries, including the United Kingdom, China, Singapore, Thailand, Saudi Arabia, Ethiopia, and Malaysia, was incorporated to demonstrate that usability- and design-related barriers occur across diverse healthcare settings. - The revised manuscript now explicitly identifies specific usability and design challenges, including complex interfaces, poor navigation structures, excessive information presentation, limited localisation, language barriers, and insufficient accessibility features. - Country-specific examples were added to demonstrate that these challenges are not limited to a single healthcare system or national context. - The discussion was strengthened through the incorporation of multiple empirical studies across different countries rather than relying primarily on secondary reviews or single-country evidence. | - Introduction: pg. 4 - 5 |
| **Problem statement:**  The authors assert that there is no comprehensive framework for inclusive design in DHT; however, this claim is not supported by sufficient evidence. It would be beneficial for the authors to include a comparative table summarising prior studies and the specific frameworks they have adopted, thereby demonstrating the extent to which these frameworks have addressed inclusivity. Furthermore, the manuscript would benefit from a clearer justification for the inclusion of additional constructs—namely psychological, social, cultural, health-related, and environmental factors—within the proposed DHT model. Providing empirical or real-world evidence to support the relevance of these factors would significantly strengthen the study’s problem statement and overall argument. | - Table 1 was substantially expanded and revised into a comparative framework table to examine integrated DHT adoption models and their representation of inclusivity-related dimensions. - The revised Table 1 now compares technological, psychological, social, cultural, health-related, and environmental dimensions across integrated DHT adoption models. - The Introduction was revised to provide a clearer progression from foundational models to integrated DHT adoption frameworks. - Additional empirical and real-world evidence was incorporated to justify the inclusion of psychological, social, cultural, health-related, and environmental factors. - The manuscript now gives clearer examples of inclusivity-related issues, including stigma, language mismatch, localisation barriers, infrastructure access, affordability, organisational readiness, and social support. - These revisions clarify that existing models provide only partial explanations of DHT adoption and inconsistently represent broader contextual determinants. | - Introduction: pg. 5 - 8 |
| **Methodology:**  The authors refer to “dynamic and multi-level DHT adoption,” yet it is unclear how this concept is defined or how it is insufficiently addressed by existing technology adoption models. The manuscript would benefit from a more detailed elaboration of this concept, including a clear explanation of its dimensions and the specific limitations of current models in capturing these aspects. | - A dedicated explanatory paragraph was added to define "dynamic" and "multi-level" DHT adoption. - "Dynamic" adoption is now explained as the evolving nature of DHT engagement across stages such as initial adoption, sustained use, discontinuance, and re-engagement. - "Multi-level" adoption is now clarified as referring to interactions between individual, interpersonal/social, organisational, technological, and environmental influences. - The manuscript now explains that existing models such as TAM and UTAUT provide limited coverage of broader contextual determinants of DHT adoption. - Additional discussion was added to explain how real-world DHT adoption is shaped by interactions between users, technologies, infrastructures, organisational conditions, and social environments. | - Introduction: pg. 6 - 7 |
| **Findings:**  The authors provide a detailed explanation of the study’s methodology, including the study period and sampling selection criteria. However, a descriptive overview of the study sample is not presented. The manuscript states that the number of studies increased over time, reaching a peak in 2024, yet no supporting data, figures, or tables are provided to substantiate this claim. The authors are encouraged to include relevant descriptive statistics, such as the number of studies by year, country, and 379 – 393 population of interest (e.g., patients, clinical workers, hospital management). Additionally, it would be beneficial to categorise the studies according to domain areas, including technological, psychological, social, cultural, health, and environmental dimensions. | - A descriptive overview of the included studies was added to the Results section. - Table 4 was added to summarise study characteristics, including publication year, country/region, study population and methodological approach. - The revised manuscript now provides supporting information for publication trends, including the increase in studies and the peak observed in 2024. - The included studies were categorised according to geographical setting, population group and methodological design to improve transparency. - The thematic findings were further elaborated to clarify how technological, psychological, social, cultural, health-related, organisational and environmental dimensions were reflected across the identified themes. - The revised manuscript also clarifies that many included studies addressed multiple interconnected domains simultaneously rather than examining them as separate categories. | - Result: pg. 25 - 26 |
| **Research gap:**  The effectiveness of the presentation would be enhanced by incorporating tables and figures to summarise the study’s findings. In its current form, the absence of sufficiently robust supporting evidence limits the clarity with which the research gaps in prior studies are articulated. | - The presentation of findings was strengthened through additional tables and figures summarising conceptual relationships and thematic findings. - Table 1 was substantially revised as noted above. - The discussion of research gaps was strengthened by explicitly identifying underrepresented contextual dimensions, including cultural, environmental, organisational, governance, equity-related, and sustainability-related factors. - The revised manuscript now more clearly demonstrates how existing models inconsistently represent broader real-world determinants of DHT adoption. | - Introduction: pg. 8 - Discussion: pg. 35 - 38 |
| **Contribution:**  The contribution of the study is not clearly articulated. It is important to specify the intended beneficiaries of the research. Given that the technology acceptance model encompasses multiple user groups—such as patients, caregivers, and healthcare administrators/clinicians—the study would benefit from a more structured presentation of findings, categorised according to each user group’s adoption context. | - The contribution section was substantially revised to more clearly articulate the theoretical, practical, and policy implications of the review. - The intended beneficiaries are now explicitly identified, including patients, caregivers, healthcare professionals, healthcare administrators, policymakers, developers, and researchers. - Table 6 was added to present key DHT adoption factors and implications according to stakeholder group. - The revised discussion now differentiates how usability, trust, workflow integration, organisational readiness, accessibility, governance, and equity influence different user groups and adoption contexts. - The manuscript now more clearly explains how the findings may support equitable, user-centred, context-sensitive, and scalable DHT implementation strategies across healthcare systems. | - Discussion: pg. 40 - 41 |

*All page numbers refer to the **Manuscript** document.
