# Supplementary material for: Advancing foundational models in digital health technology adoption: A systematic literature review of multidisciplinary factors
Source: PLOS Digit Health. 2026 Sep 2;5(9):e0001690. doi: 10.1371/journal.pdig.0001690 (PMC13537594; doi:10.1371/journal.pdig.0001690)
Supplement: Supplementary file 15 [file pdig.0001690.s015.docx]

**Response to Reviewers**

**Manuscript number:** PDIG-D-25-00816

**Manuscript Title:** Advancing foundational models in digital health technology adoption: A systematic literature review of multidisciplinary factors

We sincerely thank the Editor and the Reviewers for their constructive and insightful comments. We have carefully revised the manuscript in response to each point raised. Below, we provide a detailed point-by-point response.

| **Comment** | **Response** | **Page number** |
| --- | --- | --- |
| **Protocol registration - disclosure must be accurate and dated.** The Limitations acknowledge that amendments were made and describe them as "refinement of eligibility criteria and clarification of the analytical scope by removing the originally planned national contextualisation," stating these were "transparently documented in the PROSPERO record." This understates what occurred. The registered review was Malaysia-specific in its title, objectives, country and scope, and remained so through the second protocol version; the global reframing-including the new title, revised objectives and population, and the addition of MMAT appraisal-was introduced in a later amendment dated well after searching and screening were complete. I ask the authors to disclose this sequence explicitly and with dates in the Methods and Limitations: that the original registered scope was national; that the global scope and the appraisal approach were introduced by post-registration amendment after the corpus had been screened; and the rationale for that change. Relatedly, "prospective registration" is currently listed among the review's strengths (Methods/Discussion); given the amendment history, this should be removed or carefully qualified. The point here is not the amendment itself-protocols may legitimately be amended-but that the manuscript's account should match the registry's timeline so a reader can interpret the registration correctly. | - Revised the Materials and Methods section to provide a transparent chronological account of the PROSPERO registration and subsequent amendments. - Clarified that the review was initially registered on 26 May 2025 as a Malaysia-focused review, followed by a first amendment on 20 July 2025 and a second amendment on 5 February 2026. - Explicitly stated that study selection had been completed before the second amendment, while the subsequent data analysis and evidence synthesis were conducted in accordance with the revised protocol. - Explained that the amendments were undertaken to improve methodological alignment and transparency rather than in response to the review findings. - Replaced the description of "prospective registration" with "protocol registration with transparently documented amendments." - Revised the Limitations section to acknowledge the methodological implications of the protocol amendments and clarify that all amendments are publicly documented in the PROSPERO record. | - Materials and Methods: pg. 12 - 13 (line 204 - 222) - Discussion - Limitations: pg. 44 (line 772 - 774) |
| **Methodological coherence - the analysis is labelled reflexive thematic analysis but is operationalised as framework/template analysis.** The manuscript cites Braun and Clarke's reflexive TA, yet the procedures described are predominantly deductive and draw on incompatible traditions: open coding begins from a priori "sensitising domain descriptors" fixed to the six domains; "axial coding" and "axial-informed" mapping (grounded theory) appear throughout; completion is defined by saturation ("no substantively new concepts emerged"); and rigour is framed through Lincoln and Guba's credibility/dependability/confirmability criteria. Several of these are explicitly argued against in the reflexive-TA literature. I recommend reconciling this in one direction: either commit to reflexive TA and remove the saturation, axial-coding and trustworthiness-criterion scaffolding, or-which fits what is actually described-relabel the approach as framework- or matrix-based qualitative synthesis. The latter is the lower-burden fix and additionally dissolves the apparent tension around single-coder analysis, since framework synthesis accommodates it. | - Revised the Data analysis and synthesis section to provide a clearer and more internally consistent description of the analytical workflow. - Clarified that the review followed the structured thematic analysis workflow proposed by Zairul, which operationalises the thematic analysis principles described by Braun and Clarke through the use of a thematic analysis matrix. - Removed terminology associated with grounded theory (such as open coding, axial coding, and axial-informed mapping) to improve methodological consistency. - Clarified that the six analytical domains functioned as sensitising concepts to guide the initial coding process, while additional concepts were generated inductively and incorporated into subsequent categories and themes. - Revised the descriptions of coding, category development and theme generation to accurately reflect the analytical procedures undertaken. - Updated Figure 2, its caption, S6 Appendix, and related methodological descriptions to ensure consistency throughout the manuscript. | - Materials and Methods - Data analysis and synthesis: pg. 21 - 23 (line 345 - 380) - Figure 2 - S6 Appendix |
| **"Sustained / continued use" claims persist despite removal of the construct.** The term "sustained use" was removed from the title and abstract, but the Results and Discussion repeatedly assert "continued use," "continued adoption," "long-term engagement," DHTs being "more sustainable," and adoption being "sustained beyond pilot implementation." These reintroduce the construct the manuscript set aside. Because the corpus is largely cross-sectional and use-duration was not consistently measured, I ask the authors either to soften these to initial-adoption language consistent with the evidence, or to define and own the construct explicitly. The decision should be applied consistently across abstract, results and discussion. | - Conducted a comprehensive review of the manuscript to identify terminology implying sustained or long-term user behaviour. - Replaced expressions implying continued use, continued adoption, or long-term engagement with terminology that reflects the evidence synthesised in this review. - Retained references to sustainability only where they relate to organisational or implementation sustainability rather than sustained user behaviour. - Revised the Limitations section to clarify that the predominantly cross-sectional evidence informs factors influencing digital health technology adoption rather than sustained use or long-term engagement. | - Results: pg. 29 - 31 (line 475 - 477, 483 - 485, 517 - 519, 525 - 527) - Discussion: pg. 35 - 41 (line 626 - 630, 653 - 654, 676 - 677, 681 - 683, 721 - 724) |
| **Acronym collision.** "Thematic Analysis Matrix (TAM)" collides with the Technology Acceptance Model, which is central to this paper and recurs as TAM and TAM-HBM/TAM-TPB/TAM-SCT. Please rename the analytic matrix (e.g., "TA Matrix") and reserve TAM for the model, consistently across the Abstract, Methods and Appendix S6. | - Removed the acronym TAM when referring to the thematic analysis matrix throughout the manuscript (Abstract, Methods, Figure 2 and S6 Appendix). - Referred to the analytical tool consistently as the thematic analysis matrix. - Reserved the acronym TAM exclusively for the Technology Acceptance Model and its integrated variants (such as TAM-HBM, TAM-TPB and TAM-SCT). | - Abstract: pg. 2 (line 42 - 44) - Materials and Methods - Data analysis and synthesis: pg. 21 - 24 (line 345 - 347, 404 - 405) - Figure 2 - S6 Appendix |
| **Minor consistency and copyediting - factor taxonomy.** The factor taxonomy drifts between six domains and additional "organisational" and "equity-related" labels-please fix on one scheme. | - Clarified that the six analytical domains served as the overarching analytical framework throughout the coding process. - Explained that organisational readiness, workforce capacity, governance, affordability and equity emerged as cross-cutting concepts synthesised within the higher-order themes rather than as additional analytical domains. - Revised the Methods, Results, Discussion, and S6 Appendix to consistently distinguish the analytical domains from the higher-order synthesis themes. | - Materials and Methods - Data analysis and synthesis: pg. 22 (line 356 - 372) - Results: pg. 32 (line 541 - 549) - Discussion: pg. 34 - 35 (607 - 611, 620 - 626) - S6 Appendix |
| **Minor consistency and copyediting - Table signposting.** Table 1 and Table 5 are both model-comparison tables (dimensions vs themes); a one-line signpost distinguishing their purpose would prevent the appearance of redundancy. | - Added a signposting sentence before Table 5 to distinguish its purpose from Table 1. - Clarified that Table 1 provides a conceptual comparison of existing integrated digital health technology adoption models, whereas Table 5 maps the higher-order synthesis themes identified in this review against those models to illustrate their conceptual alignment and coverage. | - Discussion: pg. 35 (612 - 616) |
| **Minor consistency and copyediting - Table 6.** Table 6 omits developers and researchers, who are named as beneficiaries in the prose. | - Expanded Table 6 to include Developers and Researchers as stakeholder groups. - Ensured consistency between the narrative discussion and the stakeholder summary table. | - Table 6 |
| **Minor copyedit -**  **Date format.** Change "June, 18 2025" to the correct date format.  **Duplicate wording.** Correct "such as such as" in the Methods section.  **Punctuation.** Replace the full-width comma in "feedback，" in the Results section.  **Typographical error.** Correct the broken phrase "poor 'webside: manner" in the Results section.  **Missing word.** Correct "app abandonment 30 to 90 days" by inserting the missing word "within". | - Corrected the date format ("18 June 2025"). - Removed duplicated wording. - Corrected punctuation and quotation formatting. - Corrected typographical errors and inserted the missing word "within" where appropriate. - Performed an additional proofreading of the manuscript to improve consistency, readability and language throughout. | - Throughout the manuscript (line 35, 273, 282, 459, 518, 650) |
| **Strengthening practical implications (optional).** The Discussion currently provides generic recommendations (e.g., user-centred design, cultural relevance, accessibility). The reviewer suggests synthesising the concrete findings into concise implementation guidance, for example a "Design Considerations: Incorporate / Avoid" section covering localisation, multilingual interfaces, low-bandwidth/offline capability, transparent costs, actionable feedback, privacy controls, explainable AI with human oversight, EHR interoperability, workflow fit, clinical validation signals, while avoiding notification overload, opaque automated decisions, hidden costs, top-down design, and one-size-fits-all approaches. This is presented as an optional enhancement rather than a required revision. | - Strengthened the **Practical Implications** section by incorporating concise implementation guidance synthesised directly from the thematic findings. - Summarised key design considerations to incorporate, including multilingual and culturally appropriate interfaces, low-bandwidth functionality, transparent privacy controls, explainable AI with human oversight, interoperability, workflow integration and communication of clinical validation. - Summarised implementation practices to avoid, including notification overload, top-down design approaches, opaque automated decision-making, hidden costs and one-size-fits-all solutions. | - Discussion - Practical implications: pg. 41 (725 - 735) |

*All page numbers refer to the **Revised** **Manuscript** document.
