# Supplementary material for: Advancing foundational models in digital health technology adoption: A systematic literature review of multidisciplinary factors
Source: PLOS Digit Health. 2026 Sep 2;5(9):e0001690. doi: 10.1371/journal.pdig.0001690 (PMC13537594; doi:10.1371/journal.pdig.0001690)
Supplement: Peer review history

# Peer review: Decision Letter 0

**DOI:** 10.1371/journal.pdig.0001690.r001

**Authors:** Harry Hochheiser, Hadi Ghasemi

8 Jan 2026

Response to Reviewers Revised Manuscript with Track Changes Manuscript

**Journal Requirements:**

1. As required by our policy on Data Availability, please ensure your manuscript or supplementary information includes the following:

A numbered table of all studies identified in the literature search, including those that were excluded from the analyses.

For every excluded study, the table should list the reason(s) for exclusion.

If any of the included studies are unpublished, include a link (URL) to the primary source or detailed information about how the content can be accessed.

A table of all data extracted from the primary research sources for the systematic review and/or meta-analysis. The table must include the following information for each study:

Name of data extractors and date of data extraction

Confirmation that the study was eligible to be included in the review.

All data extracted from each study for the reported systematic review and/or meta-analysis that would be needed to replicate your analyses.

If data or supporting information were obtained from another source (e.g. correspondence with the author of the original research article), please provide the source of data and dates on which the data/information were obtained by your research group.

If applicable for your analysis, a table showing the completed risk of bias and quality/certainty assessments for each study or outcome.  Please ensure this is provided for each domain or parameter assessed. For example, if you used the Cochrane risk-of-bias tool for randomized trials, provide answers to each of the signalling questions for each study. If you used GRADE to assess certainty of evidence, provide judgements about each of the quality of evidence factor. This should be provided for each outcome.

An explanation of how missing data were handled.

This information can be included in the main text, supplementary information, or relevant data repository. Please note that providing these underlying data is a requirement for publication in this journal, and if these data are not provided your manuscript might be rejected.

**Reviewers' Comments:**

**Comments to the Author**

1. Does this manuscript meet PLOS Digital Health’s publication criteria?

Reviewer #1: Yes

Reviewer #2: Partly

**********

2. Has the statistical analysis been performed appropriately and rigorously?

Reviewer #1: Yes

Reviewer #2: N/A

**********

3. Have the authors made all data underlying the findings in their manuscript fully available (please refer to the Data Availability Statement at the start of the manuscript PDF file)?

The PLOS Data policy

Reviewer #1: Yes

Reviewer #2: Yes

**********

4. Is the manuscript presented in an intelligible fashion and written in standard English?

Reviewer #1: Yes

Reviewer #2: Yes

**********

Reviewer #1: This paper systematically reviews the multidisciplinary factors influencing the adoption and sustained use of digital health technologies (DHTs) by analyzing 82 studies published between 2020 and 2025. The study holds certain value for the future application of DHTs in the field of public health. It is recommended to revise the paper to further enhance its quality.

Reviewer #2: Thank you for submitting this systematic review examining factors influencing digital health technology adoption. The review addresses an important topic and represents substantial effort in synthesizing 82 studies. However, several critical issues require resolution before the manuscript can be considered for publication.

Major Concerns

1. PROSPERO Registration and Protocol Fidelity

I have carefully reviewed your PROSPERO registration (CRD420251056883, provided as S1 Protocol). The registration explicitly states in the "Additional Information" section that "the findings will be contextualized and critically evaluated for practicability and relevance to Malaysia's healthcare management." However, the submitted manuscript provides only one brief sentence mentioning Malaysian policies (lines 389-391), with no substantive contextualization, transferability analysis, or Malaysia-specific recommendations.

This represents a fundamental disconnect between what was registered and what has been delivered. The manuscript reads as a global review with a tokenistic reference to Malaysia, rather than a review with findings "critically evaluated" for the Malaysian context as promised.

You must address this in one of three ways:

Option A: Substantially revise the manuscript to fulfill the protocol commitment by adding dedicated sections analyzing how each theme applies to Malaysia's healthcare system, what adaptations are needed, and specific recommendations for Malaysian policymakers (MADANI framework, Health White Paper, Digital Economy Blueprint).

Option B: Submit an amendment to PROSPERO removing the Malaysian focus, acknowledge this as a major protocol deviation in the manuscript, and reframe the work as a global review with Malaysia as one example context.

Option C: Acknowledge that the current manuscript does not align with the registered protocol and withdraw for resubmission after conducting the promised Malaysian contextualization.

Additional timing concern: Your protocol indicates work began 23 March 2025 but PROSPERO registration occurred 26 May 2025. This two-month gap means some methodological decisions were made before prospective registration, which reduces the value of registration for preventing bias. While I understand pragmatic constraints, this should be acknowledged as a limitation.

2. Operational Definition of "Sustained Use"

The term "sustained use" appears throughout the manuscript (abstract, methods Table 2, results) but is never operationally defined. What timeframe constitutes "sustained" use? Three months? Six months? One year? How did the 82 included studies define this construct—did they use consistent definitions or was there heterogeneity?

This omission makes it impossible to evaluate whether your data genuinely support conclusions about sustained use versus initial adoption. Please add a clear operational definition in the Methods section and discuss how varying definitions across studies may have affected your synthesis.

3. Temporal Inconsistencies

There are contradictions regarding your search dates:

Abstract: "published between 2015 and June 2025"

Methods (line 161): "2015 and June 18, 2025"

Methods (line 175-176): "studies from 2015 to 2019 were further excluded"

Results (line 226): studies "between 2020 and 2025"

Table 2: "2015-2025"

PROSPERO: "1 January 2015 and before by 18 June 2025"

Your final dataset appears to include only 2020-2025 studies. If so, why search 2015-2019 at all? This should be clarified throughout the manuscript, particularly in the abstract and Table 2. If the 2015-2019 exclusion was a post-hoc decision, it should be documented as a protocol deviation with justification.

4. Quality Assessment Approach

Lines 207-212 state "A formal risk-of-bias tool was not applied, as the review did not aim to assess intervention outcomes." However, systematic reviews of factors and barriers still benefit from quality appraisal. Tools like the Mixed Methods Appraisal Tool (MMAT) or JBI Critical Appraisal Checklists are designed precisely for non-interventional studies like yours.

Your own limitations section (lines 404-405) acknowledges that "many [studies] involved small samples or cross-sectional self-reported measurements," yet you provide no systematic assessment of how study quality may have influenced your findings. Without this, readers cannot gauge the robustness of your themes.

At minimum, I recommend retrospectively scoring studies based on their reported limitations and conducting a sensitivity analysis showing whether themes are driven by higher vs. lower quality studies.

5. Rigor of Thematic Analysis

Your description of thematic analysis lacks critical methodological details:

How many researchers coded the data independently?

Was inter-rater reliability calculated (e.g., Cohen's kappa, percentage agreement)?

What software was used for coding, if any?

How were disagreements resolved?

What constituted thematic saturation?

The term "supervisory validation" (line 202) is not a recognized method for ensuring qualitative rigor. Standard approaches include inter-coder reliability testing, peer debriefing, or audit trails. Please provide specific details about your analytical process that would allow readers to evaluate its rigor.

6. Over-Claims and Unsupported Assertions

Line 306-312: The statement that findings "prove" adoption is influenced by "a broader range of factors than those embraced by existing adoption models" is problematic for several reasons:

First, "prove" is too strong for qualitative synthesis—consider "demonstrate" or "suggest."

Second, Table 1 shows that integrated models already incorporate psychological, social, cultural, and environmental factors. For example:

TAM-SCT includes usability metrics

UTAUT-SDT includes autonomy, competence, relatedness

TAM-DTPB includes service environment and self-efficacy

Your manuscript does not provide a systematic comparison showing which specific factors you identified are genuinely absent from these integrated models. Without this analysis, the claim about "broader" factors is not adequately supported.

I recommend creating a comparison table mapping your five themes to constructs in existing integrated models, explicitly identifying where gaps exist.

7. Search Strategy Concerns

Your final search string (Table 3) requires all five domains to be present, which is highly restrictive. For instance, an excellent qualitative study titled "User experiences with mHealth apps among elderly: barriers and facilitators" might be excluded if it doesn't mention "model" or "theory."

Similarly, your Domain 4 focuses exclusively on negative terms (limitation, barrier, problem, issue) but excludes facilitators and enablers. Studies emphasizing success factors may have been missed.

Did you conduct sensitivity analyses to evaluate how many relevant studies your restrictive string might have excluded? If not, this should be acknowledged as a limitation.

Moderate Concerns

8. Table 1 - Missing References

Table 1 lists ten integrated adoption models but provides no citations. Readers cannot access the original works to understand these models in depth. Please add a citations column or footnote with references for each model.

9. Missing Citations for Foundational Theories

Your discussion repeatedly references TAM, UTAUT, HBM, SCT, and SDT, but you cite only recent reviews (refs 21-25) rather than the seminal works. Consider adding:

Davis (1989) for TAM

Venkatesh et al. (2003) for UTAUT

Rosenstock (1974) for HBM

Bandura (1986) for SCT

Deci & Ryan (1985) for SDT

This would strengthen your theoretical grounding.

10. Geographic Representation in References

I noted your reference list includes studies from Uganda, Ethiopia, Saudi Arabia, Singapore, Germany, and many other countries—but I did not identify any Malaysia-specific studies among refs 56-117. If your dataset includes no Malaysian studies, this raises questions about the feasibility of the Malaysian contextualization promised in PROSPERO. Please clarify how many (if any) included studies are from or about Malaysia.

Minor Issues

11. Formatting and Style

Line 389: The sudden mention of "Malaysia's Ekonomi MADANI" feels disconnected from the preceding 70+ lines of global discussion. This should be better integrated or expanded into a full section.

Consider revising the limitations section to explicitly address: (a) retrospective registration elements, (b) absence of quality appraisal, (c) restrictive search strategy, (d) protocol deviations from PROSPERO commitments.

12. Data Availability

Your supplementary materials (S1-S5) are helpful. However, S3 Table should ideally include columns for country/setting and population characteristics to allow readers to evaluate heterogeneity and assess the Malaysian representation issue noted above.

Recommendations

This review has merit—the synthesis of 82 studies is substantial work, and the five themes are logical and relevant. However, the manuscript cannot be accepted in its current form due to the critical protocol fidelity issue and methodological gaps.

Priority actions:

Resolve the PROSPERO-manuscript disconnect (see options A-C above)

Define "sustained use" operationally

Clarify and correct temporal inconsistencies

Address quality assessment gap

Provide detailed thematic analysis methodology

Moderate claims and provide evidence for "broader factors" assertion

Add Malaysia-specific content or remove Malaysia framing entirely

I believe these issues are addressable with substantial revision. The underlying data appear sound, but transparency and alignment with registered commitments are essential for publication in PLOS Digital Health.

I hope these comments are constructive and help strengthen your manuscript.

**********

what does this mean?). If published, this will include your full peer review and any attached files.

**Do you want your identity to be public for this peer review?**  If you choose “no”, your identity will remain anonymous but your review may still be made public.

For information about this choice, including consent withdrawal, please see our Privacy Policy

Reviewer #1: No

Reviewer #2: No

**********

**Figure resubmission:**

**Reproducibility:**

To enhance the reproducibility of your results, we recommend that authors of applicable studies deposit laboratory protocols in protocols.io, where a protocol can be assigned its own identifier (DOI) such that it can be cited independently in the future. Additionally, PLOS ONE offers an option to publish peer-reviewed clinical study protocols. Read more information on sharing protocols at https://plos.org/protocols?utm_medium=editorial-email&utm_source=authorletters&utm_campaign=protocols

Attachment Submitted filename: Reviewer comment.docx

# Author comment: Author response to Decision Letter 1

**DOI:** 10.1371/journal.pdig.0001690.r002

22 Feb 2026

Attachment Submitted filename: Response to Reviewers.docx

# Peer review: Decision Letter 1

**DOI:** 10.1371/journal.pdig.0001690.r003

**Authors:** Harry Hochheiser

5 May 2026

Response to ReviewersRevised Manuscript with Track ChangesManuscript**Journal Requirements:** **Additional Editor Comments (if provided):** **Reviewers' Comments:**

**Comments to the Author**

Reviewer #2: All comments have been addressed

Reviewer #3: (No Response)

**********

publication criteria?

Reviewer #2: Yes

Reviewer #3: Partly

**********

3. Has the statistical analysis been performed appropriately and rigorously?

Reviewer #2: Yes

Reviewer #3: I don't know

**********

4. Have the authors made all data underlying the findings in their manuscript fully available (please refer to the Data Availability Statement at the start of the manuscript PDF file)?

The PLOS Data policy

Reviewer #2: Yes

Reviewer #3: No

**********

5. Is the manuscript presented in an intelligible fashion and written in standard English?

Reviewer #2: Yes

Reviewer #3: Yes

**********

Reviewer #2: Dear Authors,

I have completed my review of the revised manuscript. I am pleased to confirm that you have addressed all concerns comprehensively and with exceptional thoroughness.

Major strengths of the revision:

Methodological transparency: The formal PROSPERO amendment and candid acknowledgment of protocol deviations (lines 680-685) demonstrate scientific integrity. This honesty strengthens rather than weakens the manuscript.

Analytical rigor: The addition of MMAT appraisal (S7, S8), detailed thematic analysis workflow, and Table 4 comparison substantially elevate the methodological and theoretical contributions of this review.

Conceptual clarity: Removing "sustained use" and focusing exclusively on "adoption" eliminates ambiguity and better reflects your evidence base.

Table 4: This is the centerpiece of your theoretical contribution. The systematic mapping of themes to existing models explicitly demonstrates where conceptual gaps exist, transforming your "broader factors" claim from assertion to evidence-based conclusion.

Limitations section: Your expanded and honest discussion of limitations (lines 653-690), including protocol timing and amendments, sets a high standard for transparent reporting.

The manuscript now represents a rigorous, well-documented global synthesis with clear theoretical contributions. The five themes are robustly supported across studies of varying methodological quality (as demonstrated by your MMAT sensitivity analysis), appropriately positioned relative to existing theory, and thoroughly documented through comprehensive supplementary materials.

This manuscript makes a valuable contribution to the digital health adoption literature by systematically identifying where existing theoretical models underrepresent structural, organizational, and equity-related factors. Your transparency about methodological decisions and protocol evolution is exemplary.

Congratulations on producing high-quality scholarship.

Reviewer #3: Please refer to my review report for my comments.

**********

what does this mean?). If published, this will include your full peer review and any attached files.

**Do you want your identity to be public for this peer review?**  If you choose “no”, your identity will remain anonymous but your review may still be made public.

For information about this choice, including consent withdrawal, please see our Privacy Policy

Reviewer #2: **Yes:**  Alvaro Alaor Silva

Reviewer #3: No

**********

**Figure resubmission:**

**Reproducibility:**

To enhance the reproducibility of your results, we recommend that authors of applicable studies deposit laboratory protocols in protocols.io, where a protocol can be assigned its own identifier (DOI) such that it can be cited independently in the future. Additionally, PLOS ONE offers an option to publish peer-reviewed clinical study protocols. Read more information on sharing protocols at https://plos.org/protocols?utm_medium=editorial-email&utm_source=authorletters&utm_campaign=protocols

Attachment Submitted filename: Review report_ (Dr Nguyen).pdf

# Author comment: Author response to Decision Letter 2

**DOI:** 10.1371/journal.pdig.0001690.r004

22 May 2026

Attachment Submitted filename: Response_to_reviewers_auresp_2.docx

# Peer review: Decision Letter 2

**DOI:** 10.1371/journal.pdig.0001690.r005

**Authors:** Harry Hochheiser

2 Jul 2026

Response to ReviewersRevised Manuscript with Track ChangesManuscript**Journal Requirements:** **Additional Editor Comments (if provided):** **Reviewers' Comments:**

**Comments to the Author**

Reviewer #2: (No Response)

Reviewer #3: All comments have been addressed

**********

publication criteria?

Reviewer #2: Partly

Reviewer #3: Yes

**********

3. Has the statistical analysis been performed appropriately and rigorously?

Reviewer #2: No

Reviewer #3: Yes

**********

4. Have the authors made all data underlying the findings in their manuscript fully available (please refer to the Data Availability Statement at the start of the manuscript PDF file)?

The PLOS Data policy

Reviewer #2: Yes

Reviewer #3: Yes

**********

5. Is the manuscript presented in an intelligible fashion and written in standard English?

Reviewer #2: Yes

Reviewer #3: Yes

**********

**Reviewer #2:**  I thank the authors for a thorough and substantially improved revision. Most of the concerns I raised in the first round have been addressed at the level of the manuscript itself, not merely asserted: the introduction is now empirically grounded across multiple countries; the foundational models are properly cited; the dynamic/multi-level concept is defined; Table 1 carries references and a dimensional comparison; the new Table 5 maps the five themes against model constructs and now grounds a properly calibrated "uneven conceptual coverage" claim rather than the earlier over-statement; MMAT appraisal has been added with an influence-on-synthesis analysis; a descriptive overview (Table 4) and a stakeholder-implications table (Table 6) have been included; and the review has been reframed as a global synthesis. These are real improvements and I want to acknowledge them.

My remaining concerns are substantive enough to warrant a further round. They concern transparency, methodological framing, and claim consistency rather than the quality of the underlying data.

Protocol registration — disclosure must be accurate and dated. The Limitations acknowledge that amendments were made and describe them as "refinement of eligibility criteria and clarification of the analytical scope by removing the originally planned national contextualisation," stating these were "transparently documented in the PROSPERO record." This understates what occurred. The registered review was Malaysia-specific in its title, objectives, country and scope, and remained so through the second protocol version; the global reframing — including the new title, revised objectives and population, and the addition of MMAT appraisal — was introduced in a later amendment dated well after searching and screening were complete. I ask the authors to disclose this sequence explicitly and with dates in the Methods and Limitations: that the original registered scope was national; that the global scope and the appraisal approach were introduced by post-registration amendment after the corpus had been screened; and the rationale for that change. Relatedly, "prospective registration" is currently listed among the review's strengths (Methods/Discussion); given the amendment history, this should be removed or carefully qualified. The point here is not the amendment itself — protocols may legitimately be amended — but that the manuscript's account should match the registry's timeline so a reader can interpret the registration correctly.

Methodological coherence — the analysis is labelled reflexive thematic analysis but is operationalised as framework/template analysis. The manuscript cites Braun and Clarke's reflexive TA, yet the procedures described are predominantly deductive and draw on incompatible traditions: open coding begins from a priori "sensitising domain descriptors" fixed to the six domains; "axial coding" and "axial-informed" mapping (grounded theory) appear throughout; completion is defined by saturation ("no substantively new concepts emerged"); and rigour is framed through Lincoln and Guba's credibility/dependability/confirmability criteria. Several of these are explicitly argued against in the reflexive-TA literature. I recommend reconciling this in one direction: either commit to reflexive TA and remove the saturation, axial-coding and trustworthiness-criterion scaffolding, or — which fits what is actually described — relabel the approach as framework- or matrix-based qualitative synthesis. The latter is the lower-burden fix and additionally dissolves the apparent tension around single-coder analysis, since framework synthesis accommodates it.

"Sustained / continued use" claims persist despite removal of the construct. The term "sustained use" was removed from the title and abstract, but the Results and Discussion repeatedly assert "continued use," "continued adoption," "long-term engagement," DHTs being "more sustainable," and adoption being "sustained beyond pilot implementation." These reintroduce the construct the manuscript set aside. Because the corpus is largely cross-sectional and use-duration was not consistently measured, I ask the authors either to soften these to initial-adoption language consistent with the evidence, or to define and own the construct explicitly. The decision should be applied consistently across abstract, results and discussion.

Acronym collision. "Thematic Analysis Matrix (TAM)" collides with the Technology Acceptance Model, which is central to this paper and recurs as TAM and TAM-HBM/TAM-TPB/TAM-SCT. Please rename the analytic matrix (e.g., "TA Matrix") and reserve TAM for the model, consistently across the abstract, Methods and the S6 appendix.

Minor consistency and copyediting. The factor taxonomy drifts between six domains and additional "organisational" and "equity-related" labels — please fix on one scheme. Table 1 and Table 5 are both model-comparison tables (dimensions vs themes); a one-line signpost distinguishing their purpose would prevent the appearance of redundancy. Table 6 omits developers and researchers, who are named as beneficiaries in the prose. And several copyedits: "June, 18 2025" (date format); "such as such as" (Methods); the full-width comma in "feedback, " (Results); the broken "poor 'webside: manner" (Results); and "app abandonment 30 to 90 days" (missing "within").

Finally, an optional suggestion for strengthening rather than a required change. The Discussion states practical implications for developers and implementers, but delivers them generically ("user-centred design, cultural relevance, accessibility"). The themes themselves contain concrete, actionable material — localisation and multilingual interfaces, low-bandwidth/offline operation, transparent and itemised costs, simple actionable feedback over analytics dumps, transparent privacy controls and explainable AI with human oversight, EHR interoperability and workflow fit, visible clinical-validation signals; and on the avoid side, notification overload, top-down design without user involvement, opaque automated decisions, hidden costs, and one-size-fits-all designs that exclude older, disabled and rural users. Distilling these into a concise "design considerations: incorporate / avoid" synthesis would deliver more value to this journal's implementation-oriented readership and would let the practical contribution match the strength of the conceptual one. I offer this as encouragement; it is consistent with the review's data and not a redesign of the study.

**Reviewer #3:**  The authors have adequately addressed all my earlier comments on a point-by-point basis. The manuscript has been substantially improved and strengthened through the revisions. In view of these improvements, I recommend the manuscript for publication in its present form.

**********

what does this mean?). If published, this will include your full peer review and any attached files.

**Do you want your identity to be public for this peer review?**  If you choose “no”, your identity will remain anonymous but your review may still be made public.

For information about this choice, including consent withdrawal, please see our Privacy Policy

Reviewer #2: No

Reviewer #3: No

**********

**Figure resubmission:**

**Reproducibility:**

To enhance the reproducibility of your results, we recommend that authors of applicable studies deposit laboratory protocols in protocols.io, where a protocol can be assigned its own identifier (DOI) such that it can be cited independently in the future. Additionally, PLOS ONE offers an option to publish peer-reviewed clinical study protocols. Read more information on sharing protocols at https://plos.org/protocols?utm_medium=editorial-email&utm_source=authorletters&utm_campaign=protocols

Attachment Submitted filename: Review report 2_ (Dr Nguyen).pdf

# Author comment: Author response to Decision Letter 3

**DOI:** 10.1371/journal.pdig.0001690.r006

12 Jul 2026

Attachment Submitted filename: Response_to_reviewers_auresp_3.docx

# Peer review: Decision Letter 3

**DOI:** 10.1371/journal.pdig.0001690.r007

**Authors:** Harry Hochheiser

10 Aug 2026

Advancing foundational models in digital health technology adoption: A systematic literature review of multidisciplinary factors

PDIG-D-25-00816R3

Dear Ms Ng,

We are pleased to inform you that your manuscript 'Advancing foundational models in digital health technology adoption: A systematic literature review of multidisciplinary factors' has been provisionally accepted for publication in PLOS Digital Health.

Before your manuscript can be formally accepted you will need to complete some formatting changes, which you will receive in a follow-up email from a member of our team.

Please note that your manuscript will not be scheduled for publication until you have made the required changes, so a swift response is appreciated.

IMPORTANT: The editorial review process is now complete. PLOS will only permit corrections to spelling, formatting or significant scientific errors from this point onwards. Requests for major changes, or any which affect the scientific understanding of your work, will cause delays to the publication date of your manuscript.

If your institution or institutions have a press office, please notify them about your upcoming paper to help maximize its impact. If they'll be preparing press materials, please inform our press team as soon as possible -- no later than 48 hours after receiving the formal acceptance. Your manuscript will remain under strict press embargo until 2 pm Eastern Time on the date of publication. For more information, please contact digitalhealth@plos.org.

Thank you again for supporting Open Access publishing; we are looking forward to publishing your work in PLOS Digital Health.

Best regards,

Harry Hochheiser

Section Editor

PLOS Digital Health

***********************************************************

**Additional Editor Comments (if provided):**

Thanks for these changes. The reviewers have agreed that this paper is ready for acceptance. Please consider reviewer 3's minor suggestions in your final submission.

**Reviewer Comments (if any, and for reference):**

Reviewer's Responses to Questions

**Comments to the Author**

Reviewer #2: All comments have been addressed

**********

publication criteria?

Reviewer #2: Yes

**********

3. Has the statistical analysis been performed appropriately and rigorously?

Reviewer #2: Yes

**********

4. Have the authors made all data underlying the findings in their manuscript fully available (please refer to the Data Availability Statement at the start of the manuscript PDF file)?

The PLOS Data policy

Reviewer #2: Yes

**********

5. Is the manuscript presented in an intelligible fashion and written in standard English?

Reviewer #2: Yes

**********

Reviewer #2: Dear Authors,

Congratulations on bringing this manuscript to publication standard. Over three rounds of revision, you have demonstrated exceptional commitment to scientific transparency and methodological rigor.

The improvements are substantial: the full PROSPERO chronology with specific dates, the honest acknowledgment of protocol amendments, the comprehensive MMAT appraisal, and Table 4's systematic theory-practice comparison have transformed this into a strong contribution to the digital health adoption literature.

Your willingness to openly document the evolution from a Malaysia-focused to a global review—rather than obscuring it—exemplifies good scientific practice. This transparency strengthens rather than weakens the work.

One minor edit required for consistency:

The five themes are numbered in both the Introduction and Discussion sections but not in the Author Summary (lines 61-64). Please number them for internal consistency: "(1) access, equity and affordability; (2) usability, engagement and user empowerment; (3) trust, privacy and governance; (4) integration, workforce and sustainability; and (5) clinical effectiveness and quality of care."

Recommendation: ACCEPT

Thank you for your persistence and responsiveness throughout this process.

**********

what does this mean?). If published, this will include your full peer review and any attached files.

**Do you want your identity to be public for this peer review?**  If you choose “no”, your identity will remain anonymous but your review may still be made public.

For information about this choice, including consent withdrawal, please see our Privacy Policy

Reviewer #2: **Yes:**  Álvaro Alaor Silva, Independent Researcher, Brasília, Brazil

**********
